# 1,2,3-Triazolo-Bridged Click Coupling of Pinane-Based Azidodiol Enantiomers with Pyrimidine- and Purine-Based Building Blocks: Synthesis, Antiproliferative, and Antimicrobial Evaluation

**DOI:** 10.3390/ijms262311705

**Published:** 2025-12-03

**Authors:** Dima Depp, Kitti Tari, András Szekeres, Adriána Kovács, István Zupkó, Zsolt Szakonyi

**Affiliations:** 1 Institute of Pharmaceutical Chemistry, University of Szeged, Eötvös utca 6, H-6720 Szeged, Hungary; dima.depp@hotmail.com; 2 Department of Biotechnology and Microbiology, University of Szeged, Közép fasor 52, H-6726 Szeged, Hungary; kittitari2000@gmail.com (K.T.); andras.j.szekeres@gmail.com (A.S.); 3 Institute of Pharmacodynamics and Biopharmacy, University of Szeged, Eötvös utca 6, H-6720 Szeged, Hungary; kovacs.adriana.judit@szte.hu (A.K.); zupko.istvan@szte.hu (I.Z.)

**Keywords:** aminodiol, pinane, monoterpene, triazole, click reaction, antiproliferative activity, antimicrobial, diaminopyrimidine, purine-based

## Abstract

Key intermediate azidodiols were synthesized according to literature from commercially available (+)- and (−)-α-pinene in a four-step sequence, including epoxidation with *m*CPBA, allylic rearrangement, a second epoxidation and, finally, a regioselective azidolysis of the resulting epoxide by sodium azide, yielding the enantiomerically pure azidodiols. The pyrimidine-based alkyne building blocks were prepared from dichloropyrimidines following our method reported previously, while the purine-containing alkyne analogues were synthesized in a procedure of two or three steps. Click reactions were carried out in the presence of Cu(OAc)_2_ and sodium ascorbate. The obtained pinane-coupled 2,4-diaminopyrimidines were screened for antiproliferative activity by MTT assay on HeLa, MD231, SiHa, MCF-7, and A2780 human cancer cell lines compared with fibroblast cells (NIH/3T3), on Gram-positive and Gram-negative pathogenic bacteria, and two yeasts, and the SAR was explained in detail. The prepared compounds showed moderate antiproliferative activity. While the starting azidodiols **(+)-2** and **(−)-2** exhibited excellent and selective antibacterial activities against *S. aureus* with a moderate antimycotic effect on *C. krusei*, only the (−)-enantiomer was active against *P. aeruginosa*. In a similar manner, most pyrimidine and purine derivatives also expressed moderate antimycotic effect against *C. krusei*. One of the purine-based derivatives **(−)-30** possessed remarkable and selective antibacterial effect against *P. aeruginosa*.

## 1. Introduction

Antimicrobial resistance (AMR) and cancer are among the greatest global health concerns nowadays. These threats highlight the urgent need for new therapeutic strategies and pharmacological agents. According to the World Health Organization (WHO) report, AMR directly caused an estimated 1.27 million deaths and contributed to 4.95 million others in 2019. These numbers validate how it is making infections increasingly difficult to treat, thereby undermining the safety of essential medical procedures such as surgery. Similarly, cancer remains a leading cause of death worldwide. Over 20 million new cases and nearly 10 million deaths have been reported in 2022. The major challenges in therapy include treatment resistance and tumour heterogeneity, which further complicate effective management of the disease. These alarming figures underscore the critical need to discover and develop new compounds that could serve as potential candidates for future drug development [1,2,3].

Natural compounds have not only implied an inspiration in modern pharmaceutical research but also served as readily available raw materials in large quantities, enabling cost-effective and environmentally friendly syntheses. Terpenes are also of outstanding importance among such natural compounds. Of these, the monoterpene skeleton α-pinene is particularly attractive, because both enantiomers are cheaply available, making them useful starting materials for enantioselective syntheses. In recent years, we reported the stereoselective syntheses and applications of pinane-based bi- and trifunctional building blocks such as β-amino acids, 1,3-aminoalcohols, aminodiols, and diaminoalcohols with remarkable pharmacological and catalytic activities [4,5,6,7,8].

On the other hand, the role of nitrogen heterocycles in medicinal chemistry is unique and prominent [9,10]. Within this class, 2,4-diaminopyrimidines possess outstanding importance, as they have well-established roles in cancer chemotherapy [11,12,13], such as methotrexate or in the design of antimicrobial agents, for example, trimethoprim [14,15]. These examples underscore their central position in therapeutic development and drug discovery [16]. In parallel, purine heterocycles are also noteworthy as they form the structural foundation of nucleic acids and they are found in many clinically important drugs. These include antiviral agents (e.g., acyclovir) [17] and anticancer therapies (e.g., mercaptopurine) [18]. In addition, some purine derivatives are actively being explored for their antimicrobial potential [19].

As a continuation of our systematic research work combining the structural diversity of terpenes with the pharmacological potential of nitrogen heterocycles, our aim was to explore new chemical species and create compounds with enhanced biological activities, combining the monoterpenic α-pinane skeleton with 2,4-diaminopyrimidine and purine moieties [4,20].

## 2. Results and Discussion

### 2.1. Synthesis of Pinan-Based Azides

**Scheme 1:**
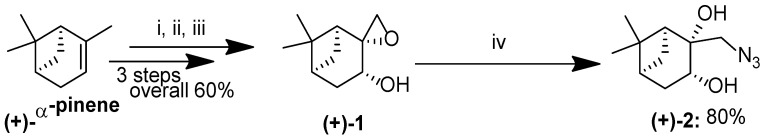
Synthesis of pinane-based azidodiols. (**i**) *m*CPBA, NaHCO_3_, DCM, 10 °C, 1.5 h, 82%; (**ii**) Al(O-iPr)_3_, dry toluene, reflux, 3 h, 70%; (**iii**) *m*CPBA, 5% NaH_2_PO_4_, 0 °C, 2 h, 60%; (**iv**) NaN_3_, 20% NH_4_Cl, EtOH/H_2_O, reflux, 36 h, 80%.

Azidodiol intermediates **(+)**- and **(−)-2** were obtained from commercially available (+)- and (−)-α-pinene, following a four-step sequence described in the literature [21,22]. The transformation involved the initial epoxidation of the pinene system with mCPBA, followed by allylic rearrangement, a subsequent epoxidation and, finally, a regioselective ring opening of the resulting epoxide with NaN_3_ in the presence of a catalytic amount of NH_4_Cl, furnishing the enantiomerically pure azidodiols in good yields (Scheme 1). All compounds were prepared in both enantiomeric forms, but for easier understanding, only the products obtained by starting from (+)-α-pinene are shown in Section 2.

### 2.2. Synthesis of Key-Intermediate Alkynes

**Scheme 2:**
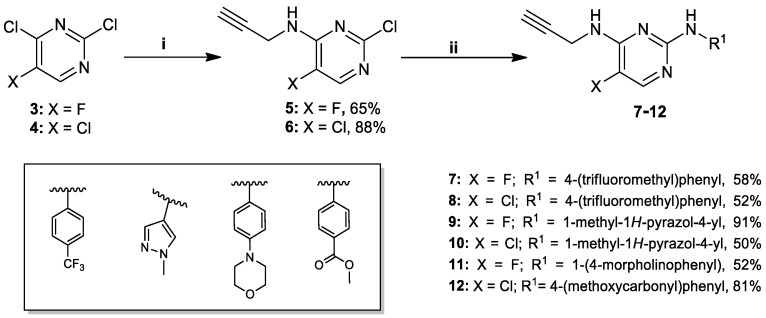
Synthesis of 2,4-diaminopyrimidine-derived alkynes **7**–**12**. (**i**) propargylamine (2 eq.), TEA (1 eq.), MeCN, 25 °C, 24 h for **5** and **6**; (**ii**) (a) 4-trifluoromethylaniline (1.1 eq.), EtOH, MW, 150 °C, 200 W, 19 bar, 2 h for **7** and **8**; (b) 1-methyl-1*H*-pyrazol-4-amine (1.1 eq.), EtOH, MW, 150 °C, 200 W, 19 bar, 2 h for **9** and **10**; (c) 4-morpholinoaniline (1 eq.), EtOH, MW, 100 °C, 200 W, 17 bar, 2 h for **11**; (d) methyl 4-aminobenzoate (1 eq.), EtOH, MW, 100 °C, 200 W, 17 bar, 2 h for **12**.

The first group of 2,4-diaminopyrimidine-based alkynes was synthesized according to the procedure published in our recent article [20]. The regioselective reaction of propargylamine with halogen-substituted pyrimidines **3** and **4**, followed by the reaction of the obtained 4-aminopyrimidines **5** and **6** with aromatic and heteroaromatic amines, resulted in 2,4-diaminopyrimidines **7**–**12** (Scheme 2).

**Scheme 3:**
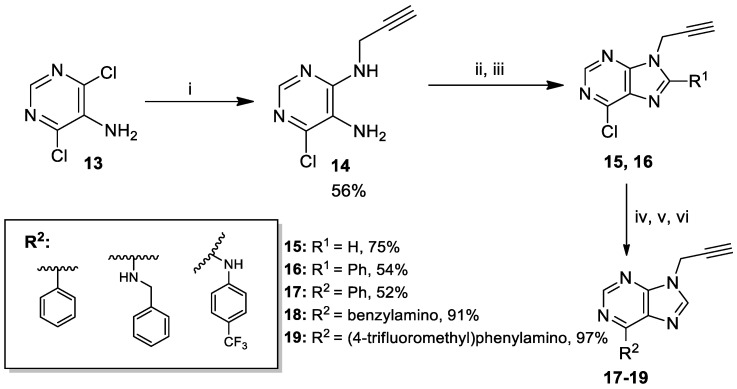
Synthesis of purine-derived alkynes **15**–**19**. (**i**) propargylamine (2 eq.), TEA (1 eq.), reflux, 24 h for **14**; (**ii**) TEOF (39 eq.), HCl (conc.) (3.5 eq.), RT, 24 h for **15**; (**iii**) benzoyl chloride (1 eq.), NH_4_Cl (6 eq.), POCl_3_ (75 eq.), toluene, 100 °C, 24 h for **16**; (**iv**) PhB(OH)_2_ (1.2 eq.), Pd(PPh_3_)_4_ (0.05 eq.), K_2_CO_3_ (2.2 eq.), dry toluene, argon atm., 100 °C, 12 h for **17**; (**v**) benzylamine (2 eq.), TEA (1.5 eq), MeCN, 40 °C, 24 h for **18**; (**vi**) 4-trifluoromethylaniline (1.5 eq.), 2 drops HCl (conc.), iPrOH, 85 °C, 4 h for **19**.

The purine-based alkynes **15**–**19** were prepared according to reported procedures [23,24], starting from 5-amino to 4,6-dichloropyrimidine and propargylamine, followed by ring closure of diamine **14** with triethyl orthoformate (TEOF) or benzoyl chloride. Compound **15** was finally substituted at position 6 via Suzuki coupling or in its reaction with aniline or benzylamine. The obtained new derivatives bearing 6-phenyl, 6-benzylamino, or *N*-(4-(trifluoromethyl)phenyl)-6-amino substituents on the 9*H*-purine scaffold are outlined in Scheme 3 [23,24,25].

### 2.3. Coupling of the Pinane Moiety with Alkyne-Functionalized Heterocycles via Click Reaction

Click chemistry enables the straightforward assembly of diverse molecular fragments in a modular fashion, similar to fitting puzzle pieces together, offering an efficient strategy for constructing a wide range of functional molecules and materials [26,27]. In particular, it is a very efficient method in the copper-catalyzed azide–alkyne cycloaddition (CuAAC) reaction, since it is a rapid, biocompatible, high-yielding reaction that allows the quick construction of complex molecules, accelerating drug discovery and optimization processes [28,29].

**Scheme 4:**
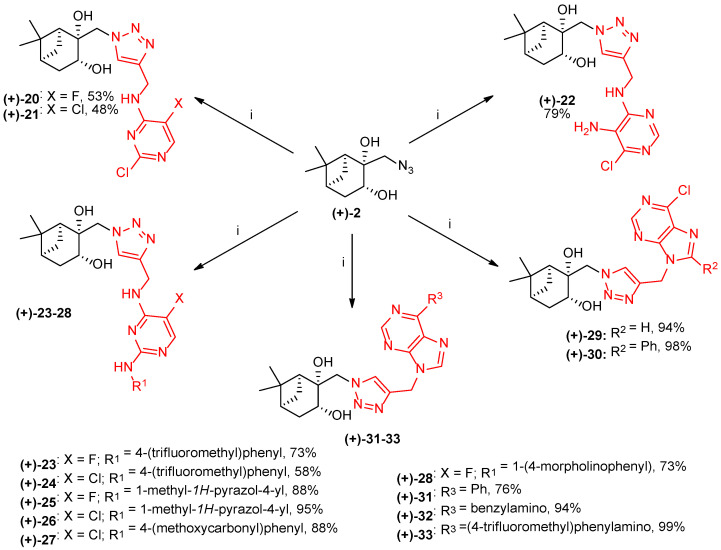
Synthesis of pinane-based hybrid compounds **20–33**. (i) Cu(OAc)_2_·H_2_O (0.05 eq.), Na ascorbate (0.1 eq.), alkyne (1.1 eq.), 45 °C, 48 h; *tert*-BuOH/H_2_O (2:1) for **(+)-20**, **(+)-21, (+)-23**–**26** and **(+)-28** or THF/H_2_O (2:1) for **(+)-22**, **(+)-27** and **(+)-29**–**33**.

In the synthesis of the new hybrid structures, both azides **(+)-2** and **(−)-2** underwent CuAAC reactions with the previously prepared alkynes **7**–**12** and **14**–**19**, using Cu(OAc)_2_·H_2_O and sodium ascorbate as catalysts, as illustrated in Scheme 4. Reactions were carried out under mild conditions, typically at 45 °C in a 2:1 mixture of *tert*-BuOH/H_2_O or THF/H_2_O, depending on the solubility of the alkyne. For the pyrimidine-based alkynes, either pure *tert*-BuOH or THF was suitable, as observed also in our previous work, resulting in **22**–**28** [20]. However, in contrast to the diterpene series we reported recently, no solubility problem or precipitation of the products was observed. For the purine-based alkynes, THF proved to be the optimal solvent choice for obtaining compounds **29**–**33**. All reactions proceeded in good to excellent yields, and the resulting compounds proved to be stable (Scheme 4).

### 2.4. In Vitro Antiproliferative Studies of Pinane-Based Diaminopyrimidines and Structure–Activity Relationship

**Figure 1:**
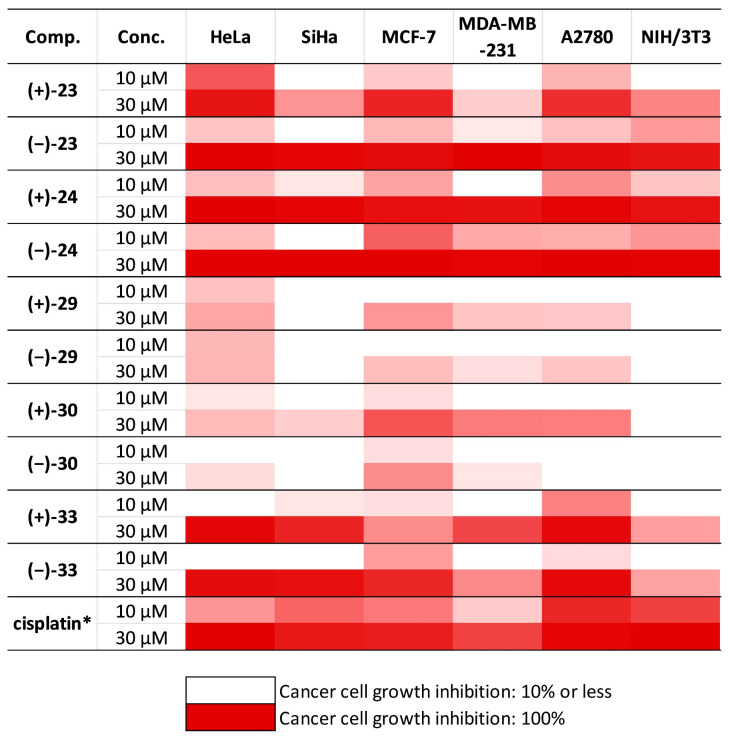
Selected antiproliferative properties of the prepared pyrimidine and purine derivatives against cancer cells and NIH/3T3 fibroblasts. Numeric values are given in Table S1 in the Supplementary Materials. *: data from reference [24].

The antiproliferative potential of the synthesized monoterpene-based compounds was assessed in vitro against a panel of human adherent cancer cell lines, including breast (MDA-MB-231, MCF-7), cervical (HeLa, SiHa), and ovarian (A2780) cells, using the MTT assay. Selected results are presented in Figure 1 and Table 1, while data for all examined products are presented in Table S1 in the Supplementary Materials. Analysis of the observed activities allowed preliminary insights into structure–activity relationships (SAR). Compounds bearing a monoamino-substituted pyrimidine unit (**20**–**22**) displayed minimal antiproliferative effects, which is in accordance with our previous results with other pinane-based amino alcohols and diols bearing similar monoaminopyrimidine substituents, but without the triazole ring linker. [4].

In the case of 2,4-diaminopyrimidine-derived compounds **23**–**28**, the activities ranged from mild to moderate. The main factors influencing activity included the type of halogen substituent on the pyrimidine ring, the nature of the ring attached to the pyrimidine core, and the type of substituent group present on the phenyl ring connected to the pyrimidine unit. Compared with our previous results with the directly diol-type pinane-condensed diaminopyrimidines, the introduction of the methylene-triazole linker increased the antiproliferative activity [4].

The most active derivatives were those bearing a trifluoromethyl group at the *para* position of the aniline substituent. Notably, the type of halogen substituent on the pyrimidine ring also played an important role: 5-fluoro-substituted **(+)-23** and **(−)-23** showed good antiproliferative activity, in particular, against HeLa adenocarcinoma cells. Meanwhile, 5-chloro analogues **(+)-24** and **(−)-24** had a more inhibitory effect toward both estrogen receptor-positive (ER+) breast cancer cells (MCF-7). The cancer selectivity of the most potent derivatives, however, is limited, as indicated by calculated selectivity indices (SIs, the ratios of the IC_50_ values obtained on fibroblasts and cancer cells) that are less than or close to 1. Furthermore, both the 5-chloro and 5-fluoro derivatives exhibited only mild effects against A2780 ovarian cancer cells (Figure 1, Table 1 and Table S1).

These observations are consistent with earlier findings by our group [4]. In another study, wherein we employed the diterpene *allo*-gibberic acid scaffold instead of the pinane system to prepare similar 2,4-diaminopyrimidine hybrids [25], we observed a comparable trend. In that work, compounds bearing 4-trifluoromethylphenyl moieties either linked through a triazole bridge or a flexible –CH_2_–CH_2_– spacer proved to be highly effective, albeit with little selectivity. Comparing the enantiomers, the stereochemistry also appeared to play a weak role, as the (−)-enantiomers (**(−)-23** and **(−)-24**) were generally more effective against MDA-MB-231 and MCF7 cells than their (+)-counterparts.

The results indicate that the type of substituent at the *para* position of the phenyl ring also influenced cell growth inhibition, which decreased notably when the trifluoromethyl group was replaced with a methyl ester function (**(+)-27** and **(−)-27**). This reduced activity may be explained by the lower lipophilicity of the methyl ester moiety compared to that of the CF_3_ group [30]. In addition, the ester functionality might be hydrolyzed by intracellular esterase enzymes, further diminishing its contribution to activity [31]. Similarly, the introduction of the 4-morpholine group (**(+)-28** and **(−)-28**), which is even more hydrophilic than the methyl ester, led to an even greater reduction in activity across nearly all tested cell lines.

These results also indicate that compounds bearing a 1-methyl-1*H*-pyrazol-4-yl ring (**25** and **26**) exhibited very low activity, consistent with our previous observation [25].

Considering the purine-hybrid compounds, they exhibited very weak antiproliferative activity in both cancerous and non-cancerous cells, except for two compounds that showed only moderate effects. Nevertheless, subtle differences in their inhibition patterns provide valuable clues for developing further structure–activity relationship (SAR) insights. For example, comparing **(+)-22** and **(−)-22** with their cyclic analogues (**(+)-29** and **(−)-29**), all four compounds showed similar weak activity against HeLa cells; however, the ring closure clearly enhanced activity against MCF-7 and MDA-MB-231 cells. In addition, introducing a phenyl substituent at position 8 of the purine ring (**(+)-30** and **(−)-30**), formed derivatives selectively improved the activity of the (+)-enantiomer, which inhibited MCF-7, MDA-MB-231, and A2780 cells at a 30 μM concentration. In contrast, substitution at C6 with a phenyl group (**(+)-31** and **(−)-31**) diminished the activity of the (+)-enantiomer at the same concentration, while both (−)-enantiomers remained inactive. In a similar manner, incorporating a benzylamine moiety at C6 did not improve the activity either. The most promising members of this series were **(+)-33** and **(−)-33**, bearing an amino-4-(trifluoromethyl)phenyl substituent at C6. These compounds showed weak to moderate activity against both MCF-7 and NIH/3T3 cells. These findings are consistent with previous reports indicating that 1,4-disubstituted 1,2,3-triazolylpurines generally display low cytotoxicity [32,33]. Consequently, further investigation of these molecules is needed, particularly regarding their structural optimization and pharmacological evaluation.

### 2.5. In Vitro Studies of Antibacterial Effects of 4-Amino- and 2,4-Diaminopyrimidine and Purine Derivatives

**Figure 2:**
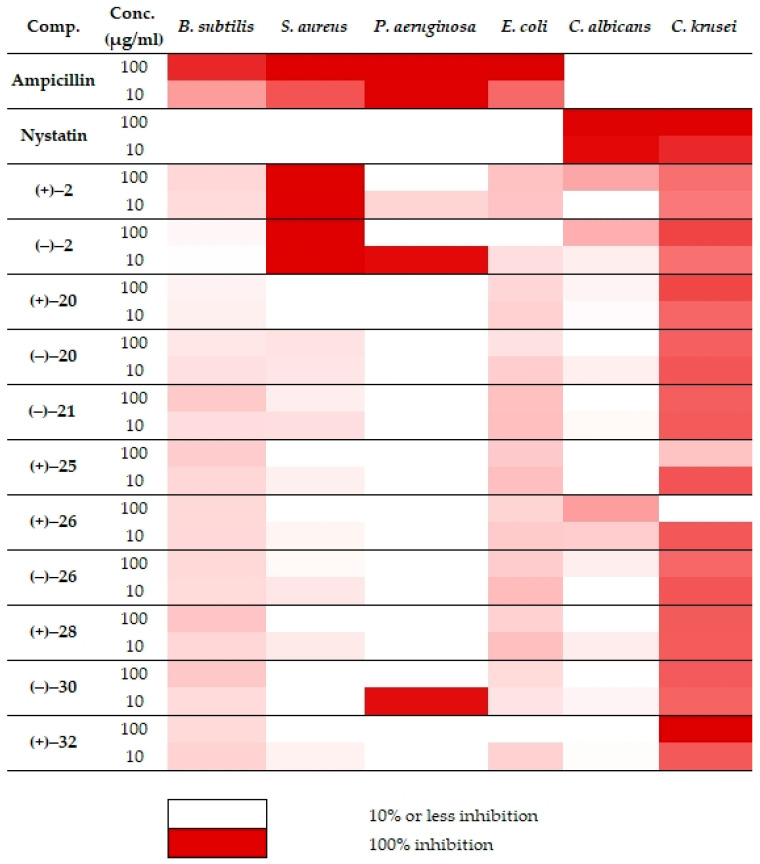
Selected antimicrobial properties of the prepared pyrimidine and purine derivatives against both Gram-negative and Gram-positive bacteria as well as yeast strains. Numeric values are given in Table 2 and Table S2 in the Supplementary Materials.

Pyrimidine and purine-based compounds, including benzothiazole derivatives, substituted adenines and aminopurines, exerted remarkable antimicrobial activities against several bacteria and yeasts [19,34]; therefore, antimicrobial activities of the analogues were tested against two Gram-positive and two Gram-negative bacteria, as well as two yeasts (Figure 2, Table 2 and Table S2). Generally, almost all examined compounds show moderate to high inhibitory activities against *Bacillus subtilis* and *Escherichia coli*. The obtained results clearly show that 4-monoamino-substituted derivatives (**(+)-** and **(−)-20**, **(+)-** and **(−)-21**) have only weak antibacterial activity, but these compounds possess remarkable antifungal activity against *Candida krusei*. In turn, the introduction of a primary amino function at position 5 (**22**) caused moderate antibacterial activity against *B. subtilis*. Introduction of an amino function at position 2 of the pyrimidine ring practically did not increase the antibacterial effect, but these compounds showed moderate antifungal activity against *C. krusei*. Similar results were obtained in the case of purine-based compounds (**29**–**33**) except compound **(−)-30**, which possessed excellent antibacterial activity against *P. aeruginosa* in 10 μg/mL concentration.

Since there are several examples of the antibacterial, antifungal or even antiviral activities of alicyclic or heterocyclic compounds bearing azido function [20,35,36,37,38], our azidodiol intermediates **(+)-** and **(−)-2** were also tested. Interestingly, they showed remarkable and selective antimicrobial activity against both Gram-positive and Gram-negative bacteria and a similar moderate activity against yeasts. Remarkable differences were observed between the two enantiomers. Namely, compound **(+)-2** showed selective inhibition against *S. aureus*, while **(−)-2** was effective against both *S. aureus* and *P. aeruginosa* in 10 μg/mL. It must also be mentioned that these compounds did not show cytotoxicity against human fibroblast cells (NIH/3T3, see Table S1 in Supplementary Materials). Compering with our previous results with the directly diol-type pinane-condensed diaminopyrimidines, the introduction of methylene-triazole linker increased the antifungal activity [4].

## 3. Materials and Methods

### 3.1. General Methods

Commercially available reagents were used as obtained from suppliers (Novochem Co., Ltd., 1089 Budapest, Hungary, Orczy út 6.; Merck Ltd., Budapest, Hungary; and VWR International Ltd., Debrecen, Hungary), while solvents were dried according to standard procedures. Chromatographic separations and monitoring of reactions were carried out on Merck Kieselgel 60 (Merck Ltd., Budapest, Hungary). Optical rotations were measured in MeOH at 20 °C with a PerkinElmer 341 polarimeter (PerkinElmer Inc., Shelton, CT, USA). Melting points were determined with a Kofler apparatus (Nagema, Dresden, Germany). HRMS flow injection analysis was performed with a Thermo Scientific Q Exactive Plus hybrid quadrupole-Orbitrap (Thermo Fisher Scientific, Waltham, MA, USA) mass spectrometer coupled to a Waters Acquity I-Class UPLC™ (Waters, Manchester, UK). 1H-, 13C J-MOD-, and 19F-NMR spectra were recorded on a Bruker Avance DRX 500 spectrometer (Bruker Biospin, Karlsruhe, Baden-Württemberg, Germany) [500 MHz (1H), 125 MHz (13C J-MOD), and 470 MHz (19F) δ = 0 (TMS)]. Chemical shifts are expressed in ppm (δ) relative to TMS as an internal reference. J values are given in Hz. All ^1^H-, ^13^C, J-MOD-, ^19^F-NMR, COSY, NOESY, 2D-HMBC, and 2D-HMQC and HRMS spectra are available in the Supporting Information file, as Figures S1–S218; Schemes S1 and S2; Tables S1 and S2.

### 3.2. Starting Materials

(+)- and (−)-α-Pinene were obtained from Merck KGaA, Darmstadt, Germany. The preparation of azidodiol intermediates **(+)**- and **(−)-2** was accomplished according to literature methods, with spectroscopic data identical to those reported therein [22]. Similarly, intermediates **7**–**12**, **14**, **15** and **17** were prepared via methods reported in the literature and in our earlier publications [20,23,25].

### 3.3. Synthesis of New Compounds

#### 3.3.1. 6-Chloro-8-phenyl-9-(prop-2-yn-1-yl)-9H-purine **16**

To a solution of **14** (0.15 g, 0.82 mmol) in dry toluene (7 mL) NH_4_Cl was added (6 eq., 0.26 g), followed by the addition of benzoyl chloride (1 eq., 96 μL). The reaction mixture was heated at 100 °C for 2 h, then it was cooled, POCl_3_ (5.6 mL) was added and the mixture was heated again to 100 °C for 24 h [23]. After stopping the reaction and cooling to room temperature, the reaction mixture was slowly added to ice water dropwise. Afterwards, pH was adjusted to 7–8 by the careful addition of ammonia water (25%), and the mixture was extracted with EtOAc (3 × 25 mL). The combined organic phase was dried over Na_2_SO_4_, filtered, and concentrated under reduced pressure. The product was purified by column chromatography using *n*-hexane/EtOAc (2:1). Yield: 0.12 g, 54%; white crystals; m.p.: 189–191 °C; ^1^H NMR (500 MHz, DMSO-d_6_) δ (ppm): 3.54 (1H, t, *J* = 2.4 Hz), 5.22 (2H, d, *J* = 2.4 Hz), 7.71–7.65 (3H, m), 8.01 (2H, dd, *J* = 2.1, 7.4 Hz), 8.87 (1H, s); ^13^C NMR (125 MHz, DMSO-d_6_) δ (ppm): 34.4, 77.2, 78.1, 127.4, 128.5, 129.6 (2C), 130.9, 131.9 (2C), 149.0, 152.2, 153.6, 155.8; HRMS (ESI): *m*/*z* calcd. for C_14_H_10_ClN_4_ [M + H]^+^: 269.05885. Found: 269.05847.

#### 3.3.2. *N*-Benzyl-9-(prop-2-yn-1-yl)-9*H*-purin-6-amine **18**

To a solution of **15** (0.10 g, 0.5 mmol) in acetonitrile (10 mL), (2 eq., 0.11 mL) of benzylamine and (1.5 eq., 0.1 mL) of TEA were added. The reaction mixture was stirred for 24 h at 40 °C [25]. After the evaporation of the solvent, the crude residue was dissolved in 30 mL EtOAc and washed with NaHCO_3_. Then, the organic layer was dried over Na_2_SO_4,_ followed by filtration and evaporation. The product was purified by column chromatography using DCM/MeOH (39:1). Yield: 0.12 g, 91%; white crystals; m.p.: 146.5–148.5 °C; ^1^H NMR (500 MHz, CDCl_3_) δ(ppm): 2.51 (1H, t, *J* = 2.3 Hz), 4.88 (2H, s), 4.97 (2H, d, *J* = 2.3 Hz), 6.19 (1H, s), 7.40–7.27 (5H, m), 7.92 (1H, s), 8.44 (1H, s); ^13^C NMR (125 MHz, CDCl_3_) δ (ppm): 33.0 (2C), 74.9, 76.0, 127.5 (2C), 127.8, 128.7 (2C), 138.4 (2C), 138.9, 153.5, 154.8 (2C); HRMS (ESI): *m*/*z* calcd. for C_15_H_14_N_5_ [M + H]^+^: 264.12437. Found: 264.12377.

#### 3.3.3. 9-(Prop-2-yn-1-yl)-*N*-(4-(trifluoromethyl)phenyl)-9*H*-purin-6-amine **19**

To a solution of **15** (0.10 g, 0.5 mmol) in isopropyl alcohol (5 mL), (1.5 eq., 0.1 mL) of 4-(trifluoromethyl)aniline and two drops of conc. HCl was added. The reaction mixture was stirred for 4 h at 85 °C [23]. After cooling to room temperature, the saturated solution of NaHCO_3_ (10 mL) was added, and extraction was made with DCM (3 × 30 mL). The organic layer was dried over Na_2_SO_4_, filtered, and concentrated. The product was purified by column chromatography using DCM/MeOH (39:1). Yield: 0.16 g, 97%; white crystals; m.p.: 161–163 °C; ^1^H NMR (500 MHz, CDCl_3_) δ (ppm): 2.56 (1H, dd, *J* = 2.5, 2.5 Hz), 5.03 (2H, d, *J* = 2.5 Hz), 7.64 (2H, d, *J* = 8.5 Hz), 7.92 (1H, s), 7.98 (2H, d, *J* = 8.5 Hz), 8.10 (1H, s), 8.61 (1H, s)); ^13^C NMR (125 MHz, CDCl_3_) δ (ppm): 33.2, 75.3, 75.7, 119.5 (2C), 121.0, 123.2, 125.0 (q, *J* = 29.4 Hz), 126.3 (q, *J* = 3.7 Hz), 140.2, 141.8, 149.5, 151.8, 152.9; ^19^F NMR (470 MHz, CDCl_3_) δ (ppm): −61.9; HRMS (ESI): *m*/*z* calcd. for C_15_H_11_F_3_N_5_ [M + H]^+^: 318.09611. Found: 318.09533.

### 3.4. General Procedure for Preparation of 1,2,3-Triazols by Click Reaction (***20*–*33***)

To a solution of azide **(+)-2** or **(−)-2** (0.05 g, 0.237 mmol) in a mixture of *tert*-BuOH/H_2_O (2:1, 12 mL), or THF/H_2_O (2:1, 12 mL), Cu(OAc)_2_·H_2_O (0.05 eq.), sodium ascorbate (0.1 eq.), and the appropriate acetylene derivatives (**5**–**12, 14**–**19**) (1.1 eq., 0.26 mmol) were added. The mixture was stirred for 48 h at 45 °C. Then, the organic solvent was evaporated, and the residue was dissolved in water (10 mL) and extracted with DCM (3 × 30 mL). The organic phase was dried over Na_2_SO_4_ and evaporated at low pressure, and the crude product was purified by column chromatography on silica gel.

#### 3.4.1. (1*S*,2*R*,3*R*,5*S*)-2-((4-(((2-Chloro-5-fluoropyrimidin-4-yl)amino)methyl)-1*H*-1,2,3-triazol-1-yl)methyl)-6,6-dimethylbicyclo [3.1.1]heptane-2,3-diol **(+)-20**

The reaction was accomplished starting from **(+)-2** and alkyne **5** in *tert*-BuOH/H_2_O (2:1) according to the general procedure. The product was purified by column chromatography on silica gel with DCM/MeOH (19:1). Yield: 0.050 g, 53%; white crystals; m.p.: 189–191 °C; ${\left[\alpha \right]}_{D}^{20}$ = +4.0 (*c* 0.1, MeOH); ^1^H NMR (500 MHz, DMSO-d_6_) δ (ppm): 1.04 (3H, s), 1.16 (3H, s), 1.29 (1H, d, *J* = 10.1 Hz), 1.52 (1H, t, *J* = 5.8 Hz), 1.59 (1H, dd, *J* = 3.0, 13.7 Hz), 1.83 (1H, s), 2.06–1.98 (1H, m), 2.43–2.34 (1H, m), 4.00–3.93 (1H, m), 4.27 (1H, d, *J* = 14.0 Hz), 4.33 (1H, d, *J* = 14.0 Hz), 4.57 (1H, s), 4.59 (2H, d, *J* = 5.8 Hz), 5.36 (1H, d, *J* = 6.3 Hz), 7.88 (1H, s), 8.11 (1H, d, *J* = 3.3 Hz), 8.69 (1H, t, *J* = 5.8 Hz); ^13^C NMR (125 MHz, DMSO-d_6_) δ (ppm): 24.2, 27.8, 28.0, 36.0, 38.0, 38.5, 40.4, 48.2, 58.5, 64.8, 74.5, 125.1, 140.4 (d, *J* = 20.4 Hz), 143.6, 145.8 (d, *J* = 256.5 Hz), 153.7 (d, *J* = 13.4 Hz), 153.9 (d, *J* = 2.8 Hz); ^19^F NMR (470 MHz, DMSO-d_6_) δ (ppm): −157.14; HRMS (ESI): *m*/*z* calcd. for C_17_H_23_ClFN_6_O_2_ [M + H]^+^: 397.15550. Found: 397.15438.

The (1*R*,2*S*,3*S*,5*R*)-enantiomer **(−)-20** was synthesized under conditions used for **(+)-20**; ${\left[\alpha \right]}_{D}^{20}$ = −4.0 (*c* 0.1, MeOH); all the spectroscopic data and the m.p. were similar to those for the (1*R*,2*S*,3*S*,5*R*)-enantiomer. HRMS (ESI): *m*/*z* calcd. for C_17_H_23_ClFN_6_O_2_ [M + H]^+^: 397.15550. Found: 397.15446.

#### 3.4.2. (1*S*,2*R*,3*R*,5*S*)-2-((4-(((2,5-Dichloropyrimidin-4-yl)amino)methyl)-1*H*-1,2,3-triazol-1-yl)methyl)-6,6-dimethylbicyclo[3.1.1]heptane-2,3-diol **(+)-21**

The reaction was accomplished starting from **(+)-2** and alkyne **6** in *tert*-BuOH/H_2_O (2:1) according to the general procedure. The product was purified by column chromatography on silica gel with DCM/MeOH (39:1). Yield: 0.047 g, 48%; yellowish white crystals; m.p.: 70–73 °C; ${\left[\alpha \right]}_{D}^{20}$ = +3.0 (*c* 0.1, MeOH); ^1^H NMR (500 MHz, CDCl_3_) δ (ppm): 1.11 (3H, s), 1.31 (3H, s), 1.43 (1H, d, *J* = 10.6 Hz), 1.67 (1H, dd, *J* = 5.2, 13.9 Hz), 1.98 (2H, d, *J* = 6.0 Hz), 2.30–2.23 (1H, m), 2.54 (1H, td, *J* = 5.9, 13.9 Hz), 2.62 (1H, d, *J* = 5.3 Hz), 3.95 (1H, s), 4.19 (1H, d, *J* = 13.8 Hz), 4.47 (1H, td, *J* = 5.0, 10.0 Hz), 4.53 (1H, d, *J* = 13.8 Hz), 4.77 (2H, ddd, *J* = 5.6, 15.3, 21.0 Hz), 6.25 (1H, s), 7.78 (1H, s), 8.04 (1H, s); ^13^C NMR (125 MHz, CDCl_3_) δ (ppm): 24.3, 27.6, 27.7, 36.6, 37.5, 38.6, 40.5, 50.4, 60.0, 65.2, 74.5, 113.5, 125.2, 142.9, 153.8, 158.3, 158.5; HRMS (ESI): *m*/*z* calcd. for C_17_H_23_C_l2_N_6_O_2_ [M + H]^+^: 413.12595. Found: 413.12510.

The (1*R*,2*S*,3*S*,5*R*)-enantiomer **(−)-21** was synthesized analogously to **(+)-21**; ${\left[\alpha \right]}_{D}^{20}$ = −3.5 (*c* 0.1, MeOH); all the spectroscopic data and the m.p. were similar to those for the (1*R*,2*S*,3*S*,5*R*)-enantiomer. HRMS (ESI): *m*/*z* calcd. for C_17_H_23_C_l2_N_6_O_2_ [M + H]^+^: 413.12595. Found: 413.12515.

#### 3.4.3. (1*S*,2*R*,3*R*,5*S*)-2-((4-(((5-Amino-6-chloropyrimidin-4-yl)amino)methyl)-1*H*-1,2,3-triazol-1-yl)methyl)-6,6-dimethylbicyclo[3.1.1]heptane-2,3-diol **(+)-22**

The reaction was accomplished starting from **(+)-2** and alkyne **14** in THF/H_2_O (2:1) according to the general procedure. The product was purified by column chromatography on silica gel with CHCl_3_/MeOH (19:1) then increased to (9:1). Yield: 0.074 g, 79%; yellow crystals; m.p.: 94–97 °C; ${\left[\alpha \right]}_{D}^{20}$ = +9.0 (c 0.1, MeOH); ^1^H NMR (500 MHz, CDCl_3_) δ (ppm): 1.09 (3H, s), 1.30 (3H, s), 1.42 (1H, d, *J* = 10.5 Hz), 1.66 (1H, dd, *J* = 4.7, 13.8 Hz), 2.01–1.94 (2H, m), 2.29–2.21 (1H, m), 2.55–2.47 (1H, m), 4.14 (1H, d, *J* = 13.9 Hz), 4.43 (1H, dd, *J* = 5.0, 9.4 Hz), 4.52 (1H, d, *J* = 13.9 Hz), 4.71 (2H, d, *J* = 5.0 Hz), 5.97 (1H, s), 7.76 (1H, s), 8.03 (1H, s); ^13^C NMR (125 MHz, CDCl_3_) δ (ppm): 24.3, 27.6, 27.7, 36.6, 37.6, 38.6, 40.4, 50.4, 60.1, 64.8, 74.5, 122.5, 125.1, 142.3, 144.2, 148.9, 153.9; HRMS (ESI): *m*/*z* calcd. for C_17_H_25_ClN_7_O_2_ [M + H]^+^: 394.17582. Found: 394.17501.

The (1*R*,2*S*,3*S*,5*R*)-enantiomer **(−)-22** was synthesized analogously to **(+)-22**; ${\left[\alpha \right]}_{D}^{20}$ = −6.0 (*c* 0.1, MeOH); all the spectroscopic data and the m.p. were similar to those for the (1*R*,2*S*,3*S*,5*R*)-enantiomer. HRMS (ESI): *m*/*z* calcd. for C_17_H_25_ClN_7_O_2_ [M + H]^+^: 394.17582. Found: 394.17480.

#### 3.4.4. (1*S*,2*R*,3*R*,5*S*)-2-((4-(((5-Fluoro-2-((4-(trifluoromethyl)phenyl)amino)pyrimidin-4-yl)amino)methyl)-1*H*-1,2,3-triazol-1-yl)methyl)-6,6-dimethylbicyclo[3.1.1]heptane-2,3-diol **(+)-23**

The reaction was accomplished starting from **(+)-2** and alkyne **7** in *tert*-BuOH/H_2_O (2:1) according to the general procedure. The product was purified by column chromatography on silica gel with DCM/MeOH (19:1). Yield: 0.090 g, 73%; white crystals; m.p.: 105–108 °C; ${\left[\alpha \right]}_{D}^{20}$ = +7.0 (*c* 0.1, MeOH); ^1^H NMR (500 MHz, CDCl_3_) δ (ppm): 1.10 (3H, s), 1.29 (3H, s), 1.40 (1H, d, *J* = 10.6 Hz), 1.67–1.63 (1H, m), 1.99–1.92 (2H, m), 2.27–2.20 (1H, m), 2.55–2.48 (1H, m), 3.92 (1H, broad s), 4.15 (1H, d, *J* = 13.8 Hz), 4.45 (1H, dt, *J* = 4.9, 4.8 Hz), 4.51 (1H, d, *J* = 13.8 Hz), 4.77 (2H, dt, *J* = 5.7, 15.8 Hz), 5.67 (1H, t, *J* = 5.6 Hz), 7.16 (1H, s), 7.53 (1H, d, *J* = 8.6 Hz), 7.68 (3H, d, *J* = 7.1 Hz), 7.83 (1H, d, *J* = 3.0 Hz); ^13^C NMR (125 MHz, CDCl_3_) δ (ppm): 24.2, 27.5, 27.7, 36.3, 37.5, 38.6, 40.5, 50.4, 60.0, 65.2, 74.5, 117.8 (2C), 124.5, 126.1 (q, *J* = 3.8 Hz) (2C), 138.9, 139.1, 140.8, 142.8, 143.2, 143.9, 152.3 (d, *J* = 12.2 Hz), 155.0 (d, *J* = 2.9 Hz); ^19^F NMR (470 MHz, CDCl_3_) δ (ppm): −167.11, −61.69; HRMS (ESI): *m*/*z* calcd. for C_24_H_28_F_4_N_7_O_2_ [M + H]^+^: 522.22351. Found: 522.22254.

The (1*R*,2*S*,3*S*,5*R*)-enantiomer **(−)-23** was synthesized analogously to **(+)-23**; ${\left[\alpha \right]}_{D}^{20}$ = −6.5 (*c* 0.1, MeOH); all the spectroscopic data and the m.p. were similar to those for the (1*R*,2*S*,3*S*,5*R*)-enantiomer. HRMS (ESI): *m*/*z* calcd. for C_24_H_28_F_4_N_7_O_2_ [M + H]^+^: 522.22351. Found: 522.22137.

#### 3.4.5. (1*S*,2*R*,3*R*,5*S*)-2-((4-(((5-Chloro-2-((4-(trifluoromethyl)phenyl)amino)pyrimidin-4-yl)amino)methyl)-1*H*-1,2,3-triazol-1-yl)methyl)-6,6-dimethylbicyclo[3.1.1]heptane-2,3-diol **(+)-24**

The reaction was accomplished starting from **(+)-2** and alkyne **8** in *tert*-BuOH/H_2_O (2:1) according to the general procedure. The product was purified by column chromatography on silica gel with DCM/MeOH (19:1). Yield: 0.074 g, 58%; white crystals; m.p.: 140–142 °C; ${\left[\alpha \right]}_{D}^{20}$ = +5.0 (*c* 0.1, MeOH); ^1^H NMR (500 MHz, DMSO) δ (ppm): 1.02 (3H, s), 1.11 (3H, s), 1.25 (1H, d, *J* = 10.4 Hz), 1.51 (1H, t, *J* = 5.8 Hz), 1.55 (1H, dd, *J* = 5.1, 13.8 Hz), 1.83–1.77 (1H, m), 1.99–1.92 (1H, m), 2.41–2.33 (1H, m), 3.98–3.92 (1H, m), 4.24 (1H, d, *J* = 14.0 Hz), 4.32 (1H, d, *J* = 14.0 Hz), 4.49 (1H, s), 4.68 (2H, d, *J* = 5.9 Hz), 5.32 (1H, d, *J* = 6.2 Hz), 7.52 (2H, d, *J* = 8.6 Hz), 7.78 (1H, t, *J* = 5.8 Hz), 7.85 (3H, d, *J* = 7.4 Hz), 8.03 (1H, s); ^13^C NMR (125 MHz, DMSO) δ (ppm): 24.2, 27.7, 27.9, 36.7, 38.0, 38.5, 40.4, 48.2, 58.5, 64.8, 74.5, 105.2, 118.5 (2C), 121.0, 121.2, 124.5, 126.0 (2C, q, *J* = 3.6 Hz), 144.8, 144.9, 153.6, 157.8, 158.0; ^19^F NMR (470 MHz, DMSO) δ (ppm): −59.82; HRMS (ESI): *m*/*z* calcd. for C_24_H_28_ClF_3_N_7_O_2_ [M + H]^+^: 538.19396. Found: 538.19358.

The (1*R*,2*S*,3*S*,5*R*)-enantiomer **(−)-24** was synthesized analogously to **(+)-24**; ${\left[\alpha \right]}_{D}^{20}$ = −6.0 (*c* 0.1, MeOH); all the spectroscopic data and the m.p. were similar to those for the (1*R*,2*S*,3*S*,5*R*)-enantiomer. HRMS (ESI): *m*/*z* calcd. for C_24_H_28_ClF_3_N_7_O_2_ [M + H]^+^: 538.19396. Found: 538.19237.

#### 3.4.6. (1*S*,2*R*,3*R*,5*S*)-2-((4-(((5-Fluoro-2-((1-methyl-1H-pyrazol-4-yl)amino)pyrimidin-4-yl)amino)methyl)-1*H*-1,2,3-triazol-1-yl)methyl)-6,6-dimethylbicyclo[3.1.1]heptane-2,3-diol **(+)-25**

The reaction was accomplished starting from **(+)-2** and alkyne **9** in *tert*-BuOH/H_2_O (2:1) according to the general procedure. The product was purified by column chromatography on silica gel with DCM/MeOH (9:1). Yield: 0.096 g, 88%; pinkish white crystals; m.p.: 110–113 °C; ${\left[\alpha \right]}_{D}^{20}$ = +1.0 (*c* 0.2, MeOH); ^1^H NMR (500 MHz, CDCl_3_) δ (ppm): 1.10 (3H, s), 1.29 (3H, s), 1.41 (1H, d, *J* = 10.5 Hz), 1.67 (1H, dd, *J* = 4.6, 14.0 Hz), 1.98–1.91 (2H, m), 2.26–2.18 (1H, m), 2.55–2.47 (1H, m), 3.85 (3H, s), 4.17 (1H, d, *J* = 13.7 Hz), 4.37 (1H, dd, *J* = 5.0, 9.5 Hz), 4.50 (1H, d, *J* = 13.7 Hz), 4.72 (2H, ddd, *J* = 5.1, 14.9, 19.3 Hz), 5.74 (1H, s), 6.91 (1H, s), 7.43 (1H, s), 7.63 (1H, s), 7.66 (1H, s), 7.74 (1H, d, *J* = 3.1 Hz); ^13^C NMR (125 MHz, CDCl_3_) δ (ppm): 24.2, 27.5, 27.6, 36.3, 37.6, 38.6, 39.2, 40.5, 50.2, 59.8, 65.0, 74.6, 121.2, 123.3, 124.5, 131.1, 138.6 (d, *J* = 20.2 Hz), 141.1 (d, *J* = 244.2 Hz), 144.2, 152.6 (d, *J* = 12.3 Hz), 155.5; ^19^F NMR (470 MHz, CDCl_3_) δ (ppm): −169.82; HRMS (ESI): *m*/*z* calcd. for C_21_H_29_FN_9_O_2_ [M + H]^+^: 458.24228. Found: 458.24172.

The (1*R*,2*S*,3*S*,5*R*)-enantiomer **(−)-25** was synthesized analogously to **(+)-25**; ${\left[\alpha \right]}_{D}^{20}$ = −3.0 (*c* 0.2, MeOH); all the spectroscopic data and the m.p. were similar to those for the (1*R*,2*S*,3*S*,5*R*)-enantiomer. HRMS (ESI): *m*/*z* calcd. for C_21_H_29_FN_9_O_2_ [M + H]^+^: 458.24228. Found: 458.24147.

#### 3.4.7. (1*S*,2*R*,3*R*,5*S*)-2-((4-(((5-Chloro-2-((1-methyl-1*H*-pyrazol-4-yl)amino)pyrimidin-4-yl)amino)methyl)-1*H*-1,2,3-triazol-1-yl)methyl)-6,6-dimethylbicyclo[3.1.1]heptane-2,3-diol **(+)-26**

The reaction was accomplished starting from **(+)-2** and alkyne **10** in *tert*-BuOH/H_2_O (2:1) according to the general procedure. The product was purified by column chromatography on silica gel with DCM/MeOH (9:1). Yield: 0.107 g, 95%; pale pink crystals; m.p.: 116–119 °C; ${\left[\alpha \right]}_{D}^{20}$ = +2.0 (*c* 0.1, MeOH); ^1^H NMR (500 MHz CDCl_3_,) δ (ppm): 1.10 (3H, s), 1.28 (3H, s), 1.40 (1H, d, *J* = 10.5 Hz), 1.67 (2H, dd, *J* = 5.2, 14.2 Hz), 1.95 (2H, d, *J* = 5.7 Hz), 2.26–2.19 (1H, m), 2.55–2.47 (1H, m), 3.85 (3H, s), 4.17 (1H, d, *J* = 13.8 Hz), 4.38 (1H, dd, *J* = 5.0, 9.4 Hz), 4.50 (1H, d, *J* = 13.9 Hz), 4.73 (2H, tdd, *J* = 6.7, 13.4, 13.4 Hz), 5.91 (1H, s), 6.97 (1H, s), 7.44 (1H, s), 7.61 (1H, s), 7.65 (1H, s), 7.86 (1H, s); ^13^C NMR (125 MHz, CDCl_3_) δ (ppm): 24.3, 27.5, 27.6, 36.8, 37.6, 38.6, 39.2, 40.5, 50.1, 59.8, 64.9, 74.5, 121.4, 122.8, 124.5, 131.2, 144.3 (2C), 152.8, 157.7, 157.9; HRMS (ESI): *m*/*z* calcd. for C_21_H_29_ClN_9_O_2_ [M + H]^+^: 474.21273. Found: 474.21236.

The (1*R*,2*S*,3*S*,5*R*)-enantiomer **(−)-26** was synthesized analogously to **(+)-26**; ${\left[\alpha \right]}_{D}^{20}$ = −2.5 (*c* 0.1, MeOH); all the spectroscopic data and the m.p. were similar to those for the (1*R*,2*S*,3*S*,5*R*)-enantiomer. HRMS (ESI): *m*/*z* calcd. for C_21_H_29_ClN_9_O_2_ [M + H]^+^: 474.21273. Found: 474.21125.

#### 3.4.8. Methyl 4-((5-chloro-4-(((1-(((1*S*,2*R*,3*R*,5*S*)-2,3-dihydroxy-6,6-dimethylbicyclo[3.1.1]heptan-2-yl)methyl)-1*H*-1,2,3-triazol-4-yl)methyl)amino)pyrimidin-2-yl)amino)benzoate **(+)-27**

The reaction was accomplished starting from **(+)-2** and alkyne **12** in THF/H_2_O (2:1) according to the general procedure. The product was purified by column chromatography on silica gel with DCM/MeOH (19:1). Yield: 0.110 g, 88%; white crystals; m.p.: 131–134 °C; ${\left[\alpha \right]}_{D}^{20}$ = +5.5 (*c* 0.1 MeOH); ^1^H NMR (500 MHz, CDCl_3_) δ (ppm): 1.09 (3H, s), 1.28 (3H, s), 1.39 (1H, d, *J* = 10.7 Hz), 1.65 (1H, dd, *J* = 5.0, 14.0 Hz), 1.98–1.93 (2H, m), 2.23 (1H, ddd, *J* = 5.7, 5.7, 10.8 Hz), 2.55–2.49 (1H, m), 2.81 (1H, broad s), 3.88 (3H, s), 3.93 (1H, s), 4.14 (1H, d, *J* = 13.8 Hz), 4.43 (1H, dd, *J* = 5.2, 9.4 Hz), 4.49 (1H, d, *J* = 13.8 Hz), 4.77 (2H, dt, *J* = 5.7, 16.2 Hz), 5.92 (1H, t, *J* = 5.4 Hz), 7.27 (1H, s), 7.64 (2H, d, *J* = 8.4 Hz), 7.67 (1H, s), 7.94 (1H, s), 7.97 (2H, d, *J* = 8.4 Hz); ^13^C NMR (125 MHz, CDCl_3_) δ (ppm): 24.2, 27.5, 27.7, 36.8, 37.4, 38.6, 40.5, 50.3, 51.8, 59.9, 65.2, 74.5, 106.1, 117.8 (2C), 123.3, 124.6, 130.8 (2C), 144.0, 144.1, 153.2, 157.4, 157.7, 166.9; HRMS (ESI): *m*/*z* calcd. for C_25_H_31_ClN_7_O_4_ [M + H]^+^: 528.21206. Found: 528.21088.

The (1*R*,2*S*,3*S*,5*R*)-enantiomer **(−)-27** was synthesized analogously to those used for **(+)-27**; ${\left[\alpha \right]}_{D}^{20}$ = −4.5 (*c* 0.1, MeOH); all the spectroscopic data and the m.p. were similar to those for the (1*R*,2*S*,3*S*,5*R*)-enantiomer. HRMS (ESI): *m*/*z* calcd. for C_25_H_31_ClN_7_O_4_ [M + H]^+^: 528.21206. Found: 528.21124.

#### 3.4.9. (1*S*,2*R*,3*R*,5*S*)-2-((4-(((5-Fluoro-2-((4-morpholinophenyl)amino)pyrimidin-4-yl)amino)methyl)-1*H*-1,2,3-triazol-1-yl)methyl)-6,6-dimethylbicyclo[3.1.1]heptane-2,3-diol **(+)-28**

The reaction was accomplished starting from **(+)-2** and alkyne **11** in *tert*-BuOH/H_2_O (2:1) according to the general procedure. The product was purified by column chromatography on silica gel with DCM/MeOH (19:1). Yield: 0.093, 73%; yellowish-white crystals; m.p.: 125–128 °C; ${\left[\alpha \right]}_{D}^{20}$ = +6.0 (*c* 0.1 MeOH); ^1^H NMR (500 MHz, CDCl_3_) δ (ppm): 1.09 (3H, s), 1.29 (3H, s), 1.40 (1H, d, *J* = 10.6 Hz), 1.64 (1H, dd, *J* = 5.0, 13.9 Hz), 1.98–1.91 (2H, m), 2.27–2.20 (1H, m), 2.54–2.46 (1H, m), 3.10 (4H, dd, *J* = 4.7, 4.7 Hz), 3.85 (4H, dd, *J* = 4.6, 4.6 Hz), 4.11 (1H, d, *J* = 13.8 Hz), 4.41 (1H, dd, *J* = 5.2, 9.6 Hz), 4.47 (1H, d, *J* = 13.8 Hz), 4.71 (2H, ddt, *J* = 5.9, 13.2, 12.2 Hz), 5.54 (1H, t, *J* = 5.7 Hz), 6.82 (1H, s), 6.88 (2H, d, *J* = 8.7 Hz), 7.43 (2H, d, *J* = 8.7 Hz), 7.59 (1H, s), 7.76 (1H, d, *J* = 2.4 Hz); ^13^C NMR (125 MHz, CDCl_3_) δ (ppm): 24.3, 27.6, 27.7, 36.1, 37.5, 38.6, 40.5, 50.2 (2C), 50.4, 59.9, 65.1, 67.0 (2C), 74.5, 116.6 (2C), 121.1 (2C), 124.8, 133.1, 139.3, 144.3, 147.0, 152.2, 152.3, 156.2; ^19^F NMR (470 MHz, CDCl_3_) δ (ppm): −169.35; HRMS (ESI): *m*/*z* calcd. for C_27_H_36_FN_8_O_3_ [M + H]^+^: 539.28944. Found: 539.28715.

The (1*R*,2*S*,3*S*,5*R*)-enantiomer **(−)-28** was synthesized analogously to **(+)-28**; ${\left[\alpha \right]}_{D}^{20}$ = −8.0 (*c* 0.1, MeOH); all the spectroscopic data and the m.p. were like those for the (1*R*,2*S*,3*S*,5*R*)-enantiomer. HRMS (ESI): *m*/*z* calcd. for C_27_H_36_FN_8_O_3_ [M + H]^+^: 539.28944. Found: 539.28820.

#### 3.4.10. (1*S*,2*R*,3*R*,5*S*)-2-((4-((6-Chloro-9*H*-purin-9-yl)methyl)-1*H*-1,2,3-triazol-1-yl)methyl)-6,6-dimethylbicyclo[3.1.1]heptane-2,3-diol **(+)-29**

The reaction was accomplished starting from **(+)-2** and alkyne **15** in THF/H_2_O (2:1) according to the general procedure. The product was purified by column chromatography on silica gel with CHCl_3_/MeOH (19:1) increased to (9:1). Yield: 0.090 g, 94%; white crystals; m.p.: 120–123 °C; ${\left[\alpha \right]}_{D}^{20}$ = +3 (*c* 0.1, MeOH); ^1^H NMR (500 MHz, CDCl_3_) δ (ppm): 1.09 (3H, s), 1.30 (3H, s), 1.39 (1H, d, *J* = 10.6 Hz), 1.68–1.62 (1H, m), 1.99–1.92 (2H, m), 2.29–2.22 (1H, m), 2.55–2.48 (1H, m), 2.63 (1H, broad s), 3.98 (1H, s), 4.18 (1H, d, *J* = 13.8 Hz), 4.39–4.34 (1H, m), 4.53 (1H, d, *J* = 13.8 Hz), 5.56 (2H, q, *J* = 12.4 Hz), 7.91 (1H, s), 8.30 (1H, s), 8.75 (1H, s); ^13^C NMR (125 MHz, CDCl_3_) δ (ppm): 24.2, 27.5, 27.6, 37.6, 38.6, 39.2, 40.4, 50.1, 60.0, 64.9, 74.5, 125.6, 131.5, 140.6, 145.2, 151.2, 151.5, 152.1; HRMS (ESI): *m*/*z* calcd. for C_18_H_23_ClN_7_O_2_ [M + H]^+^: 404.16018. Found: 404.15907.

The (1R,2S,3S,5R)-enantiomer (−)-29 was synthesized analogously to (+)-29; ${\left[\alpha \right]}_{D}^{20}$ = −6.0 (c 0.1, MeOH); all the spectroscopic data and the m.p. were similar to those for the (1R,2S,3S,5R)-enantiomer. HRMS (ESI): *m*/*z* calcd. for C_18_H_23_ClN_7_O_2_ [M + H]^+^: 404.16018. Found: 404.15909.

#### 3.4.11. (1*S*,2*R*,3*R*,5*S*)-2-((4-((6-Chloro-8-phenyl-9*H*-purin-9-yl)methyl)-1*H*-1,2,3-triazol-1-yl)methyl)-6,6-dimethylbicyclo[3.1.1]heptane-2,3-diol **(+)-30**

The reaction was accomplished starting from **(+)-2** and alkyne **16** in THF/H_2_O (2:1) according to the general procedure. The product was purified by column chromatography on silica gel with toluene/EtOH (9:1).Yield: 0.112 g, 98%; white crystals; m.p.: 159–162 °C; ${\left[\alpha \right]}_{D}^{20}$ = +5.5 (*c* 0.1, MeOH); ^1^H NMR (500 MHz, CDCl_3_) δ (ppm): 1.08 (3H, s), 1.29 (3H, s), 1.39 (1H, d, *J* = 10.6 Hz), 1.63 (1H, dd, *J* = 4.9, 14.2 Hz), 1.99–1.91 (2H, m), 2.27–2.21 (1H, m), 2.44 (1H, d, *J* = 5.7 Hz), 2.53–2.46 (1H, m), 3.90 (1H, s), 4.15 (1H, d, *J* = 13.9 Hz), 4.36 (1H, ddt, *J* = 5.0, 5.0, 4.9 Hz), 4.50 (1H, d, *J* = 13.9 Hz), 5.60 (2H, q, *J* = 14.2 Hz), 7.61–7.57 (3H, m), 7.89 (1H, s), 8.06–8.02 (2H, m), 8.73 (1H, s); ^13^C NMR (125 MHz, CDCl_3_) δ (ppm): 24.2, 27.5, 27.6, 37.4, 38.6, 39.9, 40.4, 50.2, 59.9, 65.0, 74.5, 126.2, 128.2, 129.1 (2C), 130.1 (2C), 131.3, 131.6, 141.7, 150.2, 151.5, 153.5, 156.2; HRMS (ESI): *m*/*z* calcd. for C_24_H_27_ClN_7_O_2_ [M + H]^+^: 480.19147. Found: 480.19051.

The (1*R*,2*S*,3*S*,5*R*)-enantiomer **(−)-30** was synthesized analogously to **(+)-30**; ${\left[\alpha \right]}_{D}^{20}$ = −5.0 (*c* 0.1, MeOH); all the spectroscopic data and the m.p. were similar to those for the (1*R*,2*S*,3*S*,5*R*)-enantiomer. HRMS (ESI): *m*/*z* calcd. for C_24_H_27_ClN_7_O_2_ [M + H]^+^: 480.19147. Found: 480.19019.

#### 3.4.12. (1*S*,2*R*,3*R*,5*S*)-6,6-Dimethyl-2-((4-((6-phenyl-9*H*-purin-9-yl)methyl)-1*H*-1,2,3-triazol-1-yl)methyl)bicyclo[3.1.1]heptane-2,3-diol **(+)-31**

The reaction was accomplished starting from **(+)-2** and alkyne **17** in THF/H_2_O (2:1) according to the general procedure. The product was purified by column chromatography on silica gel with DCM/MeOH (19:1). Yield: 0.080 g, 76%; white crystals; m.p.: 120–122 °C; ${\left[\alpha \right]}_{D}^{20}$ = +6.5 (*c* 0.1, MeOH); ^1^H NMR (500 MHz, CDCl_3_) δ (ppm): 1.09 (3H, s), 1.29 (3H, s), 1.39 (1H, d, *J* = 10.6 Hz), 1.65 (1H, d, *J* = 5.3 Hz), 1.95 (2H, d, *J* = 6.0 Hz), 2.28–2.21 (1H, m), 2.54–2.47 (1H, m), 2.72 (1H, d, *J* = 5.7 Hz), 4.01 (1H, s), 4.17 (1H, d, *J* = 13.9 Hz), 4.36 (1H, ddt, *J* = 5.0, 5.0, 4.9 Hz), 4.52 (1H, d, *J* = 13.9 Hz), 5.58 (2H, q, *J* = 13.9 Hz), 7.58–7.50 (3H, m), 7.91 (1H, s), 8.29 (1H, s), 8.74 (2H, dd, *J* = 1.5, 8.2 Hz), 9.00 (1H, s); ^13^C NMR (125 MHz, CDCl_3_) δ (ppm): 24.2, 27.5, 27.6, 37.5, 38.6, 38.9, 40.4, 50.2, 59.9, 64.9, 74.5, 125.6, 128.7 (2C), 129.8 (2C), 131.1, 135.5, 141.3, 144.3, 152.1 (2C), 152.4, 155.1; HRMS (ESI): *m*/*z* calcd. for C_24_H_28_N_7_O_2_ [M + H]^+^: 446.23044. Found: 446.22916.

The (1*R*,2*S*,3*S*,5*R*)-enantiomer **(−)-31** was synthesized analogously to **(+)-31**; ${\left[\alpha \right]}_{D}^{20}$ = −6.5 (*c* 0.1, MeOH); all the spectroscopic data and the m.p. were similar to those for the (1*R*,2*S*,3*S*,5*R*)-enantiomer. HRMS (ESI): *m*/*z* calcd. for C_24_H_28_N_7_O_2_ [M + H]^+^: 446.23044. Found: 446.22935.

#### 3.4.13. (1*S*,2*R*,3*R*,5*S*)-2-((4-((6-(Benzylamino)-9*H*-purin-9-yl)methyl)-1*H*-1,2,3-triazol-1-yl)methyl)-6,6-dimethylbicyclo[3.1.1]heptane-2,3-diol (+)-32

The reaction was accomplished starting from **(+)-2** and alkyne **18** in THF/H_2_O (2:1) according to the general procedure. The product was purified by column chromatography on silica gel with DCM/MeOH (19:1). Yield: 0.106 g, 94%; white crystals; m.p.: 122–125 °C; ${\left[\alpha \right]}_{D}^{20}$ = +9.0 (*c* 0.1, MeOH); ^1^H NMR (500 MHz, CDCl_3_) δ (ppm): 1.01 (3H, s), 1.10–1.04 (1H, m), 1.19 (1H, d, *J* = 10.5 Hz), 1.26 (3H, s), 1.75–1.70 (1H, m), 1.99 (1H, t, *J* = 5.7 Hz), 2.18–2.09 (2H, m), 4.05 (1H, d, *J* = 13.6 Hz), 4.15–4.08 (1H, m), 4.41 (1H, s), 4.57 (1H, d, *J* = 13.6 Hz), 4.85 (1H, d, *J* = 12.6 Hz), 5.42 (2H, s), 6.27 (1H, s), 7.37–7.26 (5H, m), 7.95 (1H, s), 8.24 (1H, s), 8.37 (1H, s); ^13^C NMR (125 MHz, CDCl_3_) δ (ppm): 24.2, 27.5, 27.6, 37.4, 38.4, 39.2, 40.4, 44.9, 50.3, 60.2, 63.0, 74.3, 118.9, 126.8, 127.6, 128.2, 128.6 (3C), 137.9, 139.6, 141.1, 148.4, 153.0, 153.9; HRMS (ESI): *m*/*z* calcd. for C_25_H_31_ClN_8_O_2_ [M + H]^+^: 475.25699. Found: 475.25567.

The (1*R*,2*S*,3*S*,5*R*)-enantiomer **(−)-32** was synthesized analogously to **(+)-32**; ${\left[\alpha \right]}_{D}^{20}$ = −9.0 (*c* 0.1, MeOH); all the spectroscopic data and the m.p. were similar to those for the (1*R*,2*S*,3*S*,5*R*)-enantiomer. HRMS (ESI): *m*/*z* calcd. for C_25_H_31_ClN_8_O_2_ [M + H]^+^: 475.25699. Found: 475.25576.

#### 3.4.14. (1*S*,2*R*,3*R*,5*S*)-6,6-Dimethyl-2-((4-((6-((4-(trifluoromethyl)phenyl)amino)-9*H*-purin-9-yl)methyl)-1*H*-1,2,3-triazol-1-yl)methyl)bicyclo[3.1.1]heptane-2,3-diol **(+)-33**

The reaction was accomplished starting from **(+)-2** and alkyne **19** in THF/H_2_O (2:1) according to the general procedure. The product was purified by column chromatography on silica gel with DCM/MeOH (19:1). Yield: 0.125 g, 99%; white crystals; m.p.: 139–141 °C; ${\left[\alpha \right]}_{D}^{20}$ = +6.5 (*c* 0.1, MeOH); ^1^H NMR (500 MHz, CDCl_3_) δ (ppm): 1.04 (3H, s), 1.30 (3H, s), 1.36–1.31 (1H, m), 1.48 (1H, d, *J* = 10.4 Hz), 1.83–1.78 (1H, m), 2.23–2.13 (2H, m), 2.33–2.26 (1H, m), 4.13 (1H, d, *J* = 13.5 Hz), 4.23–4.17 (1H, m), 4.58 (1H, s), 4.69 (1H, d, *J* = 13.5 Hz), 5.53 (2H, s), 6.22 (1H, d, *J* = 6.5 Hz), 7.32 (2H, d, *J* = 8.6 Hz), 7.52 (2H, d, *J* = 8.6 Hz), 8.06 (1H, s), 8.23 (1H, s), 8.48 (1H, s), 8.49 (1H, s); ^13^C NMR (125 MHz, CDCl_3_) δ (ppm): 24.3, 27.6, 28.0, 37.9, 38.4, 39.6, 40.5, 50.3, 60.5, 62.9, 74.5, 118.6 (2C), 119.2, 124.0 (q, *J* = 32.6 Hz), 125.8 (2C, q, *J* = 3.8 Hz), 127.5, 127.7, 140.6, 140.7, 141.8, 149.0, 150.8, 152.1; ^19^F NMR (470 MHz, CDCl_3_) δ (ppm): −61.98; HRMS (ESI): *m*/*z* calcd. for C_25_H_28_F_3_N_8_O_2_ [M + H]^+^: 529.22873. Found: 529.22747.

The (1*R*,2*S*,3*S*,5*R*)-enantiomer **(−)-33** was synthesized analogously to **(+)-33**; ${\left[\alpha \right]}_{D}^{20}$ = −4.0 (*c* 0.1, MeOH); all the spectroscopic data and the m.p. were similar to those for the (1*R*,2*S*,3*S*,5*R*)-enantiomer. HRMS (ESI): *m*/*z* calcd. for C_25_H_28_F_3_N_8_O_2_ [M + H]^+^: 529.22873. Found: 529.22742.

### 3.5. Determination of Antiproliferative Effect

The growth-inhibitory effects of the isosteviol-based 1,3-aminoalcohols were determined by a standard MTT (3-(4,5-dimethylthiazol-2-yl)-2,5-diphenyltetrazolium bromide) assay on a panel containing five cell lines, including Hela and SiHa (cervical cancer), MDA-MB-231 and MCF-7 (breast cancers), and A2780 ovarian cancer cells. NIH/3T3 fibroblasts were additionally used to obtain preliminary information on the cancer selectivity. All cell lines were purchased from the European Collection of Cell Cultures (Salisbury, UK), except for the SiHa (American Tissue Culture Collection (ATCC, Manassas, VA, USA). The cells were maintained in a minimal essential medium supplemented with 10% fetal bovine serum, 1% non-essential amino acids, and 1% penicillin–streptomycin at 37 °C in a humidified atmosphere containing 5% CO_2_. All media and supplements were obtained from Lonza Group Ltd. (Basel, Switzerland). Cancer cells were seeded into 96-well plates (5000 cells/well); after overnight incubation, the test compound was added in two different concentrations (10 µM and 30 µM) and incubated for another 72 h under cell-culturing conditions. In the next step, 20 μL of 5 mg/mL MTT solution was added to each well and incubated for a further 4 h. The medium was removed, and the precipitated formazan crystals were dissolved in DMSO after 60 min of shaking at 37 °C. As a final step, the absorbance was measured at 545 nm by using a microplate reader. Untreated cells were included as controls. In the case of the most effective compounds, the assays were repeated with a set of dilutions (1.0–30 μM) to determine the IC_50_ values. Two independent experiments were performed with five wells for each condition. Calculations were performed using the GraphPad Prism 10.0 software (GraphPad Software Inc., San Diego, CA, USA).

### 3.6. Antimicrobial Analyses

The synthesized compounds were dissolved in MeOH and diluted with H_2_O to reach concentration levels of up to 100 µg/mL and 10 µg/mL with a final MeOH content of 10%. The completeness of the dissolution was observed visually in each case. Then, the resulting solutions were investigated in a microdilution assay against bacterial and yeast strains, including *Bacillus subtilis* SZMC 0209 (Gram-positive), *Staphylococcus aureus* SZMC 14611 (Gram-positive), *Escherichia coli* SZMC 6271 (Gram-negative), and *Pseudomonas aeruginosa* SZMC 0177 (Gram-negative) as well as *Candida albicans* SZMC 1533 and *Candida krusei* SZMC 1352, respectively, according to the M07-A10 CLSI guideline and our previous work [4]. The microbe suspensions were prepared from fresh cultures cultivated overnight in different fermentation broths for both bacteria (10 g/L peptone, 5 g/L NaCl, 5 g/L yeast extract) and yeast (20 g/L peptone, 10 g/L yeast extract, 20 g/L glucose) at 37 °C, and their concentrations were set to 4 × 10^5^ cells/mL with sterile media. After that, 100 μL of suspension containing the bacterial or yeast cells and 50 μL of sterile broth as well as 50 μL of the test solutions were placed into the wells of 96-well plates and incubated for 24 h at 37 °C and 32 °C for bacteria and yeasts, respectively. The blank sample was a mixture of 150 μL broth and 50 μL of a test solution used for background correction, while 100 μL of microbial suspension supplemented with 50 μL of sterile broth and 50 μL of 10% MeOH was applied as a negative control. For the positive control, ampicillin or nystatin was applied for bacteria or fungi, respectively, at two final concentration levels (100 µg/mL and 10 µg/mL). The inhibitory effects were calculated as the percentage of the negative control after blank correction using the following formula: inhibitory effect (%) = 100 − ((ODtest − ODblank)/ODnegative) × 100)). Here, ODtest represents the OD values of the test solution, ODnegative represents the OD values of the negative control, and ODblank represents the OD values of blank samples.

## 4. Conclusions

Key intermediate azidodiols, synthesized according to literature from commercially available (+)- and (−)-α-pinene in a four-step sequence, were reacted with pyrimidine- and purine-based propargyl-type alkynes in a click reaction carried out in the presence of Cu(OAc)_2_ and Na-ascorbate. The resulting methylene-1,2,3-triazolo-linked nucleoside analogues were screened for antiproliferative activity by MTT assay on HeLa, MD231, SiHa, MCF-7 and A2780 human cancer cell lines compared with fibroblast cells (NIH/3T3). From weak to moderate antiproliferative activities were observed and the SAR determination showed that, meanwhile, the stereochemistry had a minimal but consequent effect on activity. The diaminopyrimidine ring with 5-fluoro, 5-chloro, and 2-(4-trifluoromethyl)phenyl substituents proved essential for moderate activity with selectivity on HeLa and MCF-7 cell lines.

All prepared compounds were tested on Gram-positive and Gram-negative pathogenic bacteria and two yeasts, and the SAR was explained in detail. The starting azidodiols had excellent and selective antibacterial activities against *S. aureus* and *P. aeruginosa* with moderate antifungal effect on *C. krusei*. Pyrimidines and purine derivatives also expressed a moderate antifungal effect against *C. krusei*. One of the purine-based derivatives possessed a remarkable and selective antibacterial effect against *P. aeruginosa*.

By comparing the two series of enantiomers, it can be clearly stated that compounds derived from (+)-α-pinene have a more pronounced growth-inhibitory effect on HeLa and A2780 cell lines, whereas compounds derived from (−)-α-pinene inhibit the growth of the MCF7 cell line more effectively. In case of antibacterial and antifungal activities, there are no significant differences between the enantiomeric series.

The purine-coupled monoterpene derivatives seem to be an interesting group to extend the study of the substituent effect and the modified purine structure to obtain more effective antifungal and antibacterial compounds. The antibacterial effect experienced in the case of azidodiol compounds makes it interesting and necessary to investigate additional terpene-based azidodiols in order to discover potential antibiotics.

## Figures and Tables

**Table 1:** Selected data and IC_50_ values of the most active derivatives **(+)-23**, **(−)-23**, **(+)-24** and **(−)-24**.

Comp.	Conc. (μM)	Growth Inhibition (%) ± SEM ^1,2^ Calculated IC_50_ (μM)					
		HeLa	SiHa	MCF-7	MDA-MB-231	A2780	NIH/3T3
**(+)-23**	10	66.04 ± 1.27	–	21.67 ± 2.45	–	30.45 ± 1.86	–
	30	89.81 ± 1.34	42.81 ± 2.03	84.53 ± 1.25	20.30 ± 1.89	80.44 ± 1.57	48.65 ± 2.058
	IC_50_	7.54	>30	18.59	>30	14.79	>30
**(−)-23**	10	23.39 ± 2.39	–	28.48 ± 2.42	10.18 ± 1.94	25.34 ± 1.77	40.61 ± 2.06
	30	96.09 ± 1.82	94.73 ± 2.01	93.28 ± 1.02	96.44 ± 2.98	93.19 ± 1.53	76.30 ± 3.295
	IC_50_	13.68	19.85	11.77	15.78	13.80	11.71
**(+)-24**	10	26.01 ± 2.80	10.89 ± 1.84	36.81 ± 3.59	–	45.43 ± 1.76	23.49 ± 1.60
	30	100.94 ± 0.47	95.32 ± 1.86	91.45 ± 0.90	90.81 ± 1.47	95.25 ± 1.30	90.34 ± 1.79
	IC_50_	11.06	16.21	16.15	20.93	11.86	14.64
**(−)-24**	10	26.61 ± 1.36	–	62.86 ± 0.97	34.81 ± 0.70	32.98 ± 2.64	42.45 ± 0.77
	30	97.56 ± 0.76	97.59 ± 1.28	98.24 ± 0.47	95.68 ± 2.10	101.38 ± 0.72	96.77 ± 1.22
	IC_50_	12.76	18.90	8.79	11.69	11.06	10.51

^1^: Mean ± SEM values from two determinations with five parallel wells in each. ^2^: Inhibition values less than 10% are regarded as negligible and are not given numerically.

**Table 2:** Antimicrobial activities of selected compounds according to Figure 2.

Comp. ^1,2^	Conc. (µg/mL)	Growth Inhibition (%) ^2^ ± SEM ^3^					
		*B. subtilis*	*S. aureus*	*P. aeruginosa*	*E. coli*	*C. albicans*	*C. krusei*
**Amp**	100	84.7 ± 7.0	100.0 ± 3.5	100.0 ± 3.2	100.4 ± 8.6	–	–
	10	40.1 ± 6.3	68.48 ± 2.2	99.7 ± 3.8	60.2 ± 8.3	–	–
**Nyst**	100	–	–	–	–	99.0 ± 2.2	99.0 ± 2.1
	10	–	–	–	–	95.9 ± 16.0	84.3 ± 20.0
**(+)-2**	100	16.6 ±4.1	101.3 ± 7.0	0	24.9 ± 2.6	36.8 ± 12.4	58.0 ± 13.4
	10	14.6 ± 6.4	101.2 ± 8.5	17.7 ± 6.5	24.4 ± 2.4	0	54.4 ± 13.9
**(−)-2**	100	3.3 ± 6.8	101.1 ± 8.3	0	0	32.8 ±1.5	73.9 ± 10.7
	10	0.6 ± 6.0	101.6 ± 5.1	95.1± 29.6	13.7 ± 3.1	7.5 ± 21.1	57.8 ± 10.5
**(+)-20**	100	5.2 ± 8.5	0	0	17.1 ± 7.1	4.2 ± 8.7	73.1 ± 3.4
	10	6.2 ± 4.7	0	0	18.5 ± 2.6	2.0 ± 16.3	61.4 ± 1.8
**(−)-20**	100	10.5 ± 7.1	12.1 ± 4.9	0	12.7 ± 7.2	0	64.1 ± 20.1
	10	12.8 ± 9.2	10.8 ± 5.8	0	20.2 ± 2.8	7.1 ± 9.7	67.5 ± 18.0
**(−)-21**	100	22.2 ± 2.1	7.6 ± 2.5	0	26.3 ± 2.2	0	64.5 ± 10.1
	10	14.3 ± 7.4	13.6 ± 5.2	0	26.6 ± 8.0	2.7 ± 4.5	66.2 ± 11.8
**(+)-25**	100	20.7 ± 3.5	0	0	22.0 ± 3.7	0	24.3 ± 5.8
	10	16.5 ± 7.9	6.3 ± 1.3	0	26.3 ± 2.5	0	68.3 ± 8.4
**(+)-26**	100	16.2 ± 8.6	0	0	17.8 ± 4.6	39.7 ± 3.8	0
	10	15.6 ± 9.7	4.5 ± 3.5	0	21.8 ± 5.3	20.1 ± 9.1	66.8 ± 4.3
**(−)-26**	100	16.2 ± 7.7	2.6 ± 6.6	0	20.5 ± 1.7	7.2 ± 3.4	60.7 ± 5
	10	14.9 ± 8.1	10.4 ± 3.2	0	27.8 ± 9.5	0	67.5 ± 15.2
**(+)-28**	100	23.6 ± 5.3	0	0	18.5 ± 5.7	0	66.2 ± 10.4
	10	16.5 ± 4.6	9.1 ± 3.2	0	26.6 ± 6.5	7.9 ± 13.7	65.3 ± 6.2
**(−)-30**	100	23.1 ± 5.7	0	0	15.52 ± 4.80	0	66.4 ± 2.4
	10	14.7 ± 5.5	0	94.0 ± 28.7	11.21 ± 6.53	4.2 ± 0.9	62.5 ± 12.6
**(+)-32**	100	15.9 ± 5.2	0	–	–	0	101.7 ± 16.1
	10	18.2 ± 4.2	5.3 ± 4.6	0	18.5 ± 5.4	1.4 ± 3.6	66.5 ± 9.9

^1^: Amp: ampicillin, Nys: nystatin; numeric values for all prepared monoterpenic compounds are given in Table S2 in the Supplementary Materials. ^2^: Solutions, which were not tested due to solubility problems, are marked with a en dash (–). ^3^: Mean ± SEM values from two determinations with 3 parallel wells in each.

## Data Availability

The original contributions presented in this study are included in the article/Supplementary Materials. Further inquiries can be directed to the corresponding author.

## Supplementary Materials

The following supporting information can be downloaded at: https://www.mdpi.com/article/10.3390/ijms262311705/s1.

## Abbreviations

The following abbreviations are used in this manuscript:

THF	Tetrahydrofurane
DCM	Dichlormethane
mCPBA	*meta*-Chloroperbenzoic acid
MW	Microwave
TEOF	Triethyl orthoformate
TEA	Triethylamine
CuAAC	Copper-catalyzed azide–alkyne cycloaddition
