# Supplementary material for: 1,2,3-Triazolo-Bridged Click Coupling of Pinane-Based Azidodiol Enantiomers with Pyrimidine- and Purine-Based Building Blocks: Synthesis, Antiproliferative, and Antimicrobial Evaluation
Source: Int J Mol Sci. 2025 Dec 3;26(23):11705. doi: 10.3390/ijms262311705 (PMC12692267; doi:10.3390/ijms262311705)

Supporting information

for

1,2,3-Triazolo-bridged Click coupling of pinane-based azidodiol enantiomers with pyrimidine- and purine-based building blocks: synthesis, antiproliferative and antimicrobial evaluation

Dima Depp [1], Kitti Tari [2], András Szekeres [2], Adriána Kovács [3], István Zupkó [3] and Zsolt Szakonyi [1*]

[1] Institute of Pharmaceutical Chemistry, University of Szeged, Eötvös utca 6, H-6720 Szeged, Hungary; dima.depp@hotmail.com

[2] Department of Biotechnology and Microbiology, University of Szeged, Közép fasor 52. H-6726 Szeged Hungary; AS: andras.j.szekeres@gmail.com; KT: kittitari2000@gmail.com

[3] Institute of Pharmacodynamics and Biopharmacy, University of Szeged, H-6720 Szeged, Eötvös utca 6, Hungary; IZ: zupko.istvan@szte.hu; AK: kovacs.adriana.judit@szte.hu

* Correspondence: szakonyi.zsolt@szte.hu; Tel.: +36-62-546809

Contents

Synthesis of compounds derived from (–)-α-pinene	S3
Investigation of antiproliferative activity of diaminopyrimidines and purines	S4–S5
Investigation of antibacterial activity of diaminopyrimidines and purines	S6–S7
^{1}H, ^{13}C, ^{19}F NMR, COSY, NOESY, HSQC, HMBC spectra of new compounds	S8–S194
HRMS spectra of new compounds	S195–S226

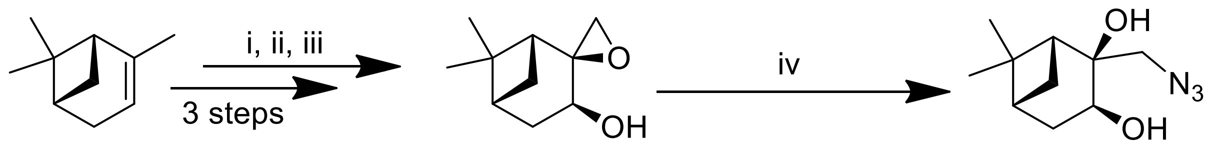

(-)-α-pinene (-)-1 (-)-2

Scheme S1. Synthesis of enantiomeric pinane based azidodiols. (i) *m*CPBA, $NaHCO_3$, DCM, 10 °C, 1.5 h, 82%; (ii) Al(O-iPr)$_3$, dry toluene, reflux, 3 h, 70%; (iii) *m*CPBA, 5% NaH_2PO_4, 0 °C, 2 h, 60%; (iv) NaN_3, 20% NH_4Cl, EtOH/H_2O, reflux, 36 h, 85%.

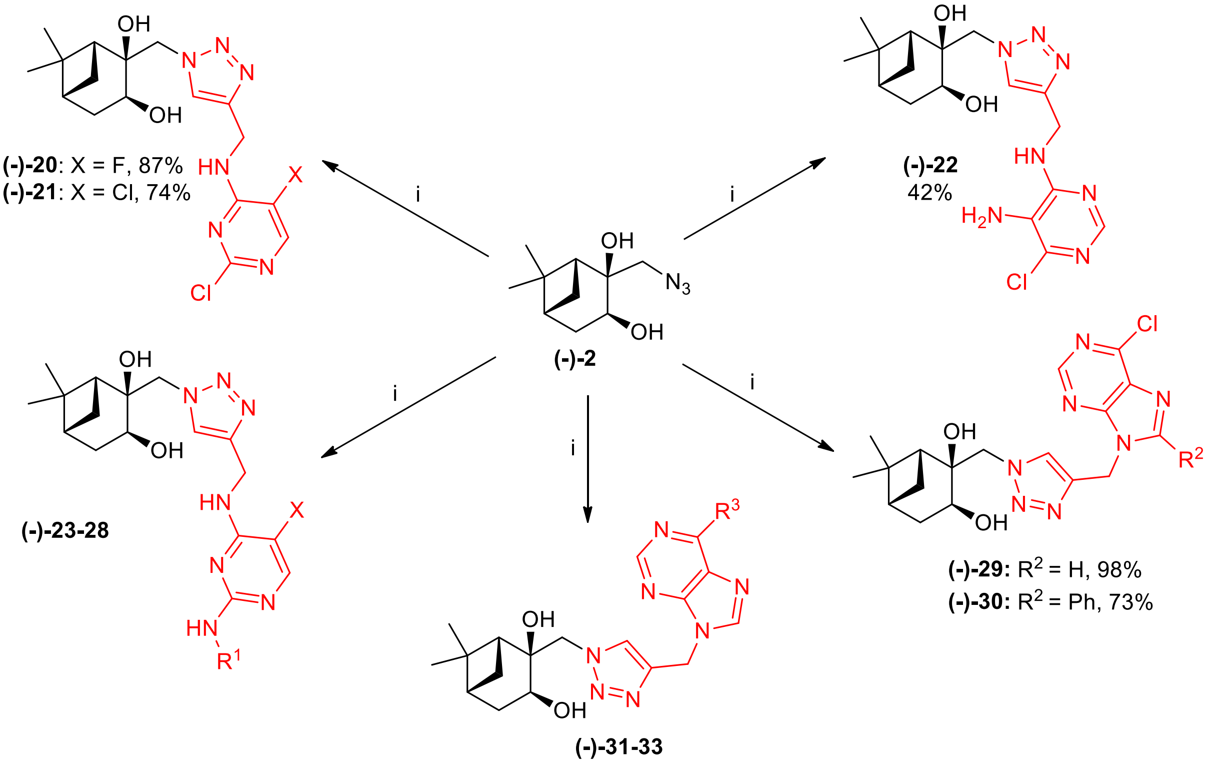

(-)-23: X = F; R^1 = 4-(trifluoromethyl)phenyl, 85%
(-)-24: X = Cl; R^1 = 4-(trifluoromethyl)phenyl, 58%
(-)-25: X = F; R^1 = 1-methyl-*1H*-pyrazol-4-yl, 67%
(-)-26: X = Cl; R^1 = 1-methyl-*1H*-pyrazol-4-yl, 83%
(-)-27: X = Cl; R^1 = 4-(methoxycarbonyl)phenyl, 75%
(-)-28: X = F; R^1 = 1-(4-morpholinophenyl), 75%
(-)-31: R^3 = Ph, 77%
(-)-32: R^3 = benzylamino, 95%
(-)-33: R^3 =(4-trifluoromethyl)phenylamino, 88%

Scheme S2. Synthesis of enantiomeric pinane-based hybrid compounds **20-33**. (i) Cu(OAc)$_2$•H_2O (0.05 eq.), Na ascorbate (0.1 eq.), alkyne (1.1 eq.), 45 °C, 48 h; *tert*-BuOH/H_2O (2:1) for **(–)-20**, **(–)-21**, **(–)-23** – **(–)-26**, and **(–)-28** or THF/H_2O (2:1) for **(–)-22**, **(–)-27**, and **(–)-29** – **(–)-33**.

Table S1 *In vitro* antiproliferative activity of the prepared compounds

Comp.	Conc. (μM)	Growth inhibition (%) ± SEM[1]<br>Calculated IC_{50} (μM) and selectivity index (SI)[2]					
		HeLa	SiHa	MCF-7	MDA-MB-231	A2780	NIH/3T3
**(+)-20**	10	–[3]	–	–	12.44 ± 2.10	–	–
	30	–	22.36 ± 0.99	14.52 ± 2.63	12.43 ± 1.80	–	–
**(–)-20**	10	–	–	–	–	–	–
	30	–	20.12 ± 0.51	–	–	–	–
**(+)-21**	10	–	–	–	14.30 ± 1.84	–	–
	30	–	20.83 ± 2.39	12.90 ± 1.26	19.68 ± 2.77	–	–
**(–)-21**	10	–	–	–	–	–	–
	30	–	–	–	–	–	–
**(+)-22**	10	24.94 ± 1.98	–	–	–	–	–
	30	41.18 ± 1.93	–	10.65 ± 2.14	–	–	–
**(–)-22**	10	17.16 ± 2.57	–		–	–	–
	30	37.77 ± 2.65	–	25.19 ± 1.54	–	–	–
**(+)-23**	10	66.04 ± 1.27	–	21.67 ± 2.45	–	30.45 ± 1.86	–
	30	89.81 ± 1.34	42.81 ± 2.03	84.53 ± 1.25	20.30 ± 1.89	80.44 ± 1.57	48.65 ± 2.06
	IC_{50}	7.54	>30	18.59	>30	14.79	>30
**(–)-23**	10	23.39 ± 2.39	–	28.48 ± 2.42	10.18 ± 1.94	25.34 ± 1.77	40.61 ± 2.06
	30	96.09 ± 1.82	94.73 ± 2.01	93.28 ± 1.02	96.44 ± 2.98	93.19 ± 1.53	76.30 ± 3.30
	IC_{50}<br>SI	13.68<br>0.86	19.85<br>0.59	11.77<br>0.99	15.78<br>0.74	13.80<br>0.85	11.71
**(+)-24**	10	26.01 ± 2.80	10.89 ± 1.84	36.81 ± 3.59	–	45.43 ± 1.76	23.49 ± 1.60
	30	100.94 ± 0.47	95.32 ± 1.86	91.45 ± 0.90	90.81 ± 1.47	95.25 ± 1.30	90.34 ± 1.79
	IC_{50}<br>SI	11.06<br>1.32	16.21<br>0.90	16.15<br>0.91	20.93<br>0.70	11.86<br>1.23	14.64
**(–)-24**	10	26.61 ± 1.36	–	62.86 ± 0.97	34.81 ± 0.70	32.98 ± 2.64	42.45 ± 0.77
	30	97.56 ± 0.76	97.59 ± 1.28	98.24 ± 0.47	95.68 ± 2.10	101.38 ± 0.72	96.77 ± 1.22
	IC_{50}<br>SI	12.76<br>0.82	18.90<br>0.56	8.79<br>1.20	11.69<br>0.90	11.06<br>0.95	10.51
**(+)-25**	10	–	–	–	–	–	–
	30	14.97 ± 2.51	38.22 ± 2.63	14.97 ± 0.99	–	–	–
**(–)-25**	10	–	–	–	–	–	–
	30	10.64 ± 1.56	11.96 ± 1.56	17.47 ± 2.05	–	–	–
**(+)-26**	10	–	–	–	–	–	–
	30	34.52 ± 2.41	37.51 ± 1.86	29.97 ± 1.53	25.12 ± 2.86	50.51 ± 1.94	37.79 ± 2.56
**(–)-26**	10	–	–	–	–	–	–
	30	36.36 ± 1.02	32.52 ± 1.19	36.51 ± 1.76	22.57 ± 1.94	42.32 ± 1.93	25.65 ± 2.06
**(+)-27**	10	–	–	–	–	–	–
	30	–	23.92 ± 1.36	60.68 ± 1.10	26.35 ± 1.50	37.11 ± 2.21	–
**(–)-27**	10	–	–	–	–	–	–
	30	27.39 ± 1.02	–	54.10 ± 1.46	28.05 ± 2.081	46.82 ± 2.68	
**(+)-28**	10	–	14.56 ± 1.31	–	–	–	–
	30	16.15 ± 1.72	24.87 ± 0.94	15.27 ± 1.02	–	–	–
**(–)-28**	10	–	11.73 ± 2.06	–	–	–	–
	30	–	26.63 ± 1.92	23.75 ± 1.01	–	–	19.49 ± 0.42
**(+)-29**	10	24.92 ± 2.87	–	–	–	–	–
	30	35.37 ± 2.64	–	41.95 ± 1.14	23.49 ± 1.91	22.37 ± 2.81	–
**(–)-29**	10	28.98 ± 1.21	–	–	–	–	–
	30	29.72 ± 2.21	–	26.52 ± 2.27	13.95 ± 2.66	23.62 ± 2.84	–
**(+)-30**	10	11.00 ± 1.67	–	12.66 ± 2.64	–	–	–
	30	27.63 ± 2.11	19.81 ± 2.89	67.32 ± 1.04	52.30 ± 2.30	51.45 ± 2.35	–
**(–)-30**	10	–	–	13.98 ± 1.83	–	–	–
	30	14.58 ± 1.62	–	45.86 ± 1.97	11.07 ± 1.25	–	–

**(+)-31**	10	–	11.73 ± 2.42	–	–	–	–
	30	–	13.74 ± 2.56	26.60 ± 2.27	–	–	21.61 ± 3.01
**(–)-31**	10	–	–	–	–	11.41 ± 1.81	–
	30	–	16.02 ± 1.46	15.91 ± 2.97	–	14.70 ± 1.83	–
**(+)-32**	10	–	–	–	–	17.98 ± 2.71	–
	30	14.67 ± 1.48	14.09 ± 2.15	14.87 ± 2.24	–	18.49 ± 2.50	–
**(–)-32**	10	–	–	–	–	4.838 ± 1.47	–
	30		20.60 ± 1.43	13.56 ± 2.92	–	14.81 ± 1.13	–
**(+)-33**	10	–	–	45.73 ± 1.72	–	50.04 ± 1.42	–
	30	94.31 ± 0.86	84.42 ± 3.25	85.52 ± 1.26	71.96 ± 0.06	93.95 ± 3.20	38.71 ± 2.45
**(–)-33**	10	–	–	38.98 ± 2.45	–	15.61 ± 1.88	–
	30	93.00 ± 1.85	91.46 ± 1.31	83.61 ± 1.62	46.85 ± 3.58	93.61 ± 3.37	37.12 ± 1.94
**Cisplatin**[4]	10	42.61 ± 2.33	60.98 ± 0.92	53.03 ± 2.29	20.84 ± 0.81	83.57 ± 1.21	73.88 ± 1.63
	30	99.93 ± 0.26	88.95 ± 0.53	86.90 ± 1.22	74.47 ± 1.20	95.02 ± 0.28	97.10 ± 0.15
	IC_{50}	12.41	4.29	8.34	19.10	5.27	5.50
	SI	0.44	1.28	0.66	0.29	1.04	

[1]: Mean ± SEM values from two determinations with 5 parallel wells in each

[2]: Selectivity index (SI) is defined as the ratio of $IC_{50\ NIH/3T3}$ and $IC_{50\ cancer\ cell}$. A higher SI value indicates a more selective action. The SI value has not been calculated when $IC_{50\ NIH/3T3}$ >30 μM.

[3]: Inhibition values less than 10% are regarded as negligible and are not given numerically

[4]: The values of reference agent cisplatin were published earlier [*RSC Adv.* **2024,** 14, (49), 36698-36712]

Table S2. Antimicrobial activities of the synthesised compounds.

Comp.[1]	Conc. (μg/ml)	Growth inhibition (%)[2] ± SEM[3]					
		B. subtilis SZMC0209	*S. aureus* SZMC14611	*P. aeruginosa* SZMC0177	*E. coli* SZMC6271	*C. albicans* SZMC1533	*C. krusei* SZMC1352
**Amp**	100	84.7 ± 7	100 ± 3.5	100 ± 3.2	100.4 ± 8.6	-	-
	10	40.1 ± 6.3	68.5 ± 2.2	99.7 ± 3.8	60.2 ± 8.3	-	-
**Nys**	100	–	–	–	–	99 ± 2.2	99.5 ± 2.1
	10	–	–	–	–	95.9 ± 16	84.3 ± 20
**(+)-2**	100	16.6 ± 4.1	101.3 ± 6.95	0	24.9 ± 2.6	36.8 ± 12.4	58 ± 13.4
	10	14.6 ± 6.4	101.2 ± 8.5	17.7±6.5	24.4 ± 2.4	0	54.4 ± 13.9
**(–)-2**	100	3.3 ± 6.8	101.1 ± 8.3	0	0	32.8 ± 1.5	73.9 ± 10.7
	10	0.6 ± 5.96	101.6 ± 5.1	95.1±29.6	13.7 ± 3.1	7.5 ± 21.1	57.8 ± 10.5
**(+)-20**	100	5.2 ± 8.5	0	0	17.1 ± 7.1	4.2 ± 8.7	73.1 ± 3.4
	10	6.2 ± 4.7	0	0	18.5 ± 2.6	2 ± 16.3	61.4 ± 1.8
**(–)-20**	100	10.5 ± 7.1	12.1 ± 4.9	0	12.7 ± 7.2	0	64.1 ± 20.1
	10	12.8 ± 9.2	10.8 ± 5.8	0	20.2 ± 2.8	7.1 ± 9.7	67.5 ± 18
**(+)-21**	100	11 ± 7.1	0	0	23± 2.7	0	0
	10	12.6 ± 9.9	5.1 ± 2.5	0	22.6 ± 1.1	0	60.5 ± 7.5
**(–)-21**	100	22.2 ± 2.1	7.6 ± 2.5	0	26.3 ± 2.2	0	64.5 ± 10.1
	10	14.3 ± 7.4	13.6 ± 5.2	0	26.6 ± 7.98	2.7 ± 4.5	66.2 ± 11.8
**(+)-22**	100	47.9 ± 16.2	0	0	22.1 ± 6.8	0	20.6 ± 25.5
	10	17.3 ± 3.95	9 ± 3	0	30.3 ± 4.8	0	60.6 ± 6.1
**(–)-22**	100	43 ± 3.8	0	0	22.1 ± 6.9	0	4.5 ± 12.3
	10	18.6 ± 4	8.1 ± 2.4	5.3±6.1	28.2 ± 3.3	0	60.1 ± 5.3
**(+)-23**	100	–	–	–	–	–	–
	10	11.9 ± 3.5	0	0	14.7 ± 10.2	33.3 ± 23.1	3.8 ± 9.9
**(–)-23**	100	–	–	–	–	–	–
	10	4.1 ± 7.1	28.7 ± 5.8	0	34.6 ± 9.6	0	65.6 ± 10.6
**(+)-24**	100	–	–	–	–	–	–
	10	6.4 ± 4.8	29.6 ± 5.5	0	0	0	49.6 ± 9.2
**(–)-24**	100	–	–	–	–	–	–
	10	8.9 ± 6.1	28.7 ± 4.8	0	13.7 ± 7.6	22.9 ± 16.6	1.2 ± 7.1
**(+)-25**	100	20.7 ± 3.5	0	0	22 ± 3.7	0	24.3 ± 5.8
	10	16.5 ± 7.9	6.3 ± 1.3	0	26.34 ± 2.5	0	68.3 ± 8.4
**(–)-25**	100	11.6 ± 8.9	0	0	26.8 ± 6.3	0	64.9 ± 12.7
	10	20.4 ± 6.7	4.4 ± 6.97	0	26.7 ± 4.2	3.5 ± 14	53 ± 2.2
**(+)-26**	100	16.2 ± 8.6	0	0	17.8 ± 4.6	39.7 ± 3.8	0
	10	15.6 ± 9.7	4.5 ± 3.5	0	21.8 ± 5.3	20.1 ± 9.1	66.8 ± 4.3
**(–)-26**	100	16.2 ± 7.7	2.6 ± 6.7	0	20.5 ± 1.7	7.2 ± 3.4	60.7 ± 5
	10	14.9 ± 8.1	10.4 ± 3.2	0	27.8 ± 9.5	0	67.5 ± 15.2
**(+)-27**	100	-	-	-	-	-	-

	10	10.9 ± 7.2	0	0	21.2 ± 6.2	0	0
**(–)-27**	100	–	–	–	–	–	–
	10	2.6 ± 6.4	8.1 ± 6.5	0	19.1 ± 2	0	56.7 ± 27.3
**(+)-28**	100	23.6 ± 5.3	0	0	18.5 ± 5.7	0	66.2 ± 10.4
	10	16.5 ± 4.6	9.1 ± 3.2	0	26.6 ± 6.5	7.9 ± 13.7	65.3 ± 6.2
**(–)-28**	100	45 ± 6.1	0	0	38.3 ± 0.9	18 ± 22.4	50.9 ± 15.8
	10	6.1 ± 1.7	1.5 ± 2.4	0	28.2 ± 8.1	12.6 ± 4.3	53.9 ± 13.8
**(+)-29**	100	–	–	–	–	–	–
	10	6.7 ± 6.5	12.3 ± 3.9	0	26 ± 5.9	0	59.1 ± 11
**(–)-29**	100	18.5 ± 6.6	0	0	6.7 ± 1.5	0	0
	10	13.4 ± 5.6	0	0	4.7 ± 8.2	0	57.5 ± 6.5
**(+)-30**	100	23.7 ± 3.7	0	0	26.6 ± 6.4	0	20.1 ± 14.2
	10	19.4 ± 4.4	0.3 ± 8	0	31.8 ± 3.5	0	61.5 ± 10.9
**(–)-30**	100	23.1 ± 5.7	0	0	15.5 ± 4.8	0	66.4 ± 2.4
	10	14.7 ± 5.5	0	94.0±28.7	11.2 ± 6.5	4.2 ± 0.9	62.5 ± 12.6
**(+)-31**	100	4.4 ± 1.9	0	0	0	0	15.5 ± 15
	10	6.6 ± 2.98	0	0	0	0.5 ± 23.8	0
**(–)-31**	100	14.4 ± 1.5	15.2 ± 7.5	0	15.4 ± 4.3	7.9 ± 24.6	65.1 ± 6.4
	10	12.9 ± 6.4	13 ± 4.3	0	23.4 ± 4.7	5 ± 12.4	57.8 ± 23.6
**(+)-32**	100	15.9 ± 5.2	0	–	–	0	101.7 ± 16.1
	10	18.2 ± 4.2	5.3 ± 4.6	0	18.5 ± 5.4	1.4 ± 3.6	66.5 ± 9.9
**(–)-32**	100	24.1 ± 8.9	9.8 ± 7.6	–	–	11.5 ± 18.9	0
	10	22.5 ± 9.9	16.4 ± 5.4	0	15.8 ± 4.2	18.8 ± 9.4	13 ± 29.2
**(+)-33**	100	–	–	–	–	–	–
	10	8.8 ± 2.9	28.3 ± 3.8	0	39.8 ± 0.8	0	52.3 ± 6.3
**(–)-33**	100	–	–	–	–	–	–
	10	0.8 ± 5.4	17.9 ± 4.8	0	36.5 ± 3.6	0	53.6 ± 10.2

[1]: Amp: ampicillin, Nys: nystatin

[2]: Solutions, which were not tested due to the solubility problems are marked with hyphen (–).

[3]: Mean ± SEM values from two determinations with 3 parallel wells in each.

6-Chloro-8-phenyl-9-(prop-2-yn-1-yl)-9*H*-purine (**16**)

Figure S 1. ^{1}H-NMR of compound **16**

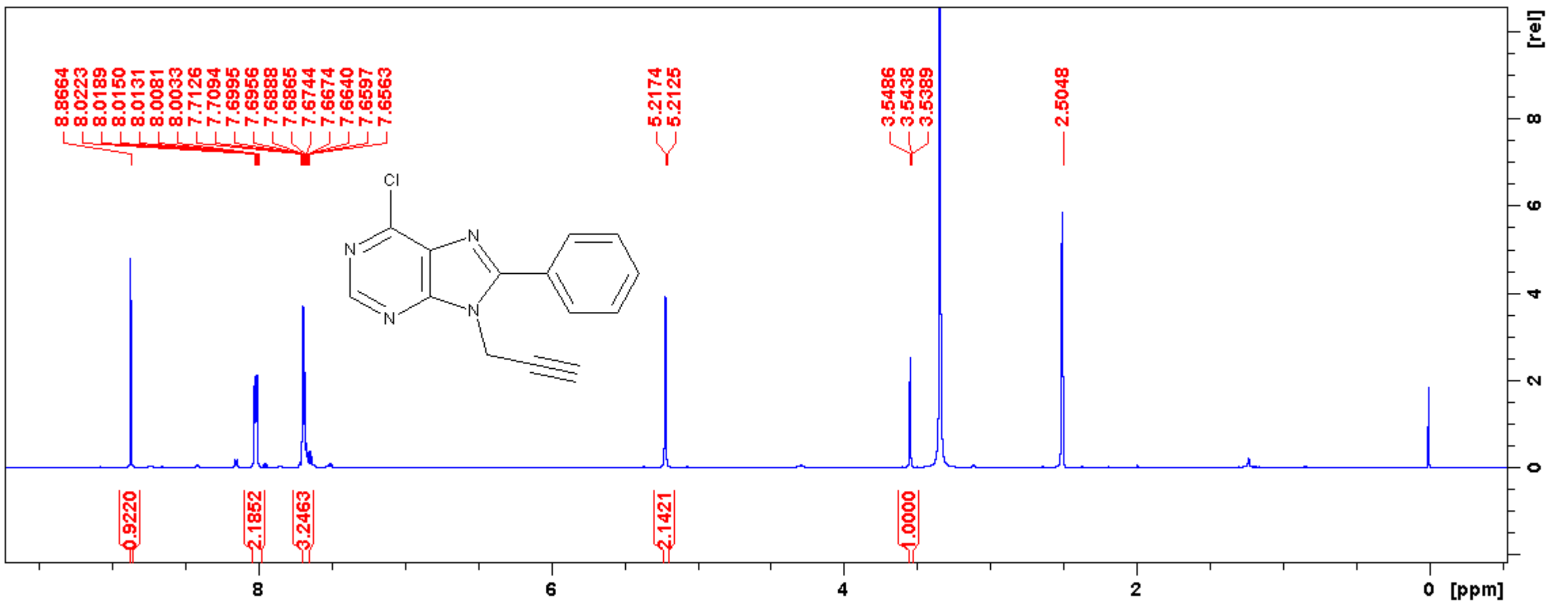

Figure S 2. ^{13}C-NMR of compound **16**

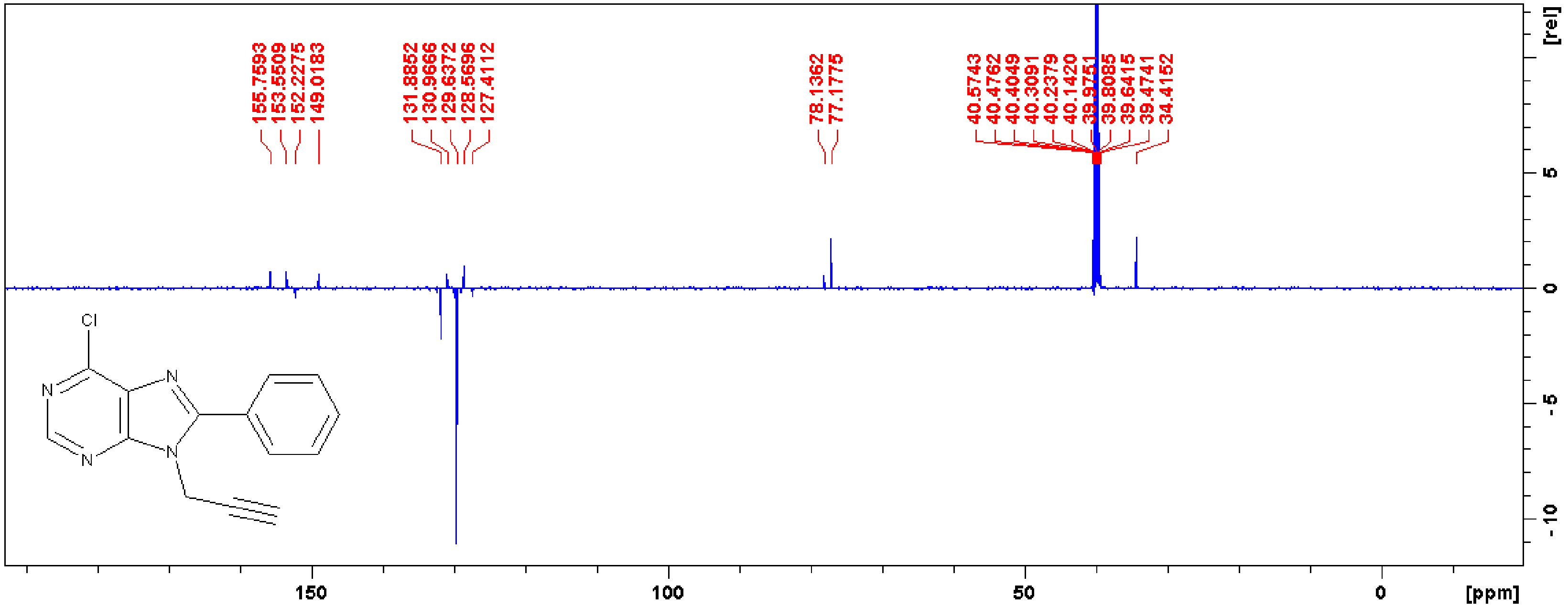

N-Benzyl-9-(prop-2-yn-1-yl)-9*H*-purin-6-amine (**18**)

Figure S 3. ^{1}H-NMR of compound **18**

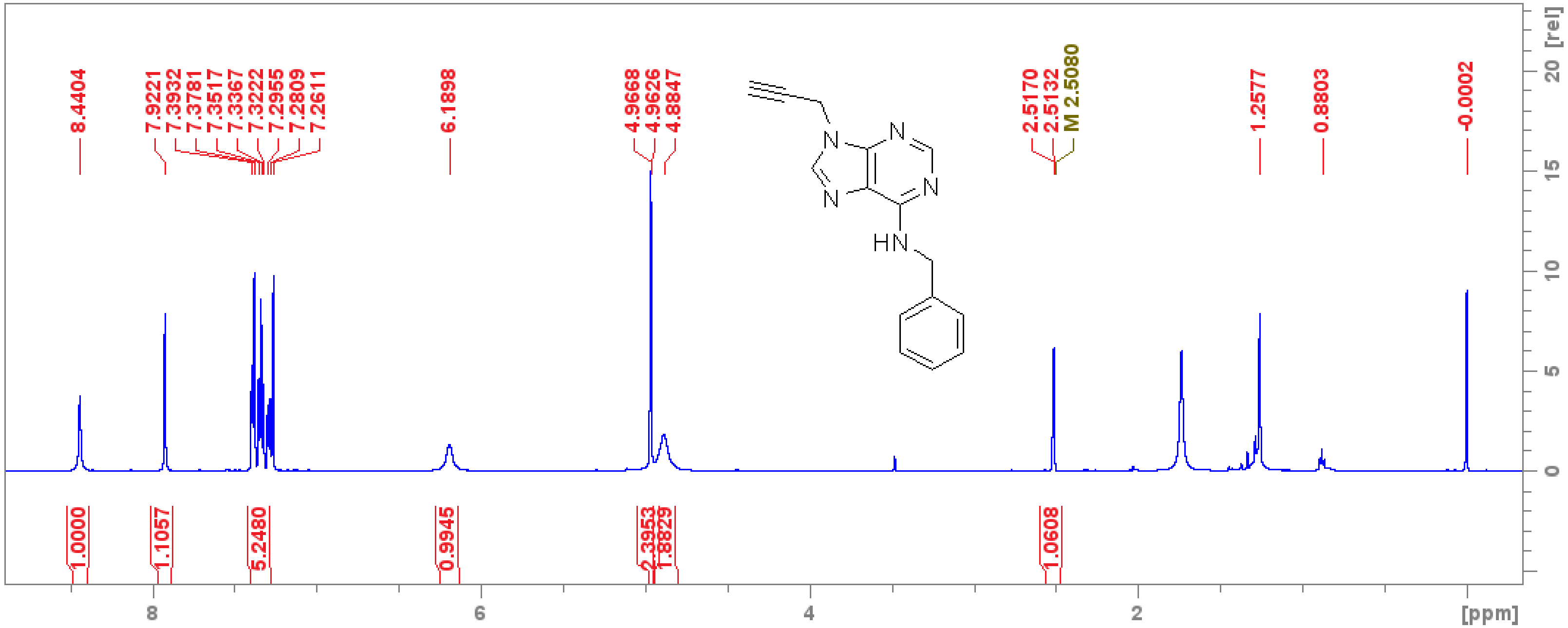

Figure S 4. ^{13}C-NMR of compound **18**

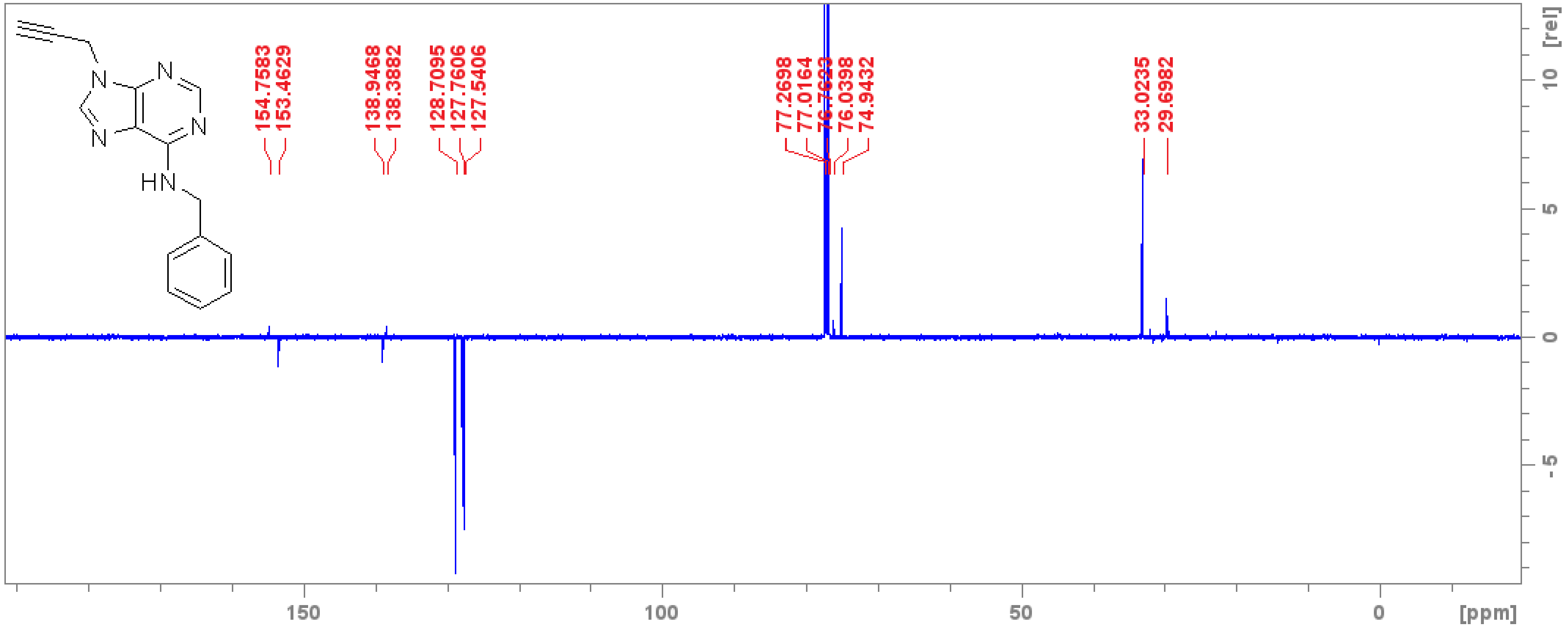

9-(prop-2-yn-1-yl)-*N*-(4-(trifluoromethyl)phenyl)-9*H*-purin-6-amine (**19**)

Figure S 5. ^{1}H-NMR of compound **19**

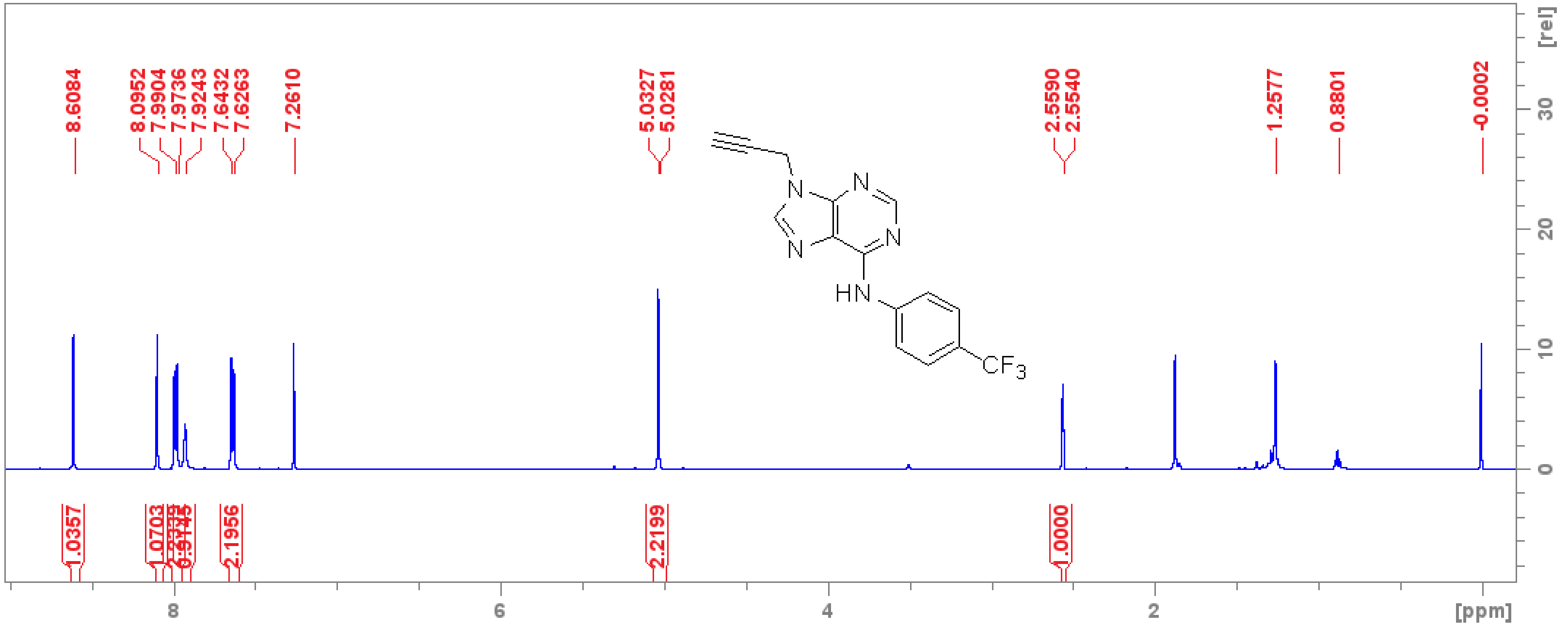

Figure S 6. ^{13}C-NMR of compound **19**

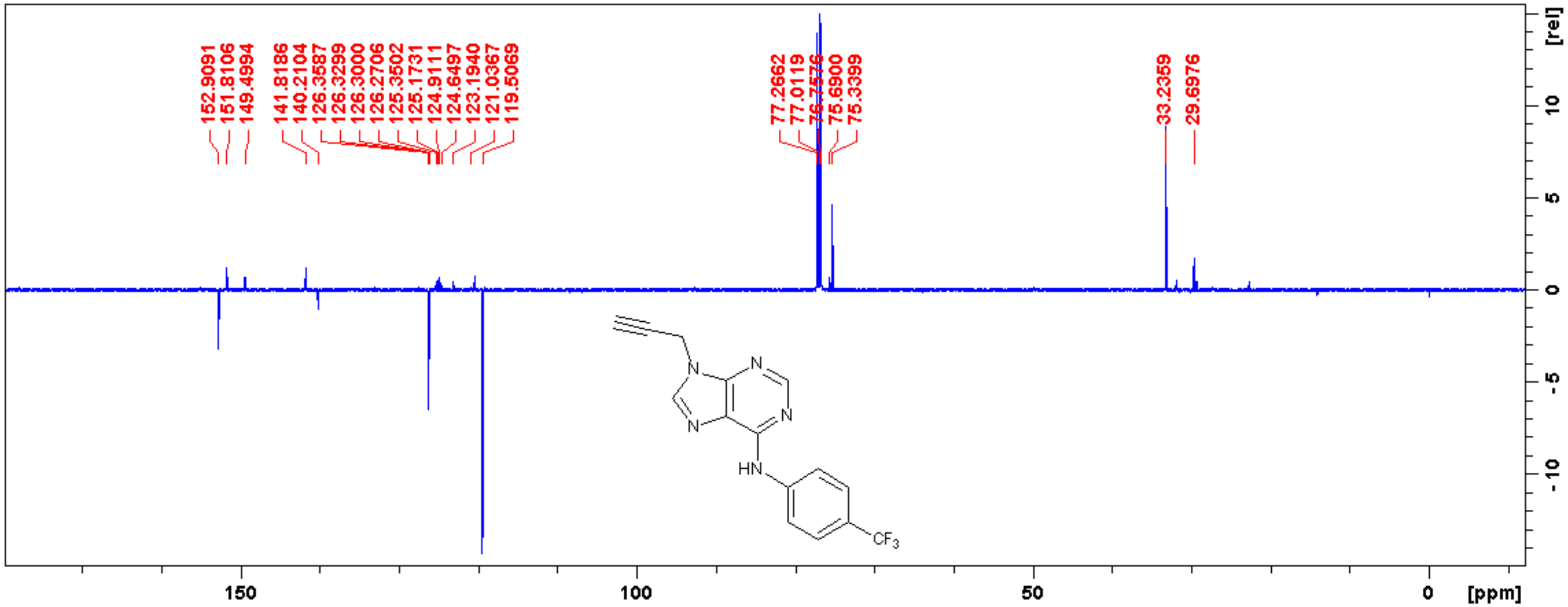

Figure S 7. ^{19}F-NMR of compound **19**

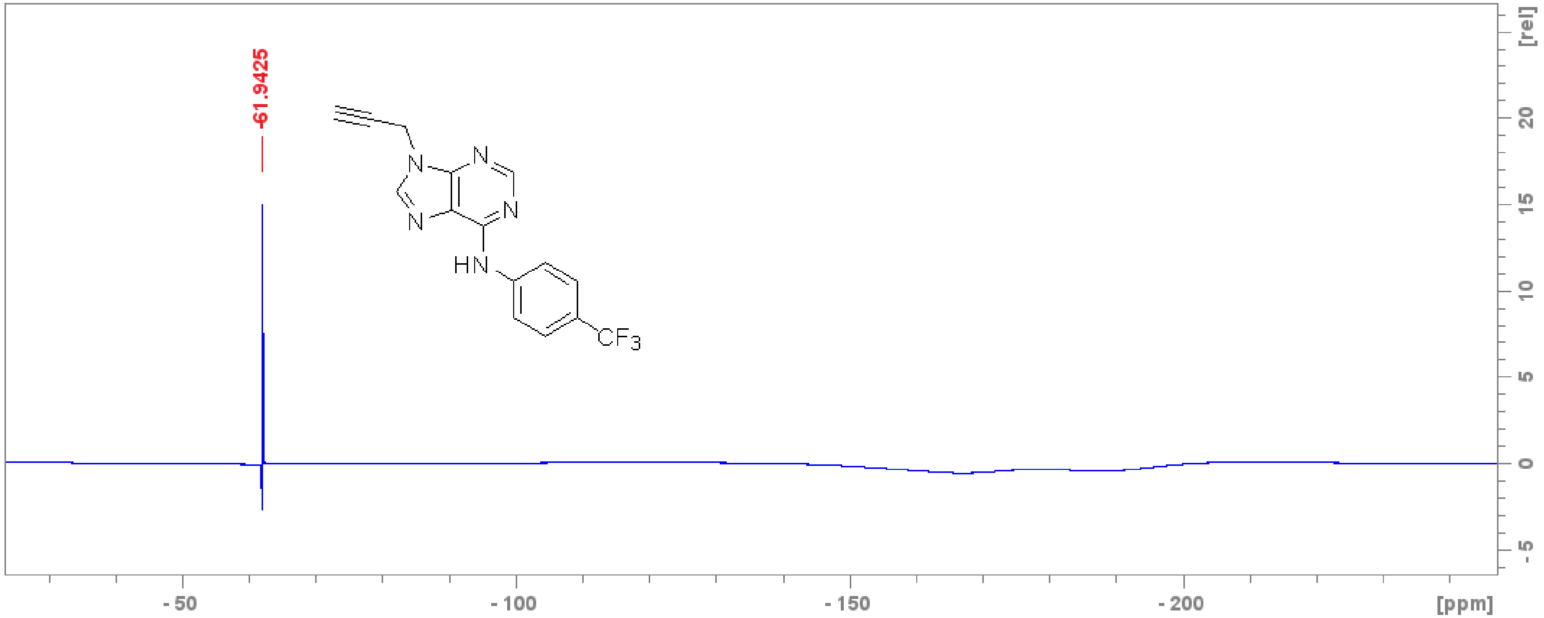

(1*S*,2*R*,3*R*,5*S*)-2-((4-(((2-Chloro-5-fluoropyrimidin-4-yl)amino)methyl)-1*H*-1,2,3-triazol-1-yl)methyl)-6,6-dimethylbicyclo[3.1.1]heptane-2,3-diol **(+)-20**

Figure S 8. ^{1}H-NMR of compound **(+)-20**

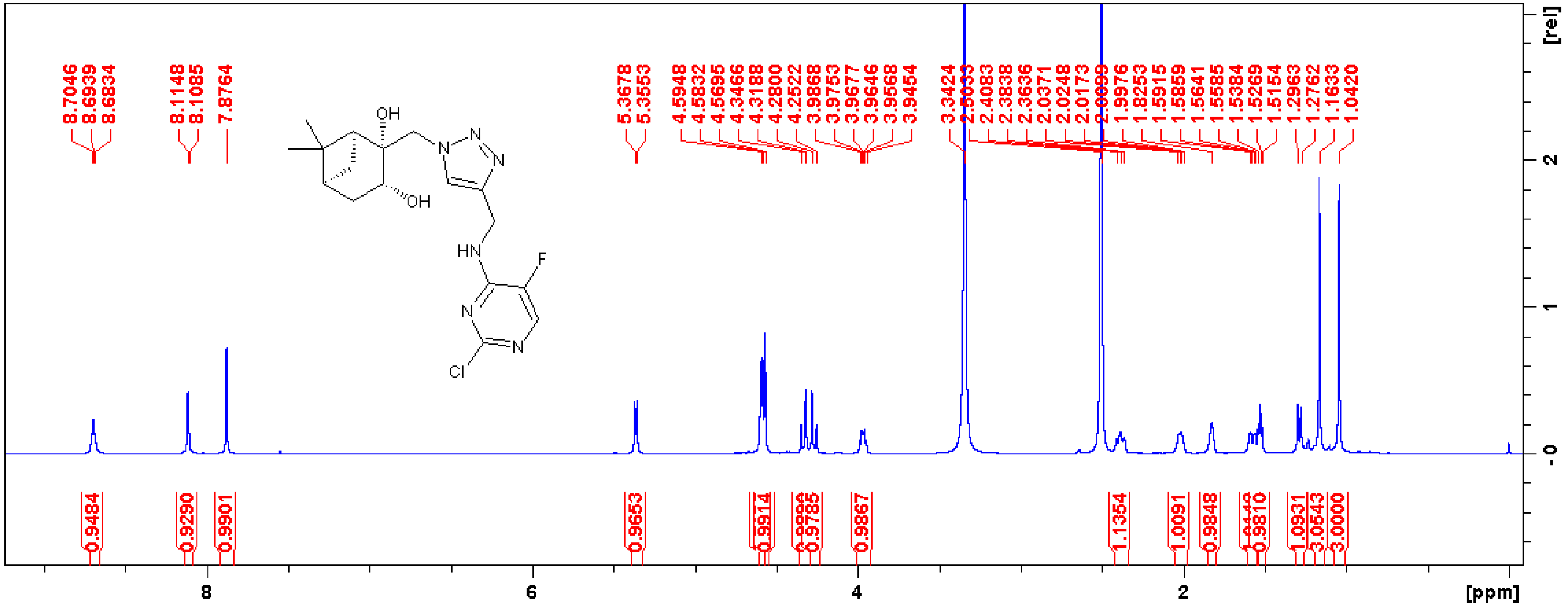

Figure S 9. ^{13}C-NMR of compound **(+)-20**

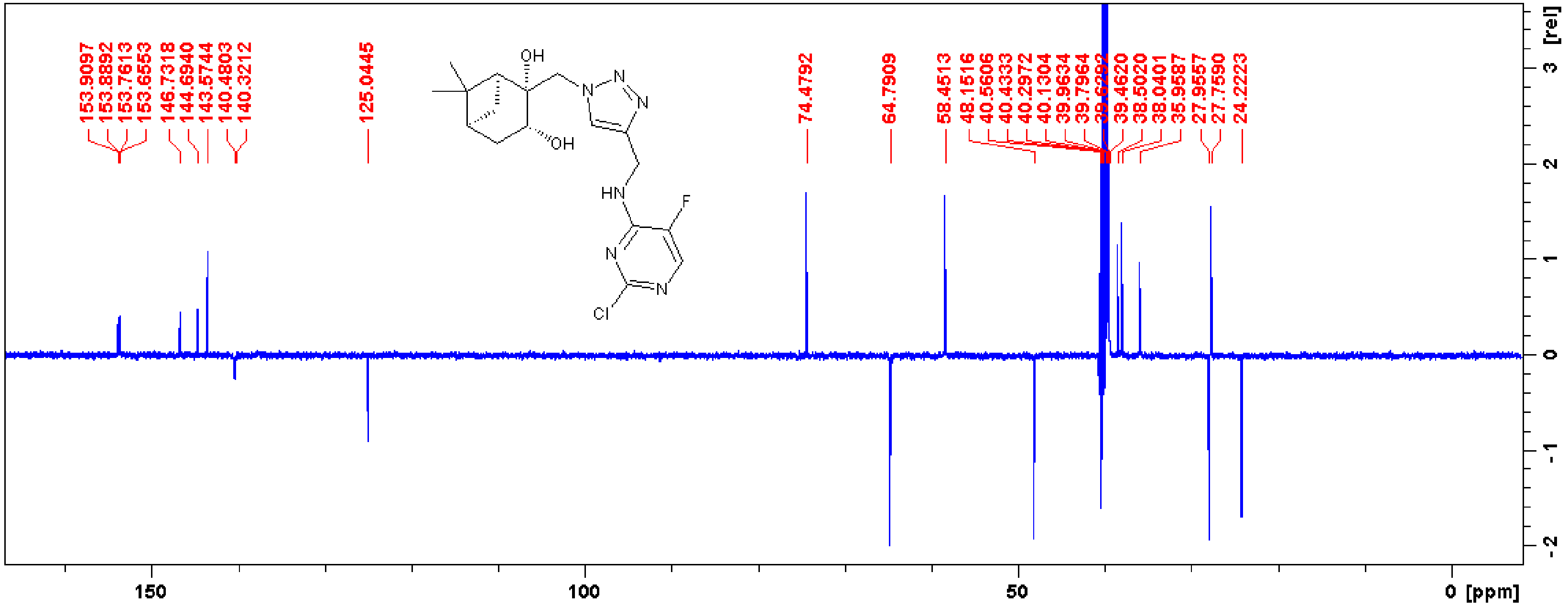

Figure S 10. COSY-NMR of compound **(+)-20**

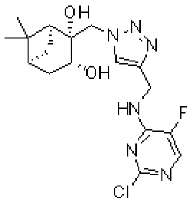

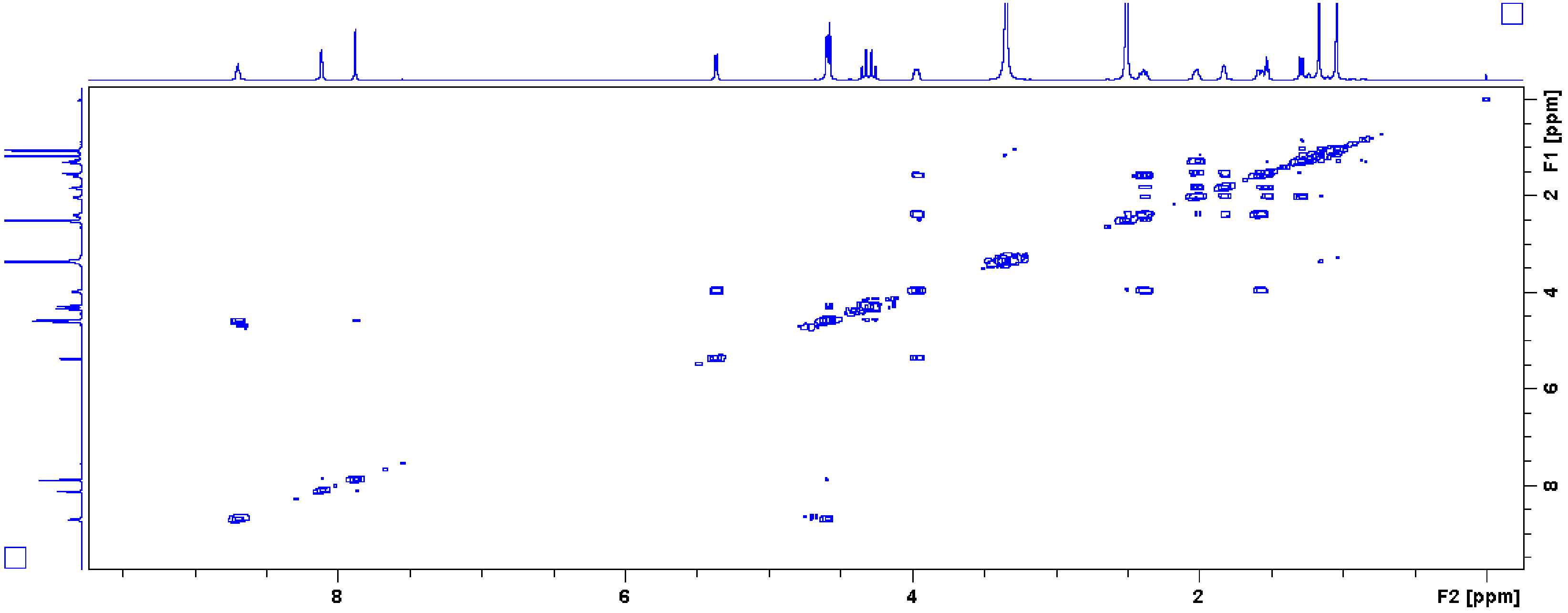

Figure S 11. NOESY-NMR of compound **(+)-20**

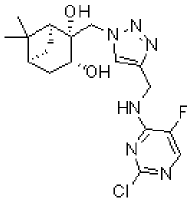

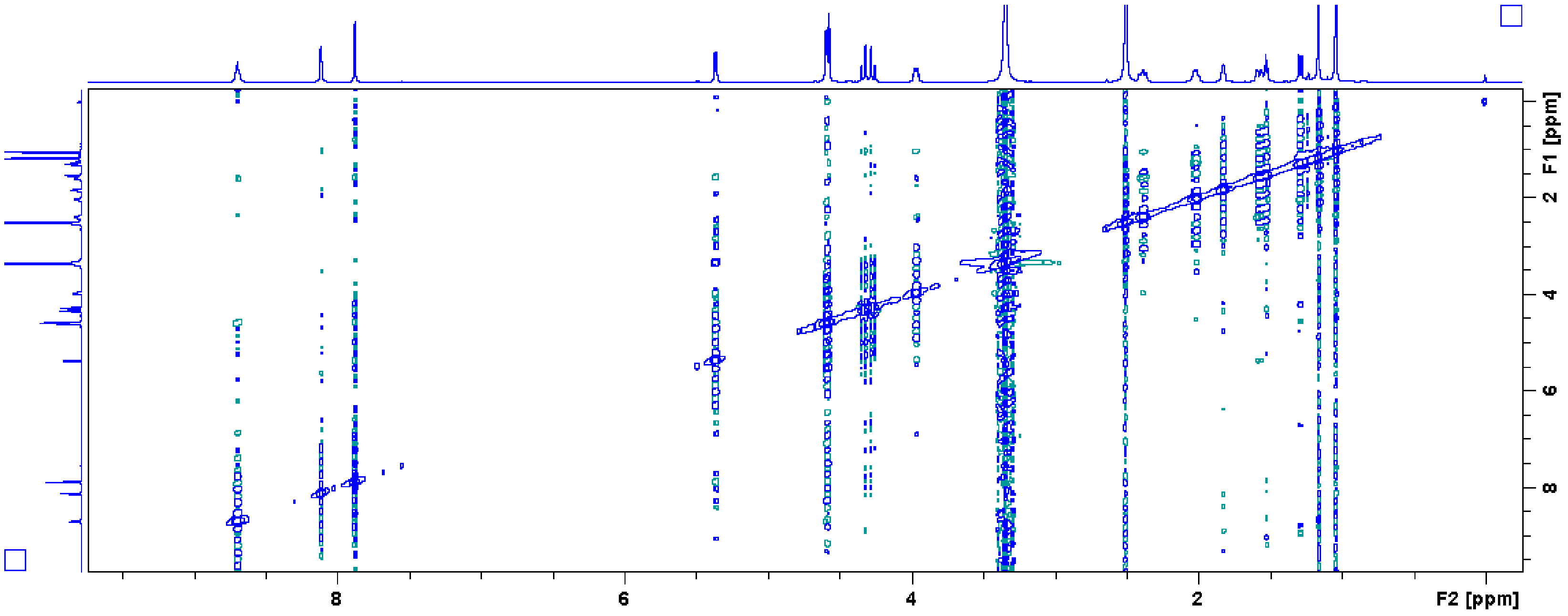

Figure S 12. HSQC-NMR of compound **(+)-20**

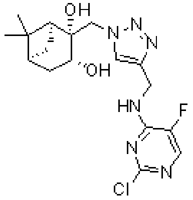

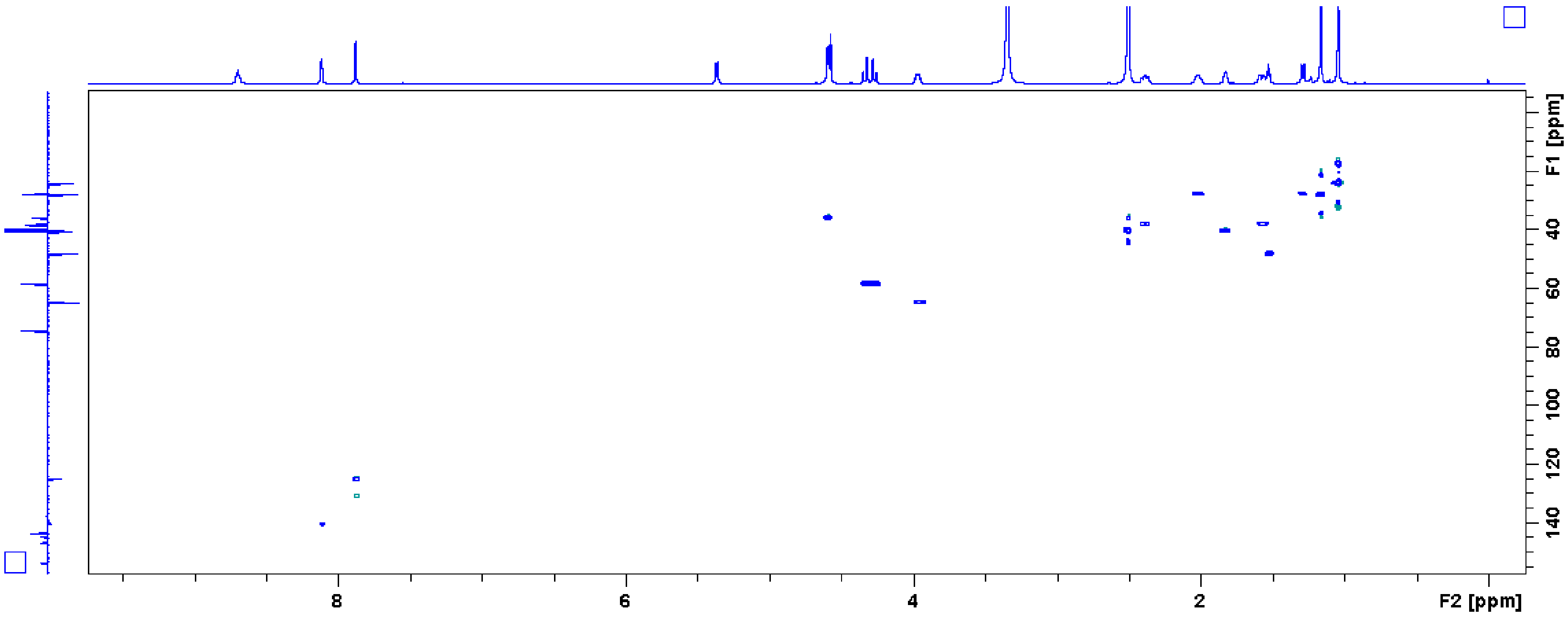

Figure S 13. HMBC-NMR of compound **(+)-20**

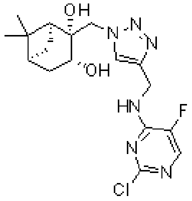

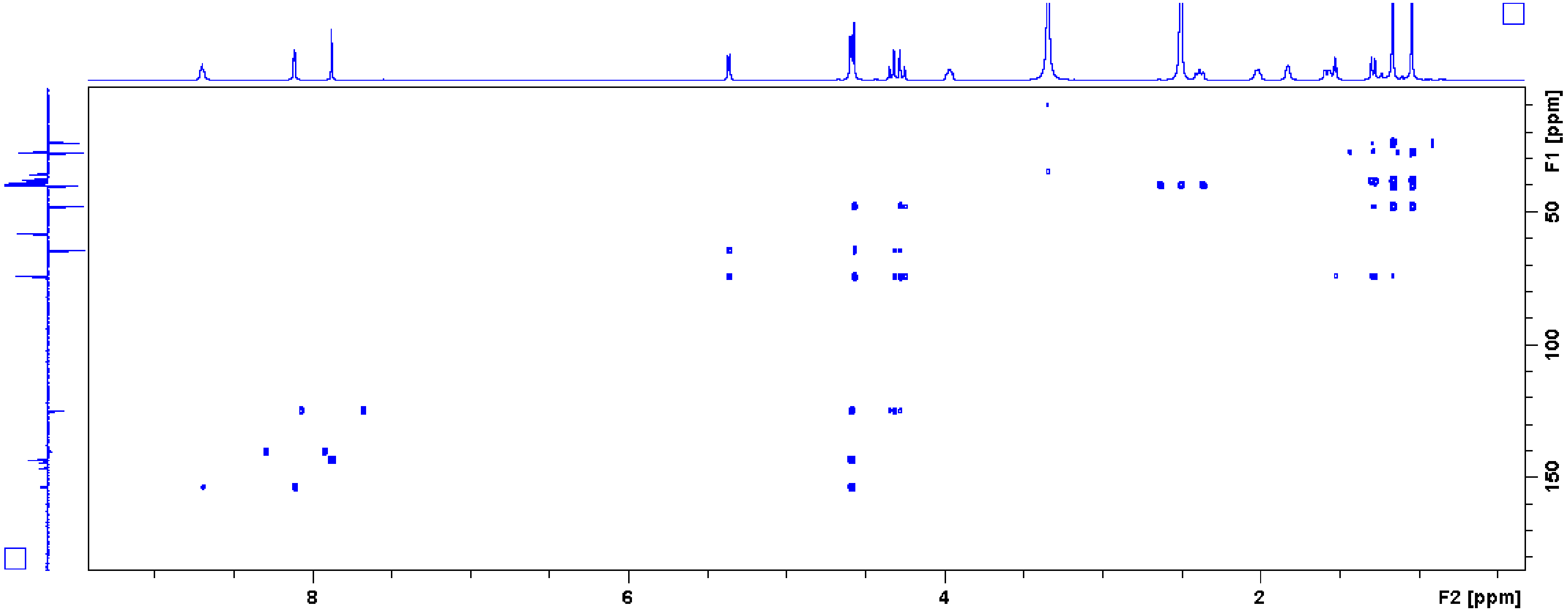

Figure S 14. ^{19}F-NMR of compound **(+)-20**

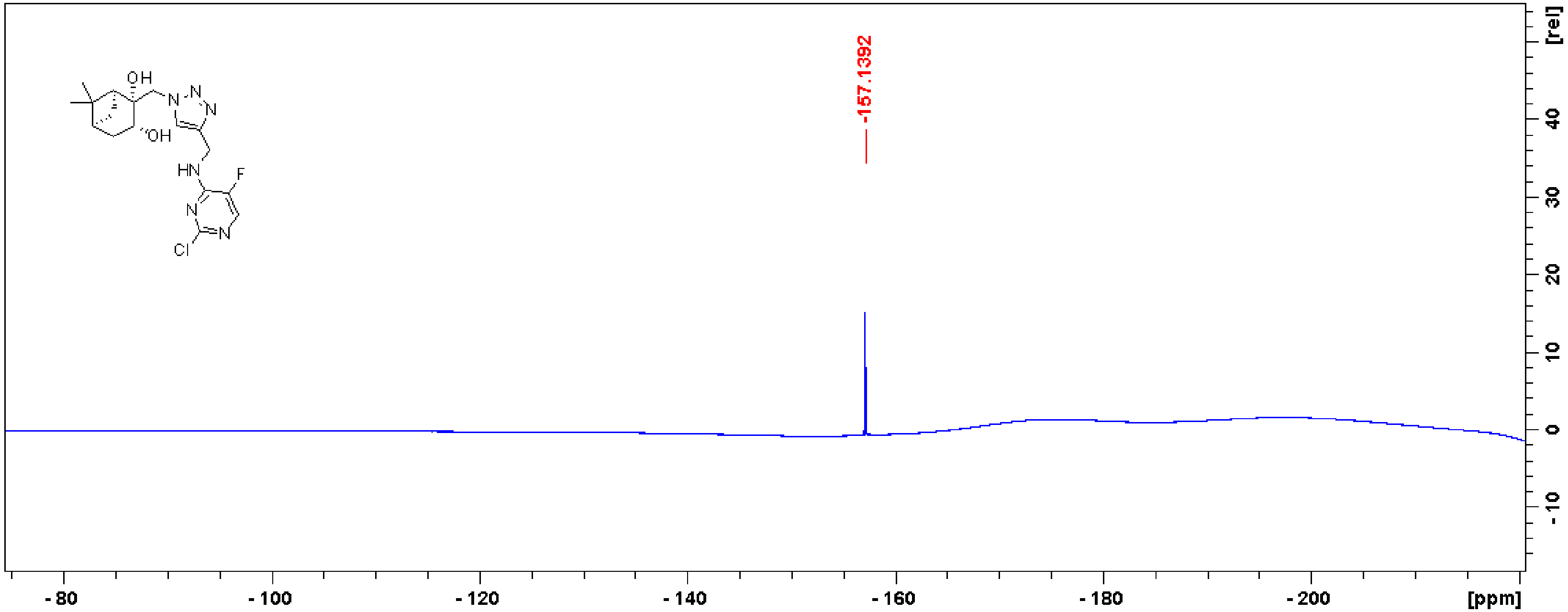

(1*R*,2*S*,3*S*,5*R*)-2-((4-(((2-Chloro-5-fluoropyrimidin-4-yl)amino)methyl)-1*H*-1,2,3-triazol-1-yl)methyl)-6,6-dimethylbicyclo[3.1.1]heptane-2,3-diol **(–)-20**

Figure S 15. ^{1}H-NMR of compound **(–)-20**

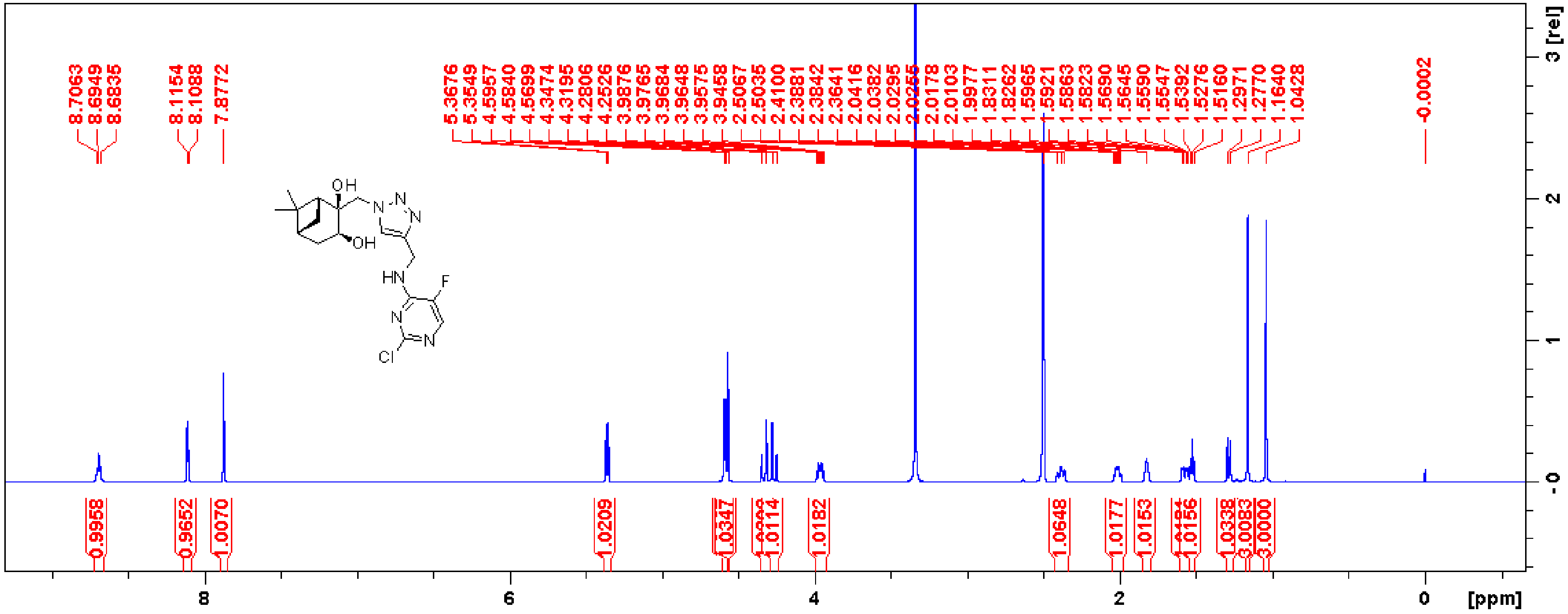

Figure S 16. ^{13}C-NMR of compound **(–)-20**

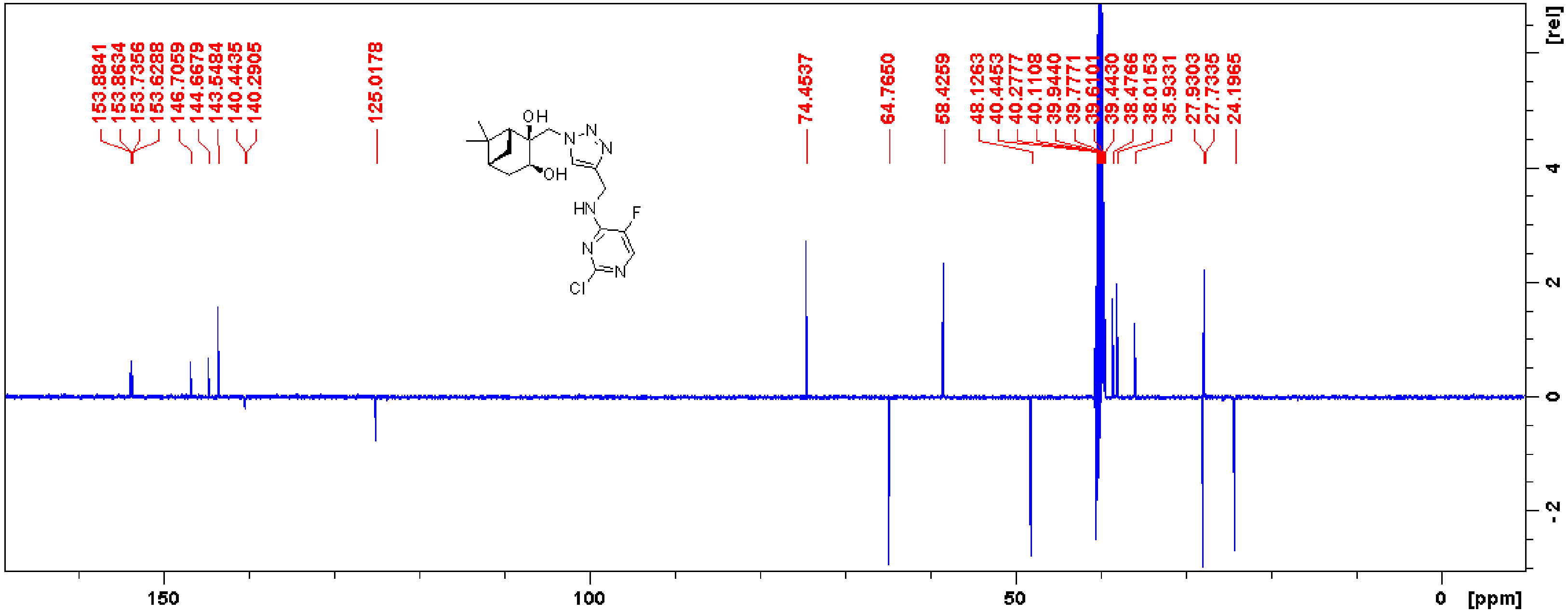

Figure S 17. COSY-NMR of compound **(–)-20**

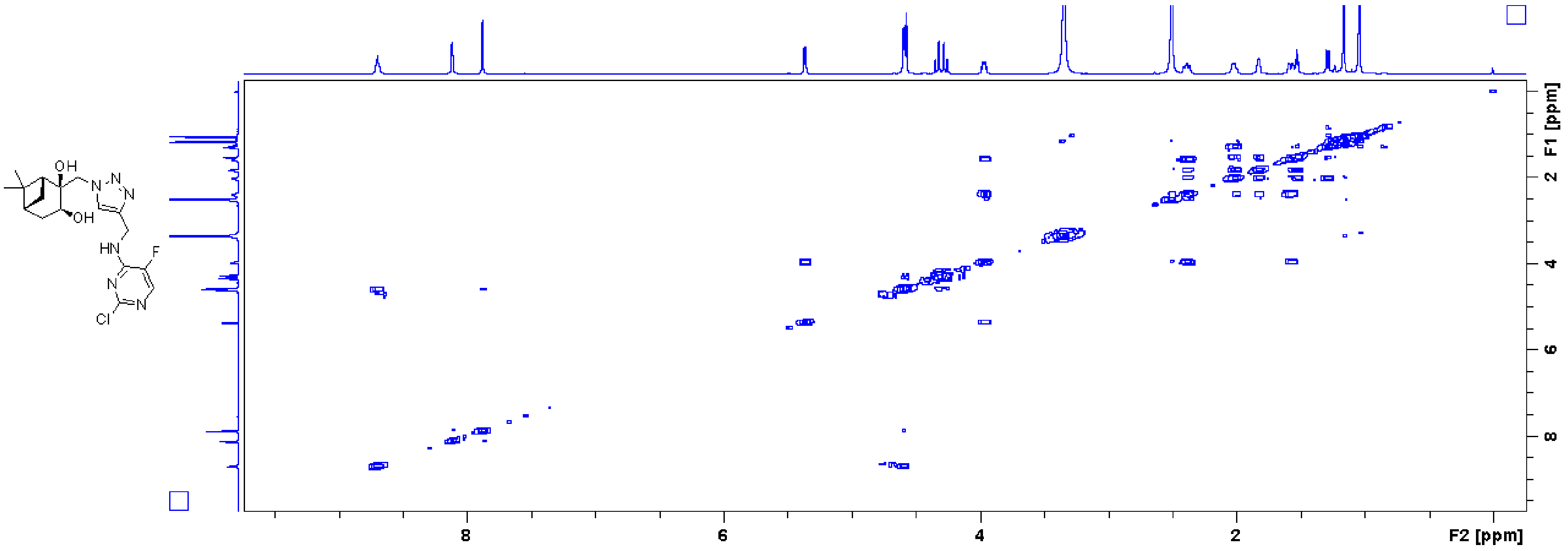

Figure S 18. NOESY-NMR of compound **(–)-20**

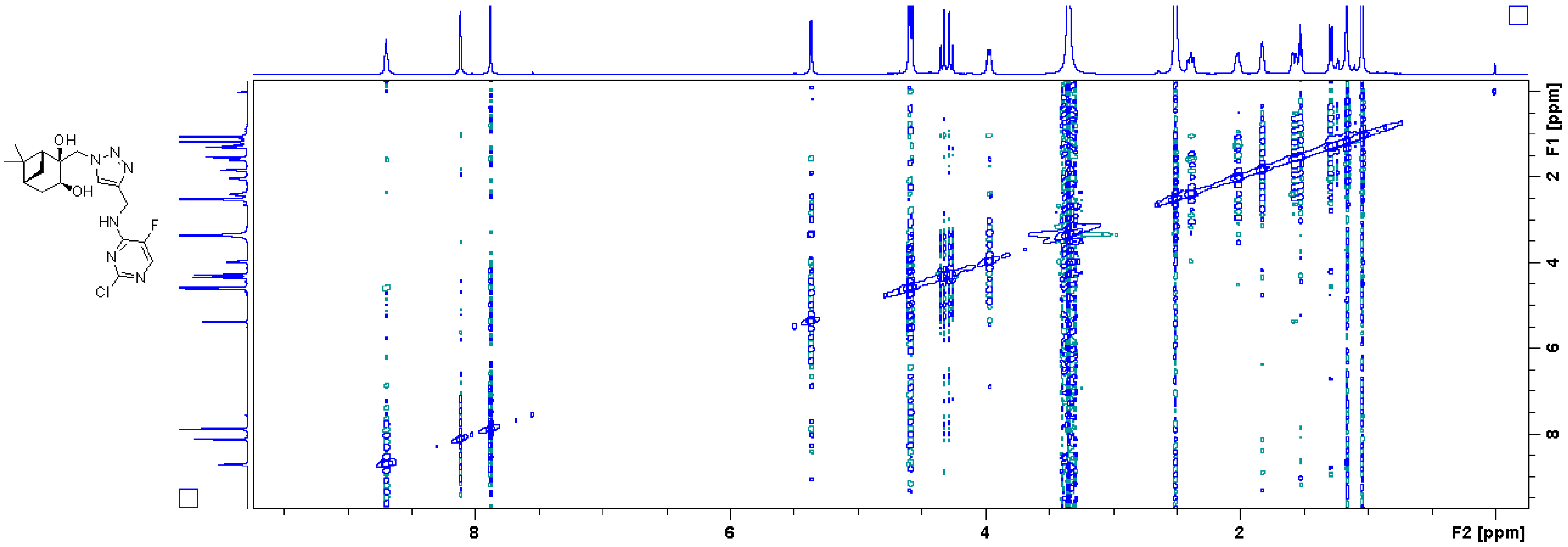

Figure S 19. HSQC-NMR of compound **(–)-20**

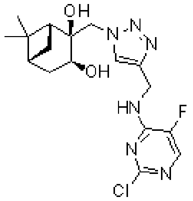

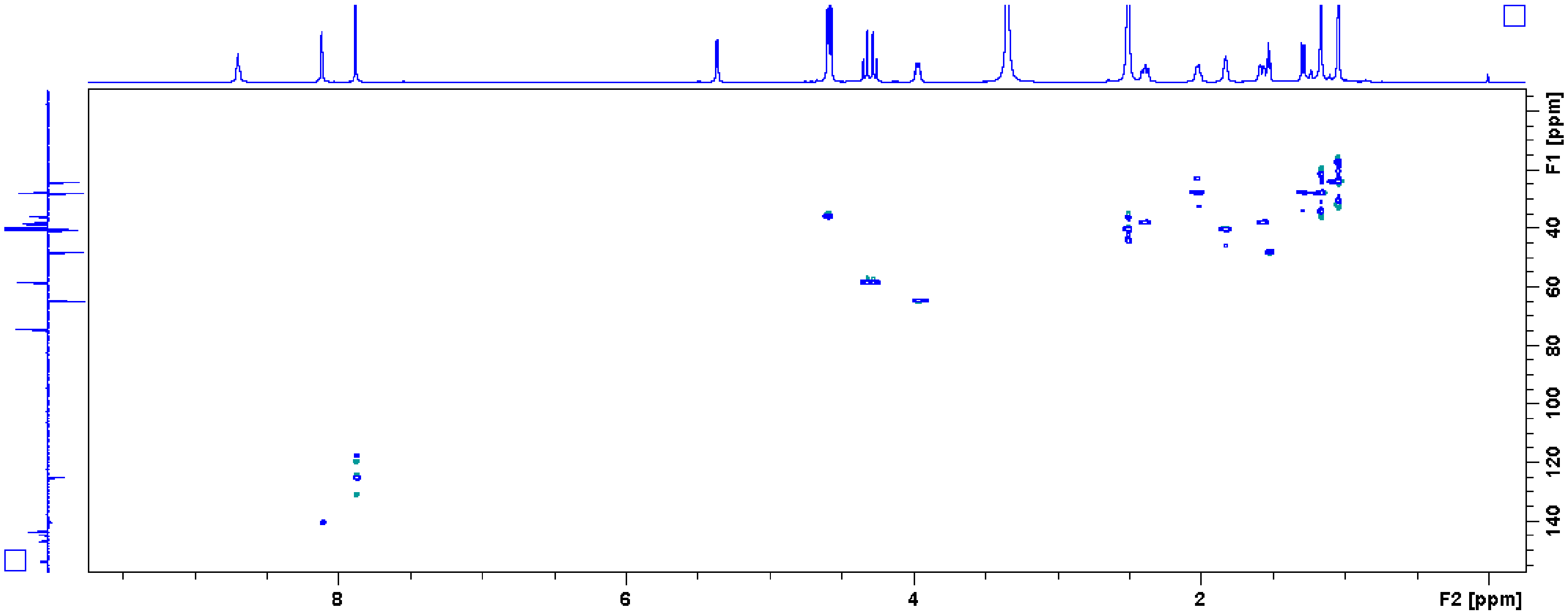

Figure S 20. HMBC-NMR of compound **(–)-20**

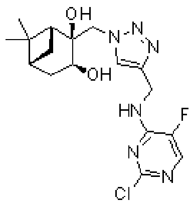

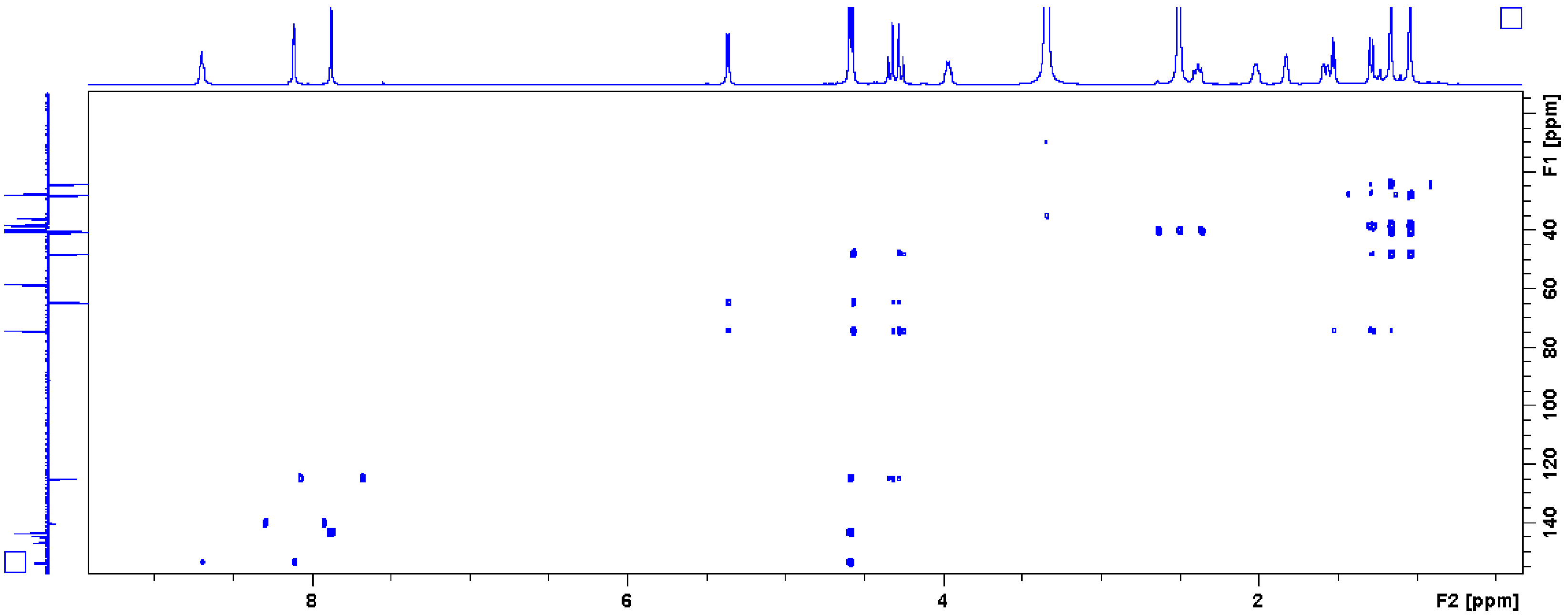

Figure S 21. ^{19}F-NMR of compound **(–)-20**

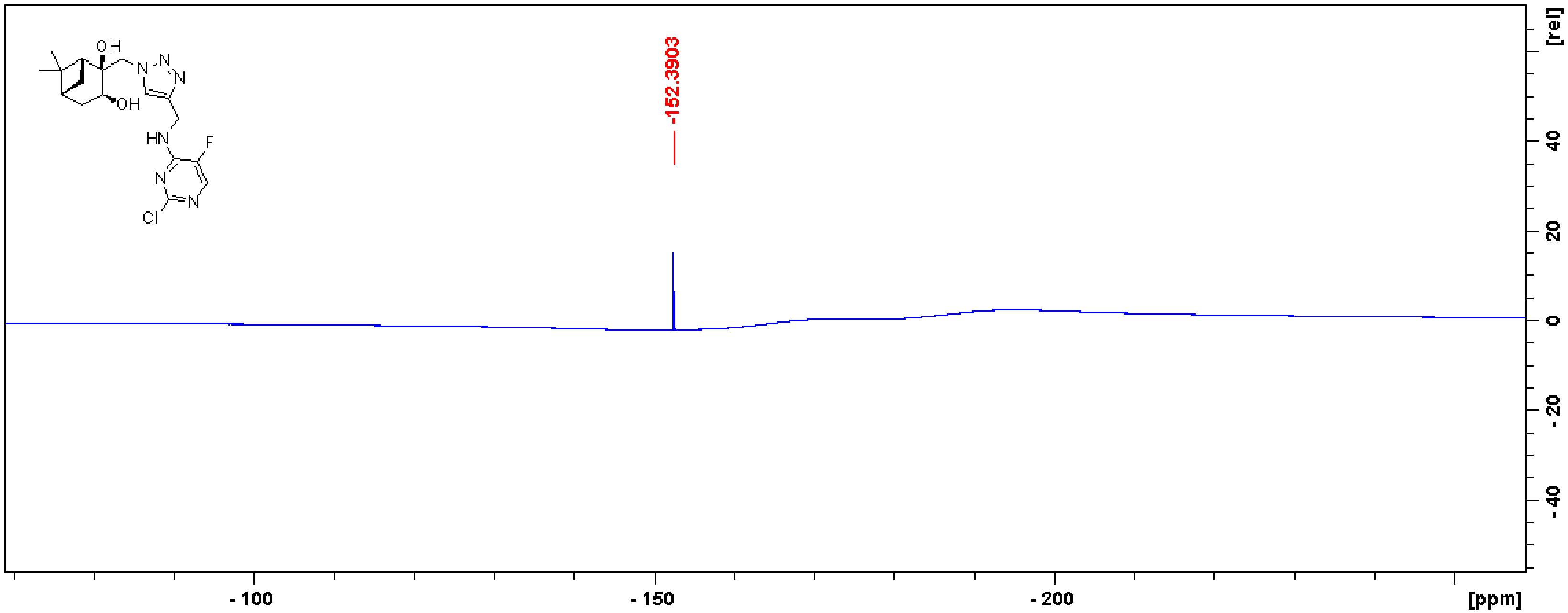

(1*S*,2*R*,3*R*,5*S*)-2-((4-(((2,5-Dichloropyrimidin-4-yl)amino)methyl)-1*H*-1,2,3-triazol-1-yl)methyl)-6,6-dimethylbicyclo[3.1.1]heptane-2,3-diol **(+)-21**

Figure S 22. ^{1}H-NMR of compound **(+)-21**

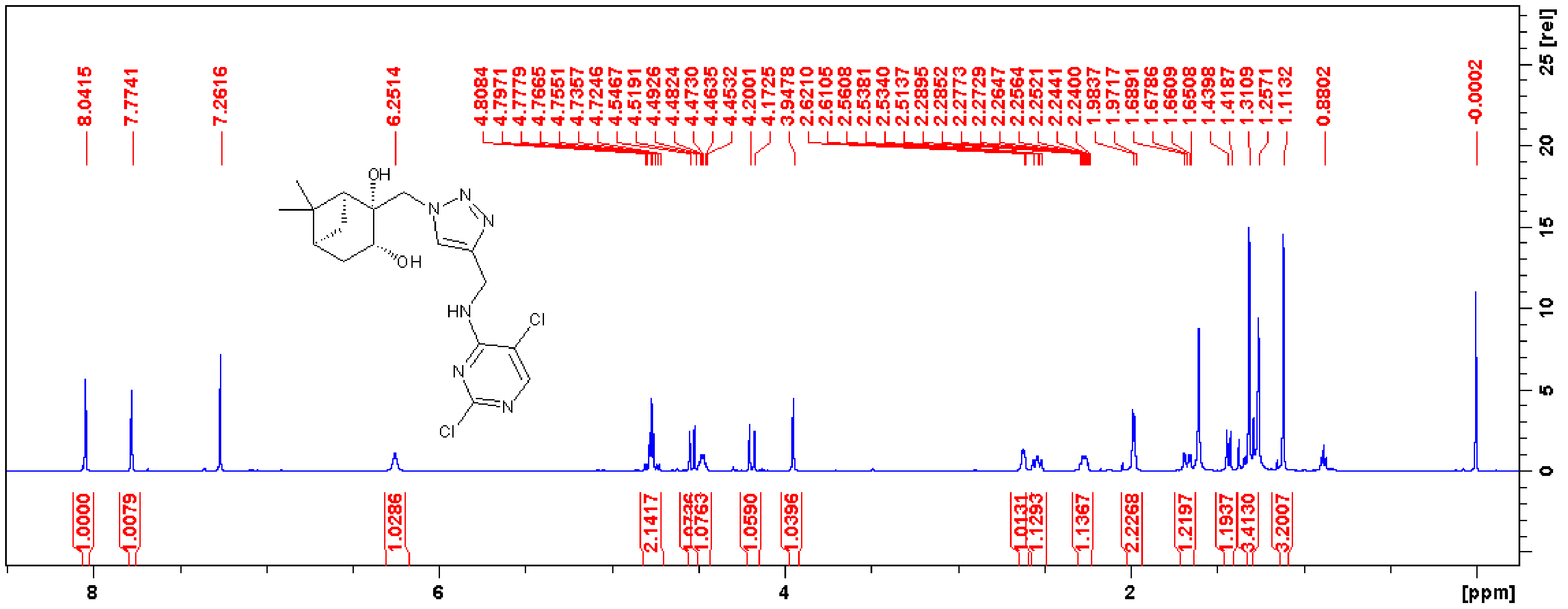

Figure S 23. ^{13}C-NMR of compound **(+)-21**

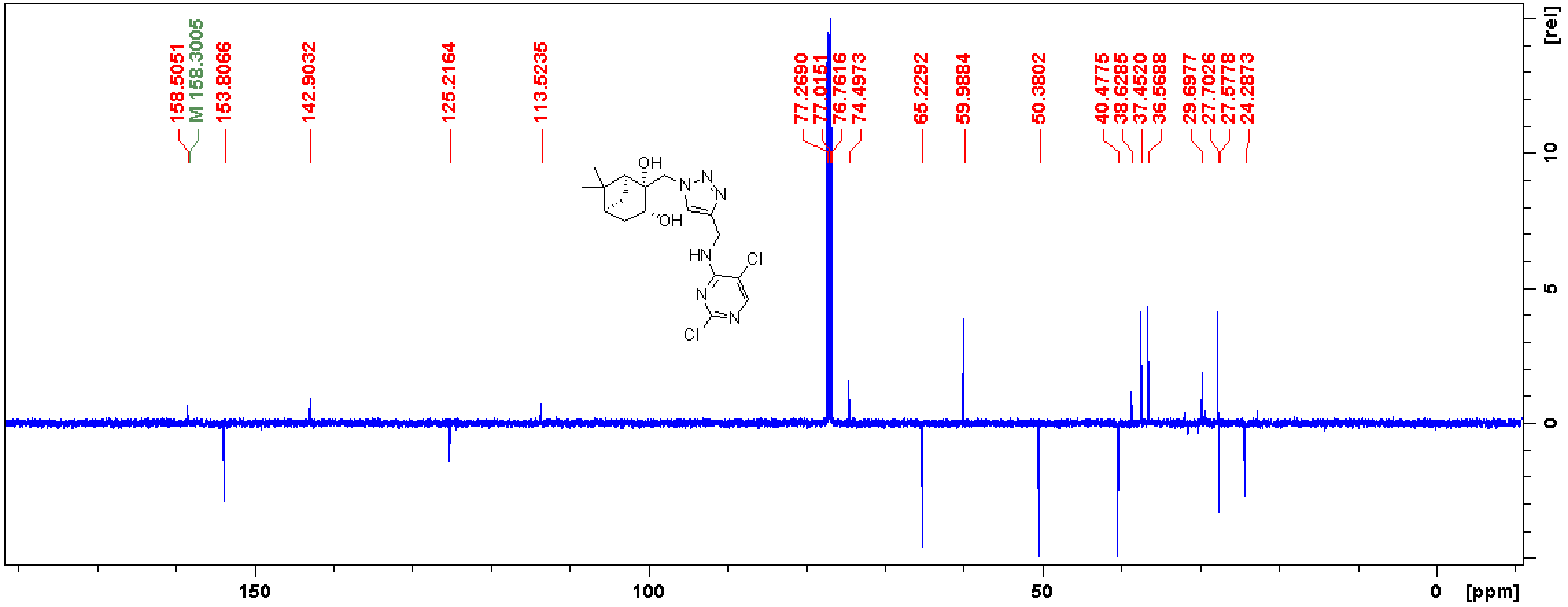

Figure S 24. COSY-NMR of compound **(+)-21**

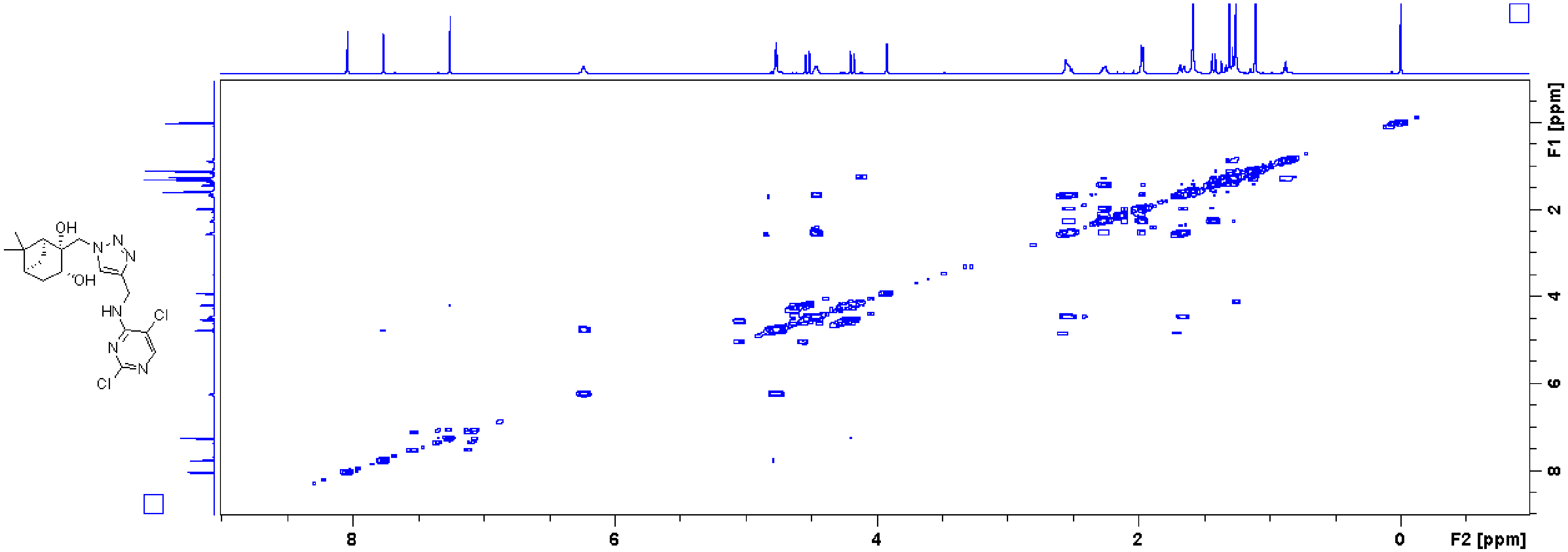

Figure S 25. NOESY-NMR of compound **(+)-21**

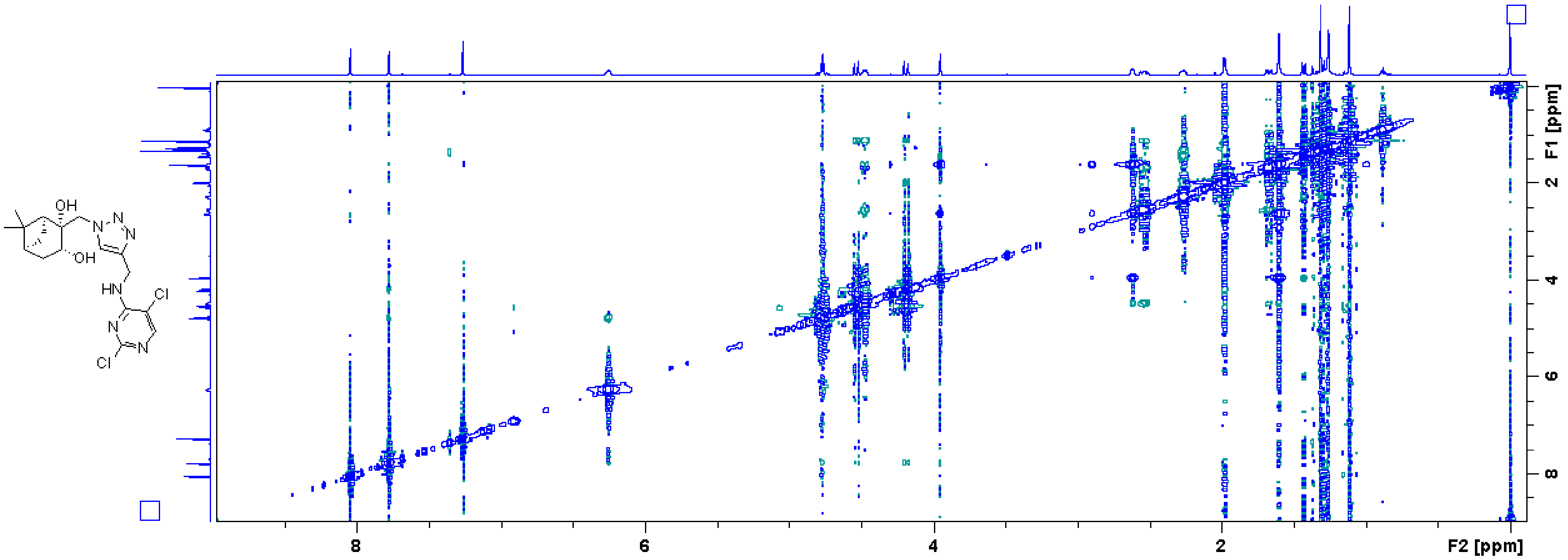

Figure S 26. HSQC-NMR of compound **(+)-21**

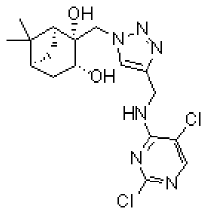

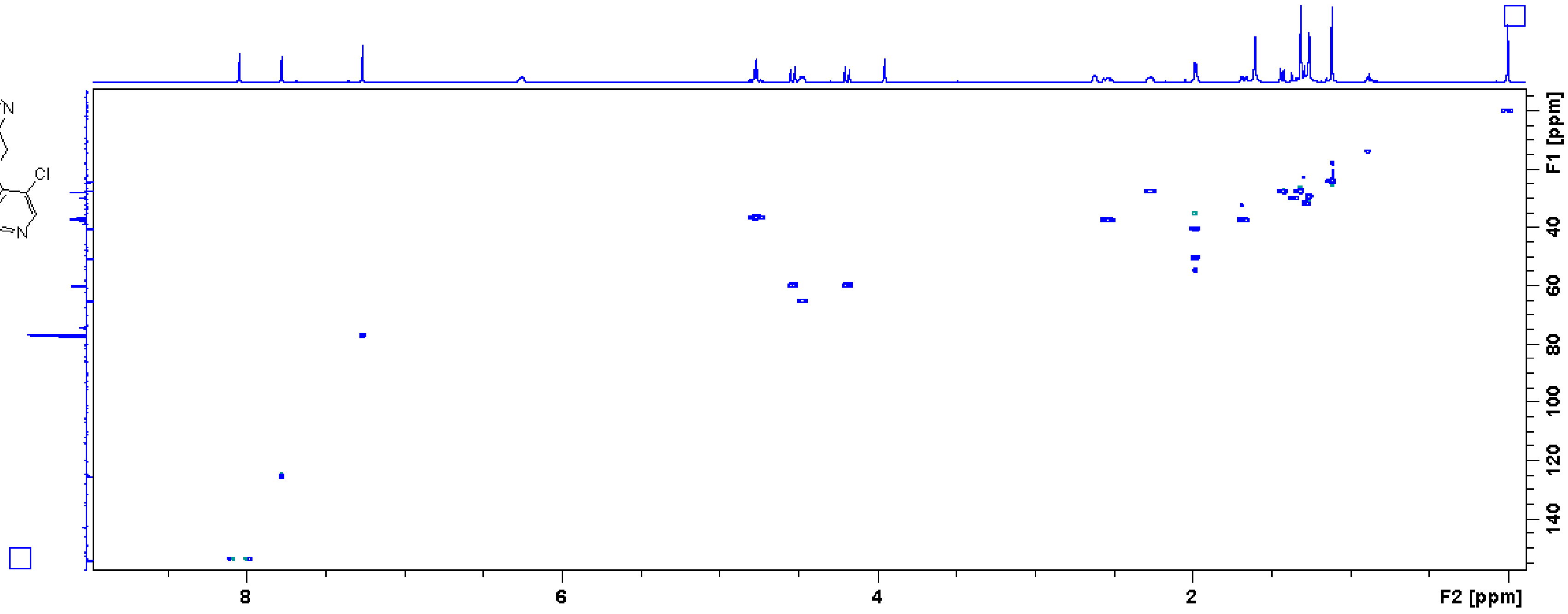

Figure S 27. HMBC-NMR of compound **(+)-21**

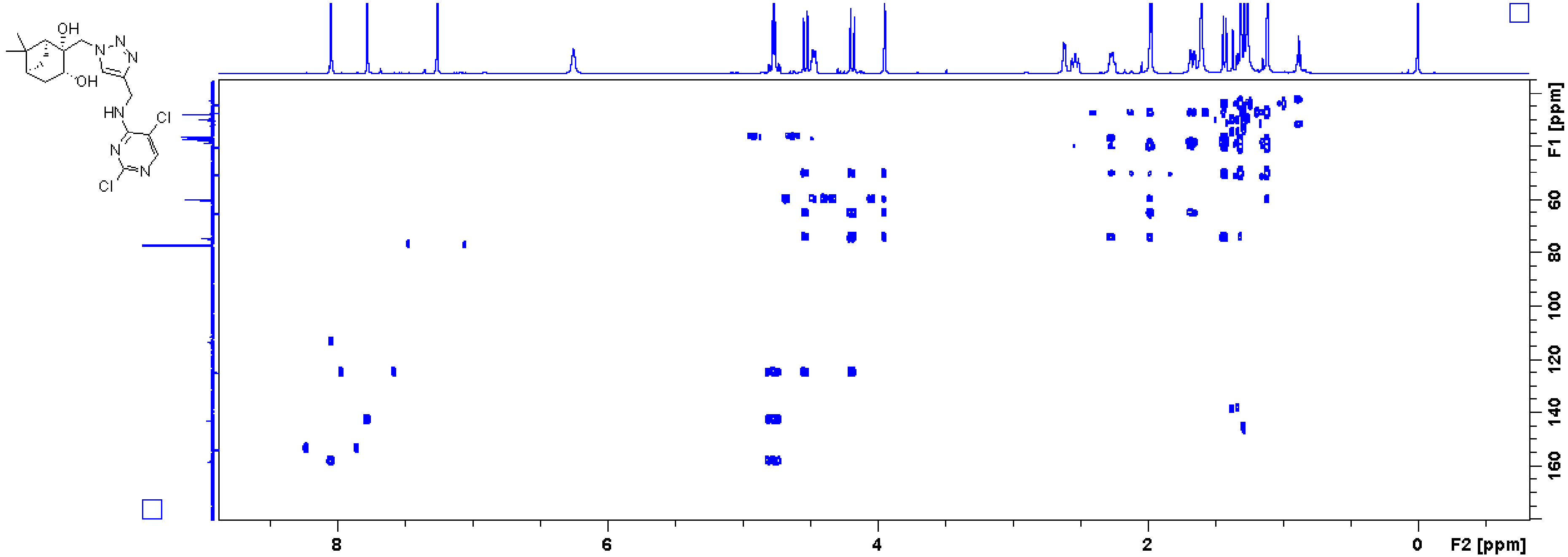

(1*R*,2*S*,3*S*,5*R*)-2-((4-(((2,5-Dichloropyrimidin-4-yl)amino)methyl)-1*H*-1,2,3-triazol-1-yl)methyl)-6,6-dimethylbicyclo[3.1.1]heptane-2,3-diol **(–)-21**

Figure S 28. ^{1}H-NMR of compound **(–)-21**

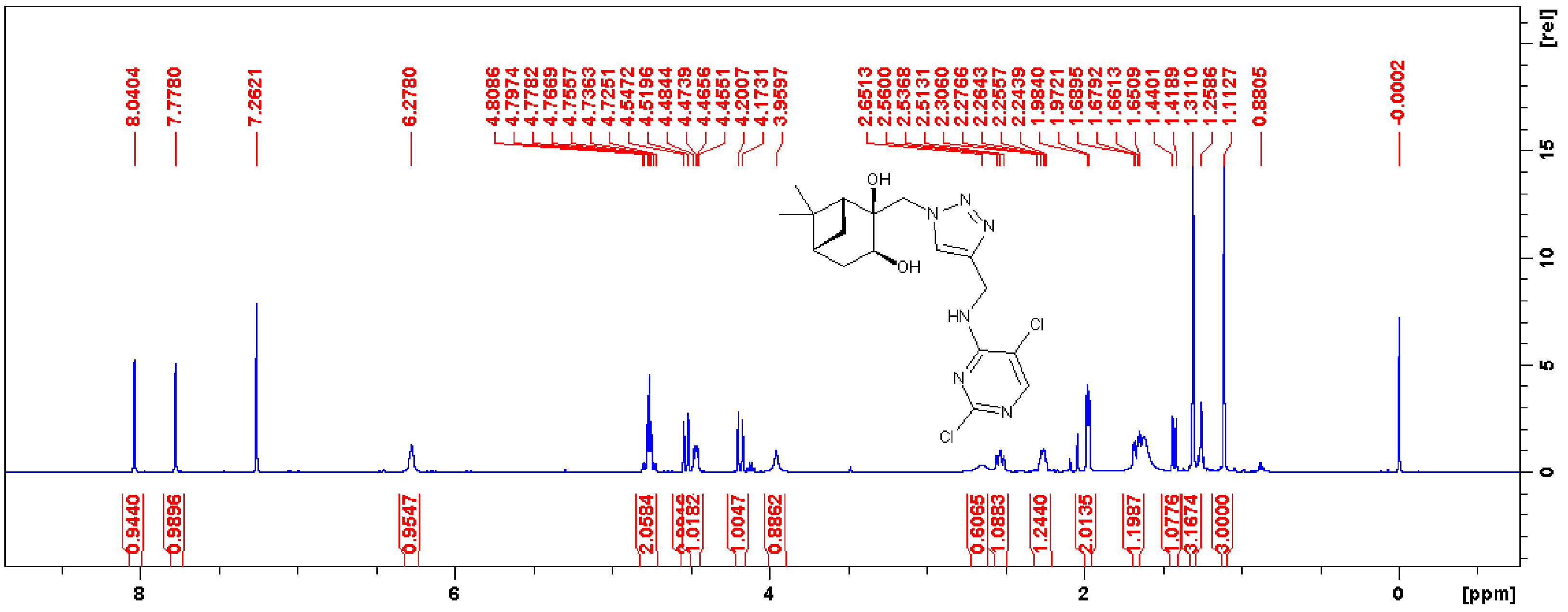

Figure S 29. ^{13}C-NMR of compound **(–)-21**

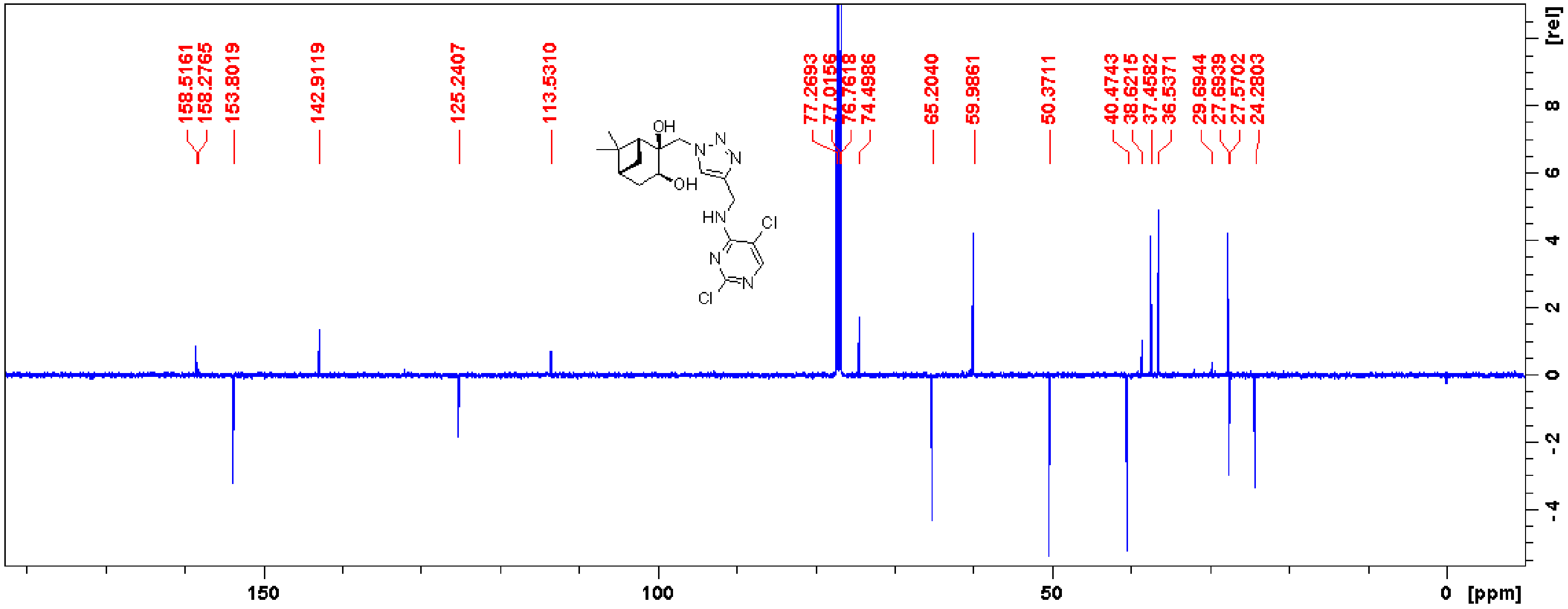

Figure S 30. COSY-NMR of compound **(–)-21**

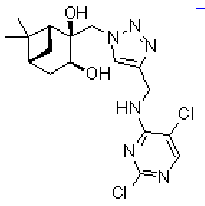

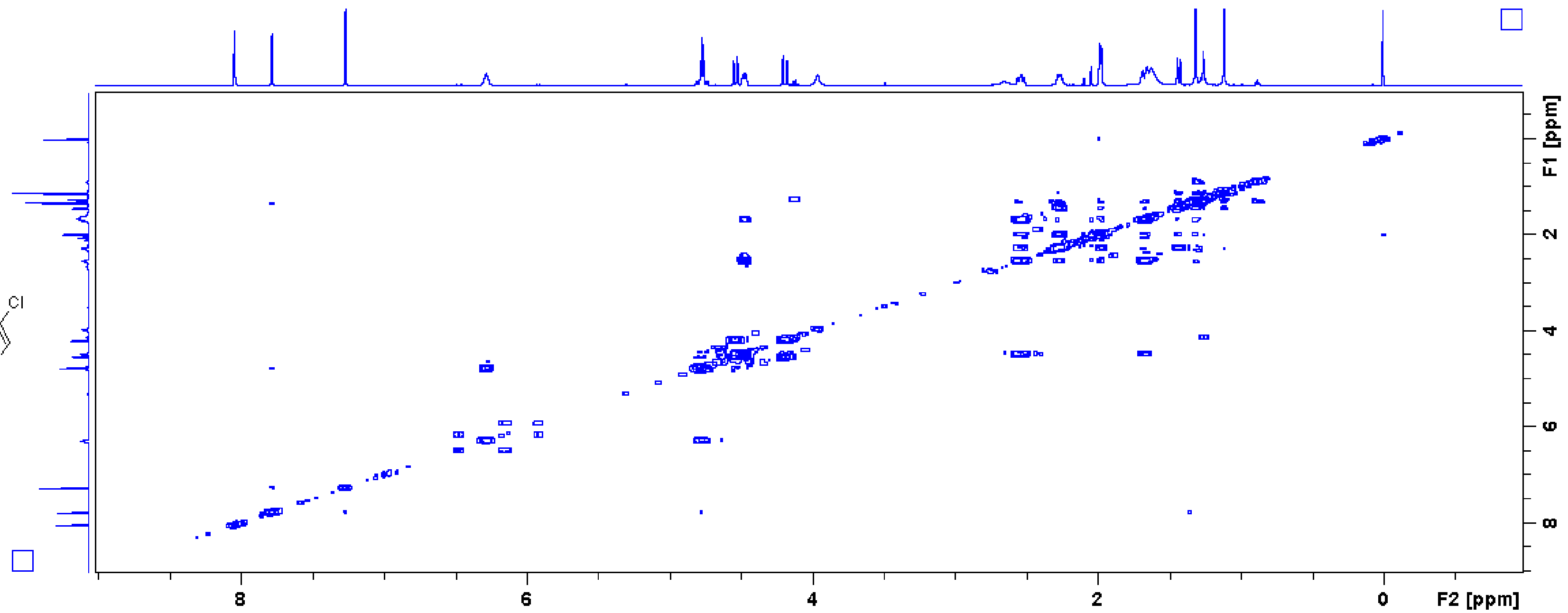

Figure S 31. NOESY-NMR of compound **(–)-21**

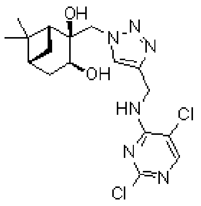

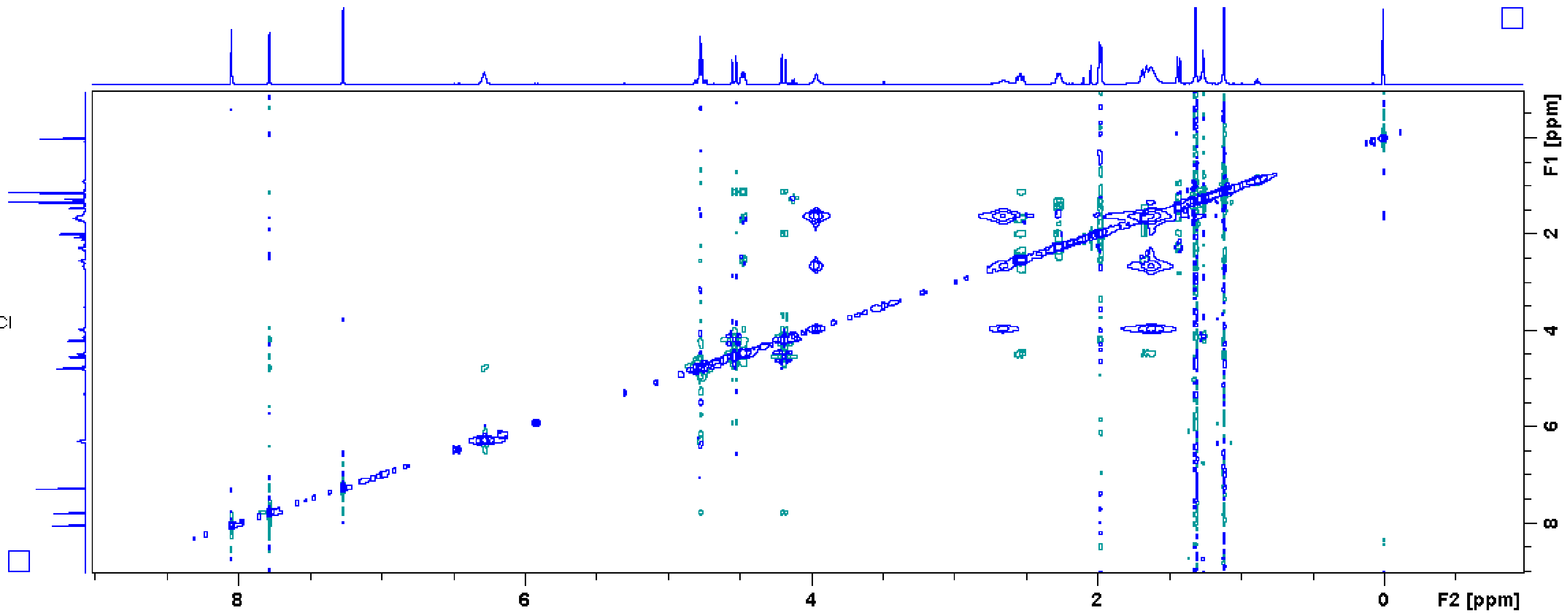

Figure S 32. HSQC-NMR of compound **(–)-21**

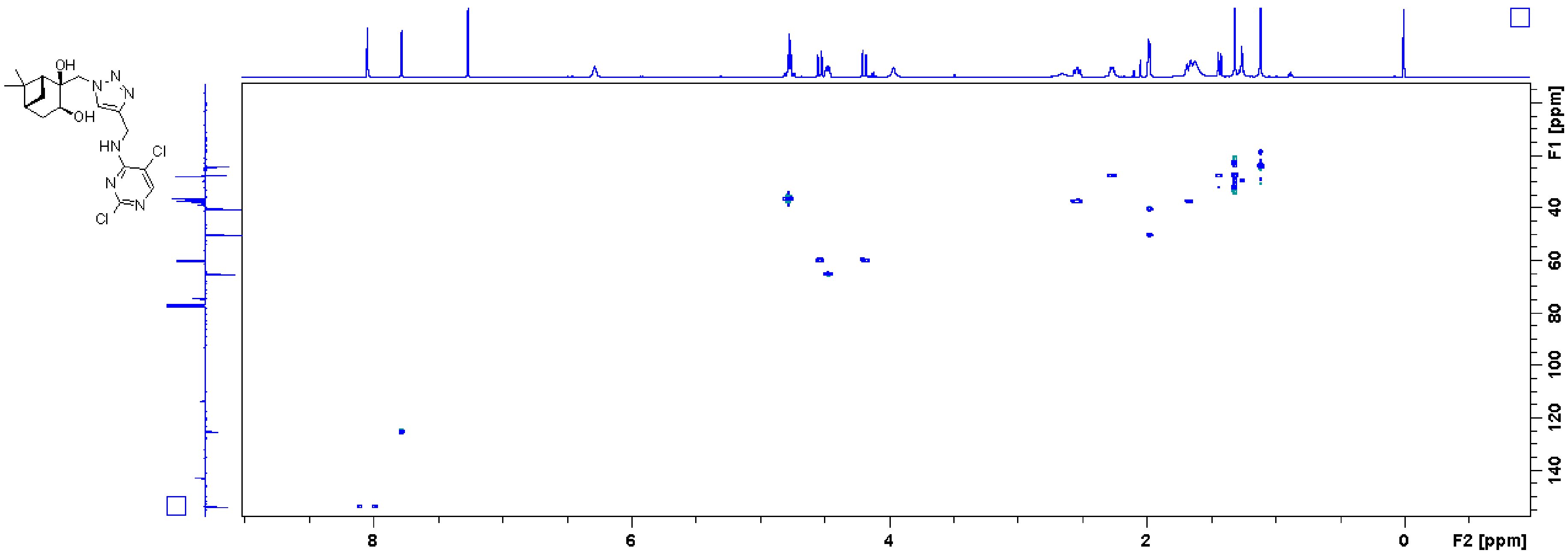

Figure S 33. HMBC-NMR of compound **(–)-21**

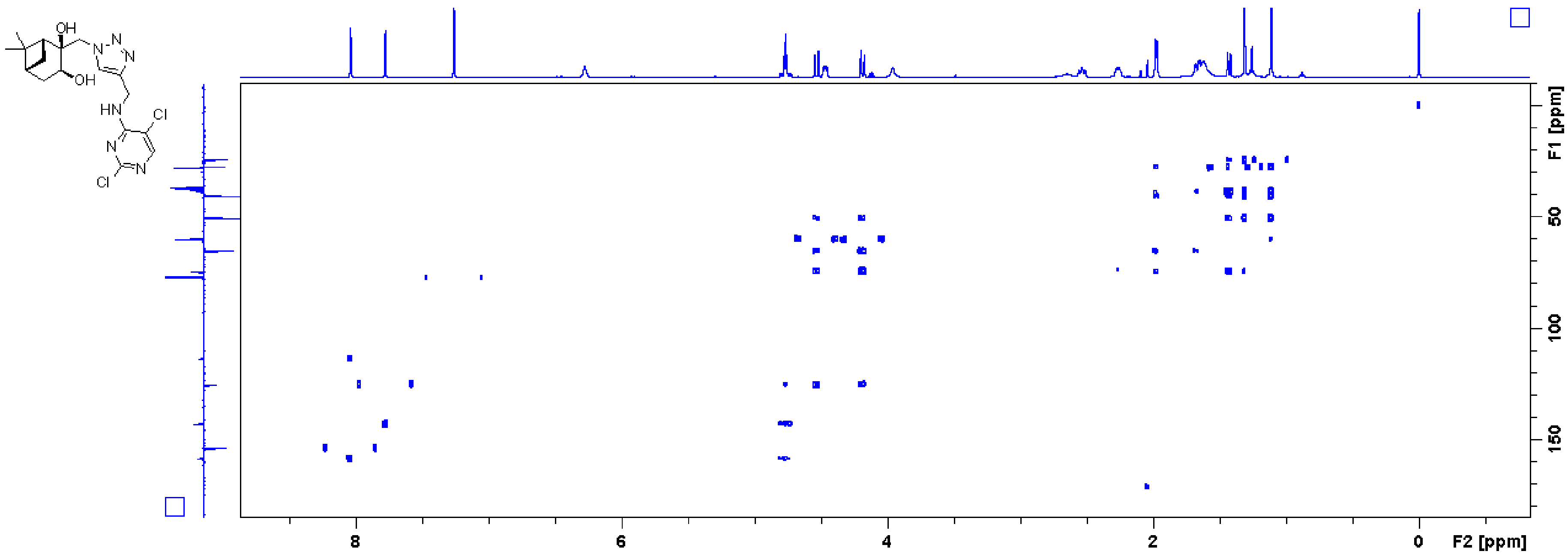

(1*S*,2*R*,3*R*,5*S*)-2-((4-(((5-Amino-6-chloropyrimidin-4-yl)amino)methyl)-1*H*-1,2,3-triazol-1-yl)methyl)-6,6-dimethylbicyclo[3.1.1]heptane-2,3-diol **(+)-22**

Figure S 34. ^{1}H-NMR of compound **(+)-22**

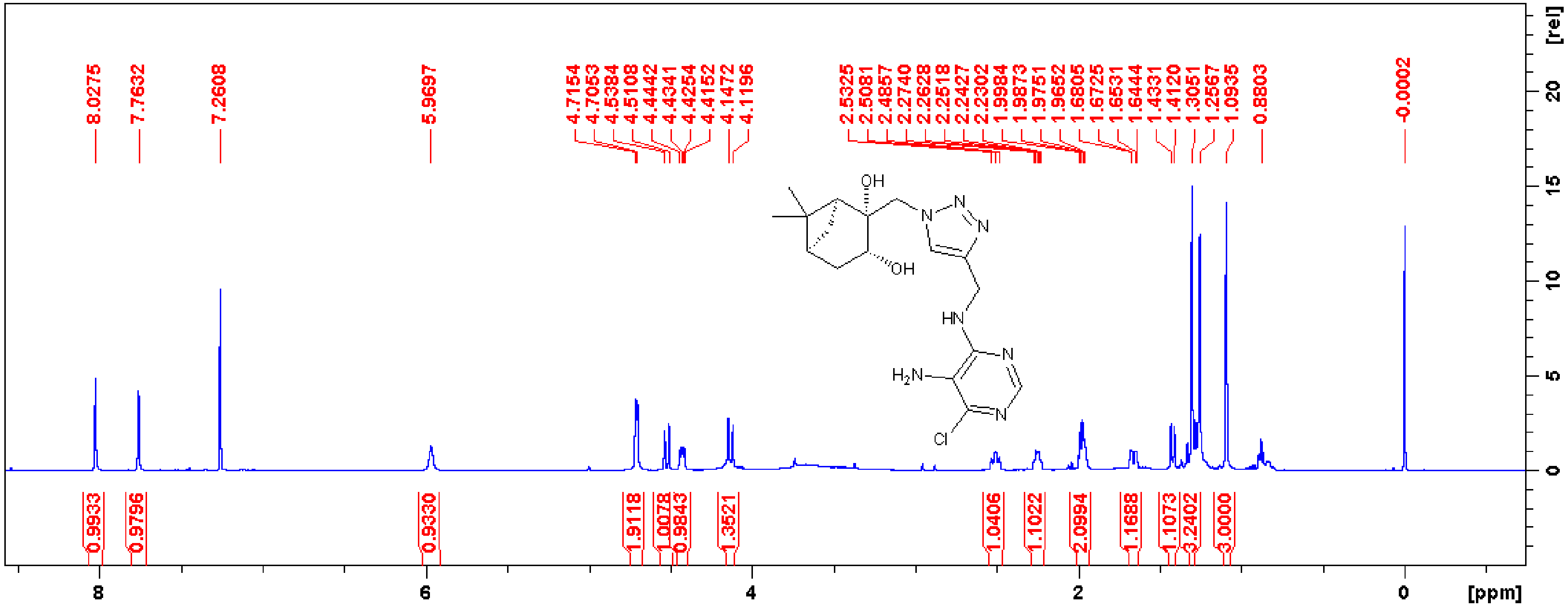

Figure S 35. ^{13}C-NMR of compound **(+)-22**

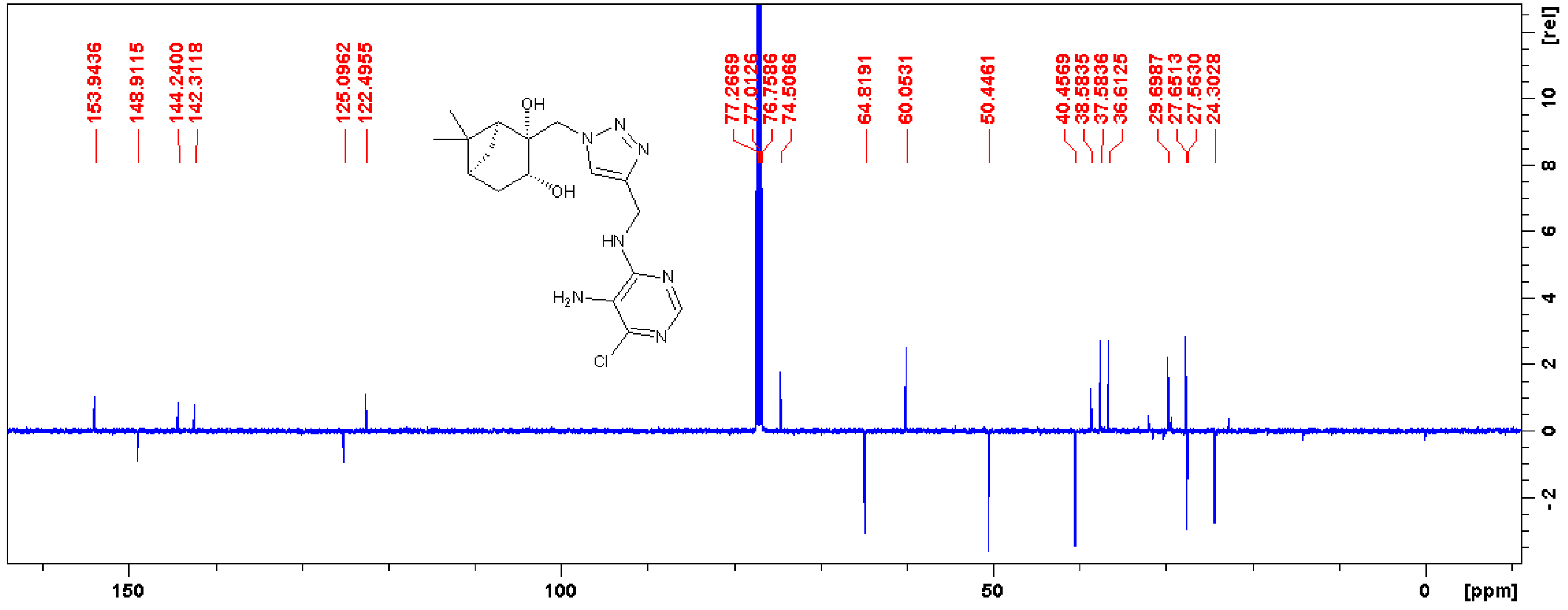

Figure S 36. COSY-NMR of compound **(+)-22**

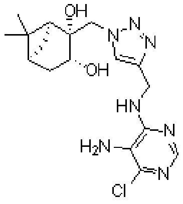

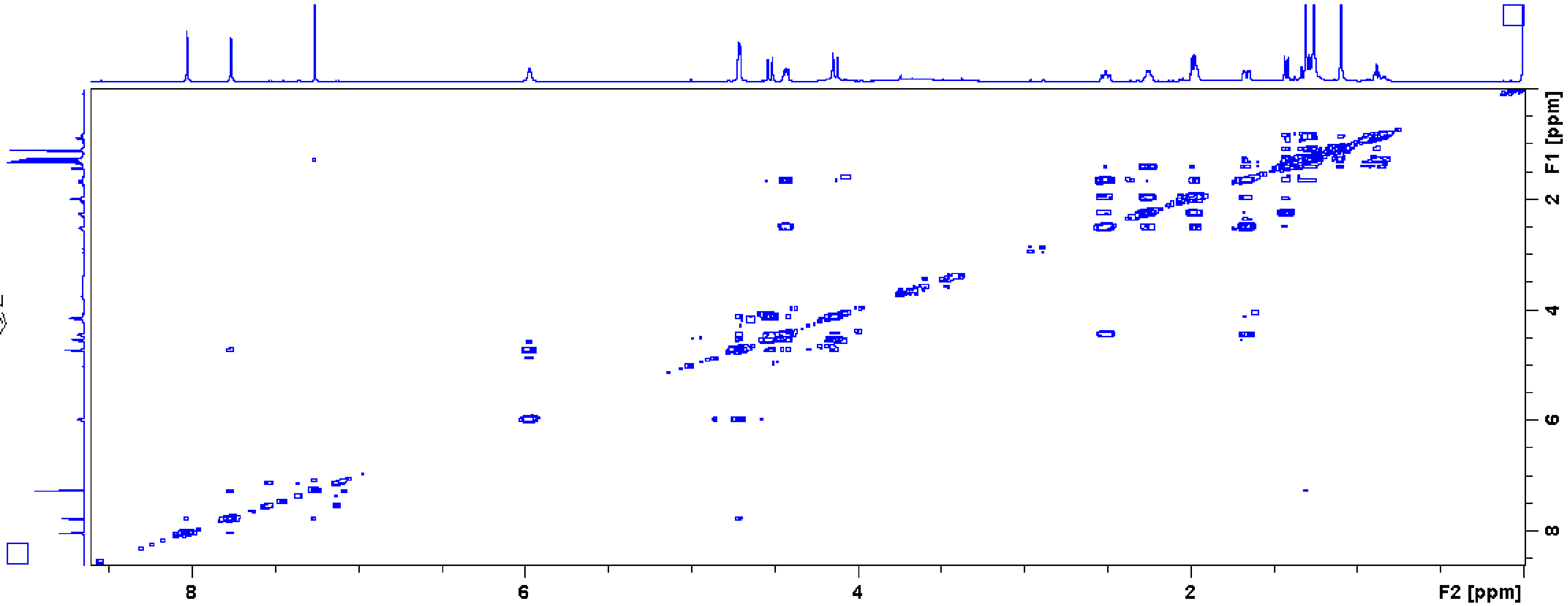

Figure S 37. NOESY-NMR of compound **(+)-22**

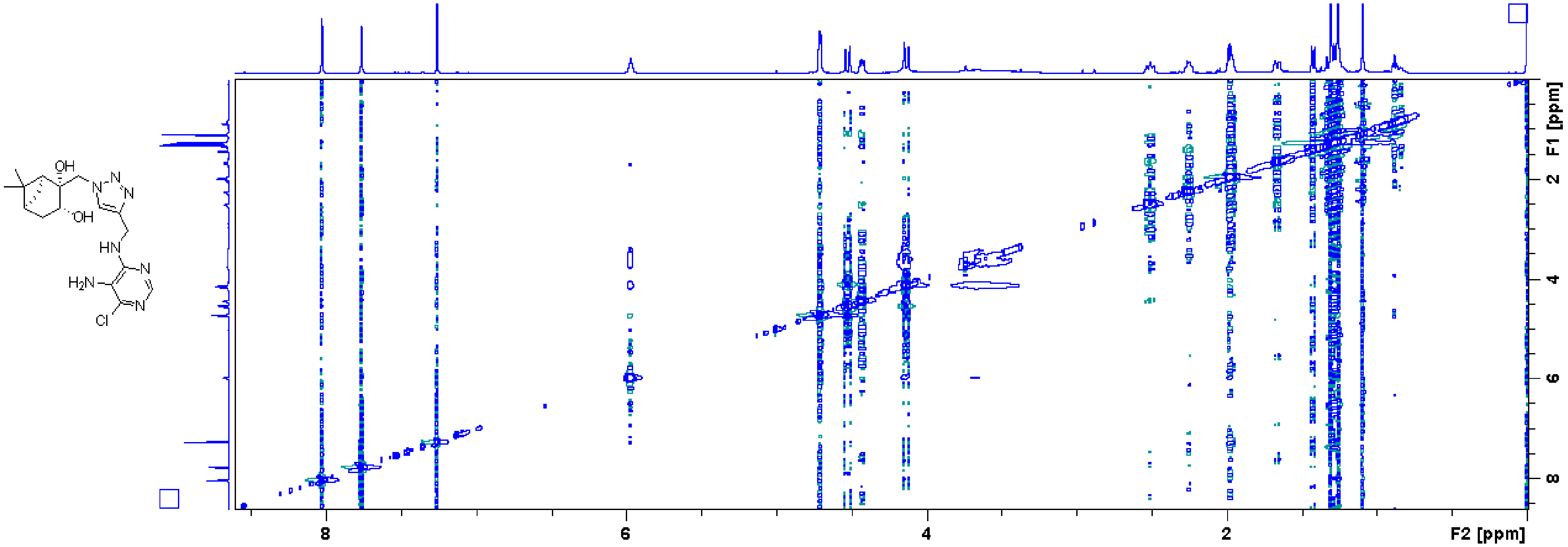

Figure S 38. HSQC-NMR of compound **(+)-22**

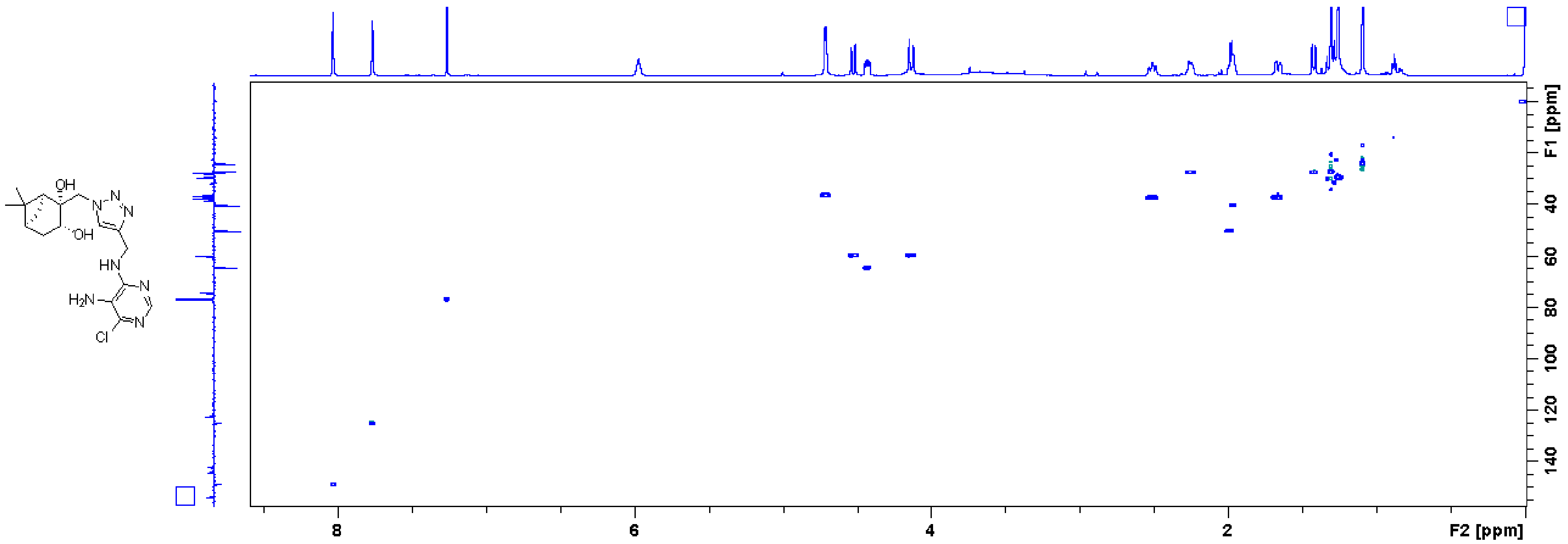

Figure S 39. HMBC-NMR of compound **(+)-22**

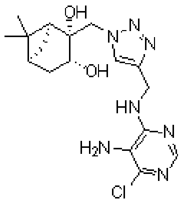

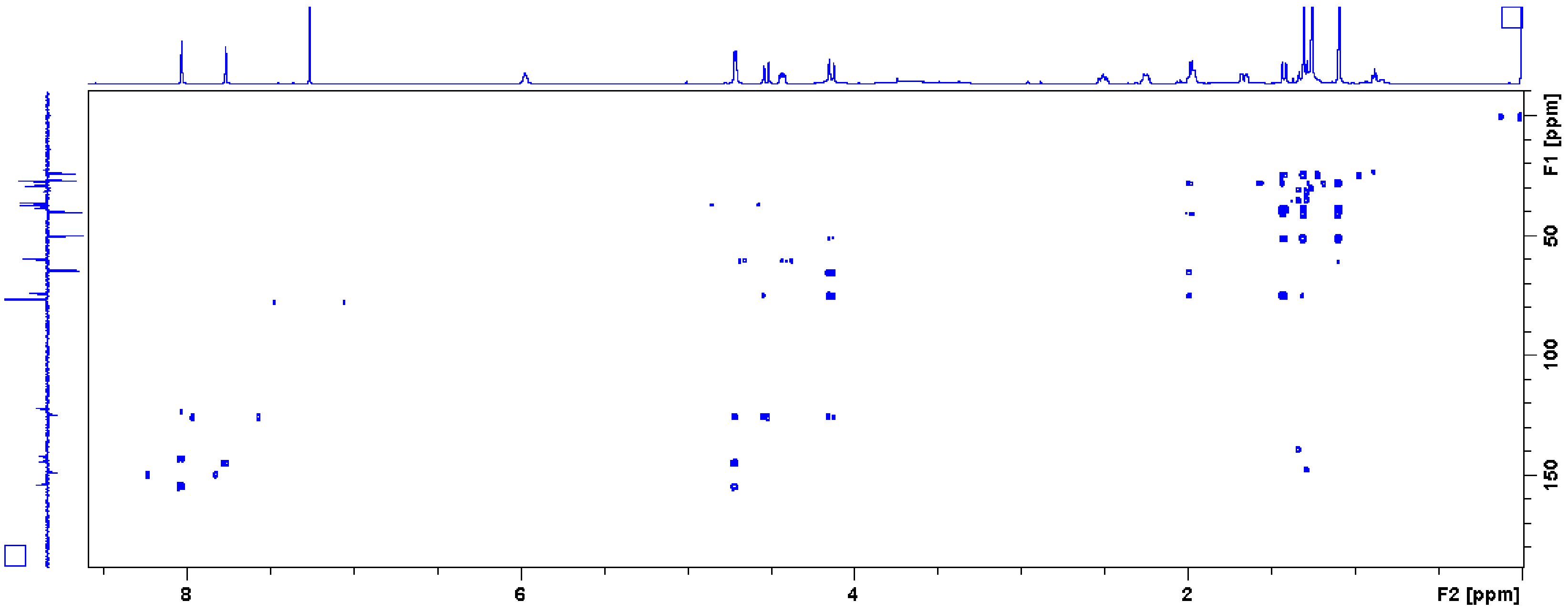

(1*R*,2*S*,3*S*,5*R*)-2-((4-(((5-Amino-6-chloropyrimidin-4-yl)amino)methyl)-1*H*-1,2,3-triazol-1-yl)methyl)-6,6-dimethylbicyclo[3.1.1]heptane-2,3-diol **(–)-22**

Figure S 40. ^{1}H-NMR of compound **(–)-22**

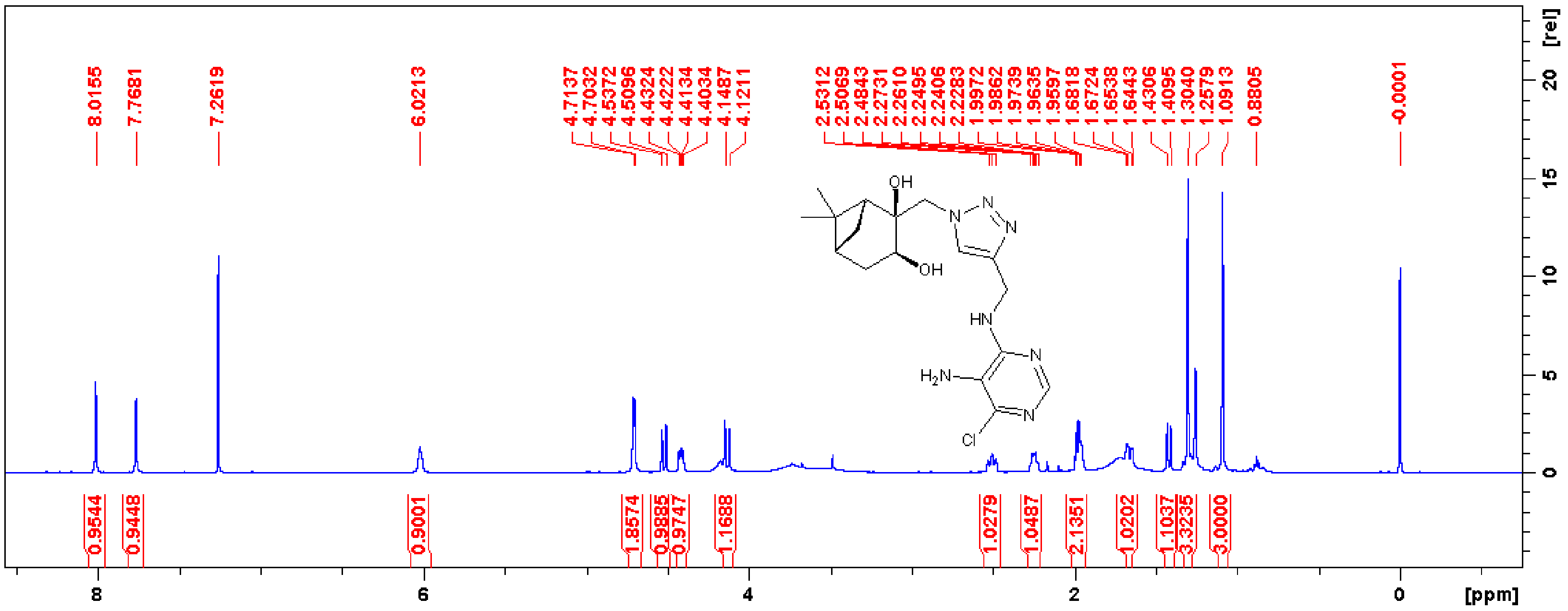

Figure S 41. ^{13}C-NMR of compound **(–)-22**

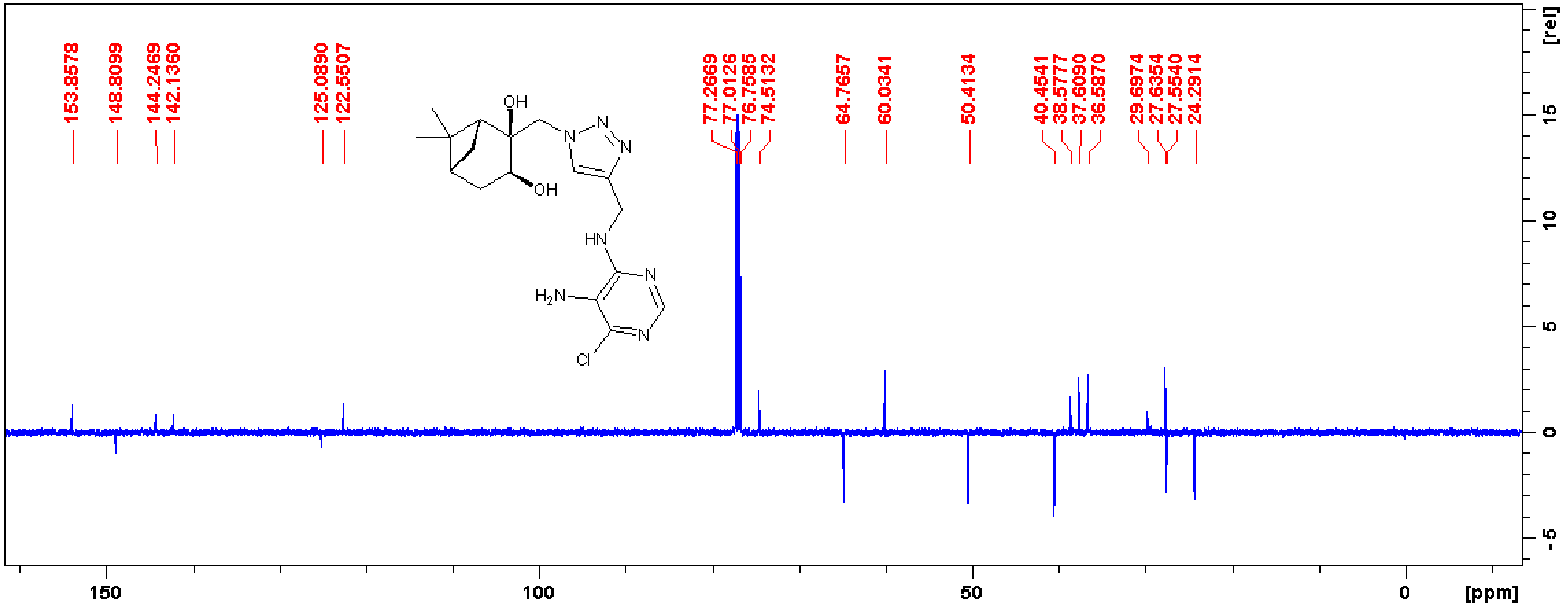

Figure S 42. COSY-NMR of compound **(–)-22**

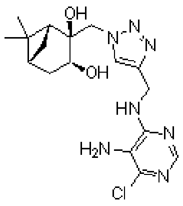

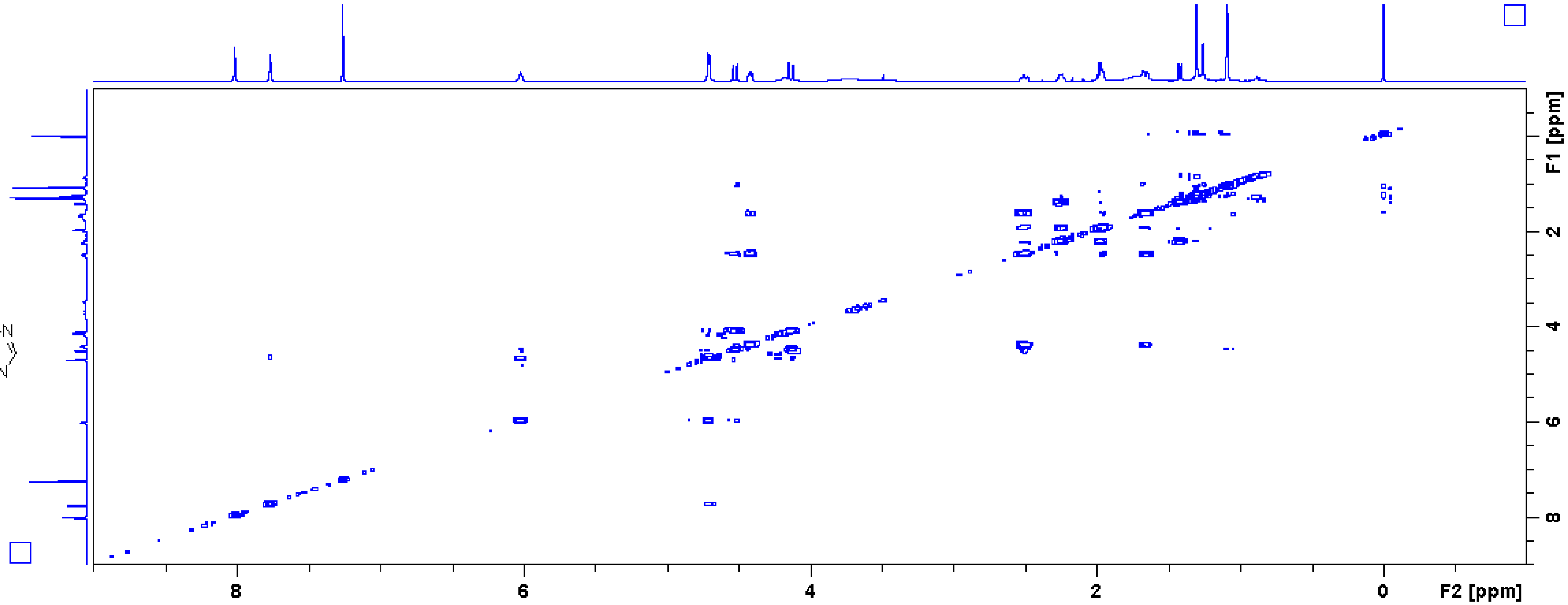

Figure S 43. NOESY-NMR of compound **(–)-22**

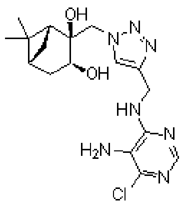

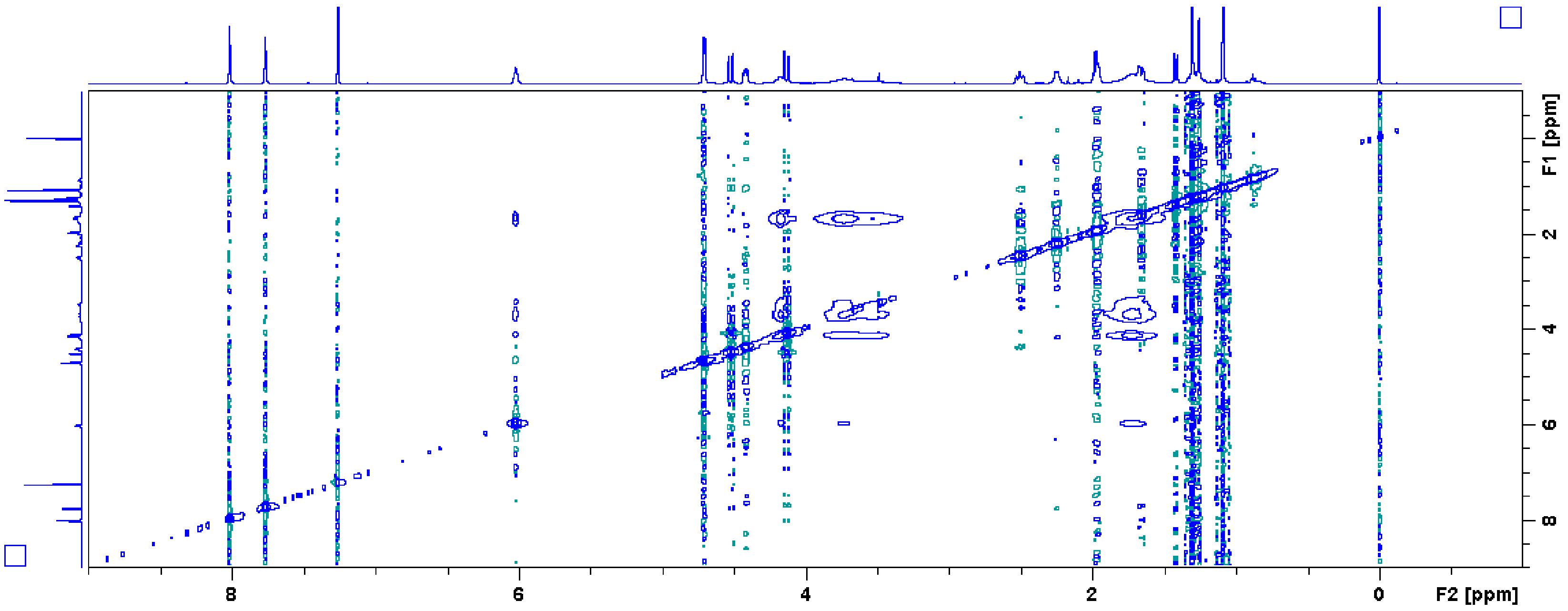

Figure S 44. HSQC-NMR of compound **(–)-22**

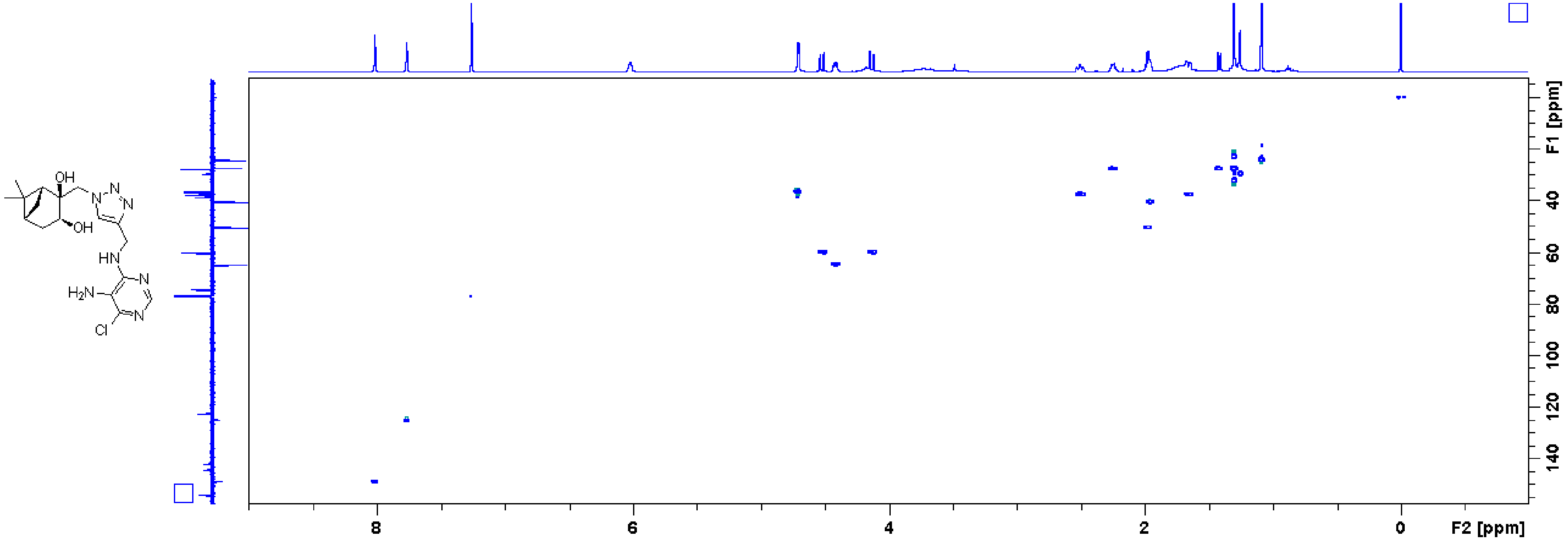

Figure S 45. HMBC-NMR of compound **(–)-22**

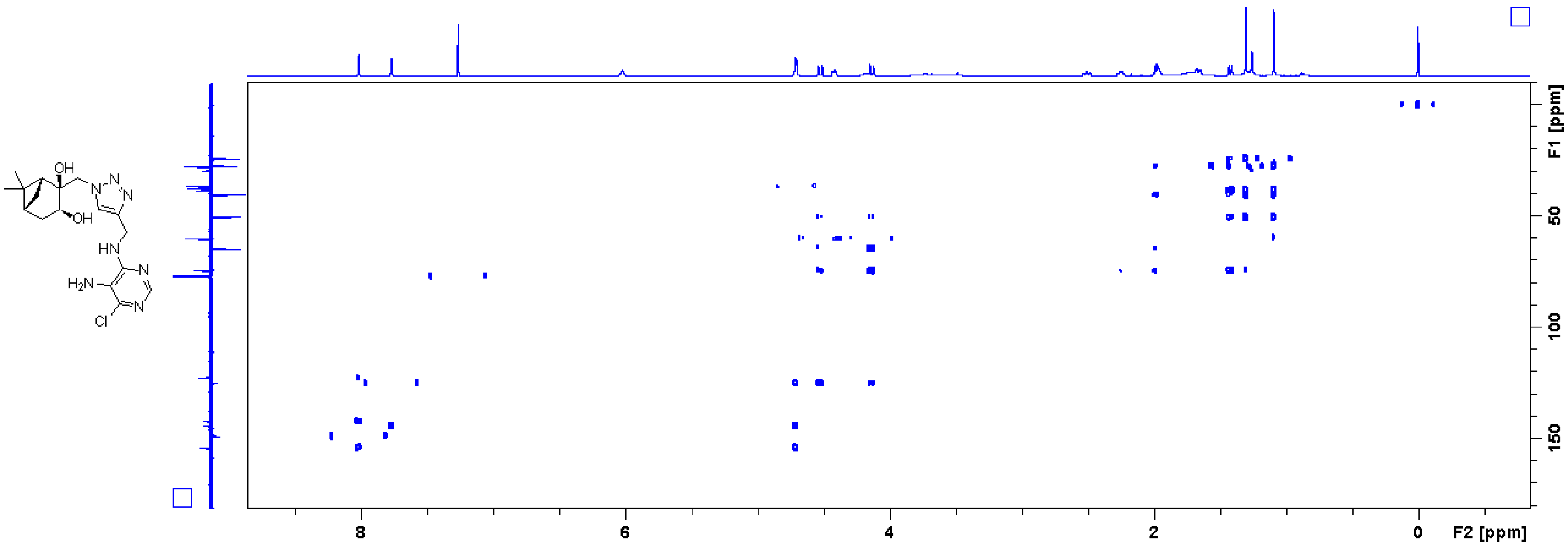

(1*S*,2*R*,3*R*,5*S*)-2-((4-(((5-Fluoro-2-((4-(trifluoromethyl)phenyl)amino)pyrimidin-4-yl)amino)methyl)-1*H*-1,2,3-triazol-1-yl)methyl)-6,6-dimethylbicyclo[3.1.1]heptane-2,3-diol **(+)-23**

Figure S 46. ^{1}H-NMR of compound **(+)-23**

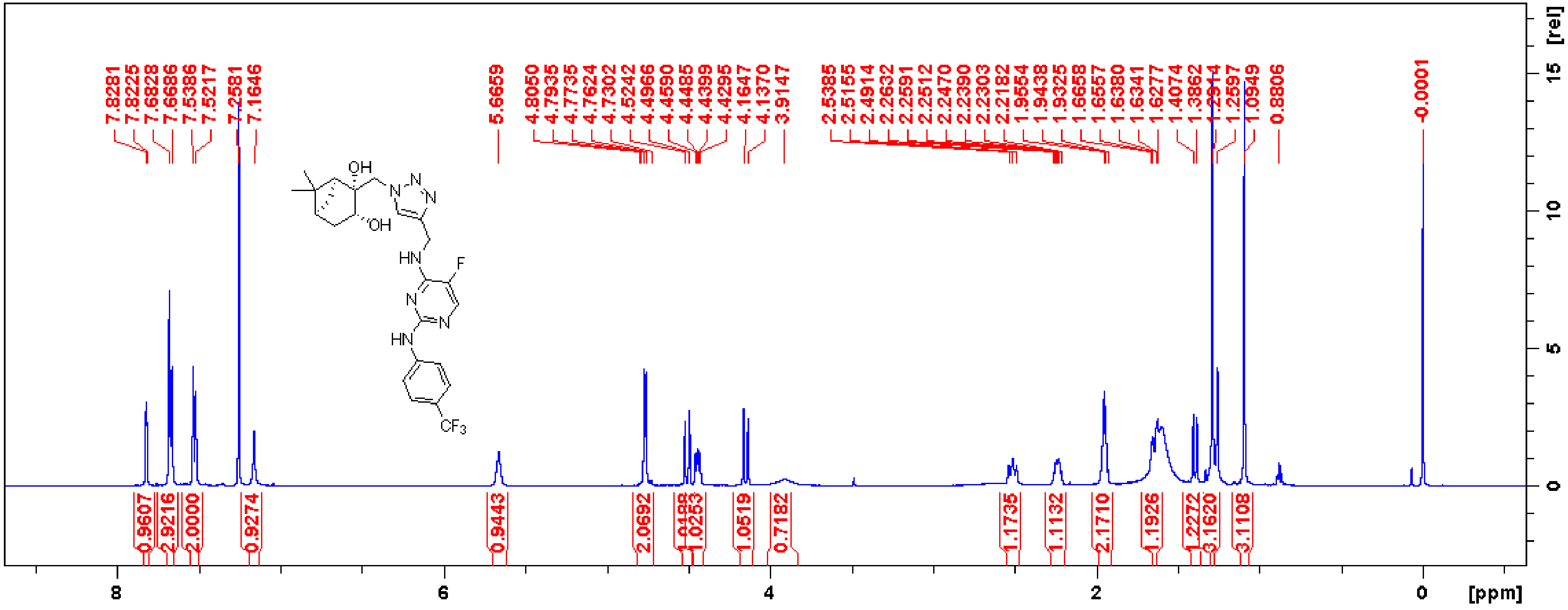

Figure S 47. ^{13}C-NMR of compound **(+)-23**

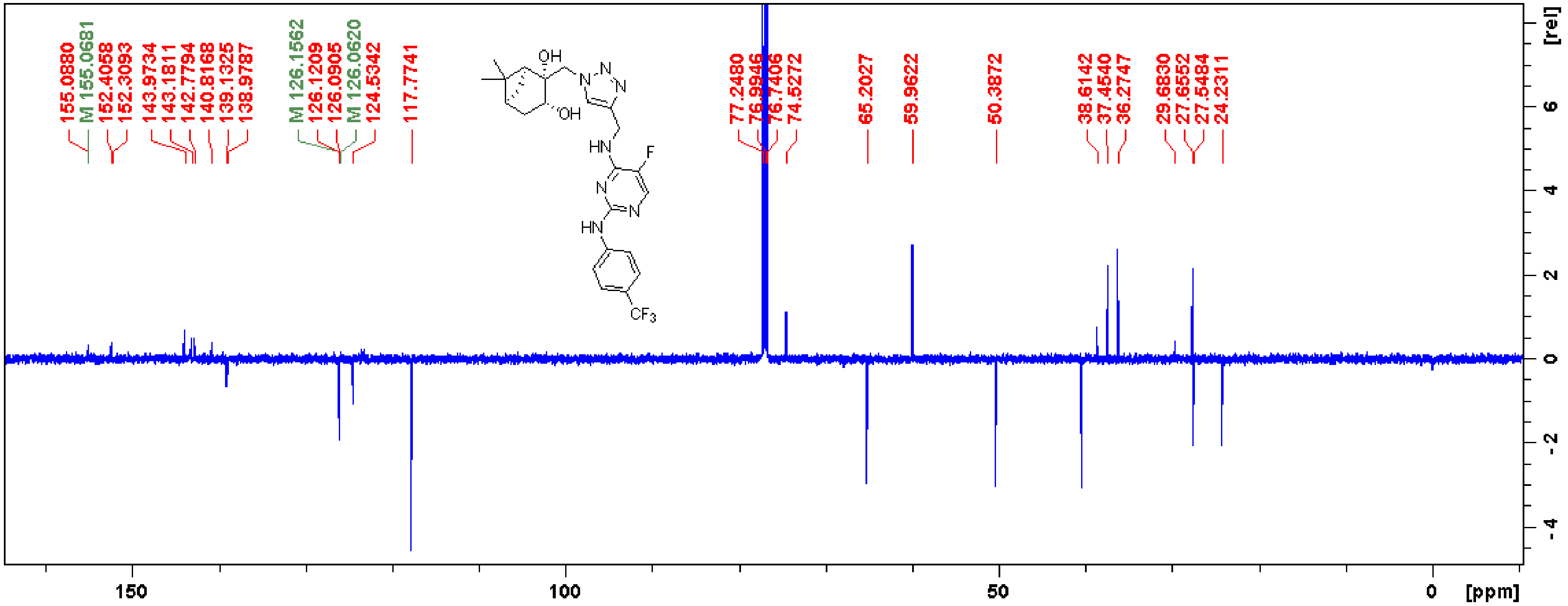

Figure S 48. COSY-NMR of compound **(+)-23**

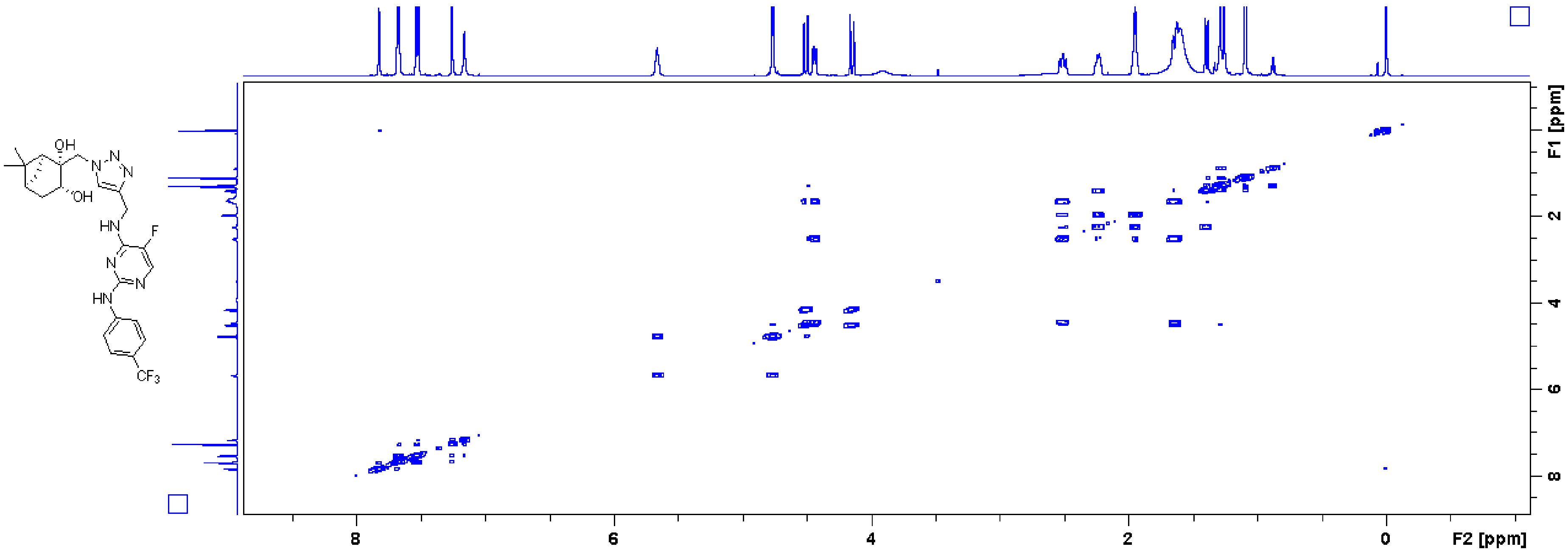

Figure S 49. NOESY-NMR of compound **(+)-23**

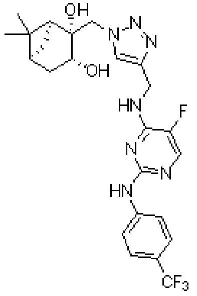

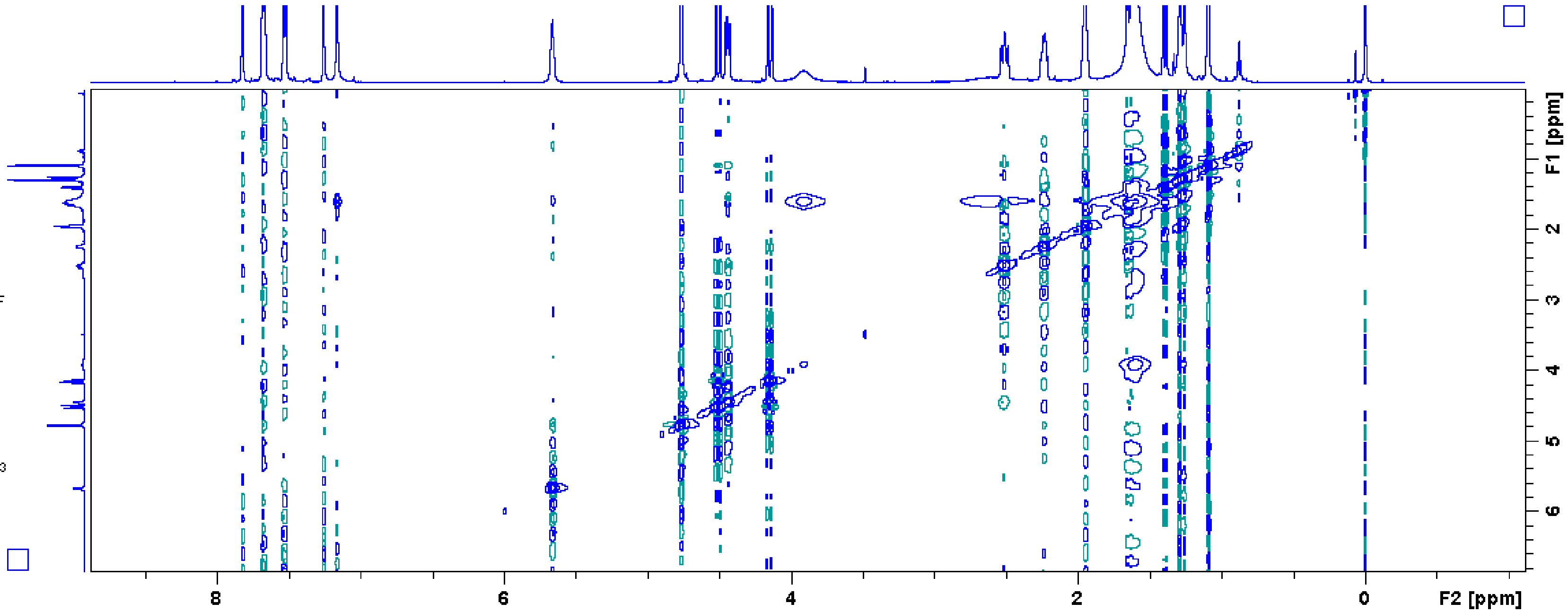

Figure S 50. HSQC-NMR of compound **(+)-23**

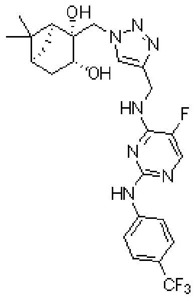

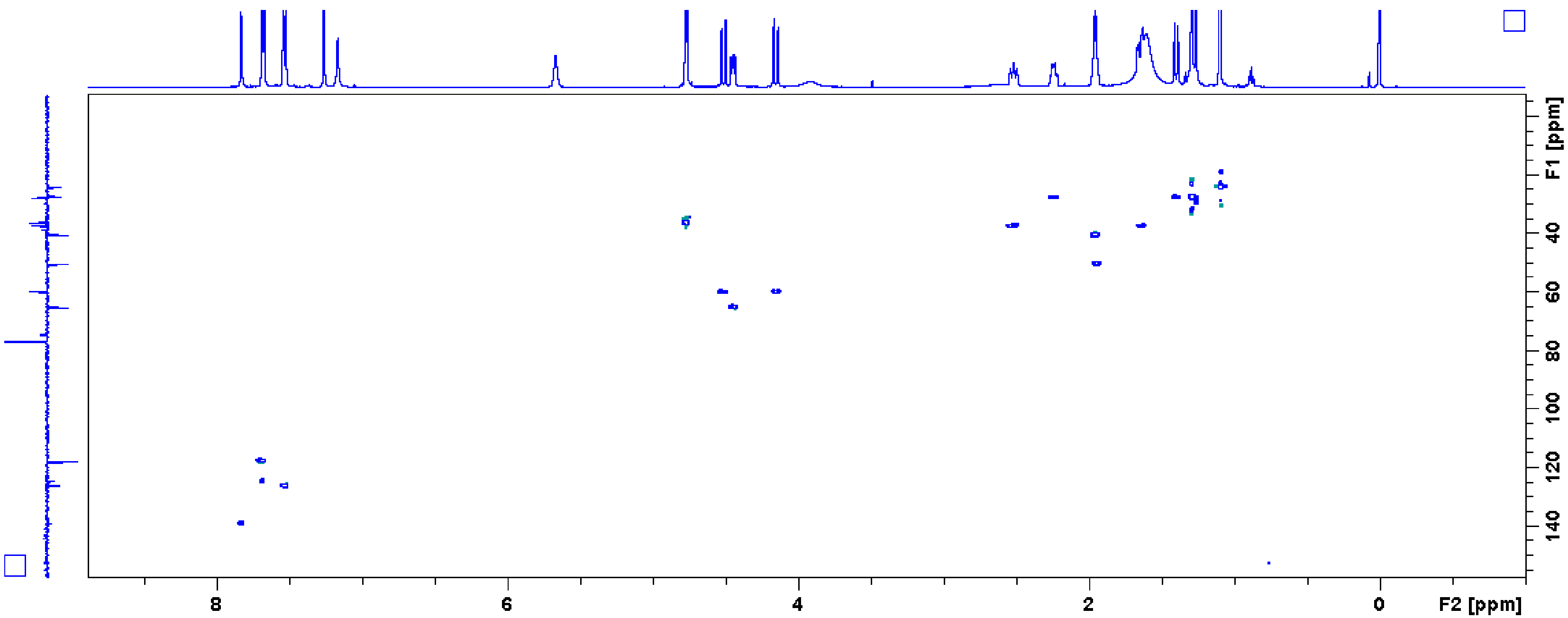

Figure S 51. HMBC-NMR of compound **(+)-23**

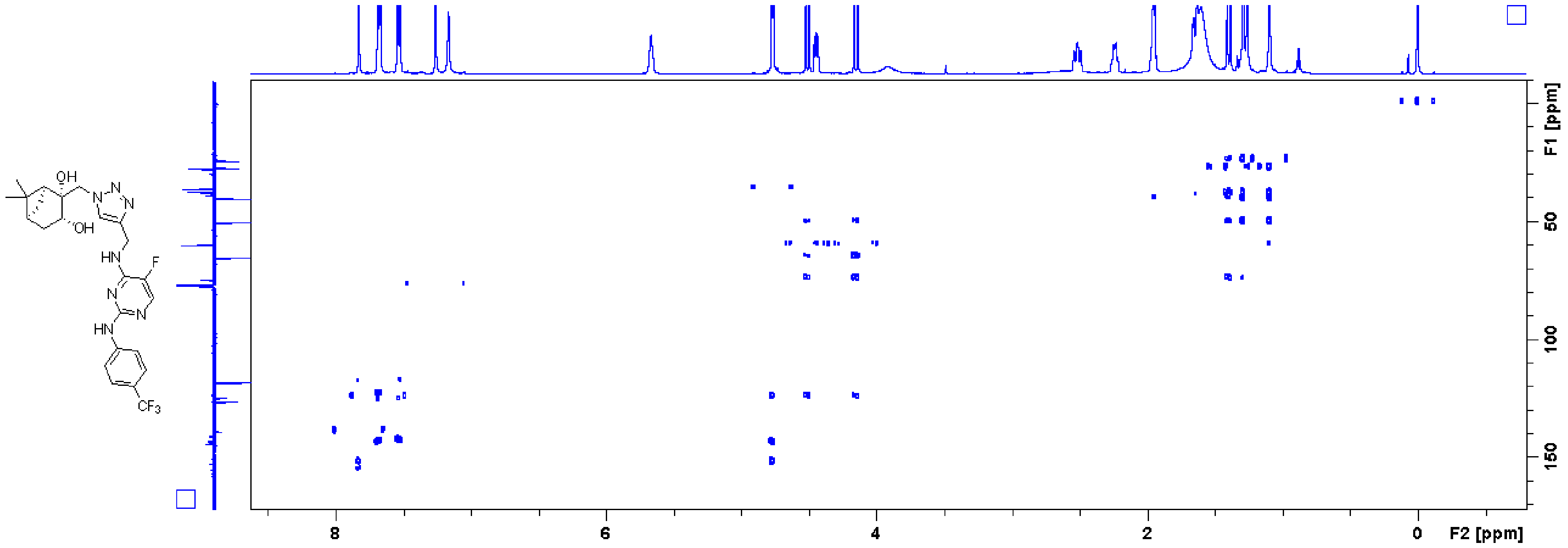

Figure S 52. ^{19}F-NMR of compound **(+)-23**

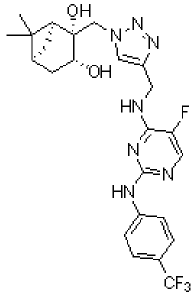

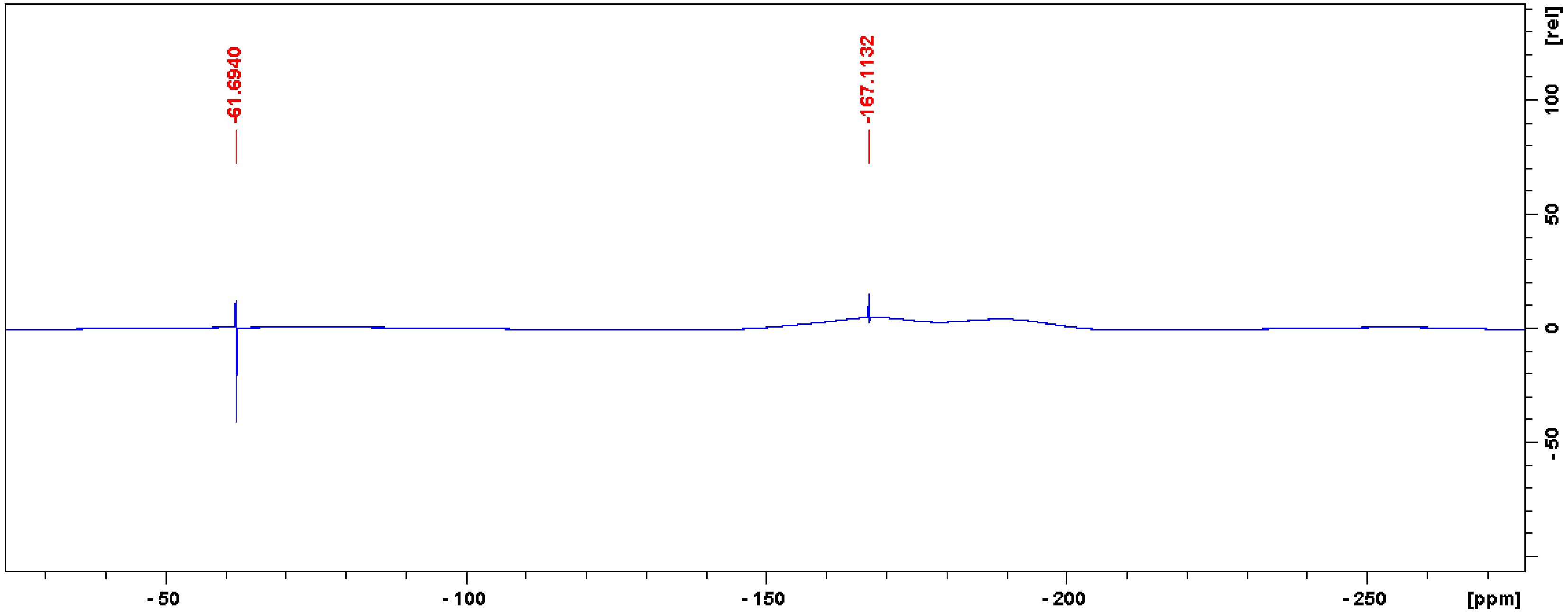

(1*R*,2*S*,3*S*,5*R*)-2-((4-(((5-Fluoro-2-((4-(trifluoromethyl)phenyl)amino)pyrimidin-4-yl)amino)methyl)-1*H*-1,2,3-triazol-1-yl)methyl)-6,6-dimethylbicyclo[3.1.1]heptane-2,3-diol **(–)-23**

Figure S 53. ^{1}H-NMR of compound **(–)-23**

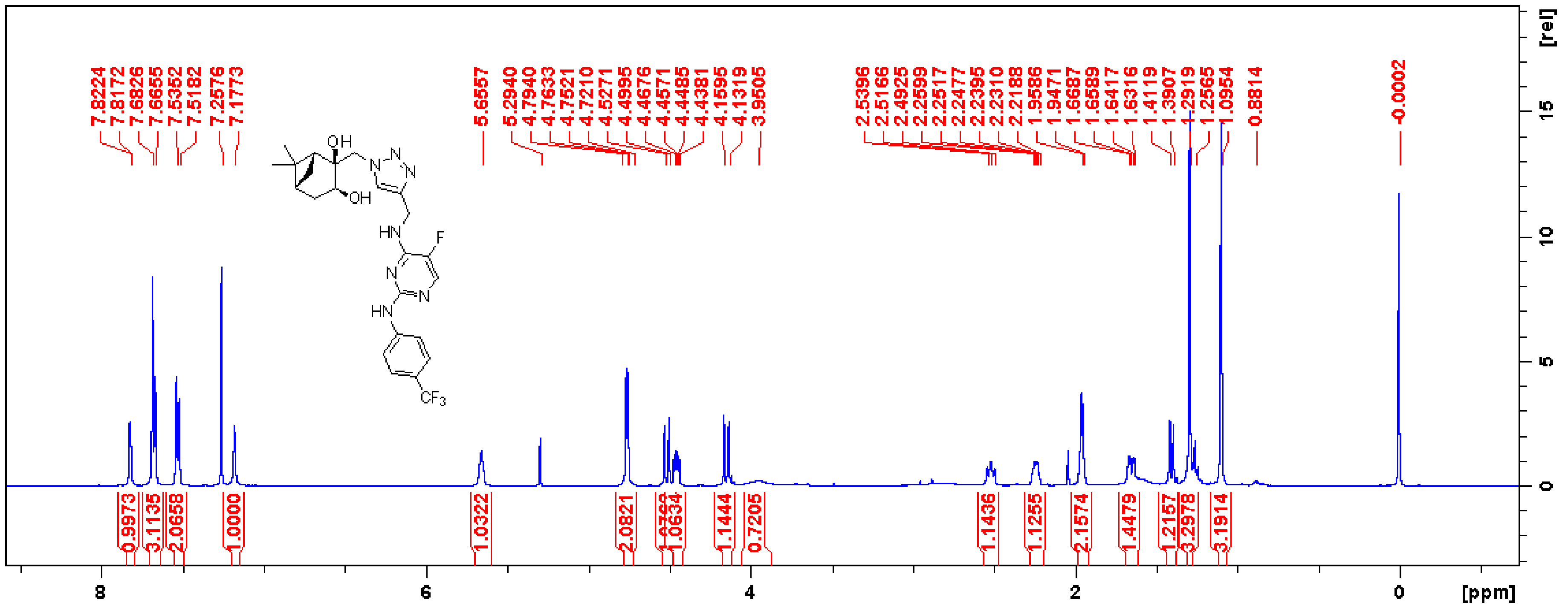

Figure S 54. ^{13}C-NMR of compound **(–)-23**

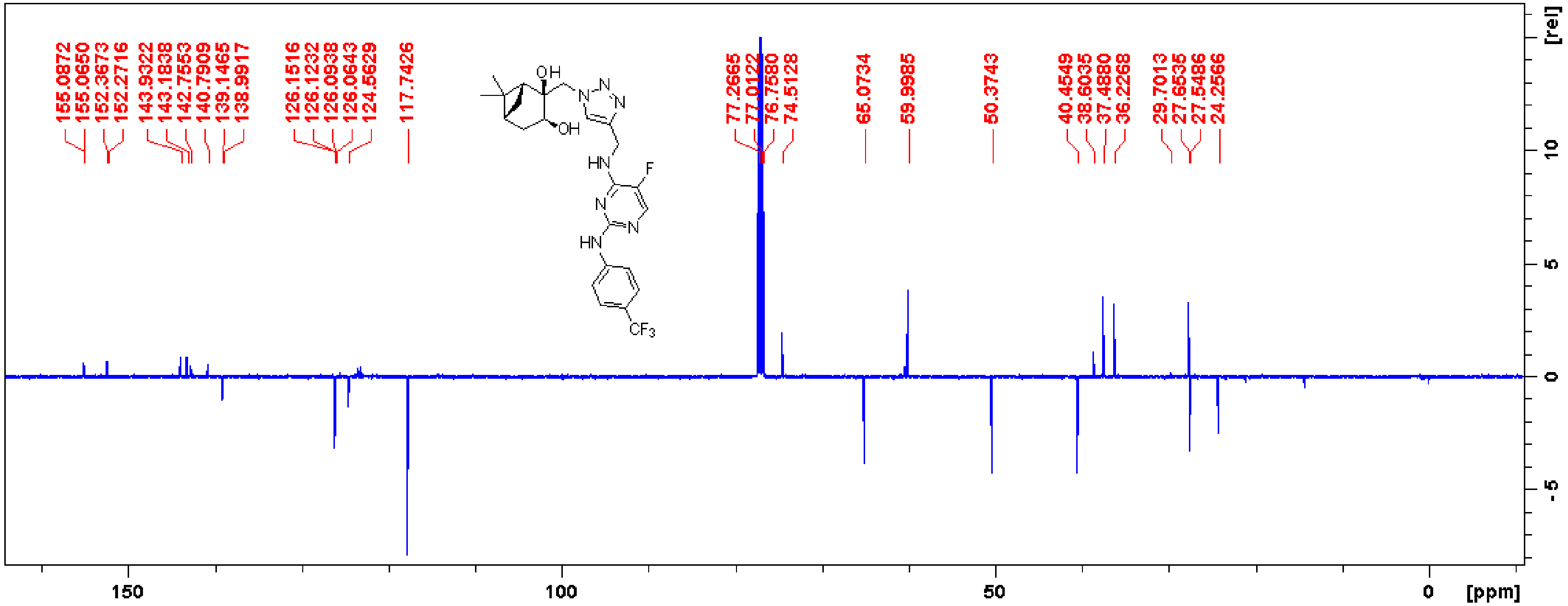

Figure S 55. COSY-NMR of compound **(–)-23**

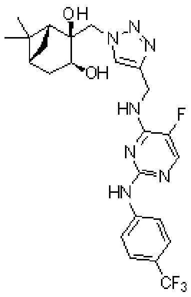

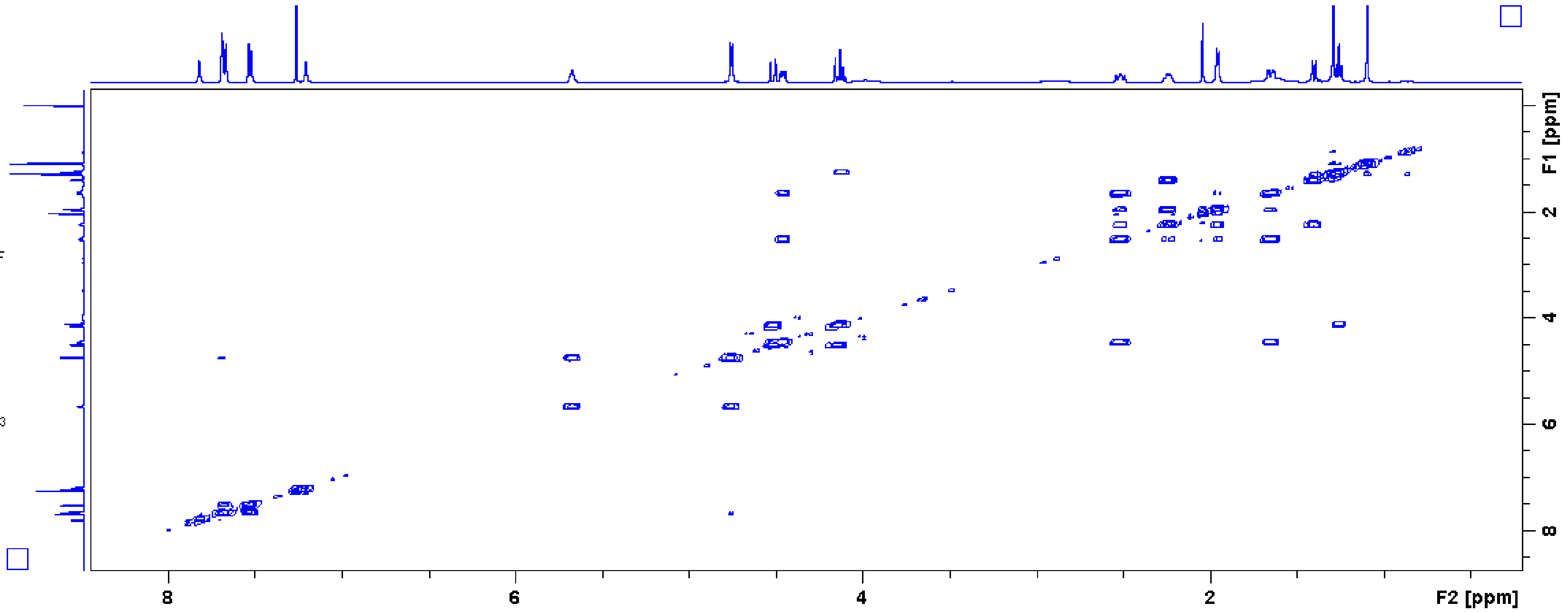

Figure S 56. NOESY-NMR of compound **(–)-23**

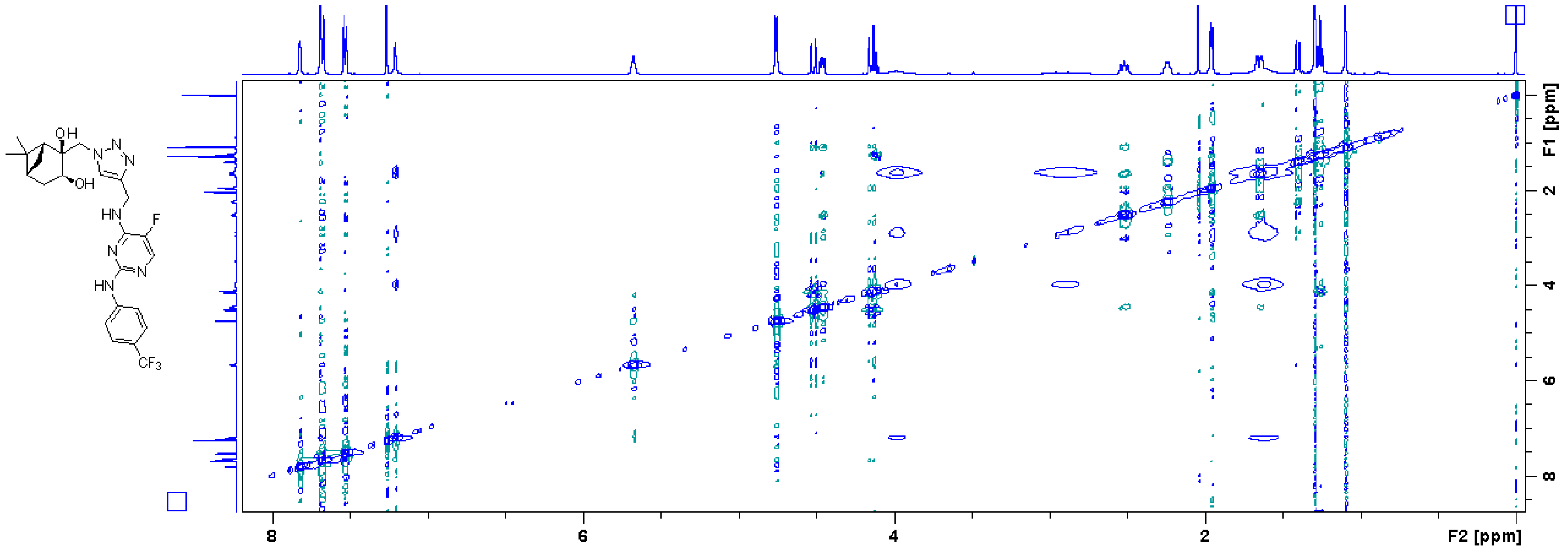

Figure S 57. HSQC-NMR of compound **(–)-23**

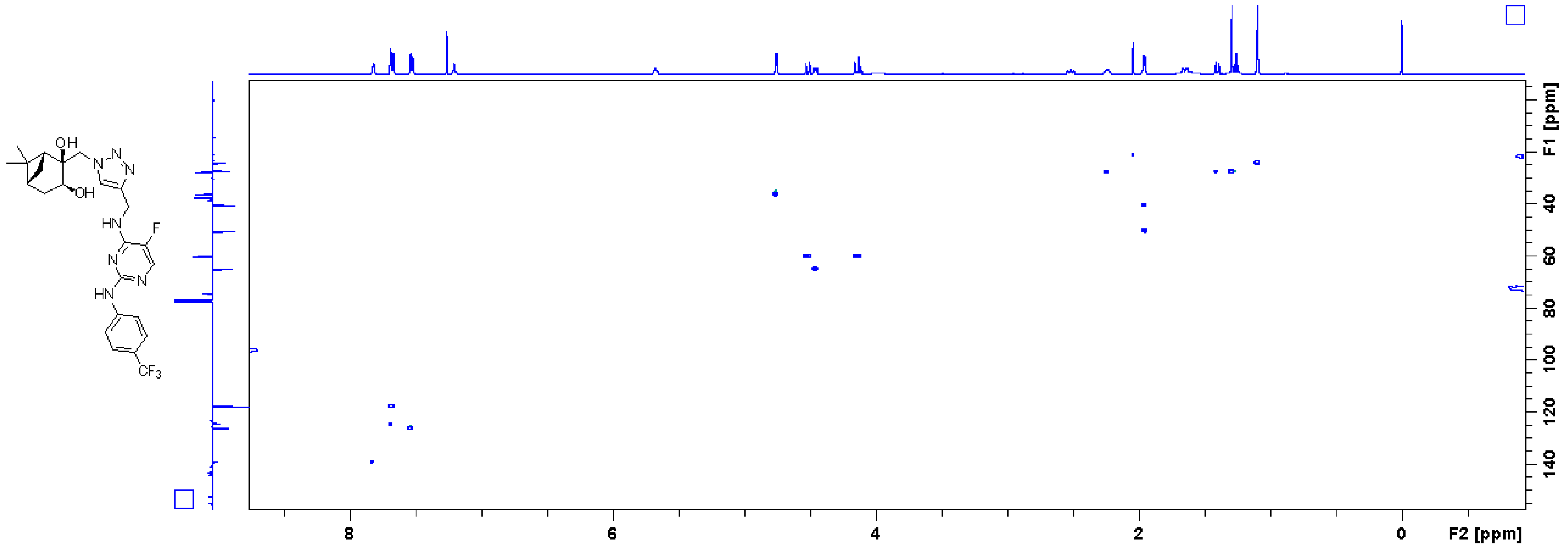

Figure S 58. HMBC-NMR of compound **(–)-23**

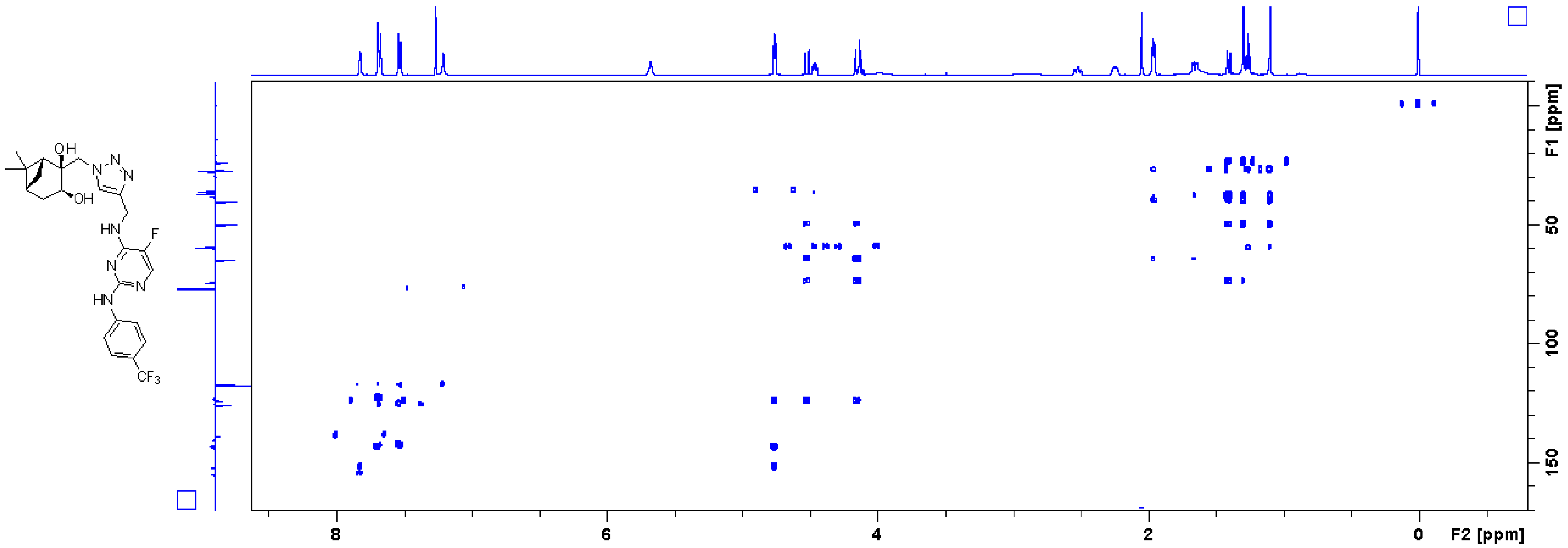

Figure S 59. ^{19}F-NMR of compound **(–)-23**

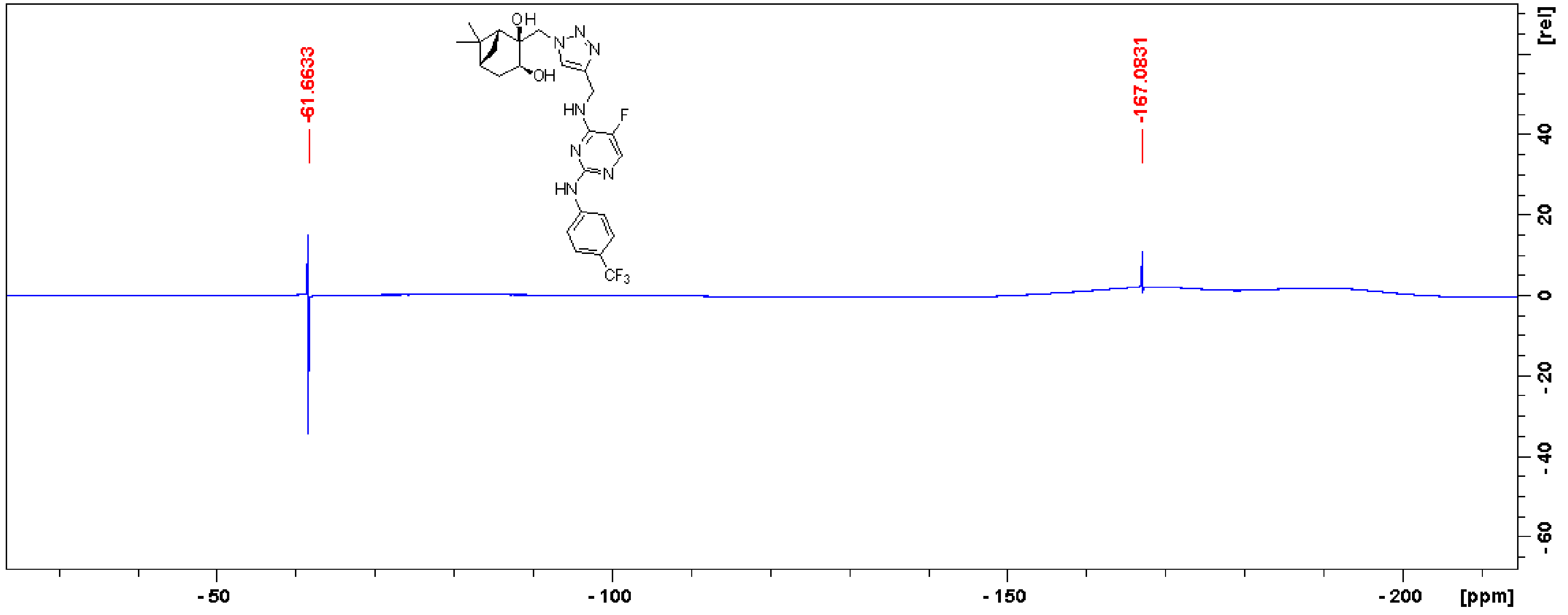

(1*S*,2*R*,3*R*,5*S*)-2-((4-(((5-Chloro-2-((4-(trifluoromethyl)phenyl)amino)pyrimidin-4-yl)amino)methyl)-1*H*-1,2,3-triazol-1-yl)methyl)-6,6-dimethylbicyclo[3.1.1]heptane-2,3-diol **(+)-24**

Figure S 60. ^{1}H-NMR of compound **(+)-24**

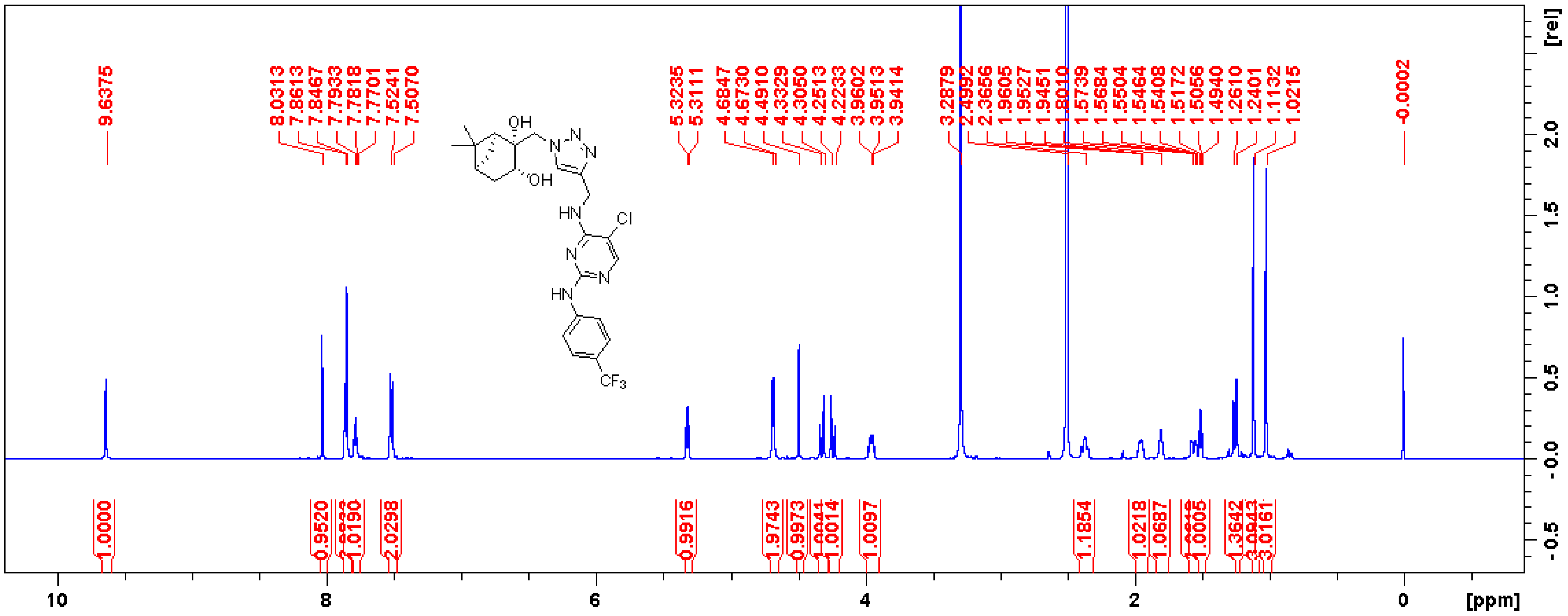

Figure S 61. ^{13}C-NMR of compound **(+)-24**

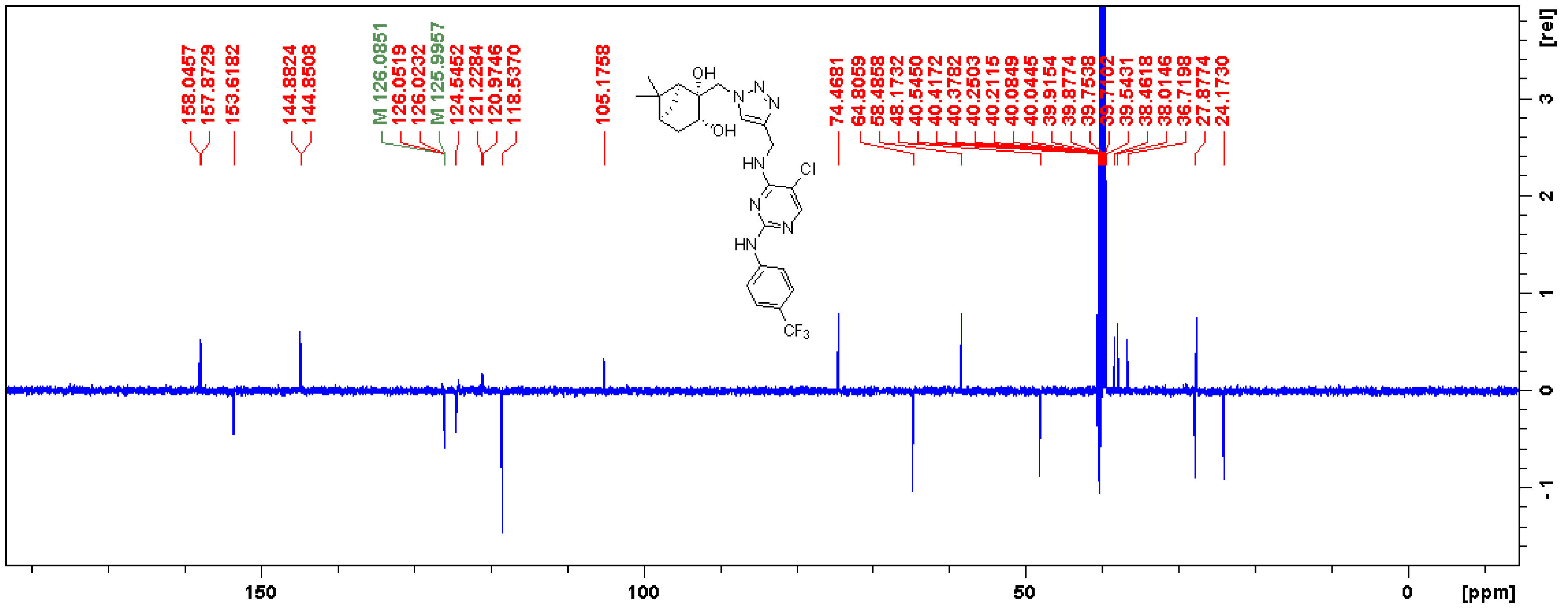

Figure S 62. COSY-NMR of compound **(+)-24**

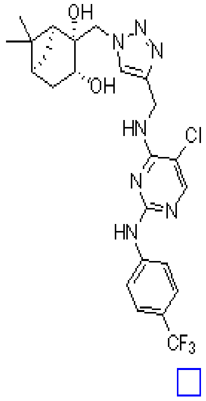

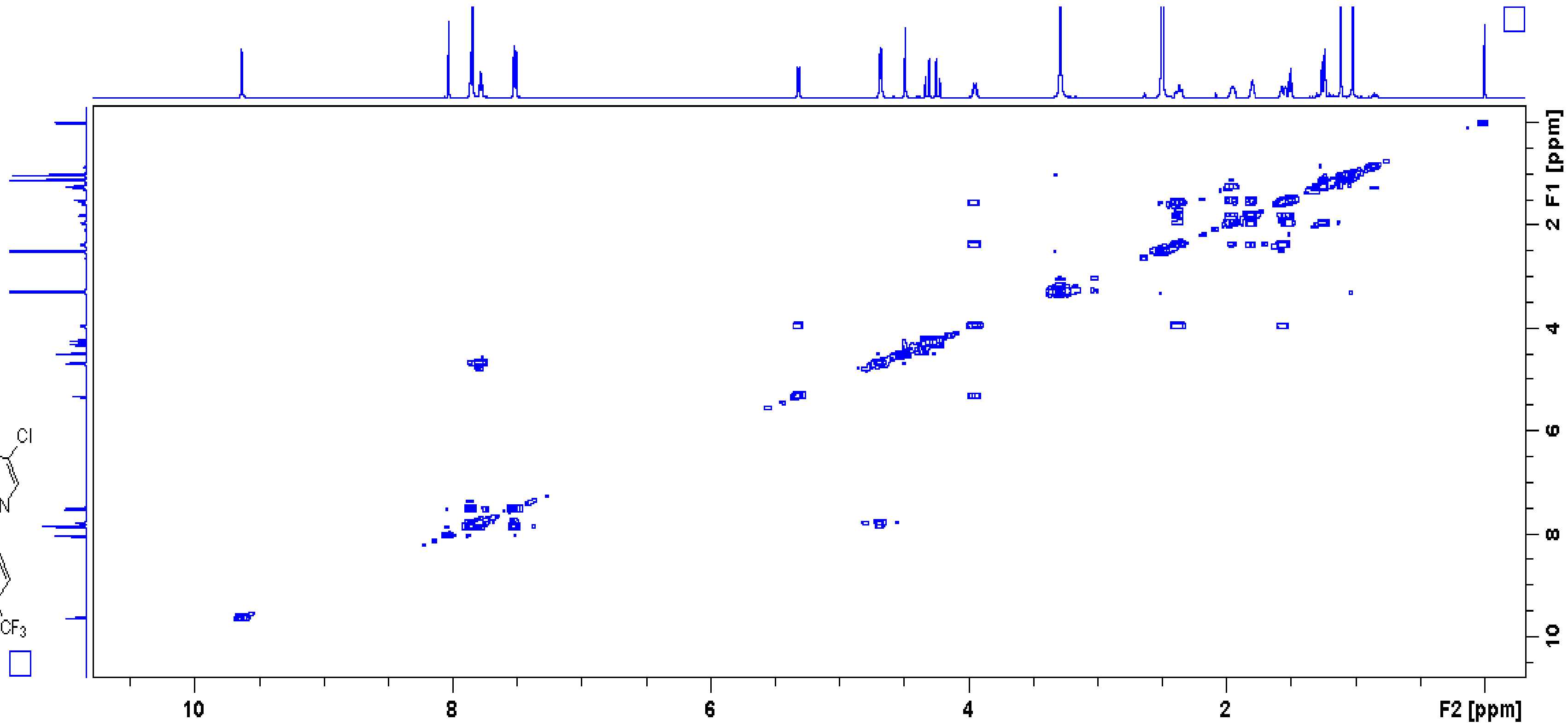

Figure S 63. NOESY-NMR of compound **(+)-24**

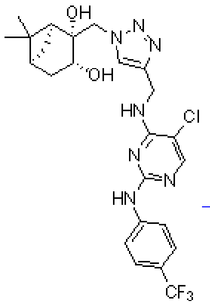

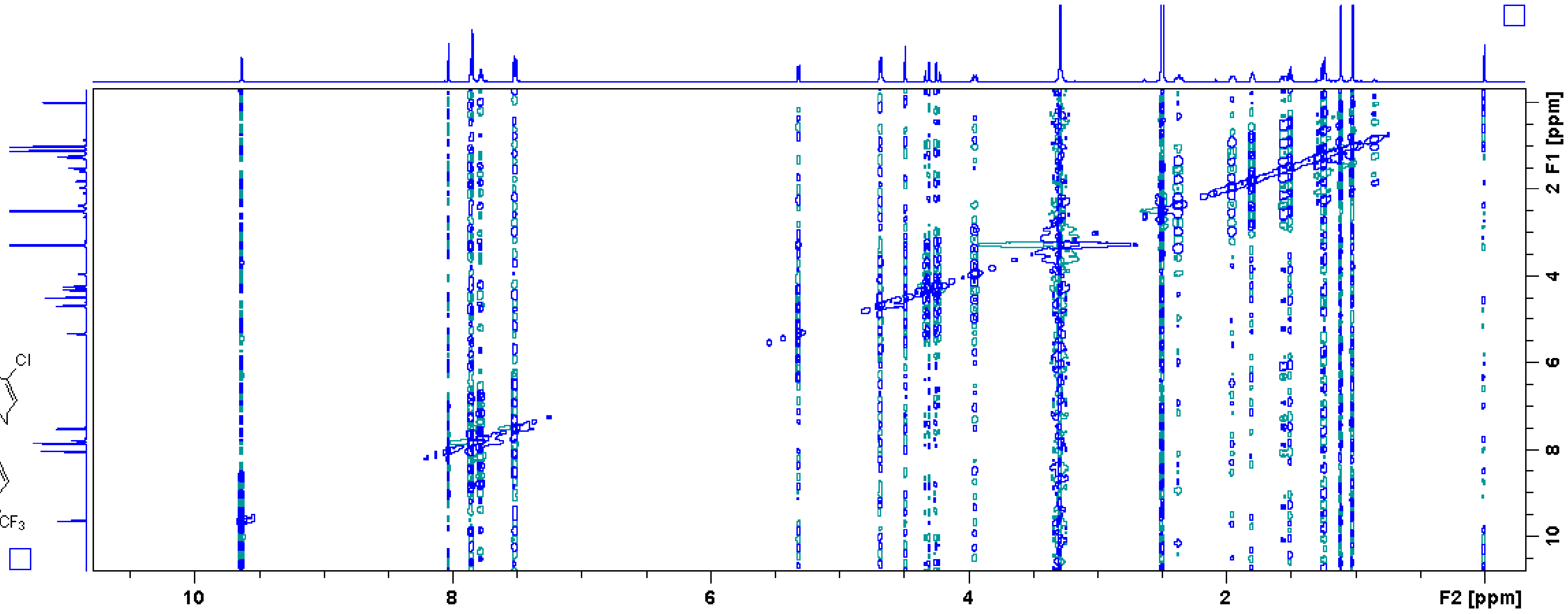

Figure S 64. HSQC-NMR of compound **(+)-24**

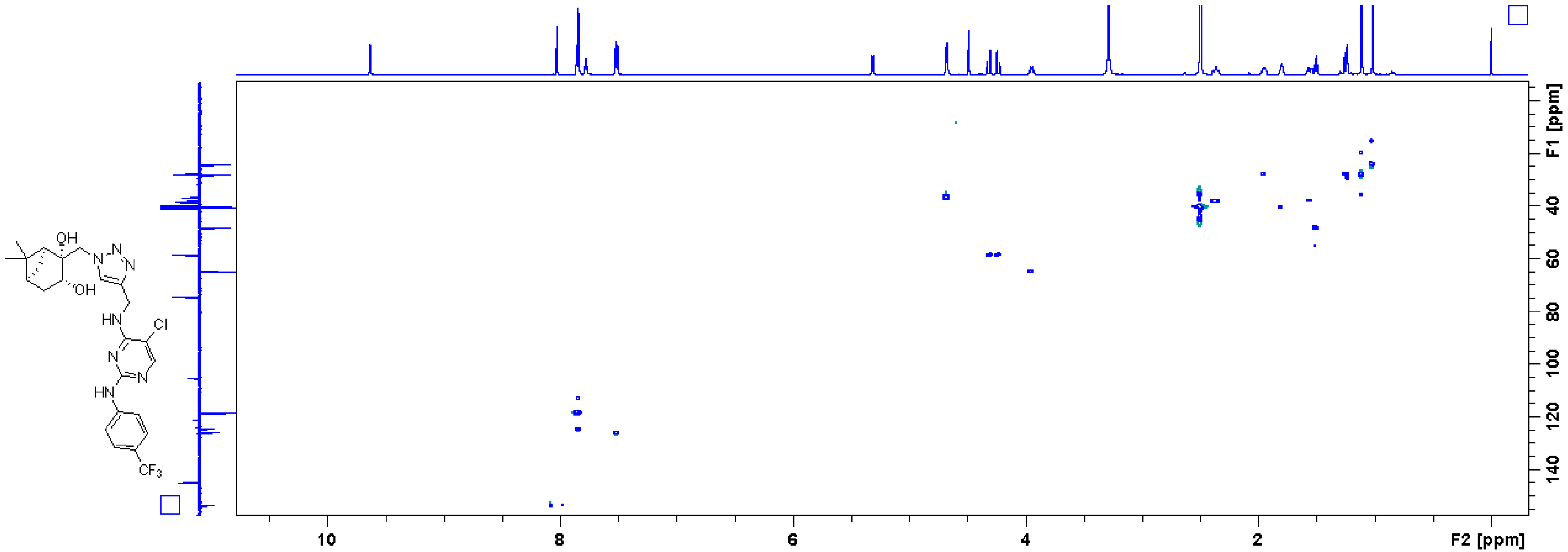

Figure S 65. HMBC-NMR of compound **(+)-24**

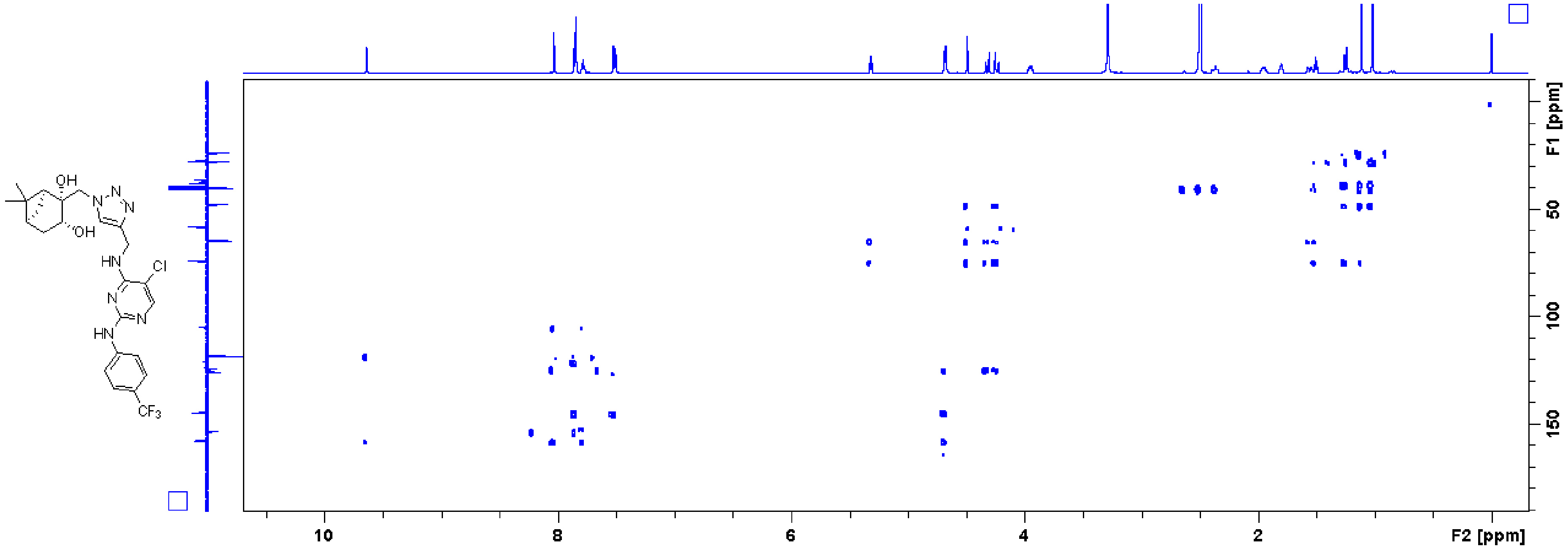

Figure S 66. ^{19}F-NMR of compound **(+)-24**

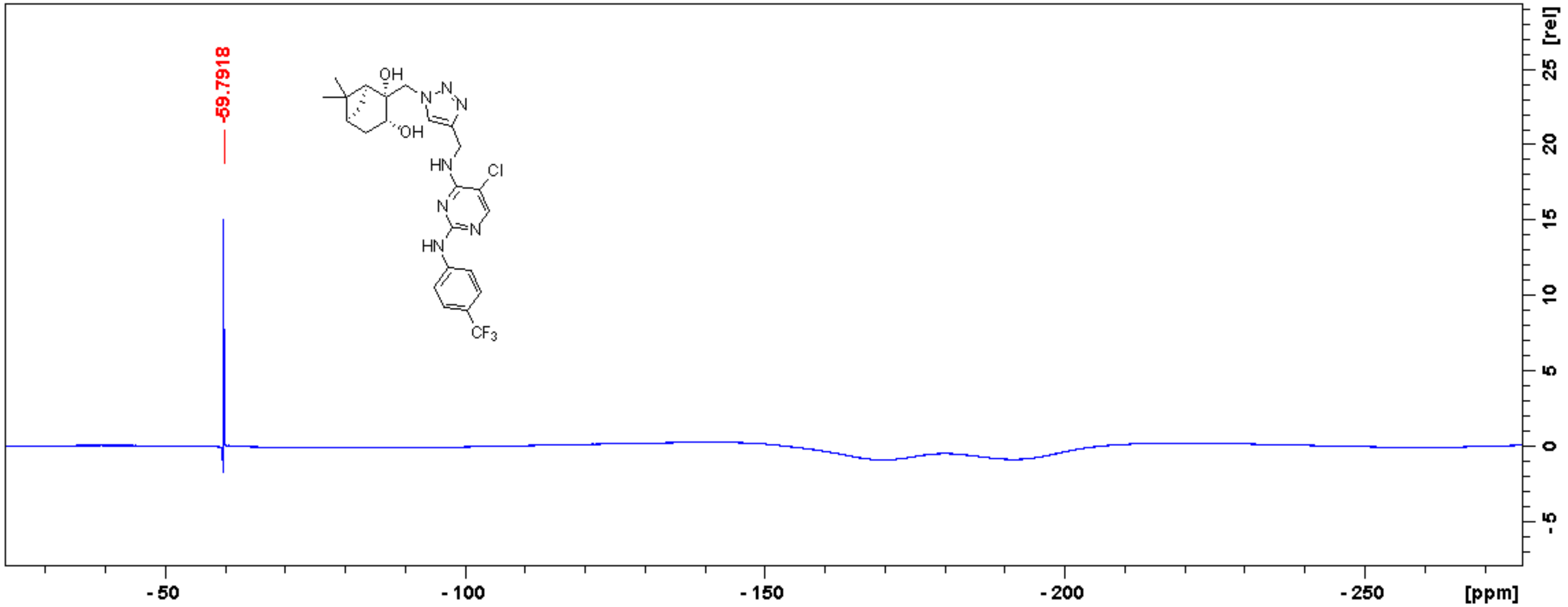

(1*R*,2*S*,3*S*,5*R*)-2-((4-(((5-chloro-2-((4-(trifluoromethyl)phenyl)amino)pyrimidin-4-yl)amino)methyl)-1H-1,2,3-triazol-1-yl)methyl)-6,6-dimethylbicyclo[3.1.1]heptane-2,3-diol **(–)-24**

Figure S 67. ^{1}H-NMR of compound **(–)-24**

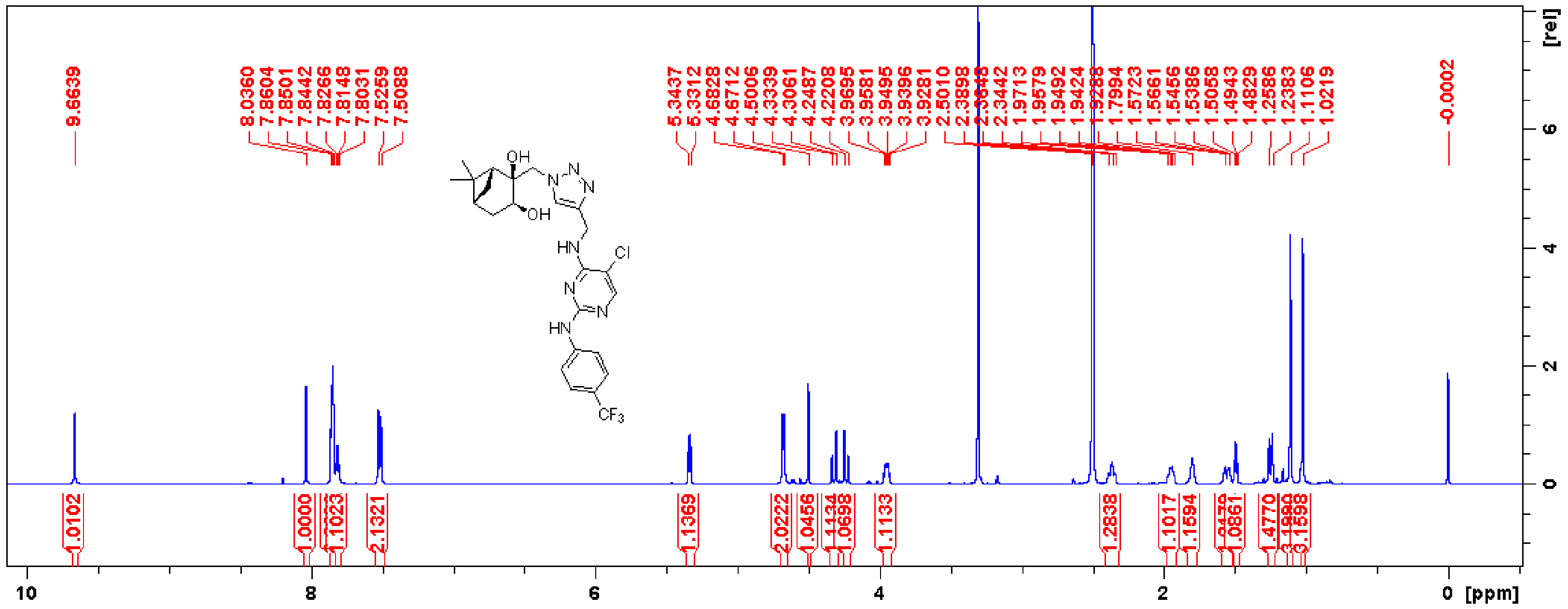

Figure S 68. ^{13}C-NMR of compound **(–)-24**

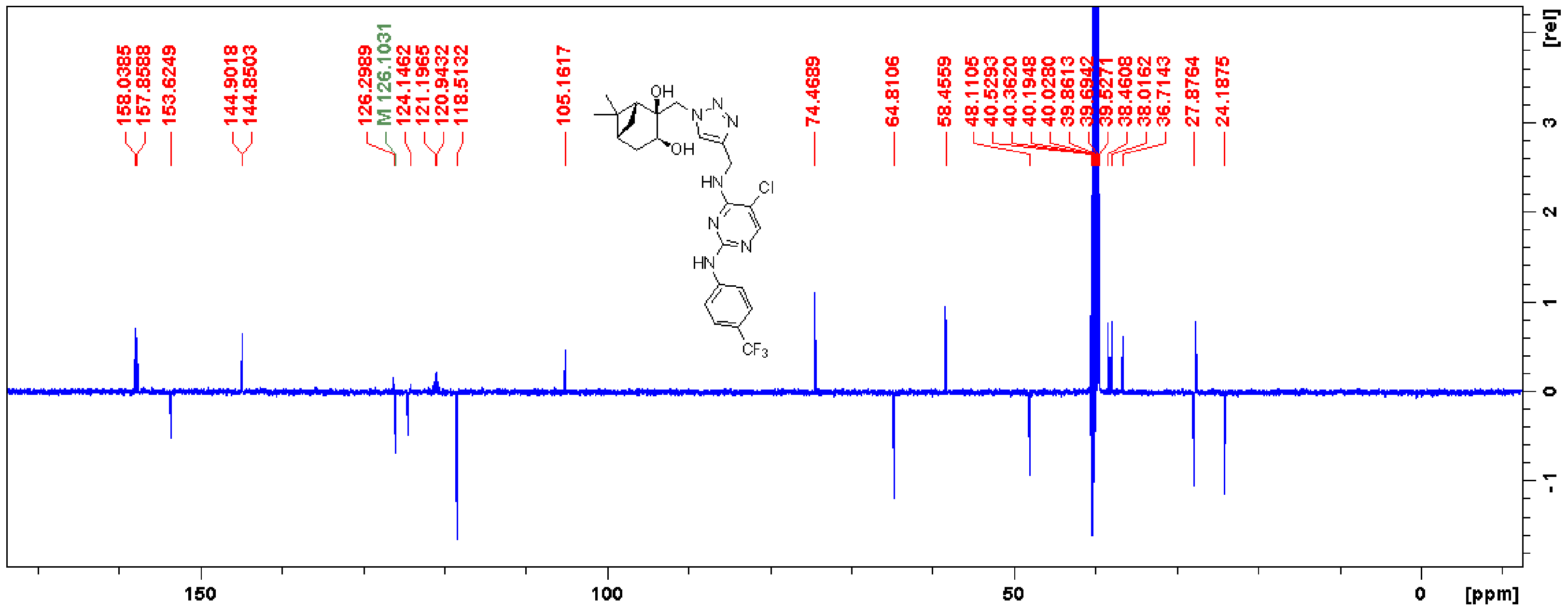

Figure S 69. COSY-NMR of compound **(–)-24**

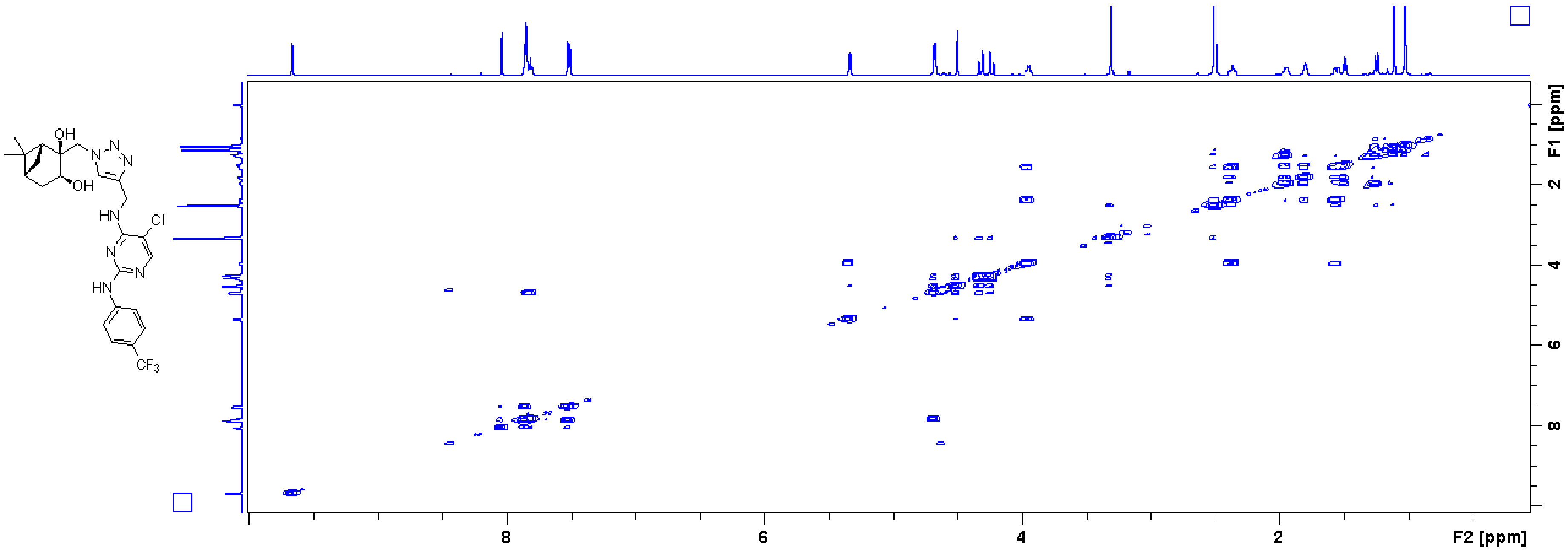

Figure S 70. NOESY-NMR of compound **(–)-24**

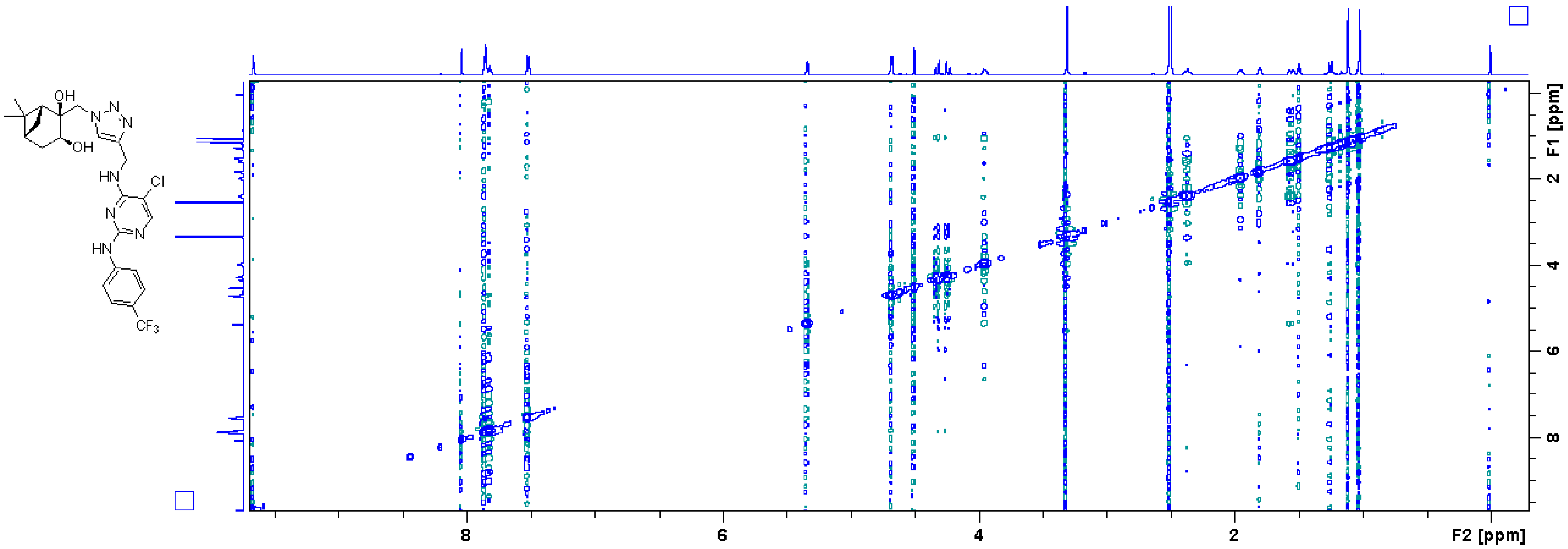

Figure S 71. HSQC-NMR of compound **(–)-24**

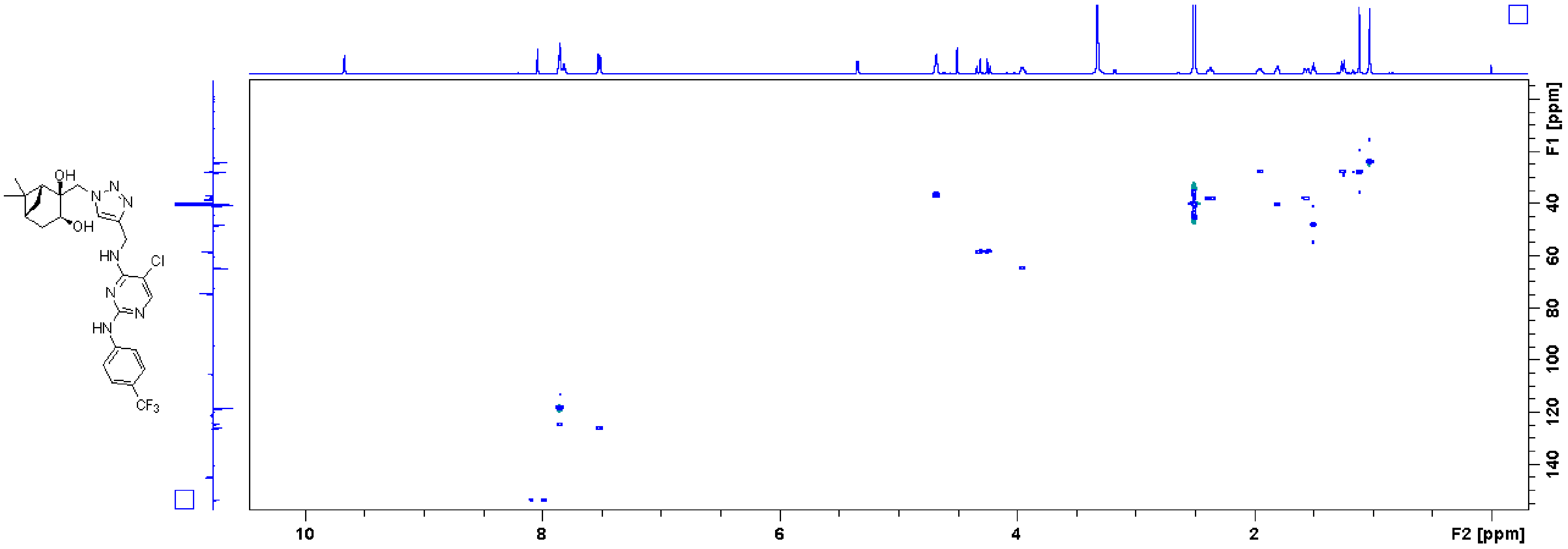

Figure S 72. HMBC-NMR of compound **(–)-24**

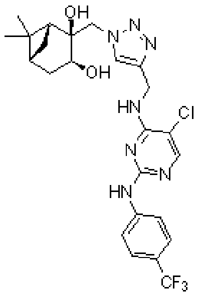

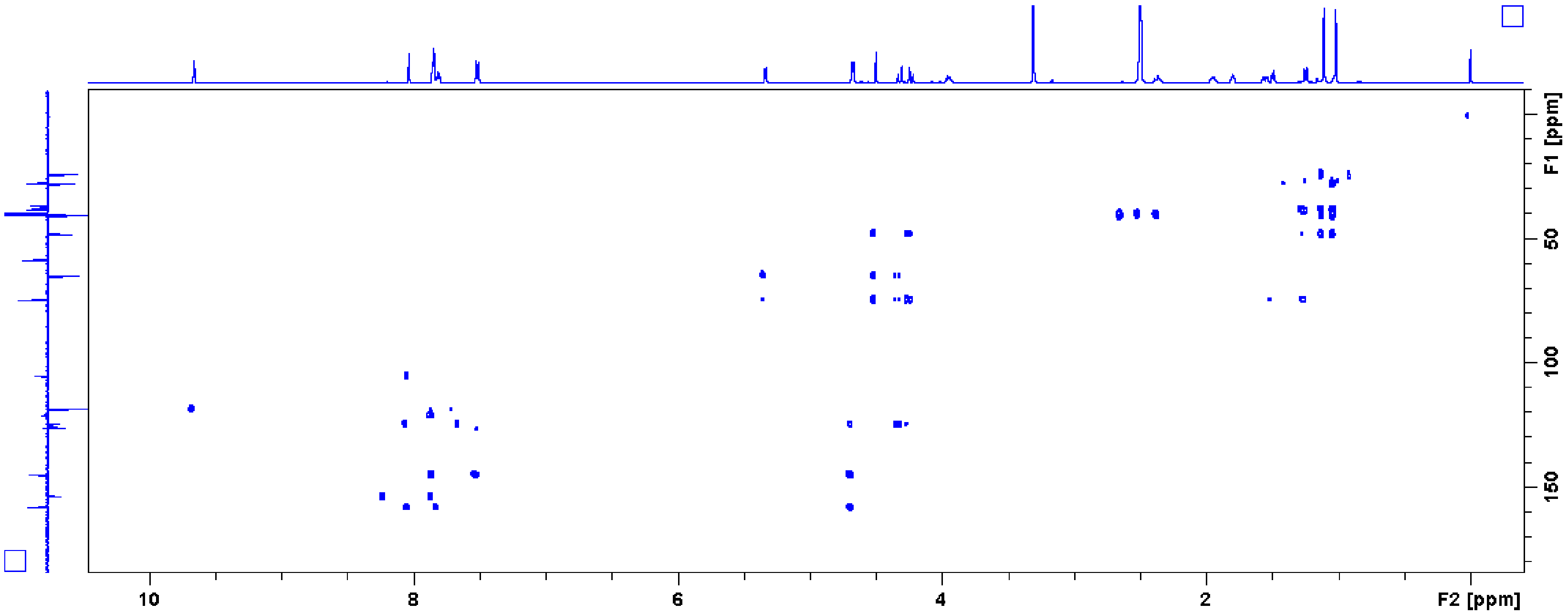

Figure S 73. ^{19}F-NMR of compound **(–)-24**

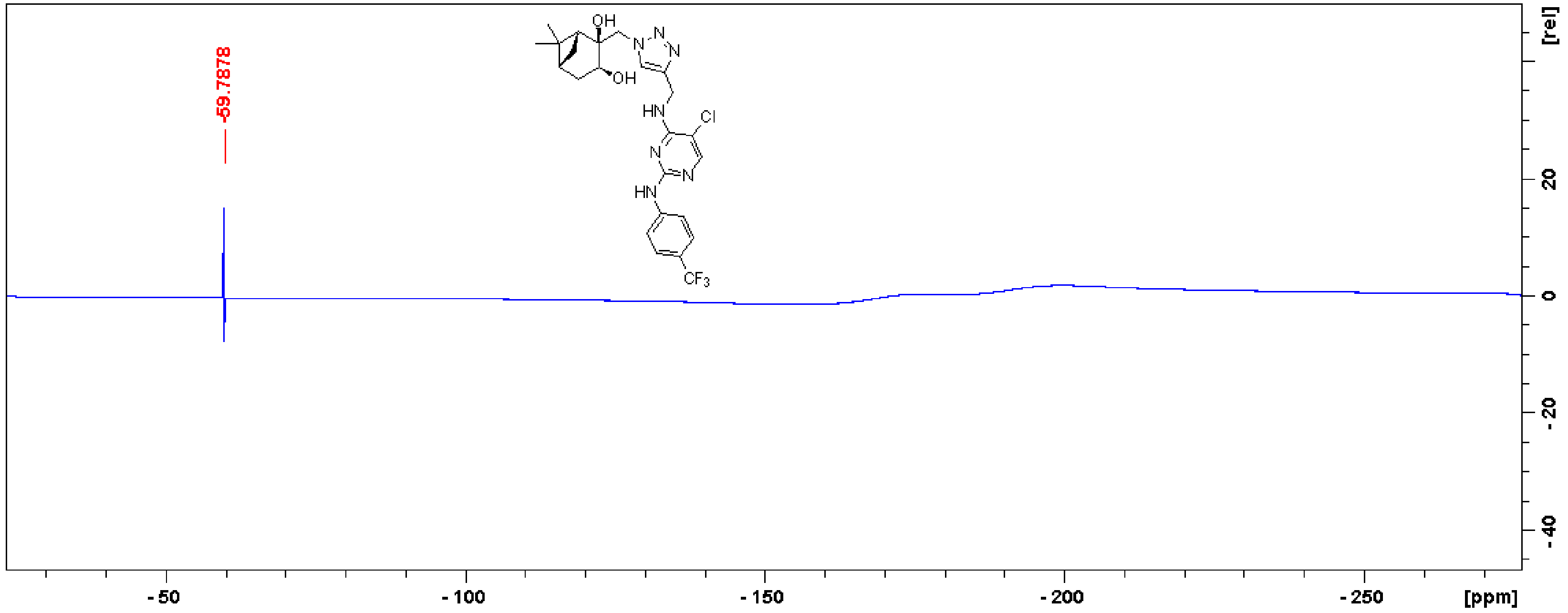

(1*S*,2*R*,3*R*,5*S*)-2-((4-(((5-Fluoro-2-((1-methyl-1H-pyrazol-4-yl)amino)pyrimidin-4-yl)amino)methyl)-1*H*-1,2,3-triazol-1-yl)methyl)-6,6-dimethylbicyclo[3.1.1]heptane-2,3-diol **(+)-25**

Figure S 74. ^{1}H-NMR of compound **(+)-25**

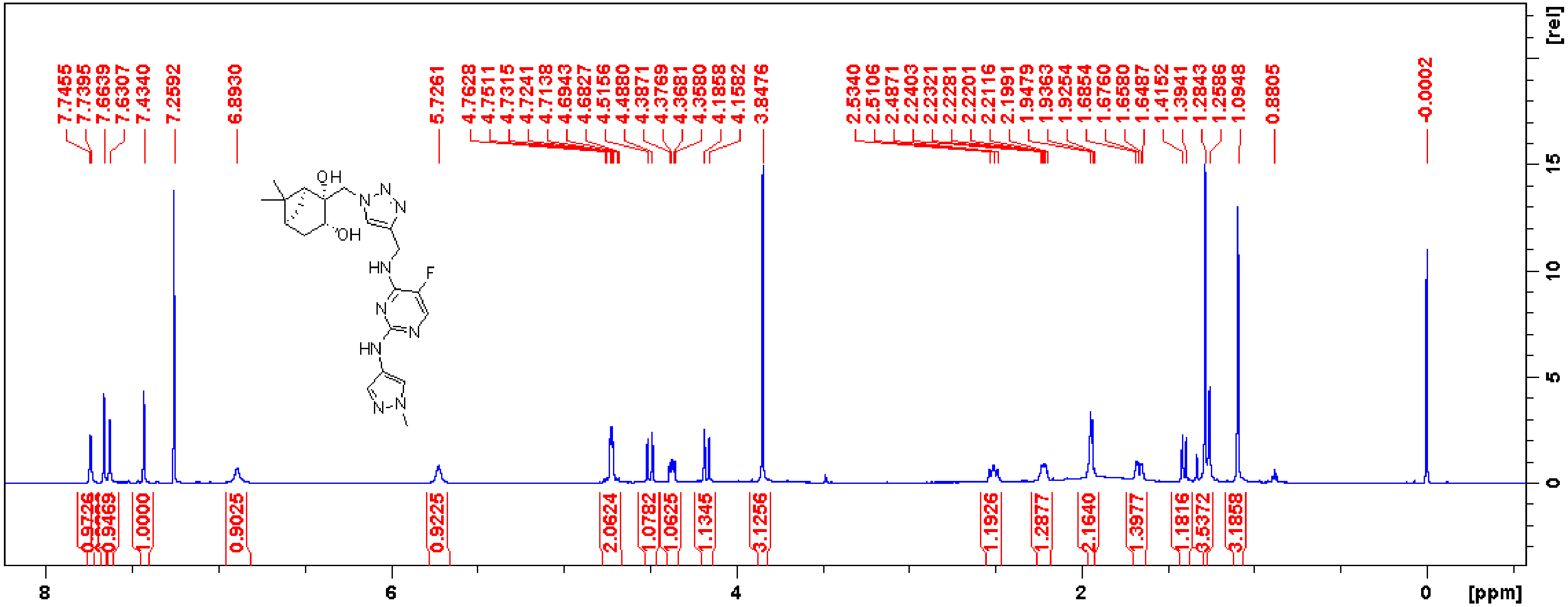

Figure S 75. ^{13}C-NMR of compound **(+)-25**

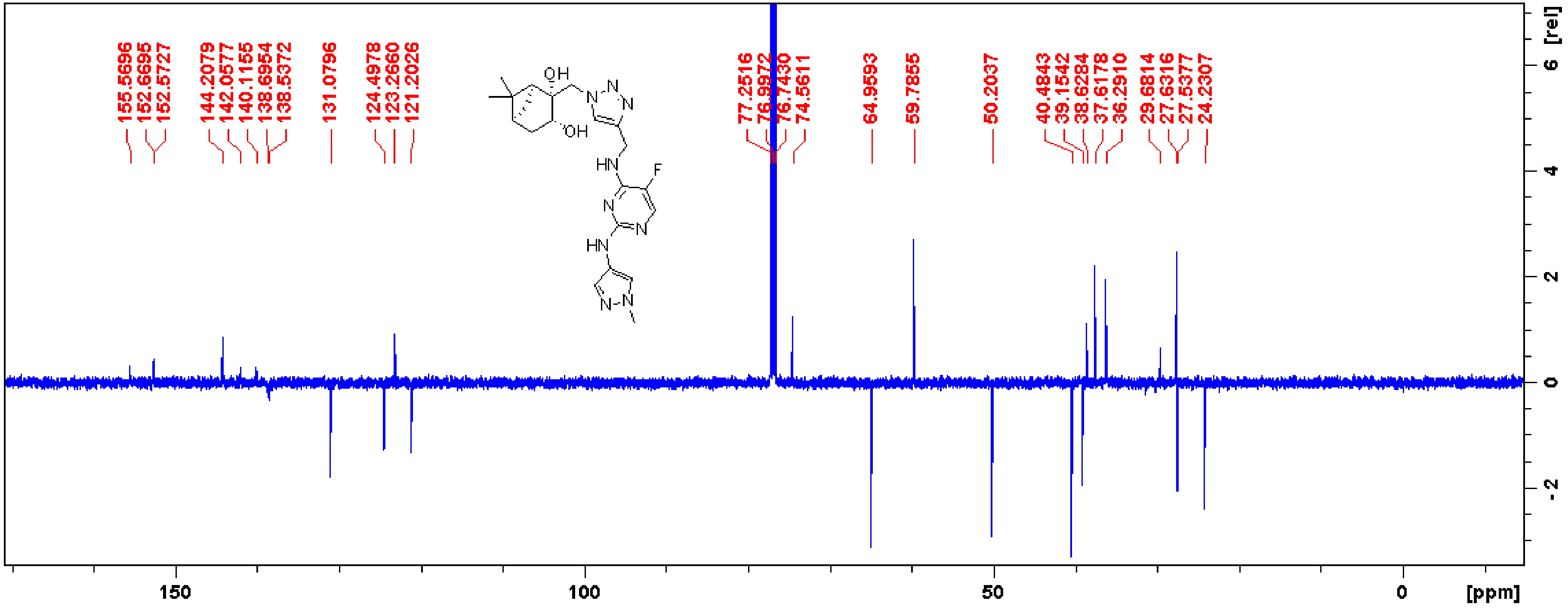

Figure S 76. COSY-NMR of compound **(+)-25**

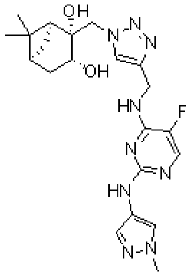

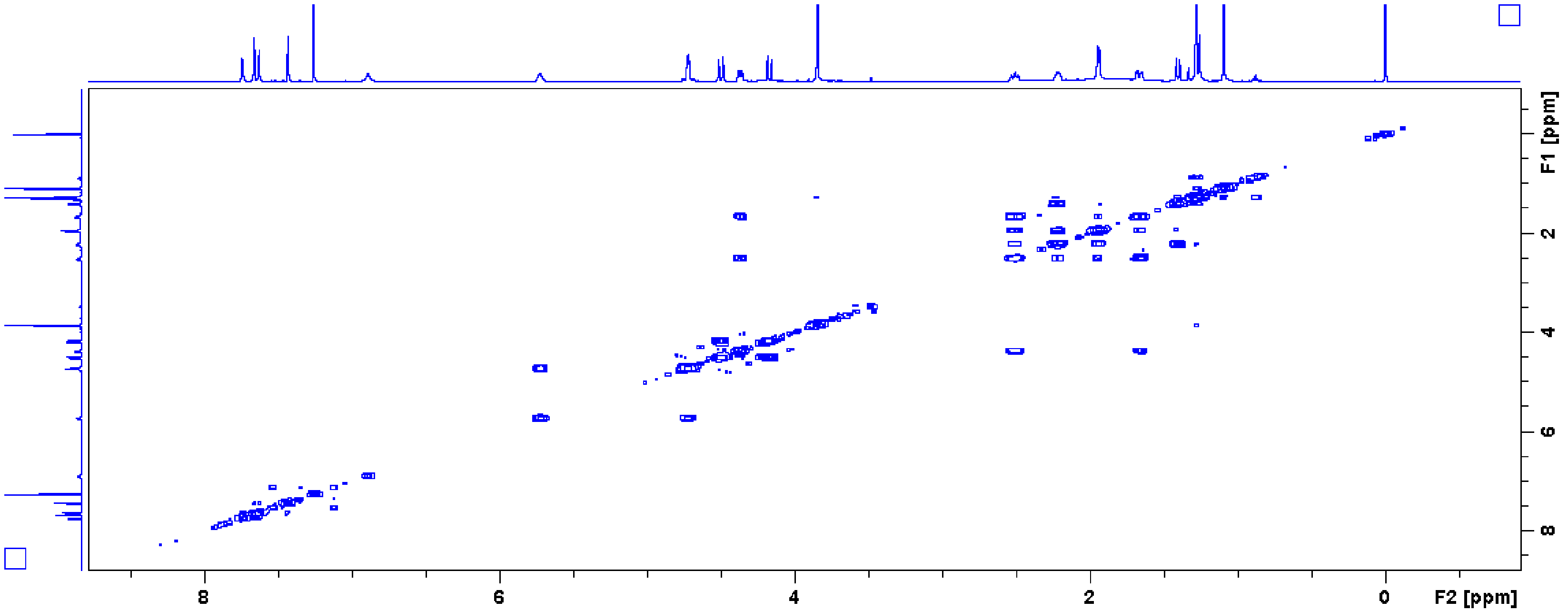

Figure S 77. NOESY-NMR of compound **(+)-25**

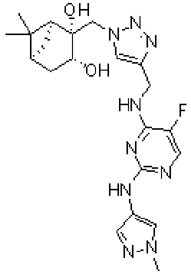

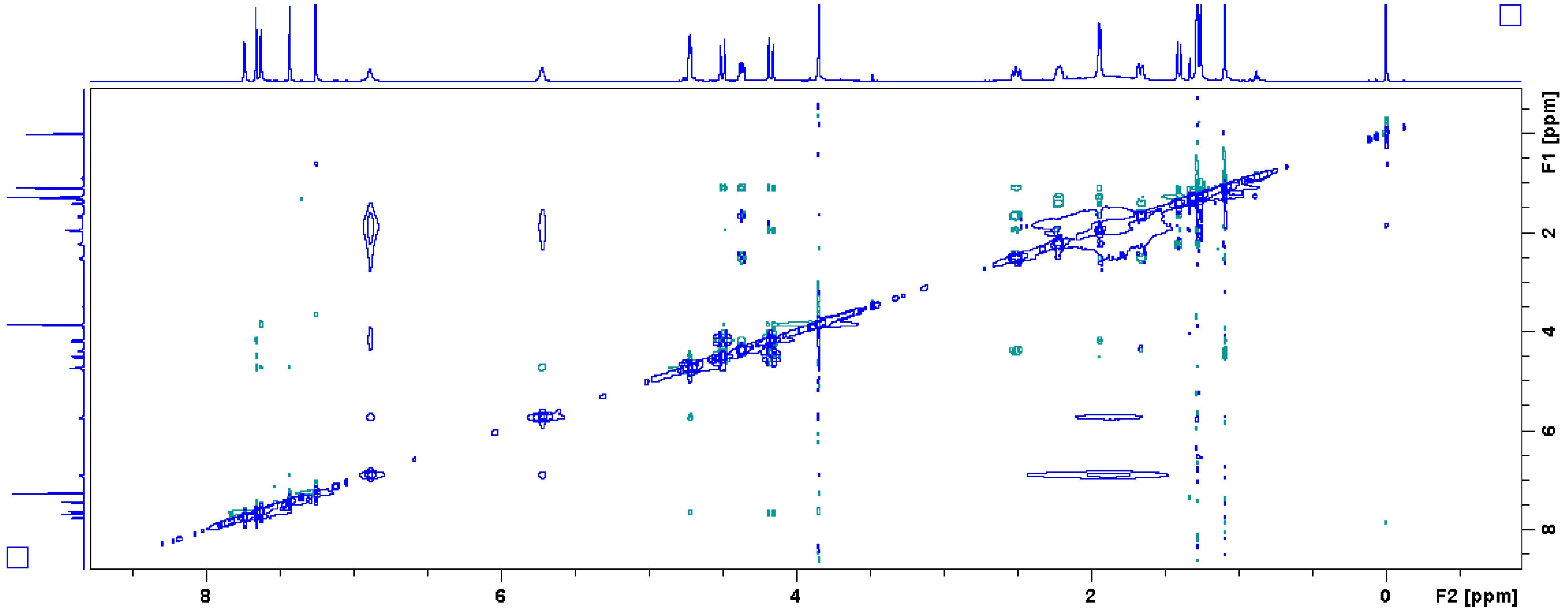

Figure S 78. HSQC-NMR of compound **(+)-25**

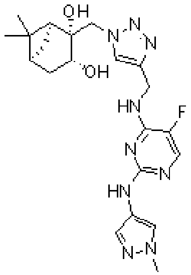

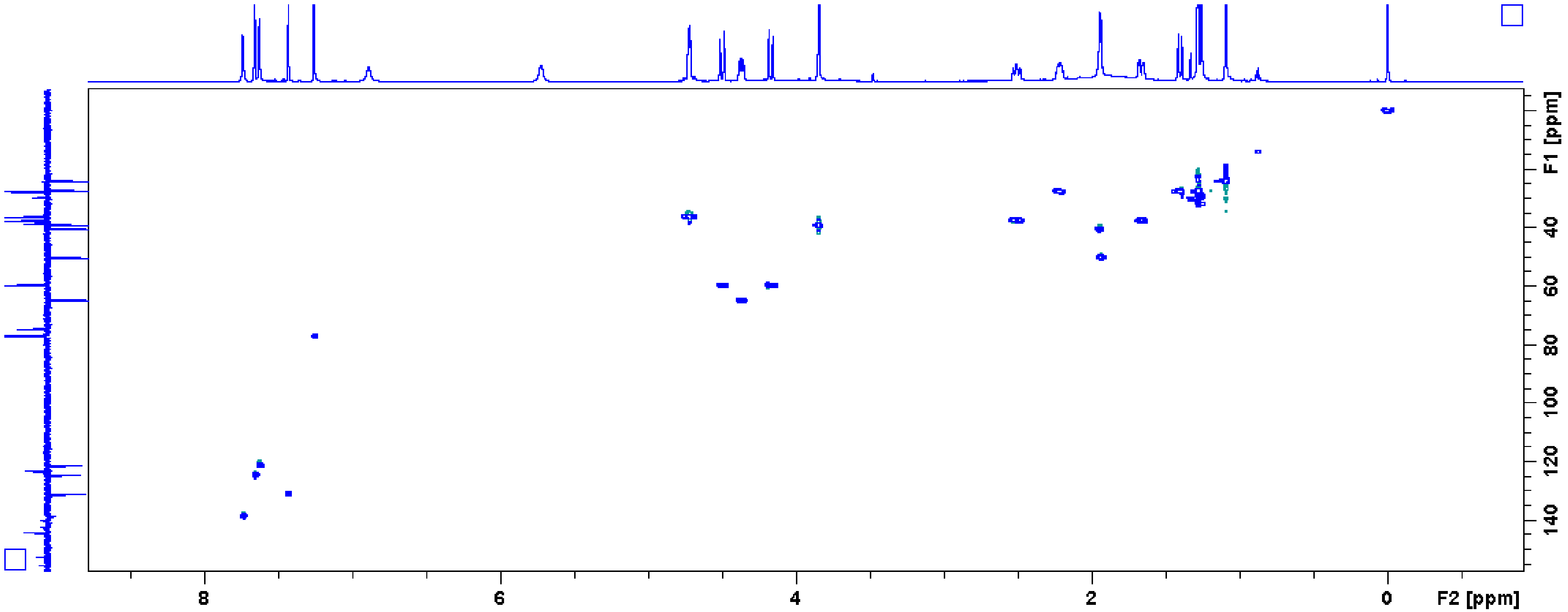

Figure S 79. HMBC-NMR of compound **(+)-25**

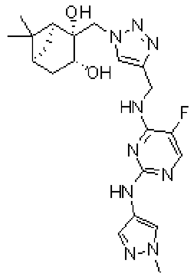

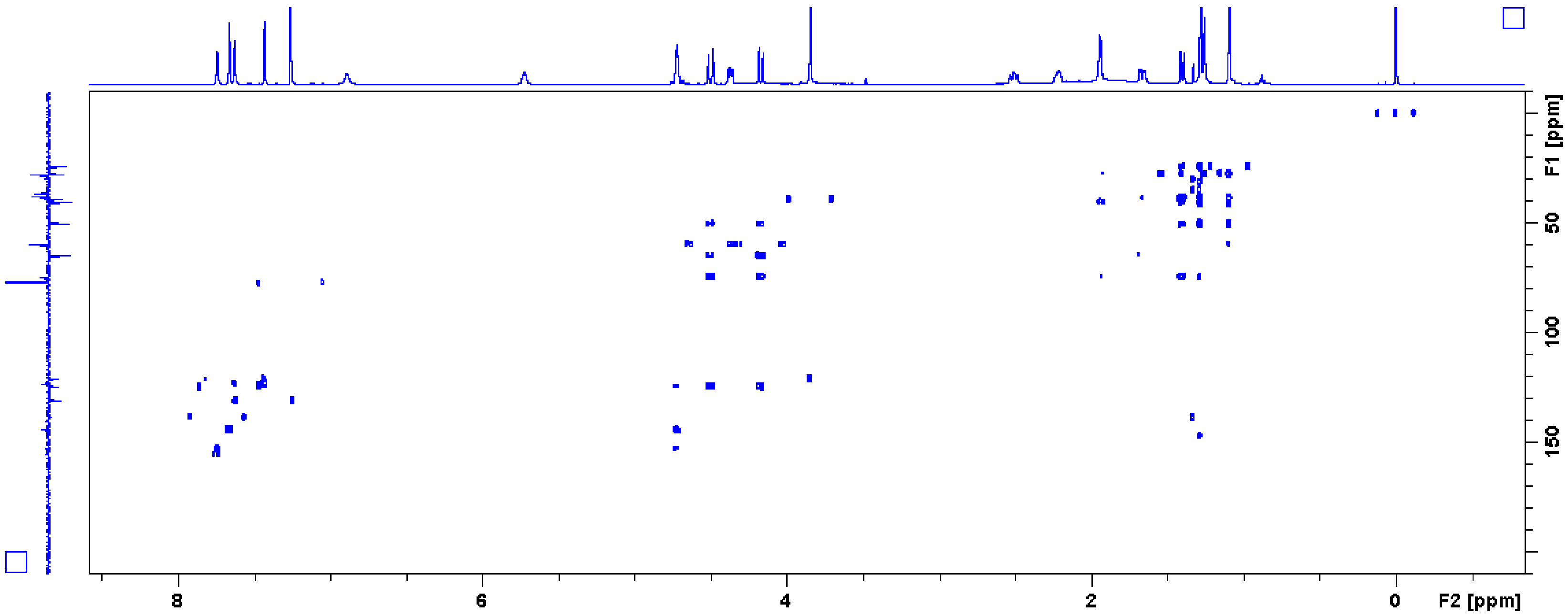

Figure S 80. ^{19}F-NMR of compound **(+)-25**

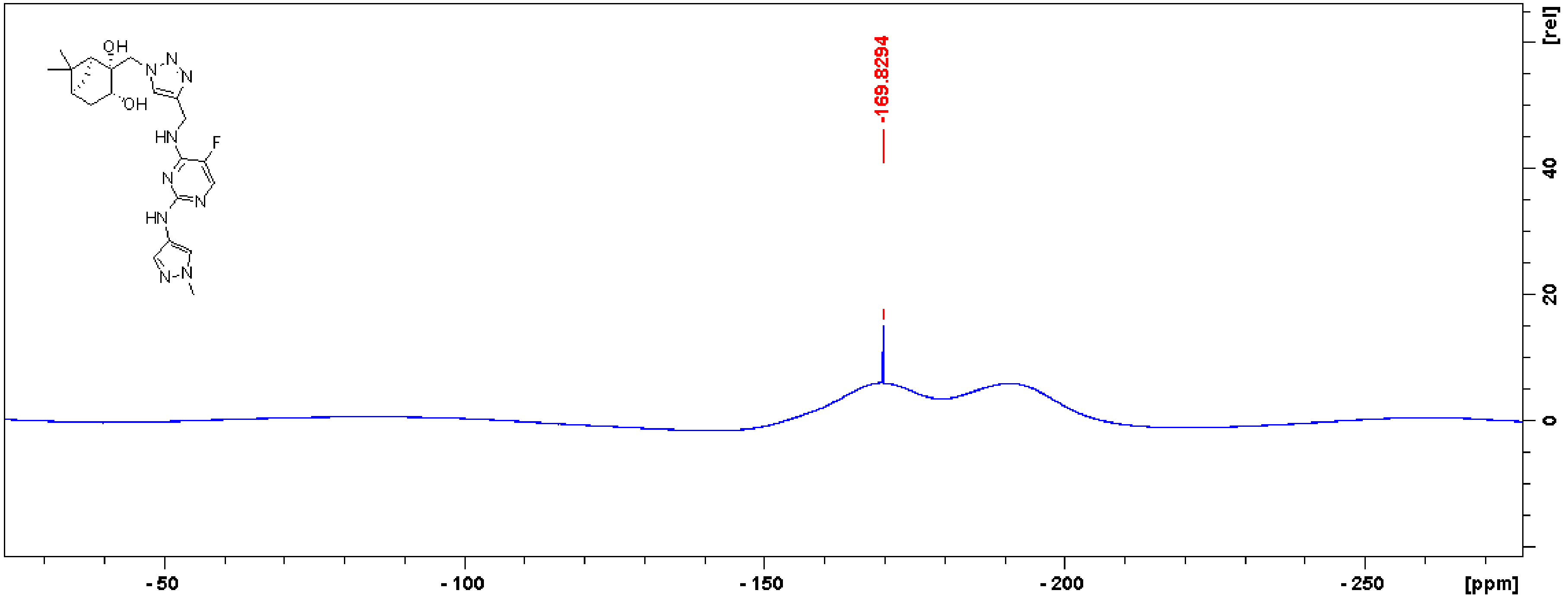

(1*R*,2*S*,3*S*,5*R*)-2-((4-(((5-fluoro-2-((1-methyl-1H-pyrazol-4-yl)amino)pyrimidin-4-yl)amino)methyl)-1H-1,2,3-triazol-1-yl)methyl)-6,6-dimethylbicyclo[3.1.1]heptane-2,3-diol **(–)-25**

Figure S 81. ^{1}H-NMR of compound **(–)-25**

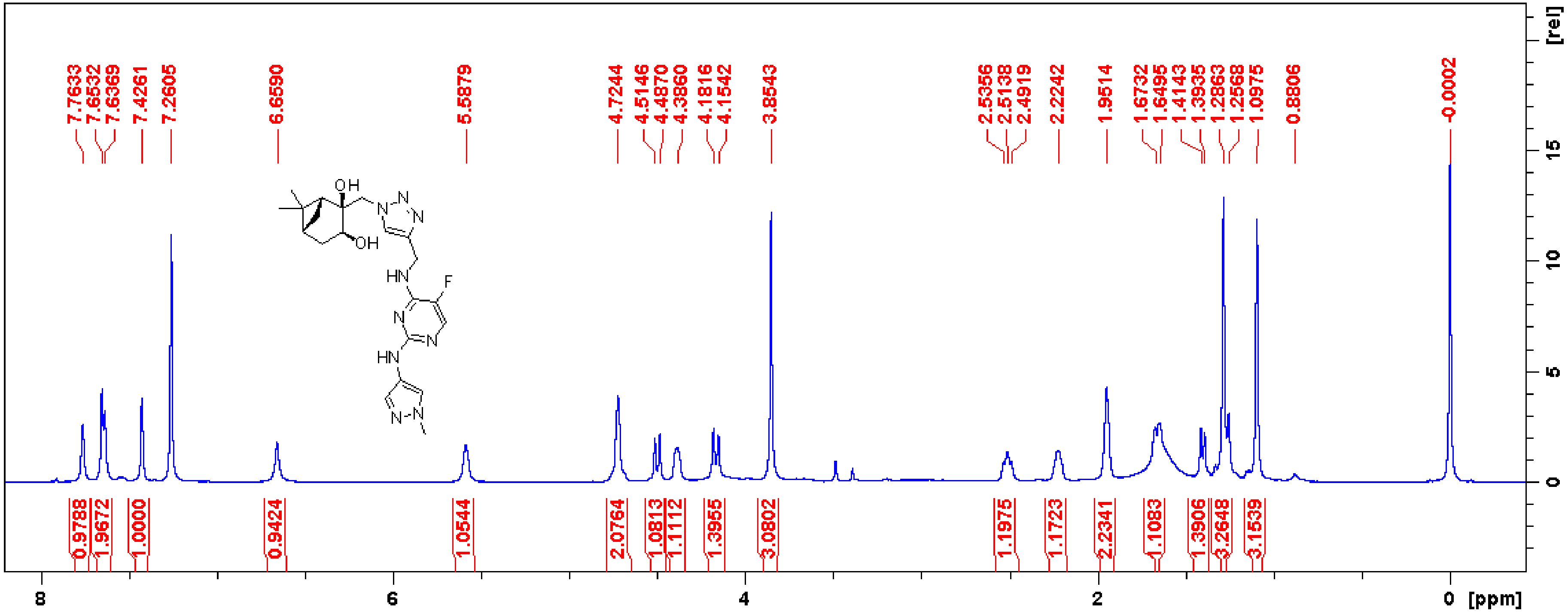

Figure S 82. ^{13}C-NMR of compound **(–)-25**

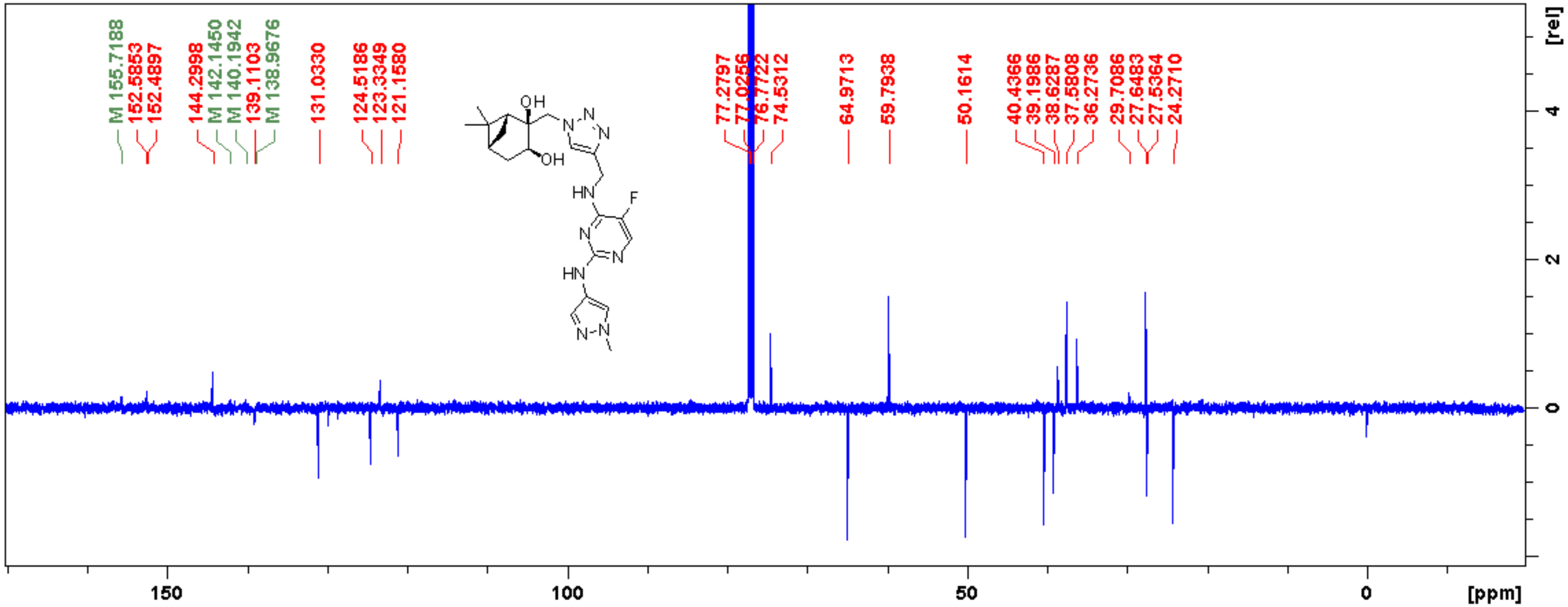

Figure S 83. COSY-NMR of compound **(–)-25**

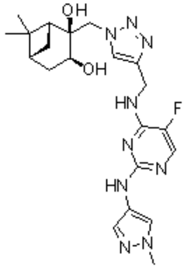

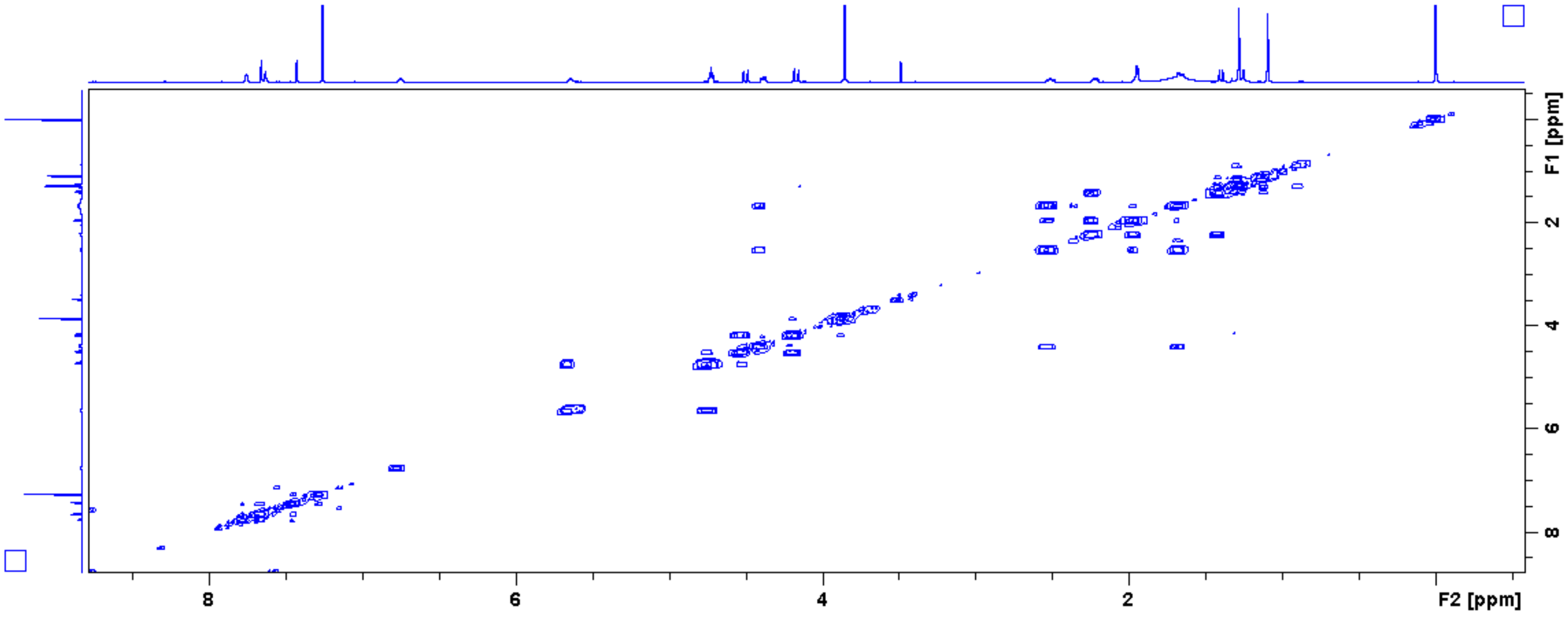

Figure S 84. NOESY-NMR of compound **(–)-25**

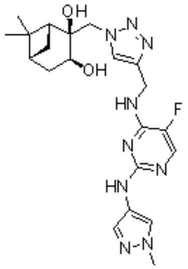

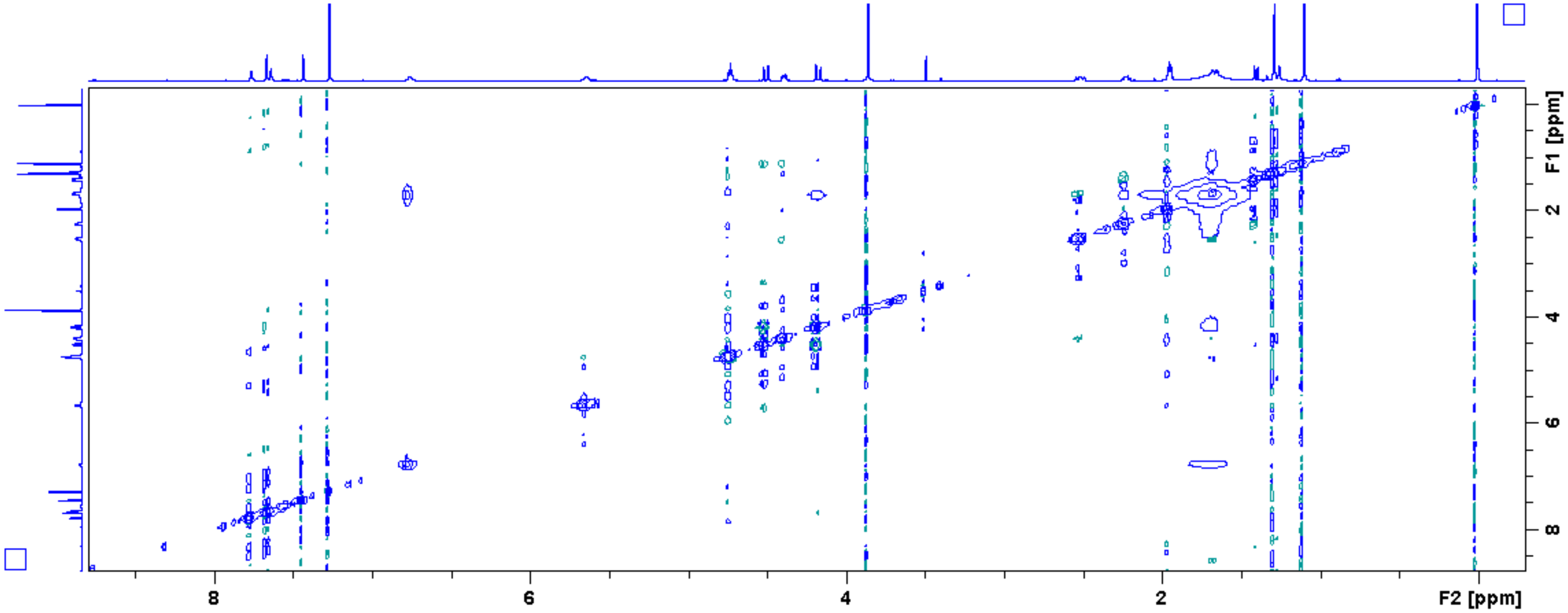

Figure S 85. HSQC-NMR of compound **(–)-25**

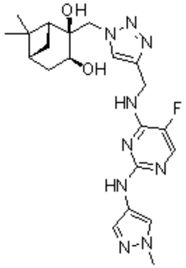

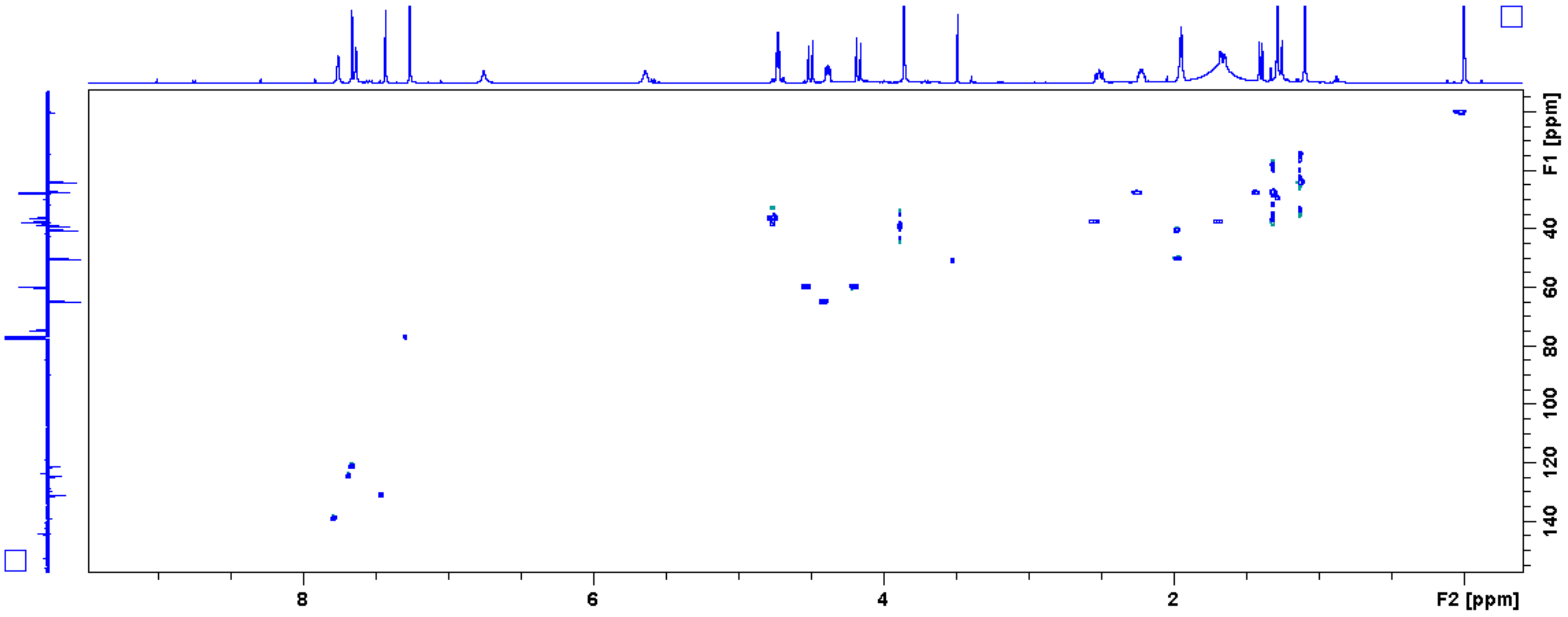

Figure S 86. HMBC-NMR of compound **(–)-25**

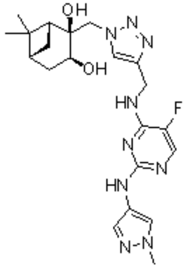

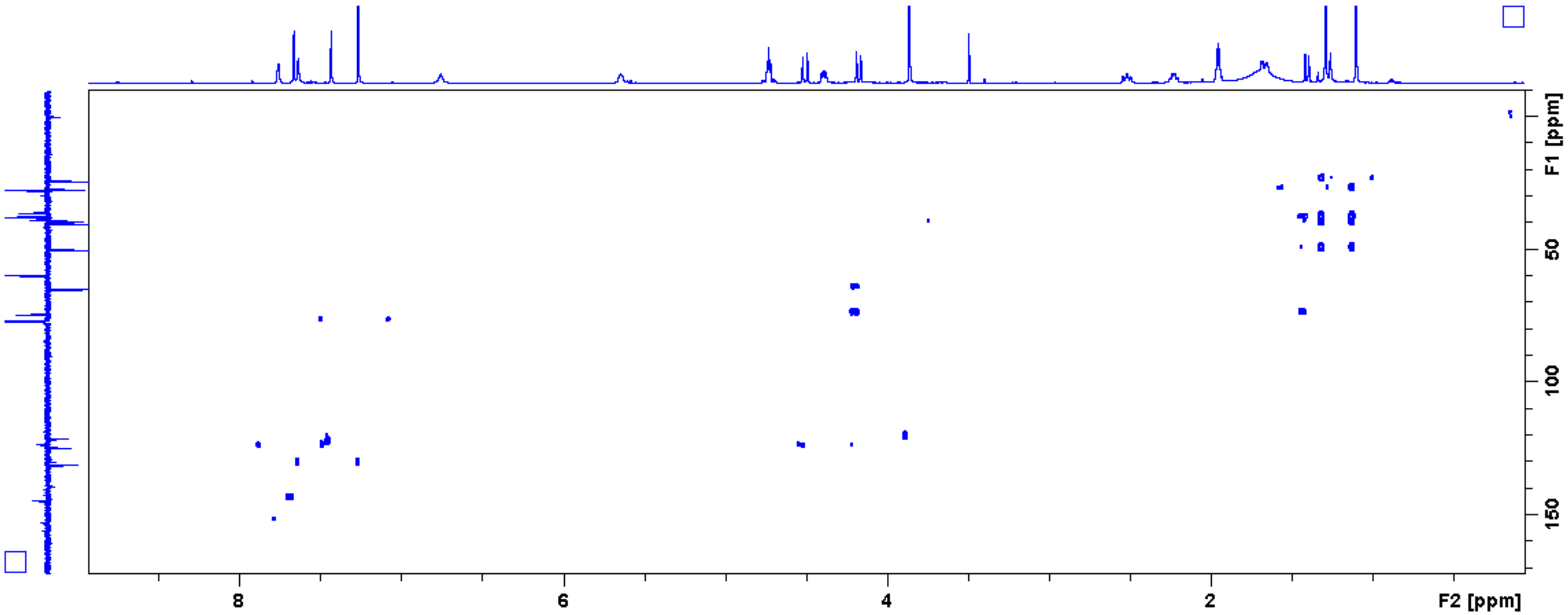

Figure S 87. ^{19}F-NMR of compound **(–)-25**

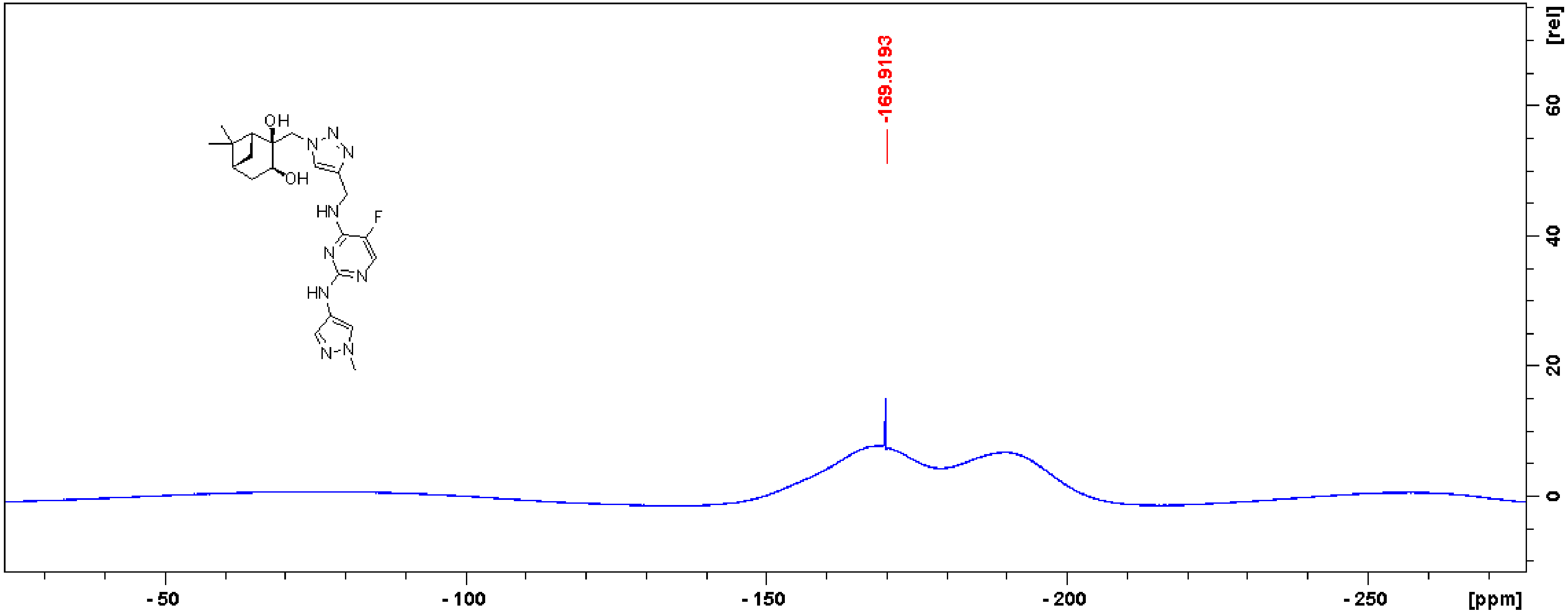

(1*S*,2*R*,3*R*,5*S*)-2-((4-(((5-Chloro-2-((1-methyl-1H-pyrazol-4-yl)amino)pyrimidin-4-yl)amino)methyl)-1*H*-1,2,3-triazol-1-yl)methyl)-6,6-dimethylbicyclo[3.1.1]heptane-2,3-diol **(+)-26**

Figure S 88. ^{1}H-NMR of compound **(+)-26**

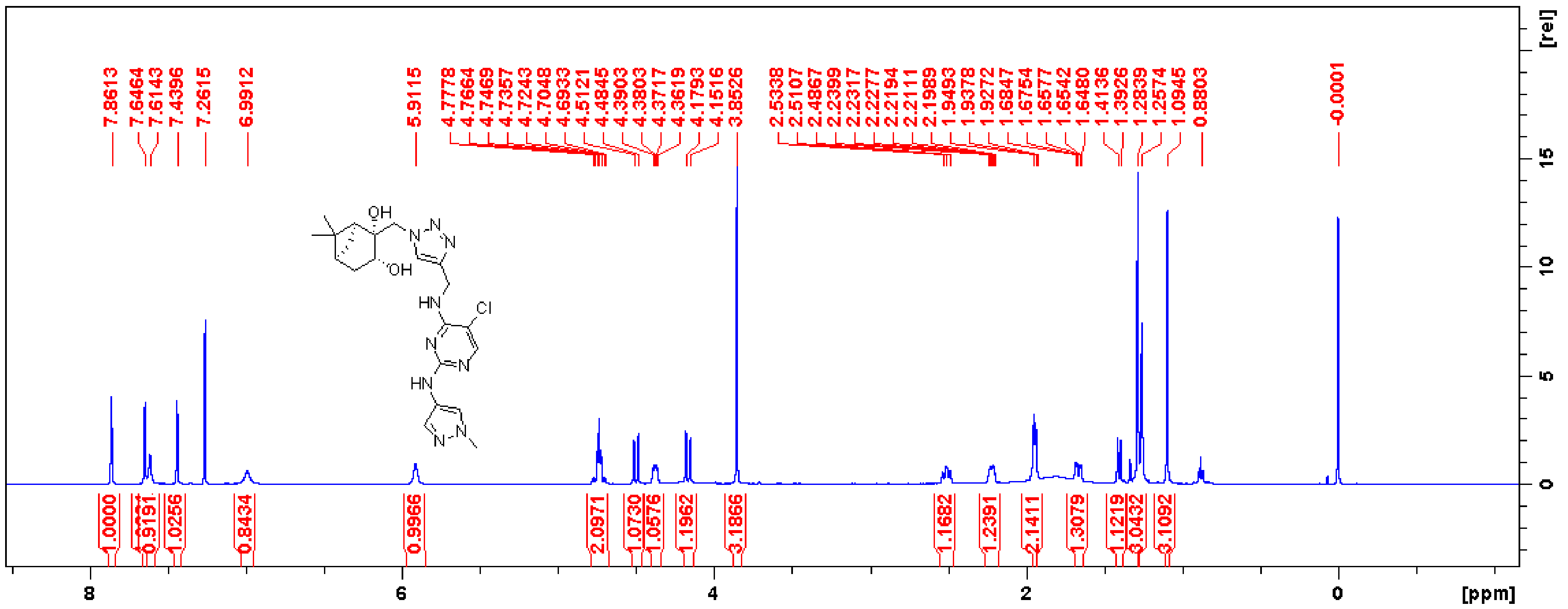

Figure S 89. ^{13}C-NMR of compound **(+)-26**

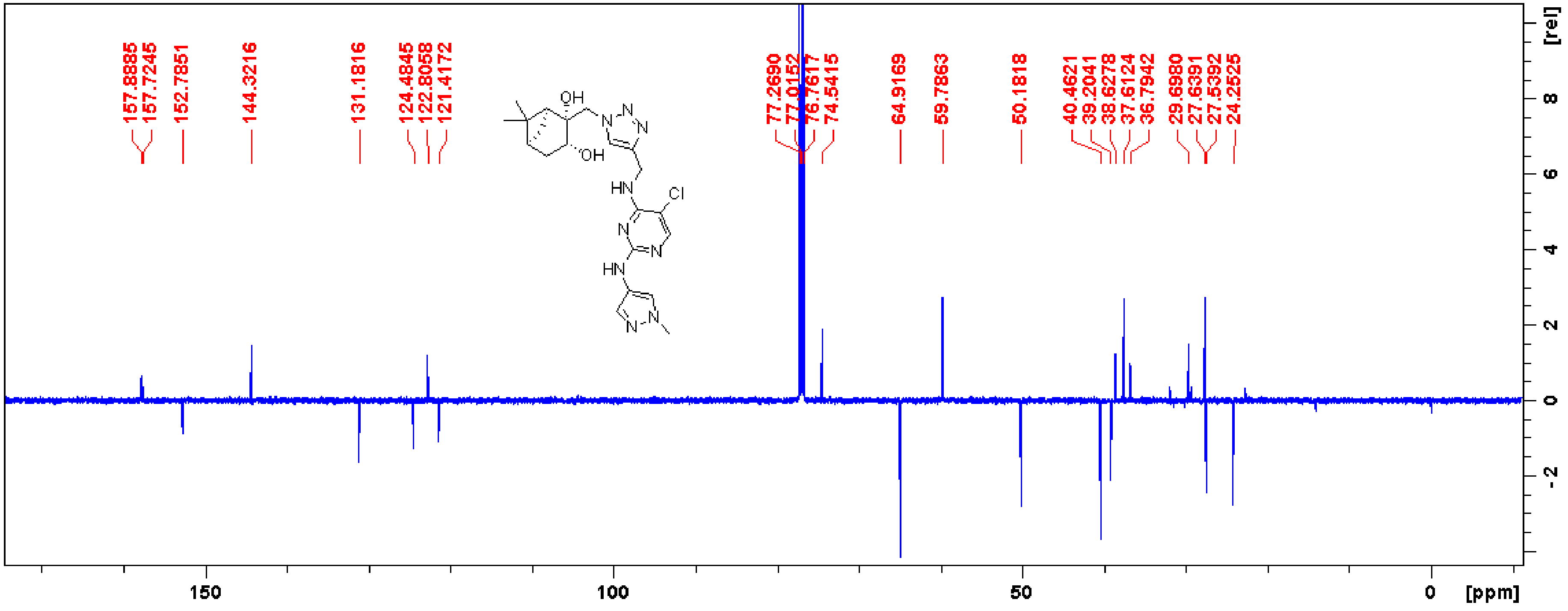

Figure S 90. COSY-NMR of compound **(+)-26**

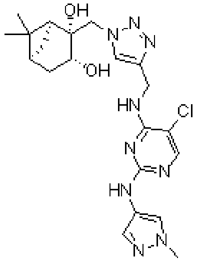

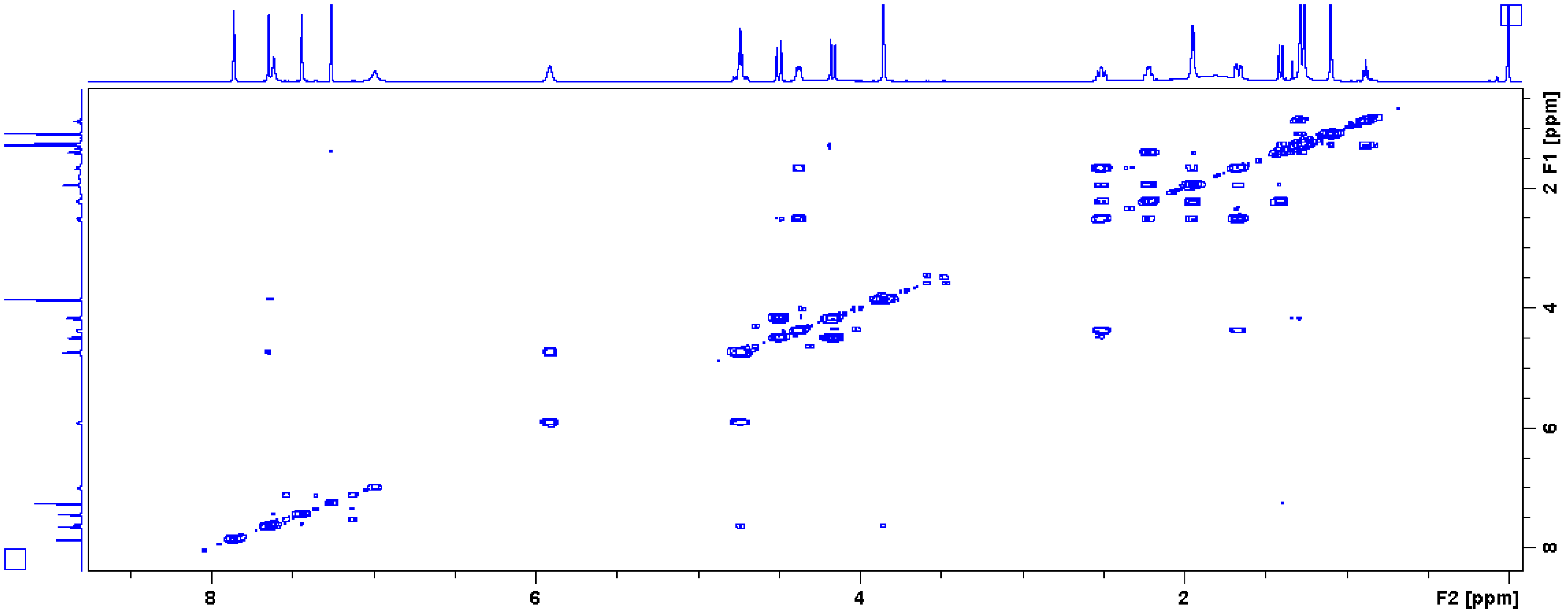

Figure S 91. NOESY-NMR of compound **(+)-26**

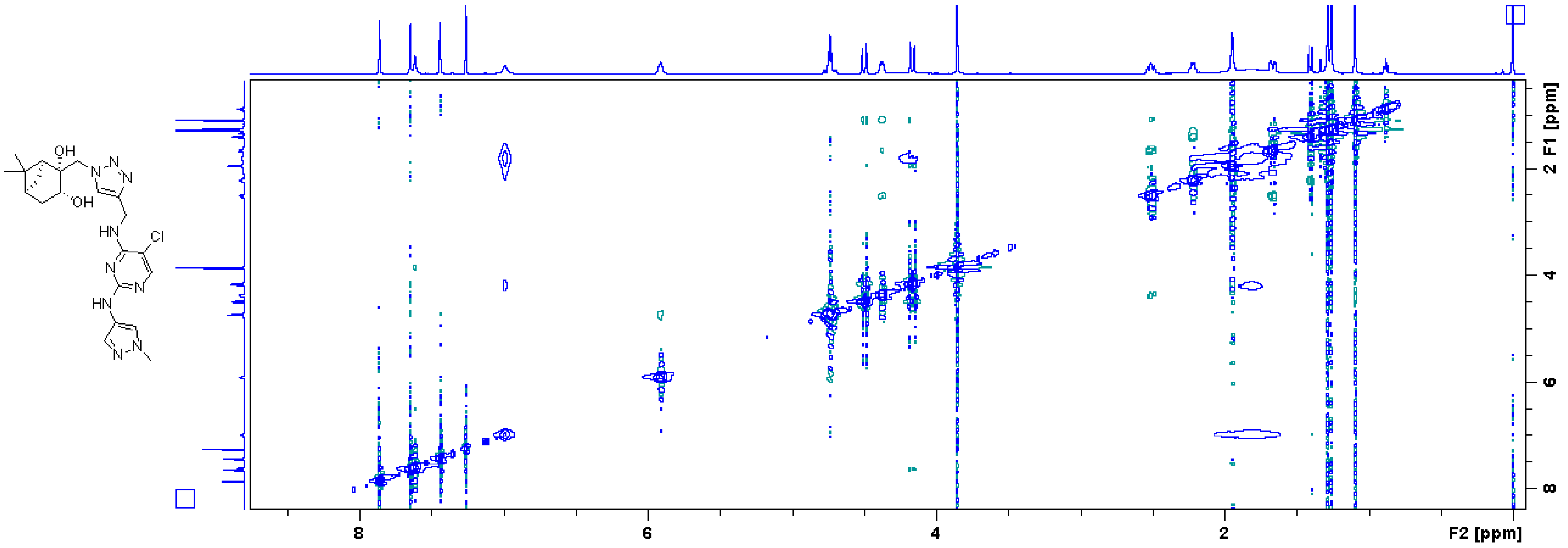

Figure S 92. HSQC-NMR of compound **(+)-26**

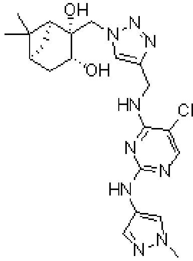

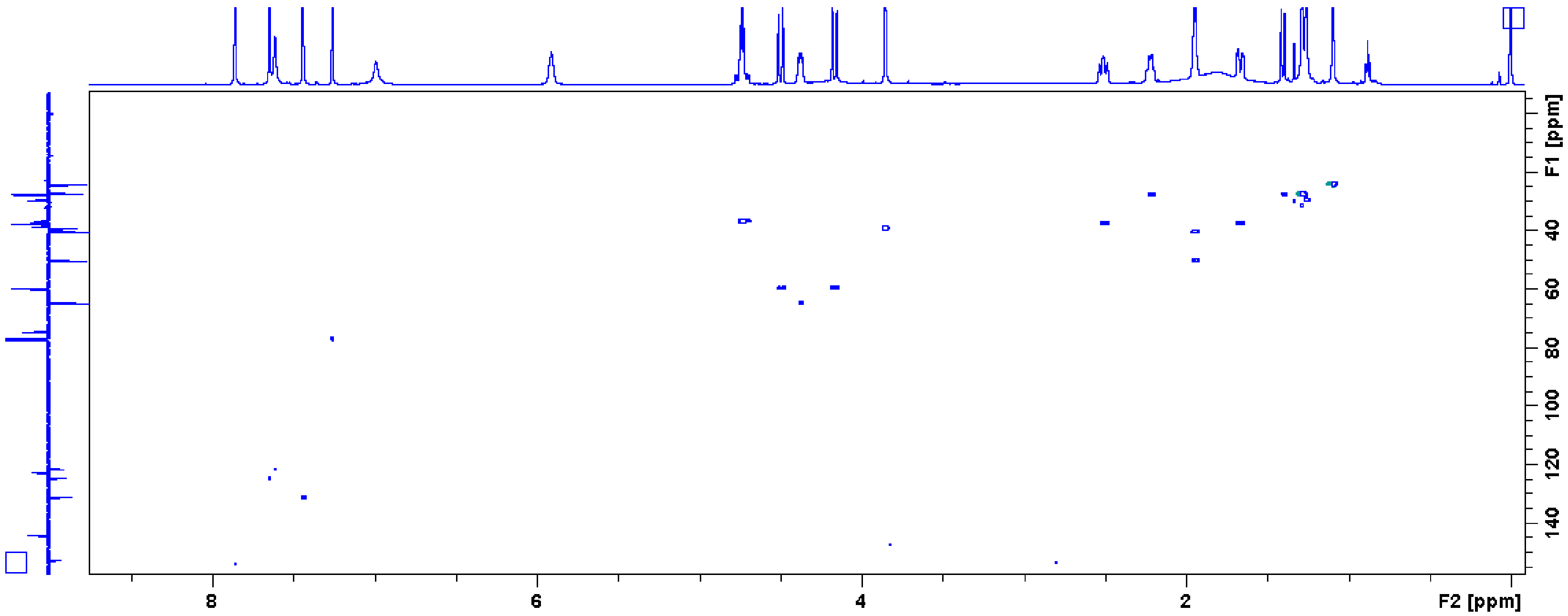

Figure S 93. HMBC-NMR of compound **(+)-26**

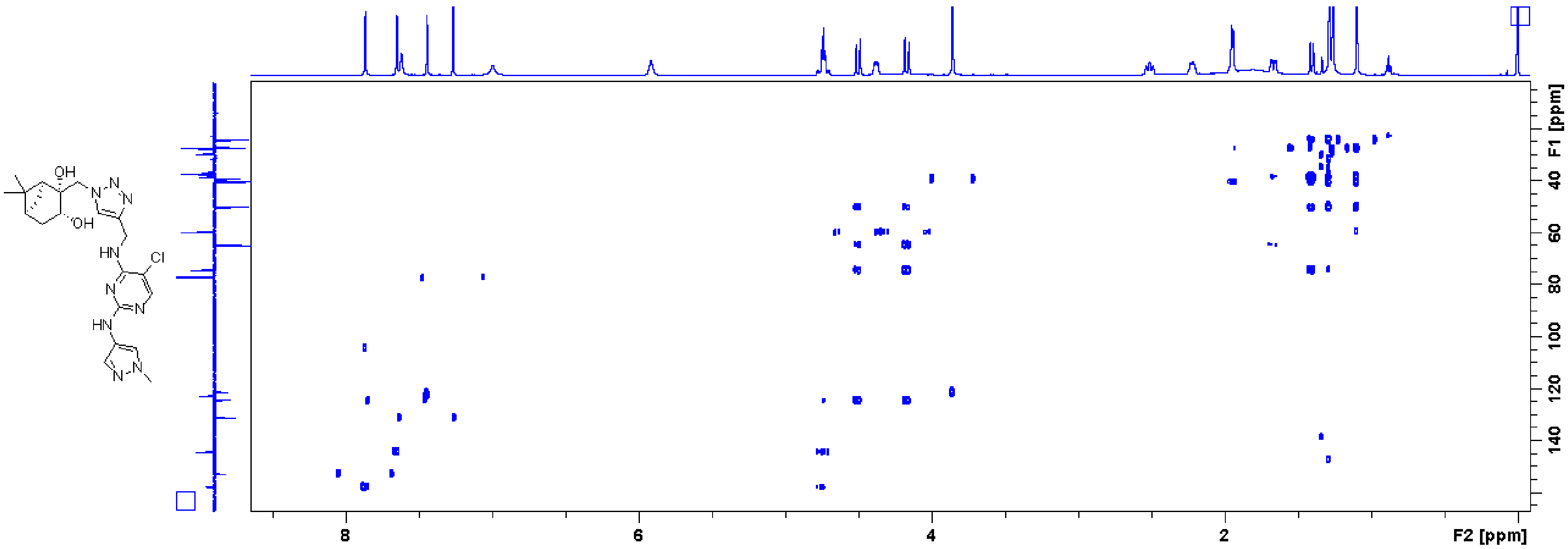

(1*R*,2*S*,3*S*,5*R*)-2-((4-(((5-chloro-2-((1-methyl-1H-pyrazol-4-yl)amino)pyrimidin-4-yl)amino)methyl)-1H-1,2,3-triazol-1-yl)methyl)-6,6-dimethylbicyclo[3.1.1]heptane-2,3-diol **(–)-26**

Figure S 94. ^{1}H-NMR of compound **(–)-26**

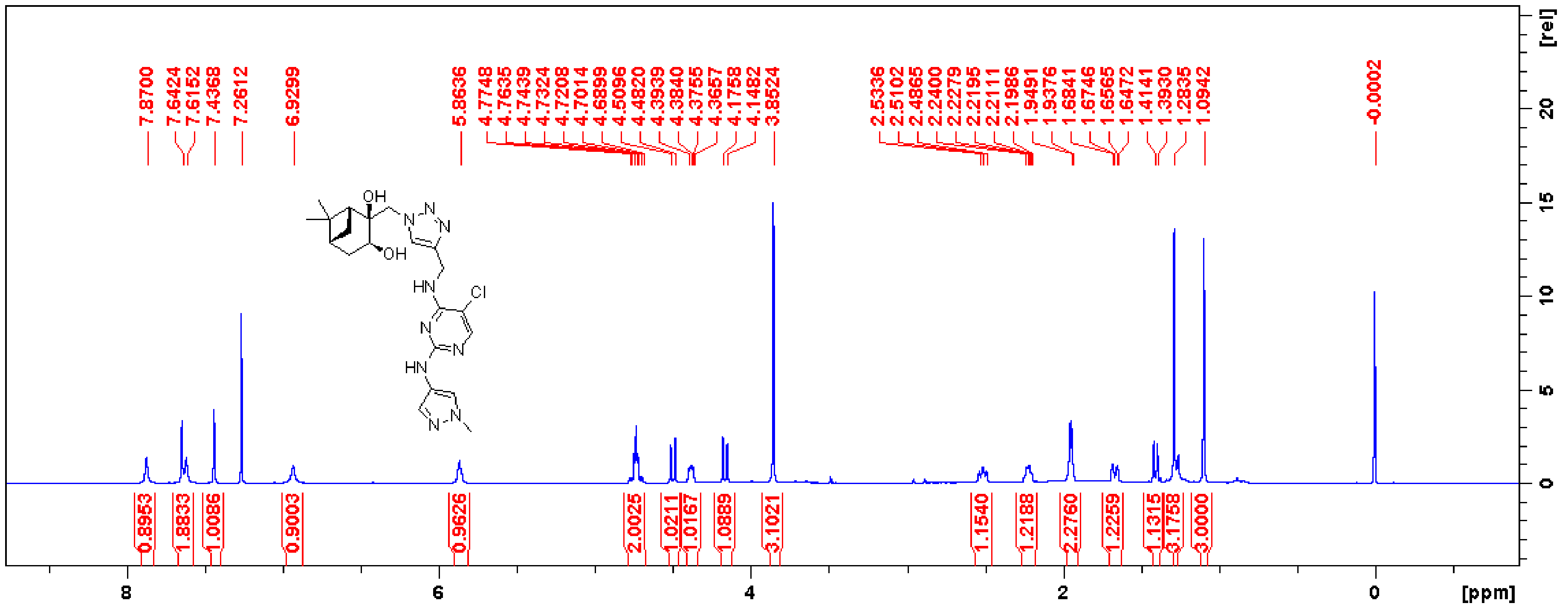

Figure S 95. ^{13}C-NMR of compound **(–)-26**

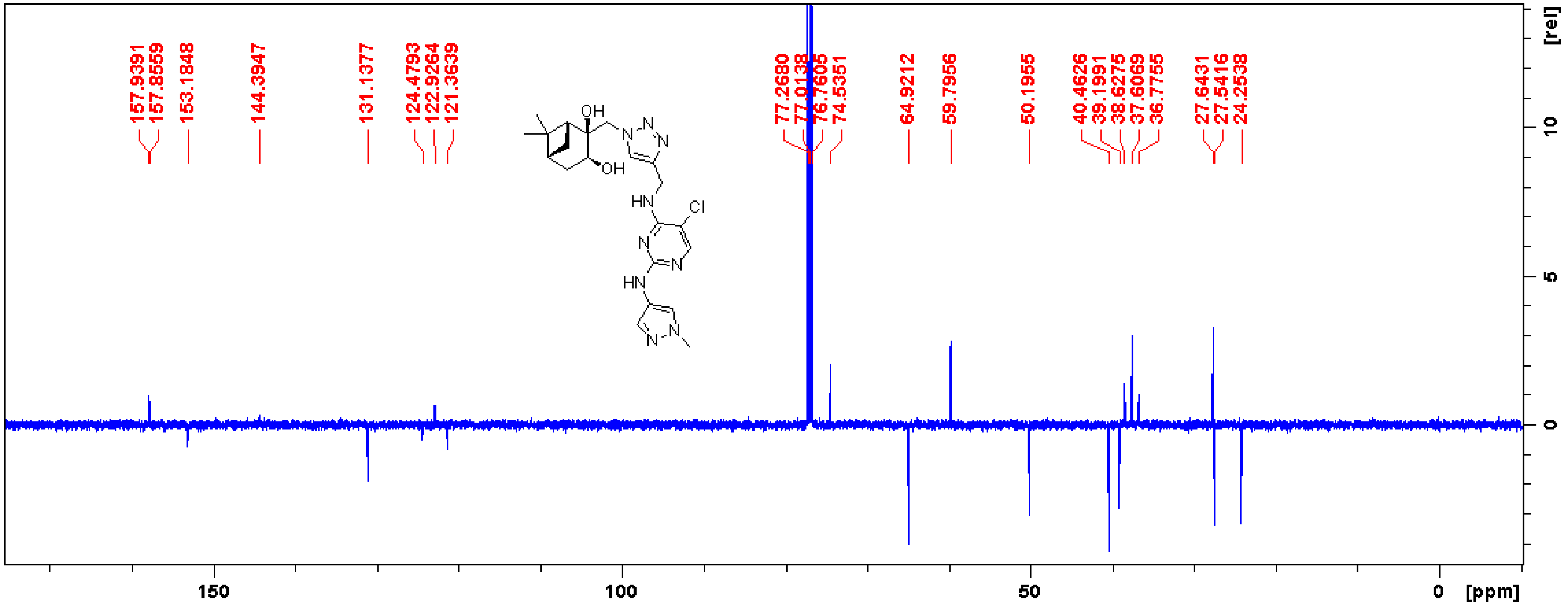

Figure S 96. COSY-NMR of compound **(–)-26**

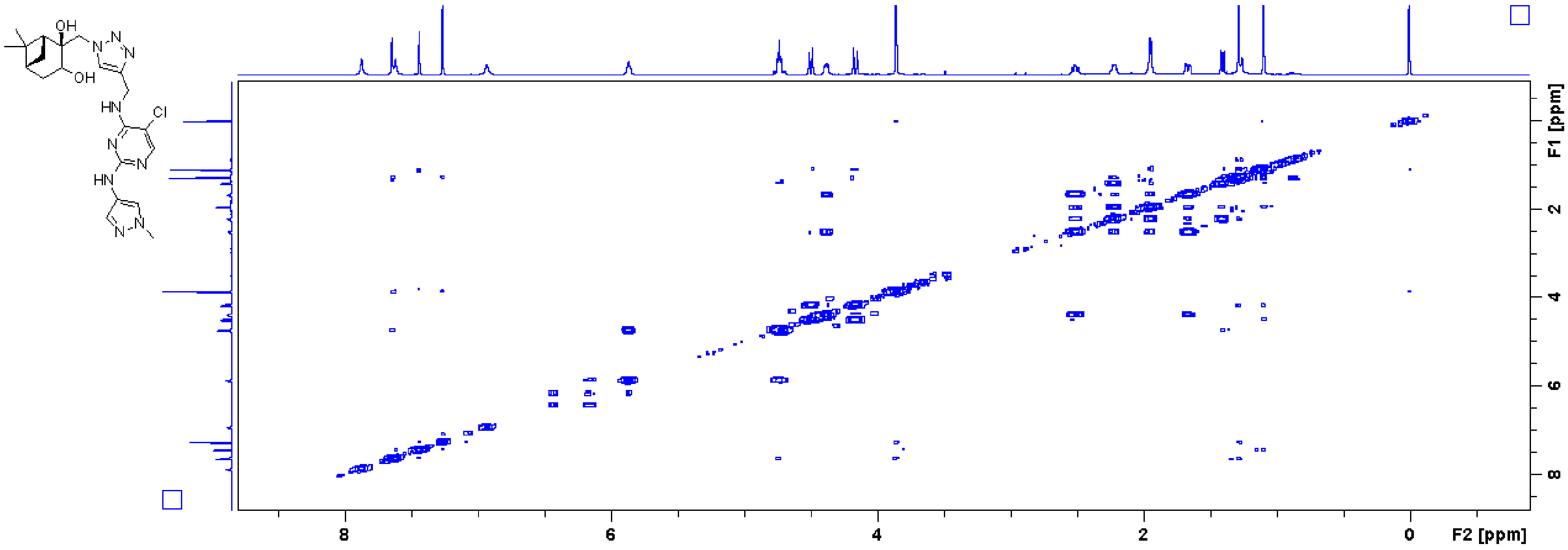

Figure S 97. NOESY-NMR of compound **(–)-26**

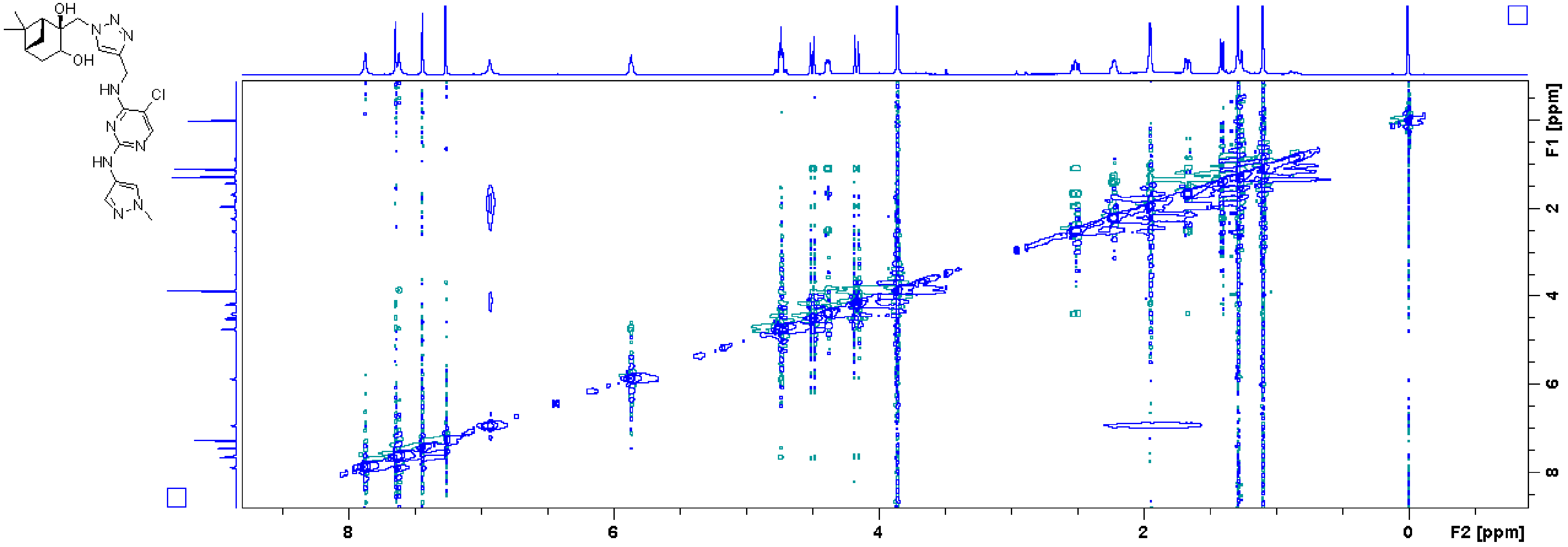

Figure S 98. HSQC-NMR of compound **(–)-26**

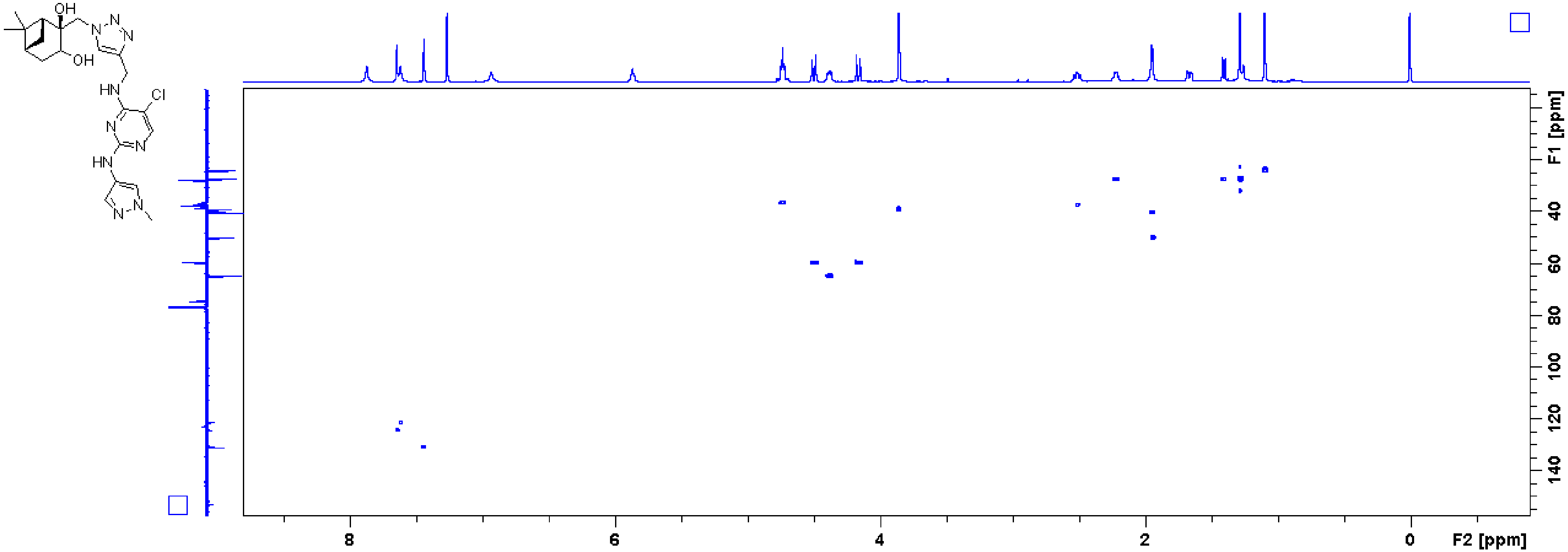

Figure S 99. HMBC-NMR of compound **(–)-26**

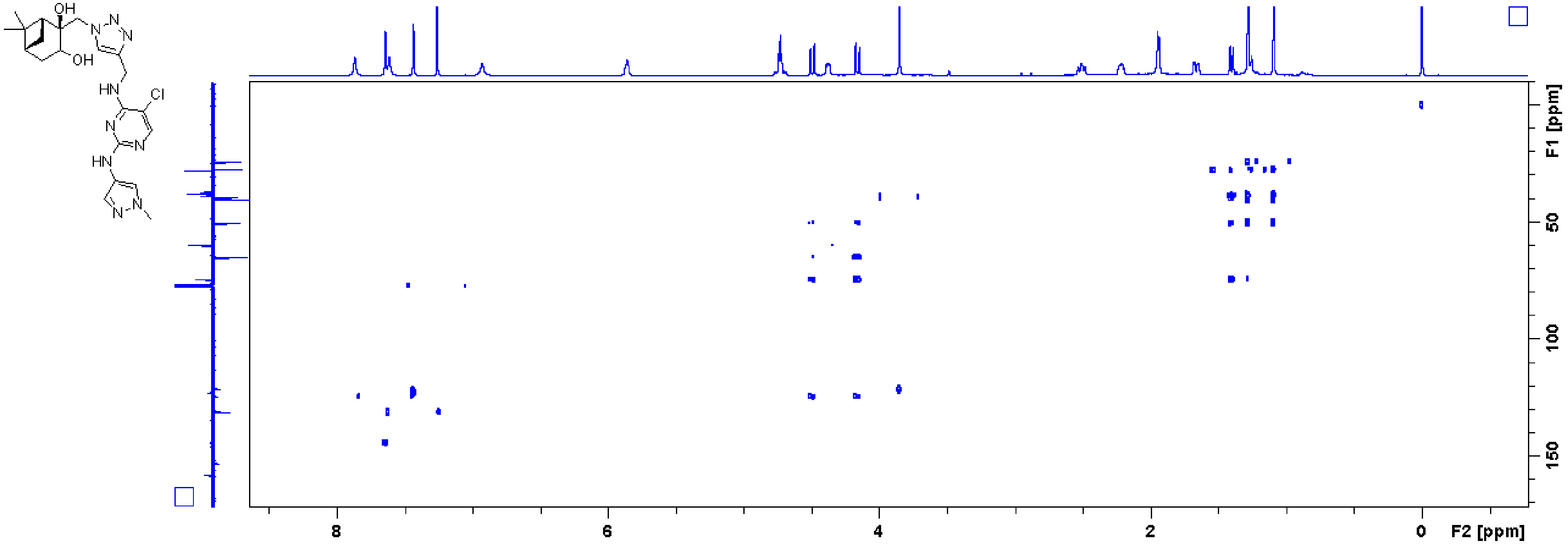

methyl 4-((5-Chloro-4-(((1-(((1*S*,2*R*,3*R*,5*S*)-2,3-dihydroxy-6,6-dimethylbicyclo[3.1.1]heptan-2-yl)methyl)-1*H*-1,2,3-triazol-4-yl)methyl)amino)pyrimidin-2-yl)amino)benzoate **(+)-27**

Figure S 100. ^{1}H-NMR of compound **(+)-27**

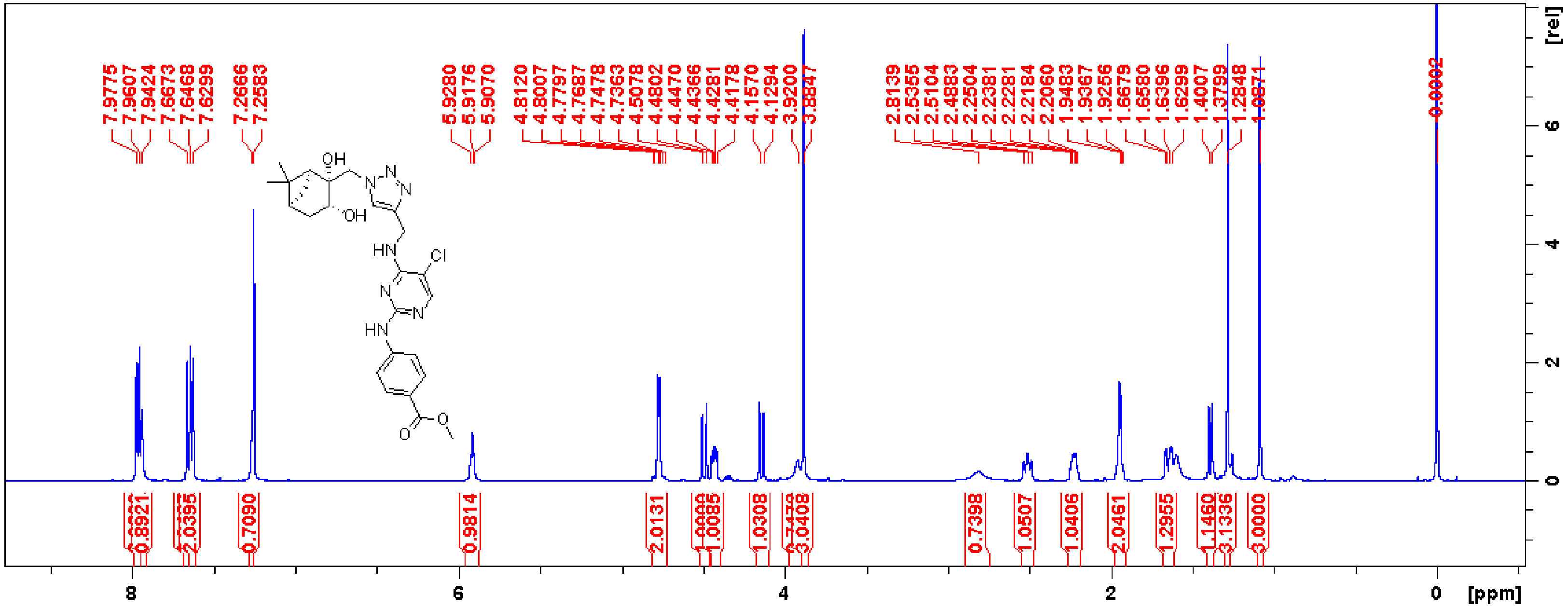

Figure S 101. ^{13}C-NMR of compound **(+)-27**

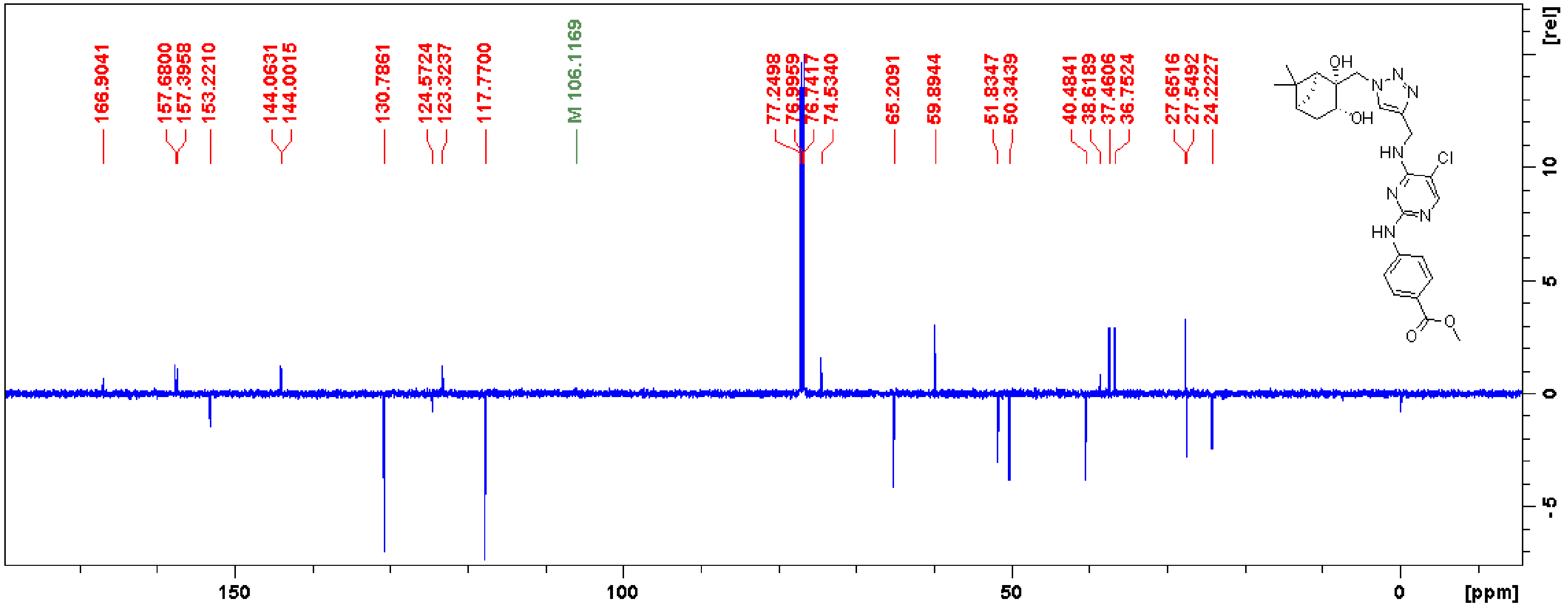

Figure S 102. COSY-NMR of compound **(+)-27**

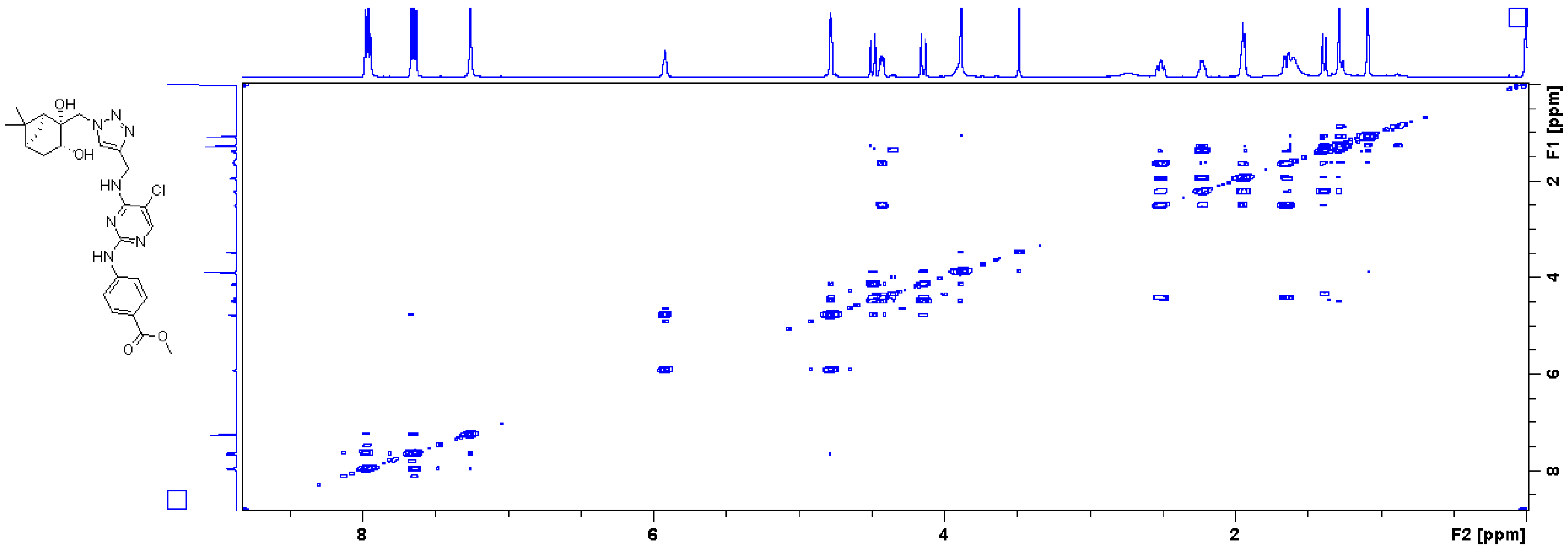

Figure S 103. NOESY-NMR of compound **(+)-27**

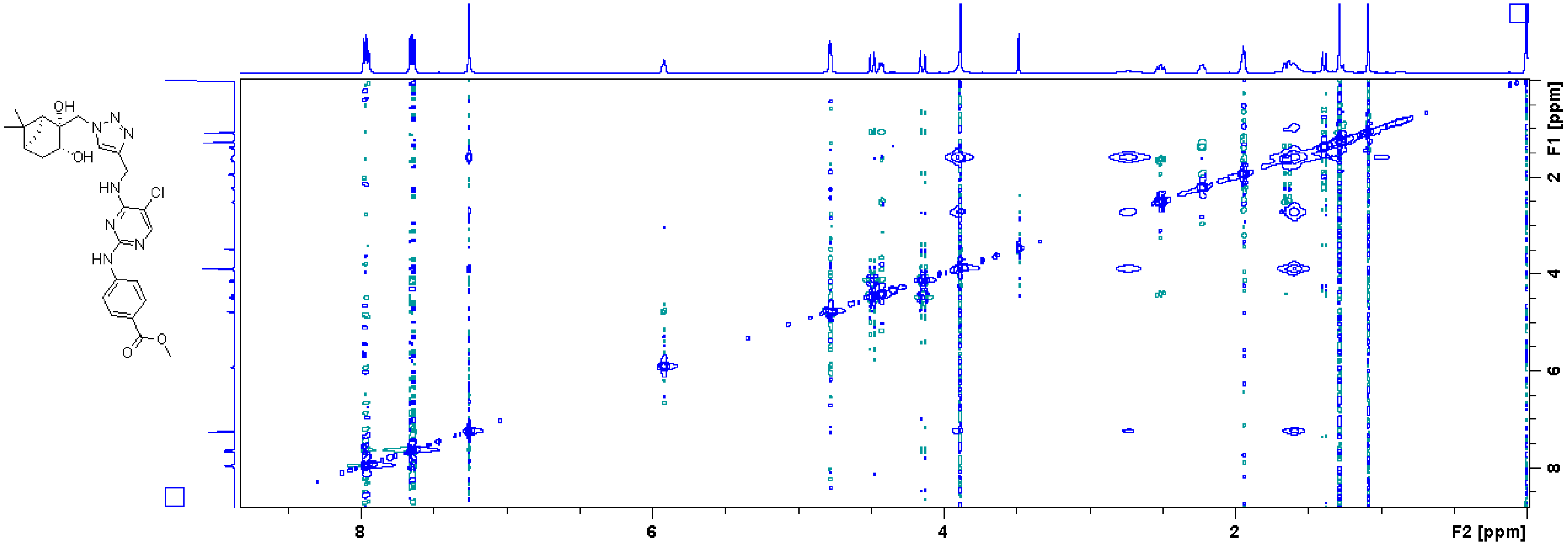

Figure S 104. HSQC-NMR of compound **(+)-27**

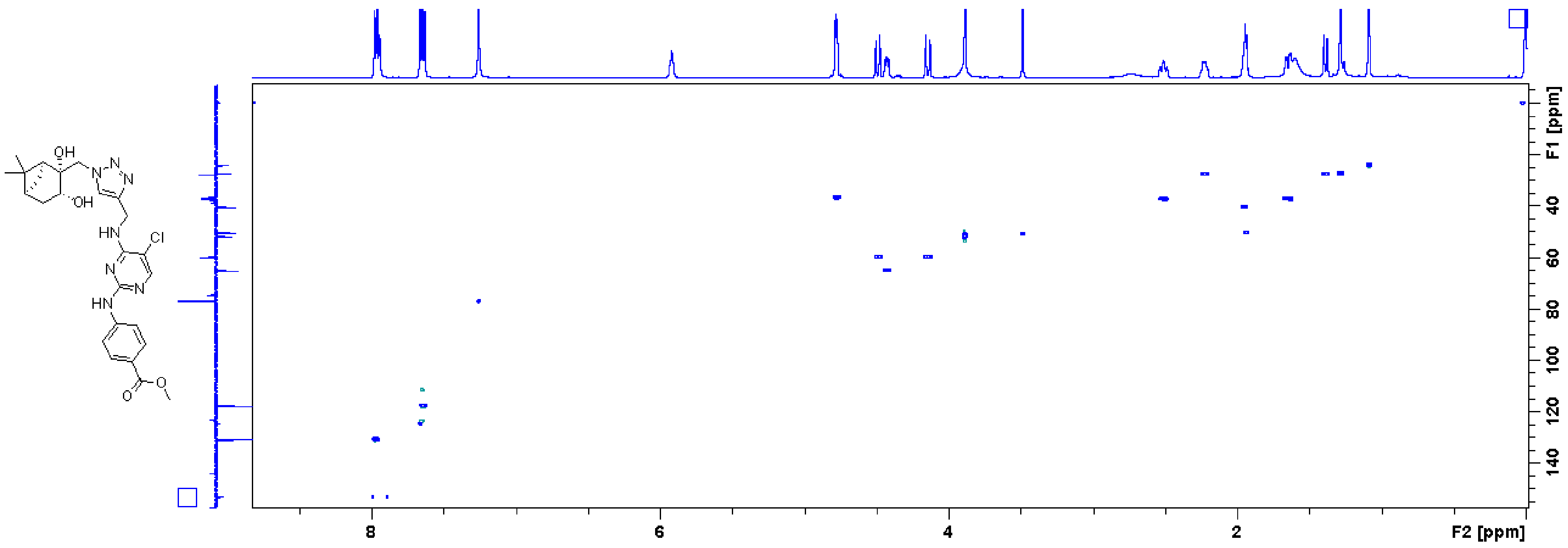

Figure S 105. HMBC-NMR of compound **(+)-27**

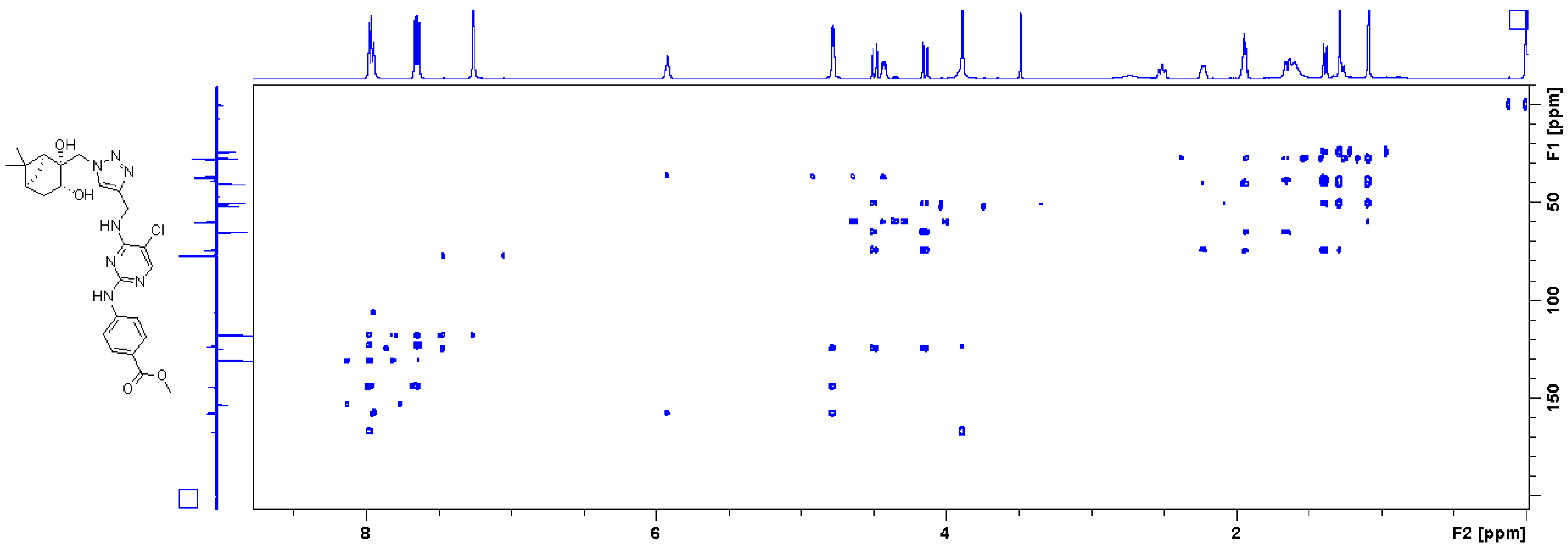

methyl 4-((5-chloro-4-(((1-(((1*R*,2*S*,3*S*,5*R*)-2,3-dihydroxy-6,6-dimethylbicyclo[3.1.1]heptan-2-yl)methyl)-1H-1,2,3-triazol-4-yl)methyl)amino)pyrimidin-2-yl)amino)benzoate **(–)-27**

Figure S 106. ^{1}H-NMR of compound **(–)-27**

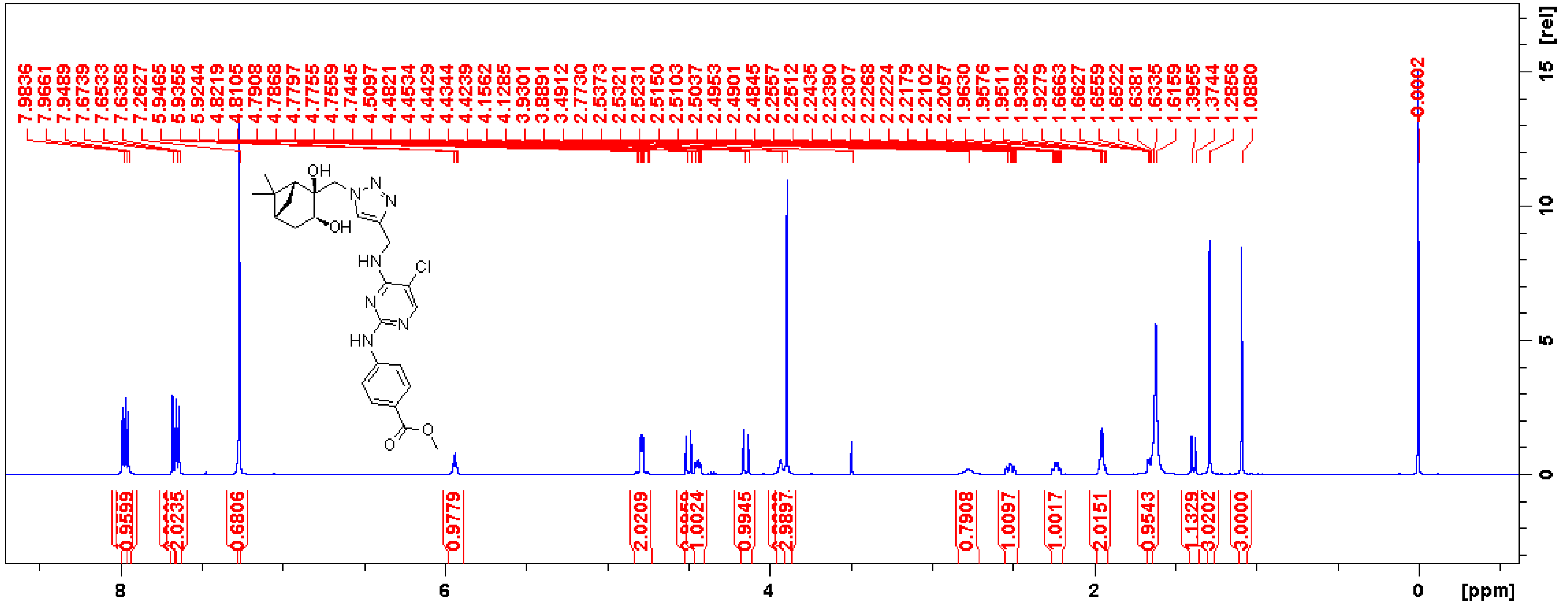

Figure S 107. ^{13}C-NMR of compound **(–)-27**

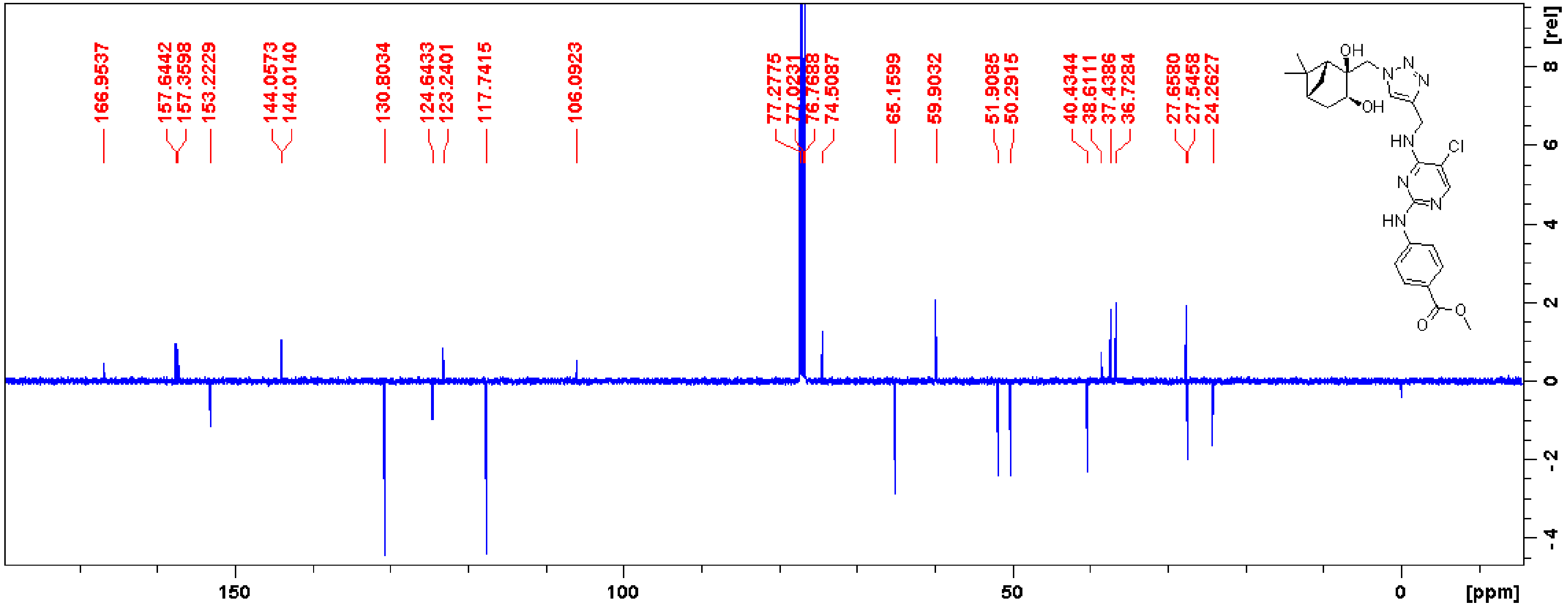

Figure S 108. COSY-NMR of compound **(–)-27**

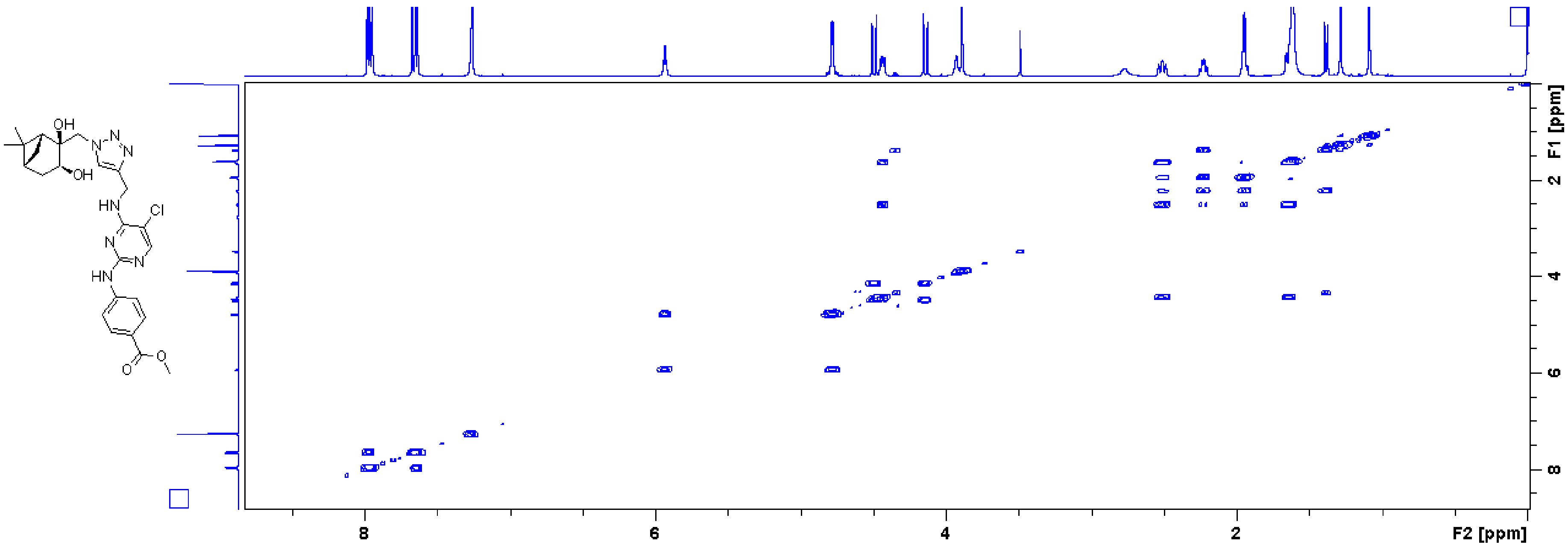

Figure S 109. NOESY-NMR of compound **(–)-27**

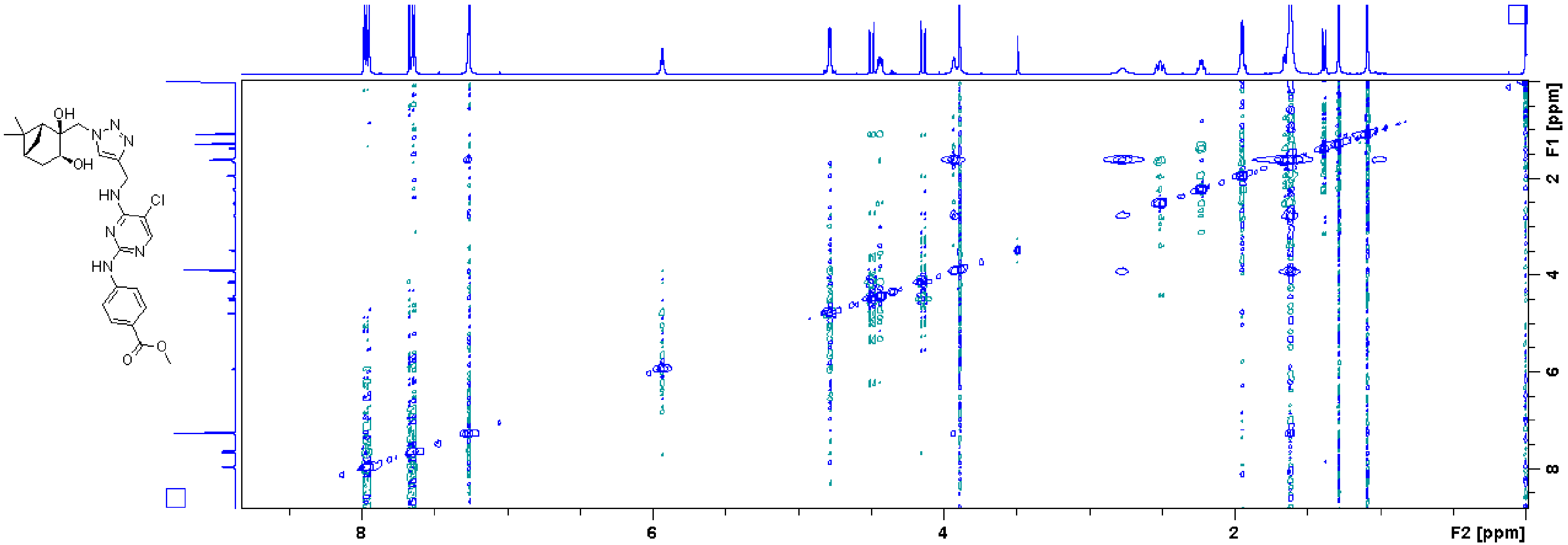

Figure S 110. HSQC-NMR of compound **(–)-27**

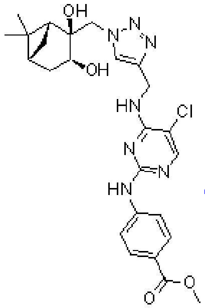

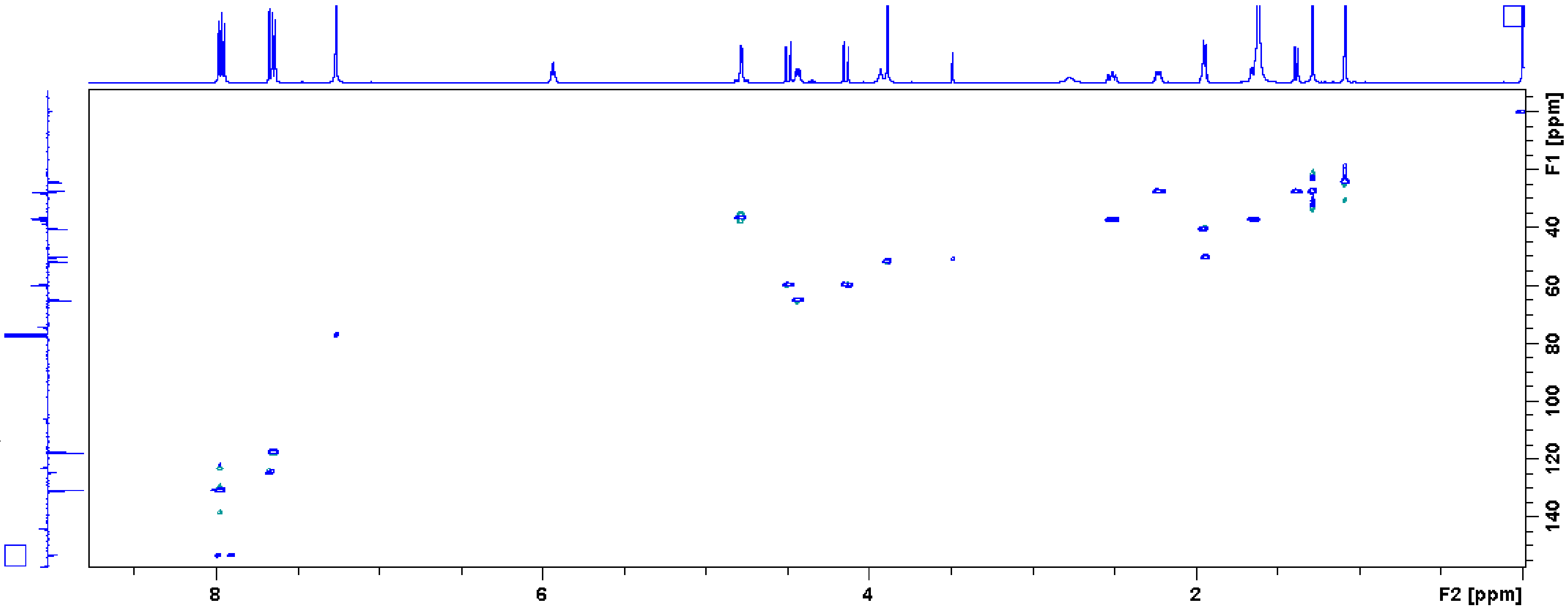

Figure S 111. HMBC-NMR of compound **(–)-27**

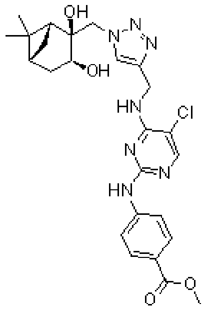

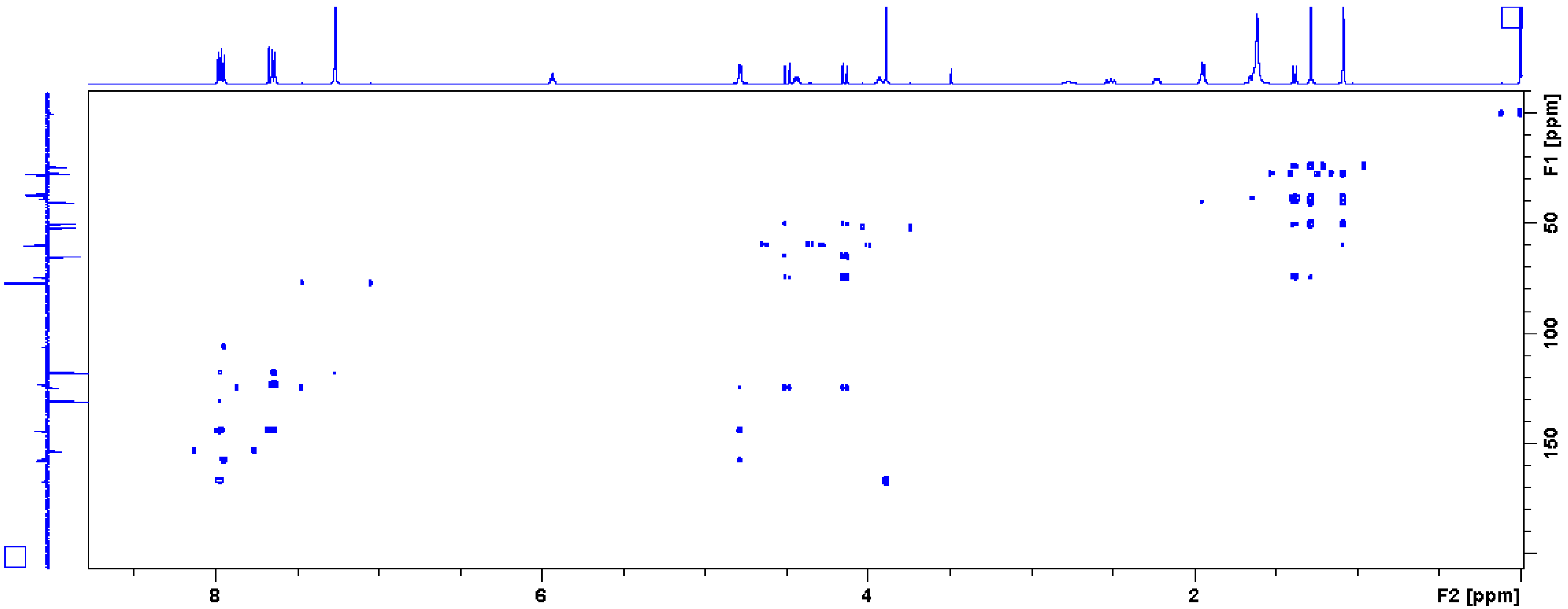

(1*S*,2*R*,3*R*,5*S*)-2-((4-(((5-Fluoro-2-((4-morpholinophenyl)amino)pyrimidin-4-yl)amino)methyl)-1*H*-1,2,3-triazol-1-yl)methyl)-6,6-dimethylbicyclo[3.1.1]heptane-2,3-diol **(+)-28**

Figure S 112. ^{1}H-NMR of compound **(+)-28**

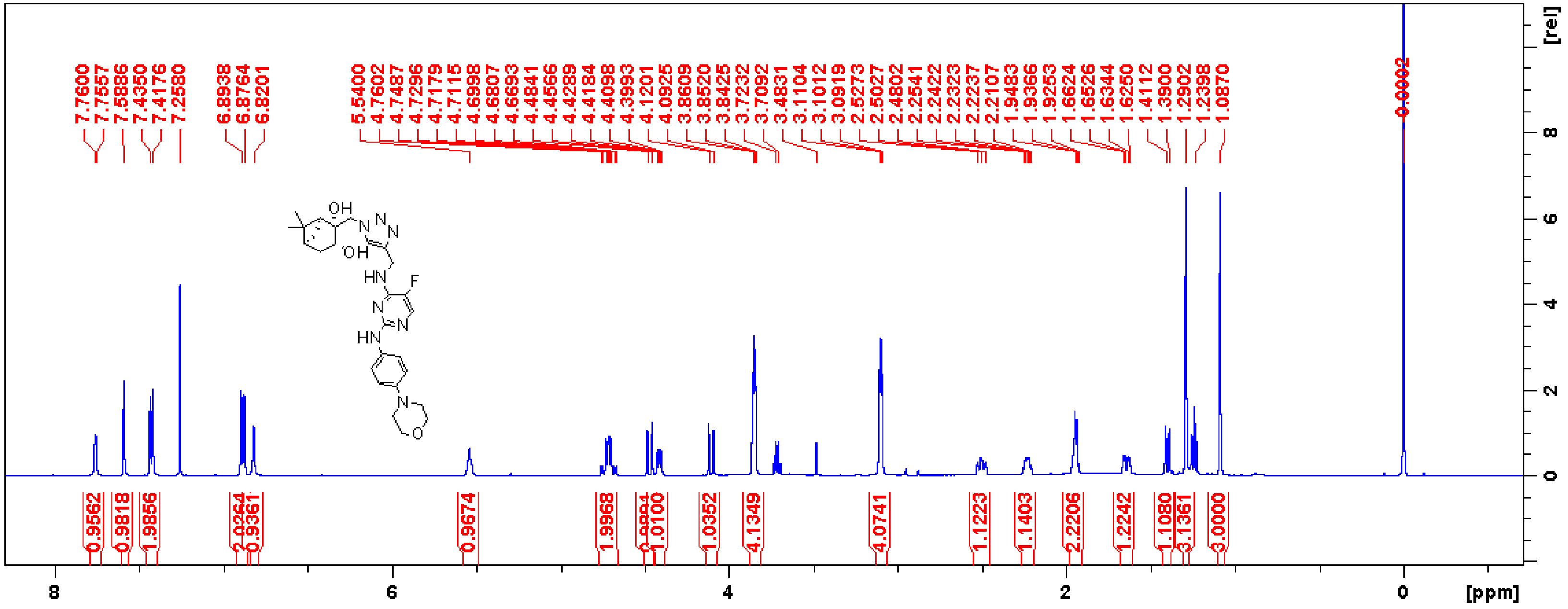

Figure S 113. ^{13}C-NMR of compound **(+)-28**

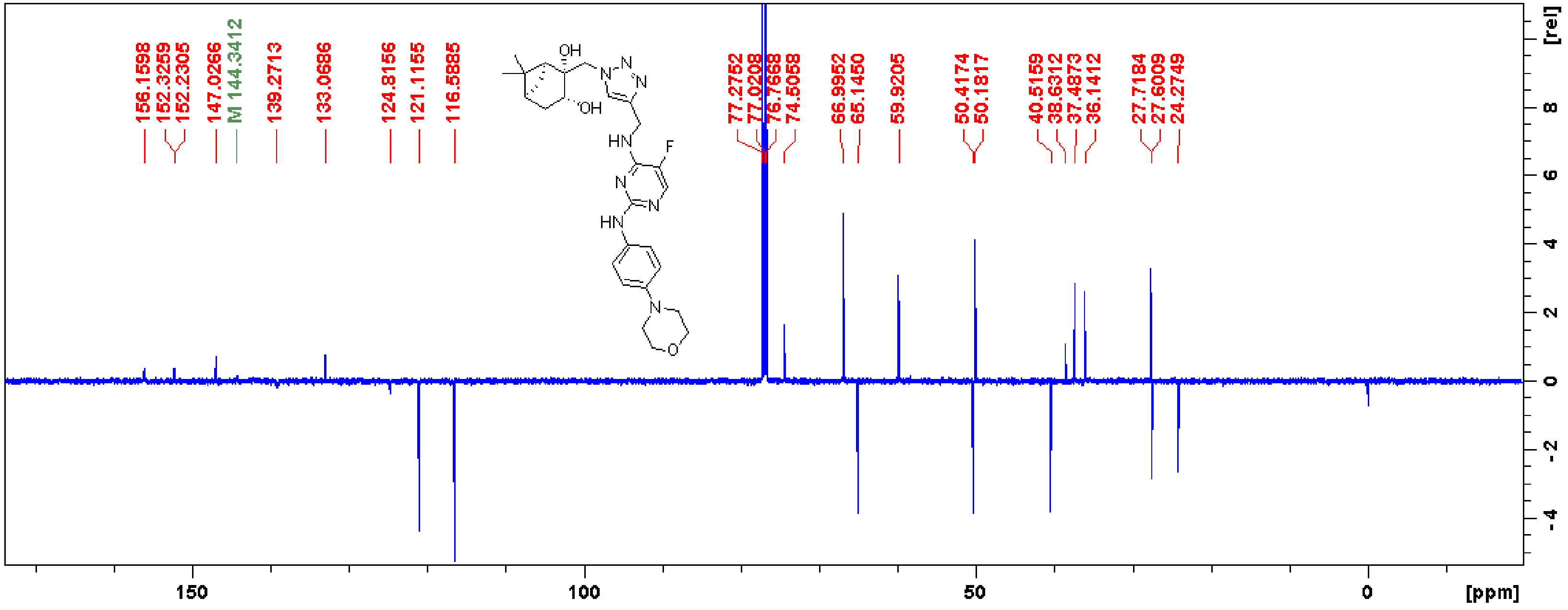

Figure S 114. COSY-NMR of compound **(+)-28**

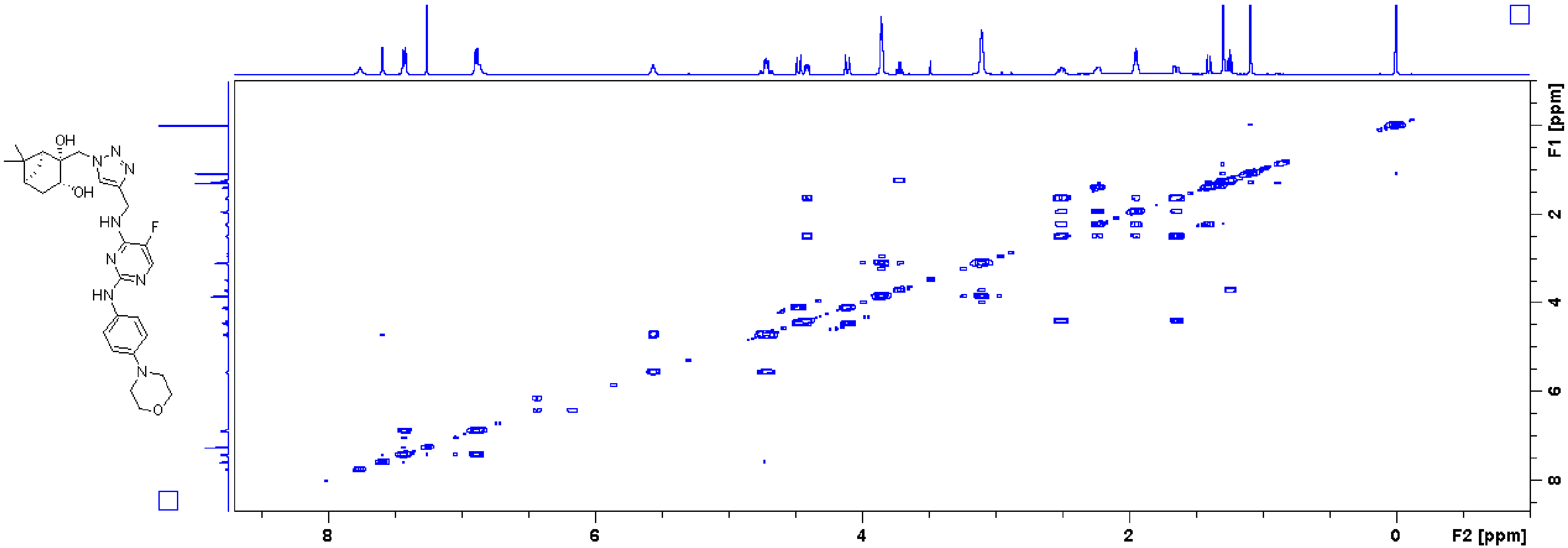

Figure S 115. NOESY-NMR of compound **(+)-28**

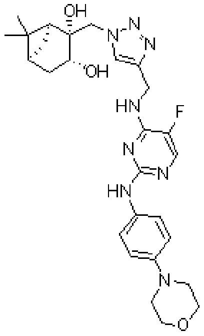

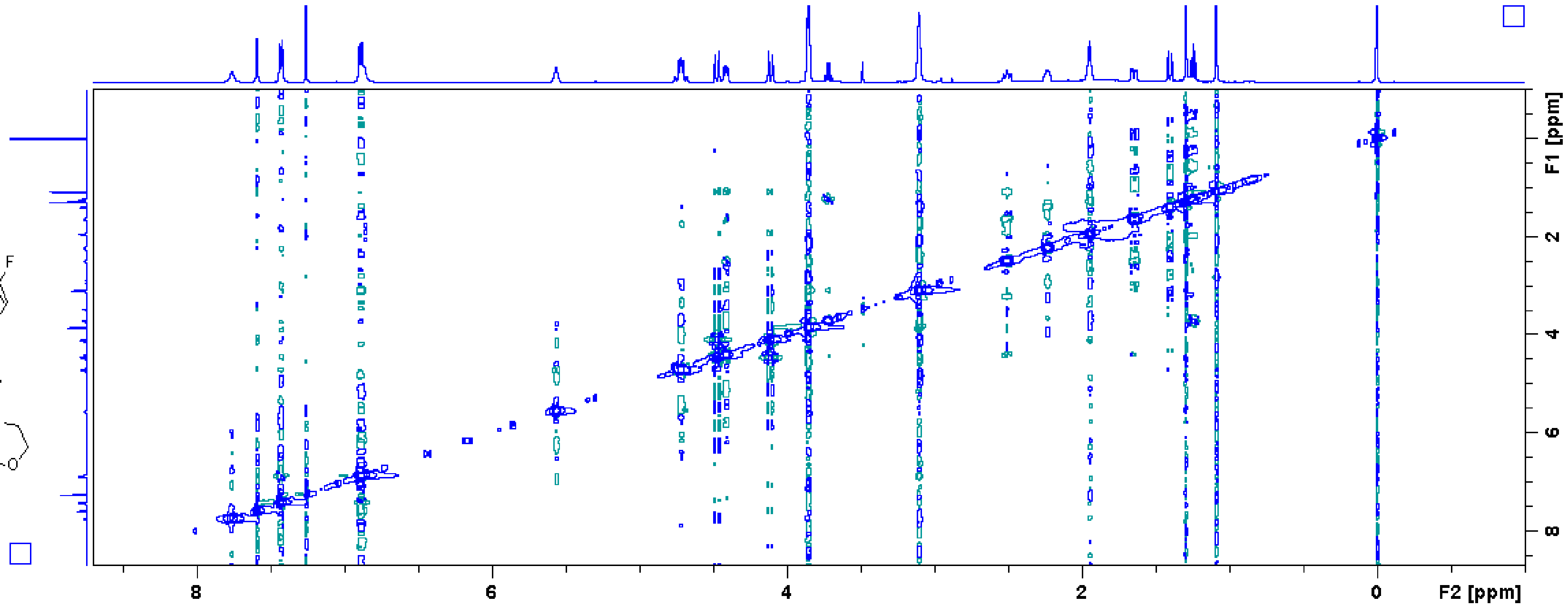

Figure S 116. HSQC-NMR of compound **(+)-28**

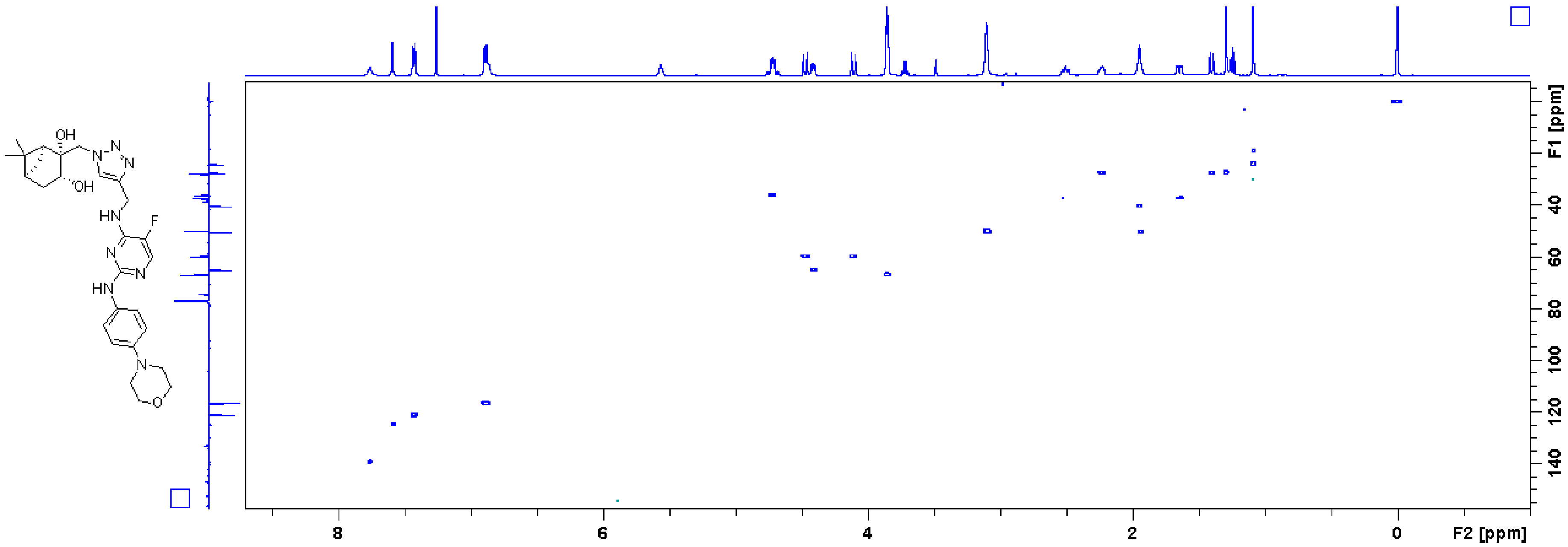

Figure S 117. HMBC-NMR of compound **(+)-28**

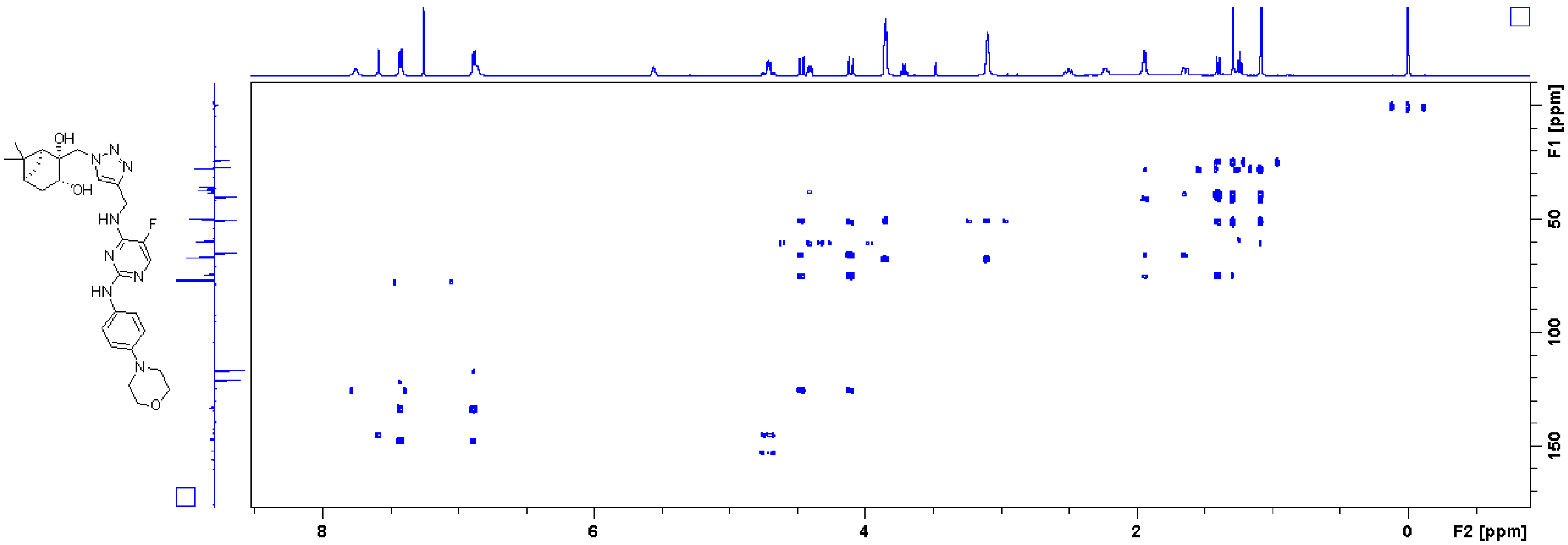

Figure S 118. ^{19}F-NMR of compound **(+)-28**

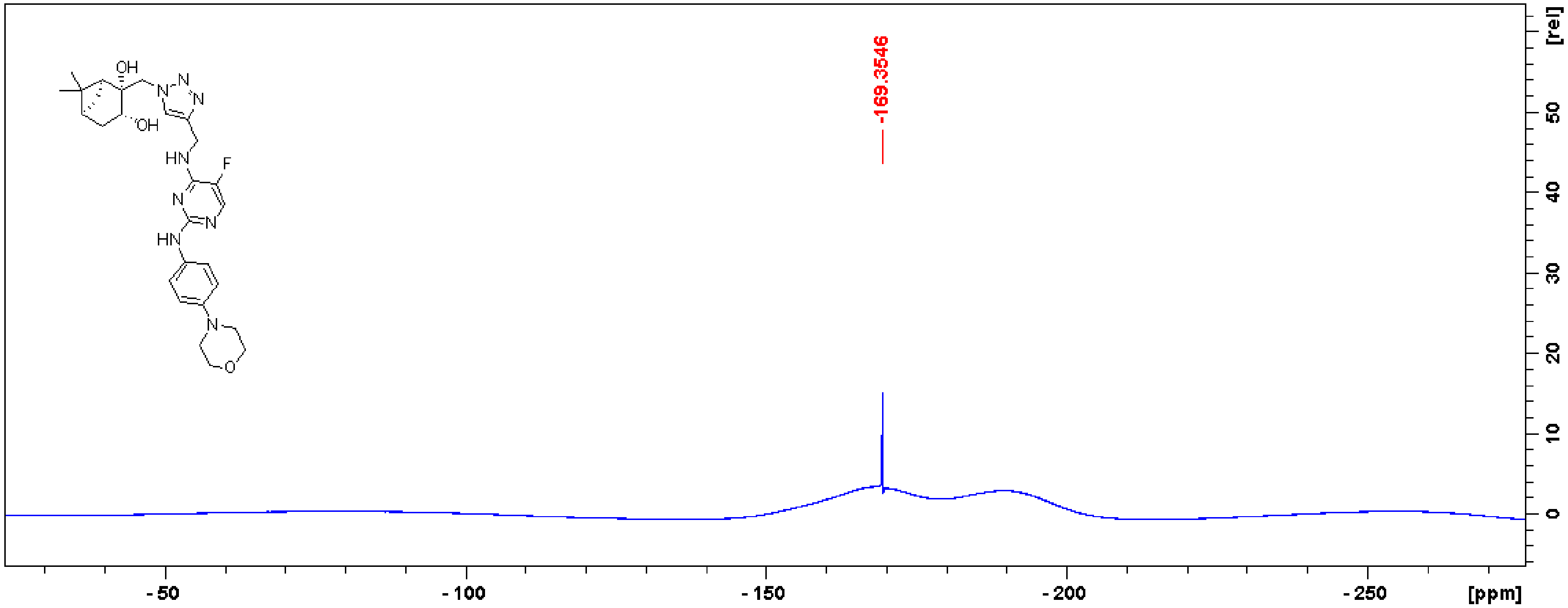

(1*R*,2*S*,3*S*,5*R*)-2-((4-(((5-Fluoro-2-((4-morpholinophenyl)amino)pyrimidin-4-yl)amino)methyl)-1*H*-1,2,3-triazol-1-yl)methyl)-6,6-dimethylbicyclo[3.1.1]heptane-2,3-diol **(–)-28**

Figure S 119. ^{1}H-NMR of compound **(–)-28**

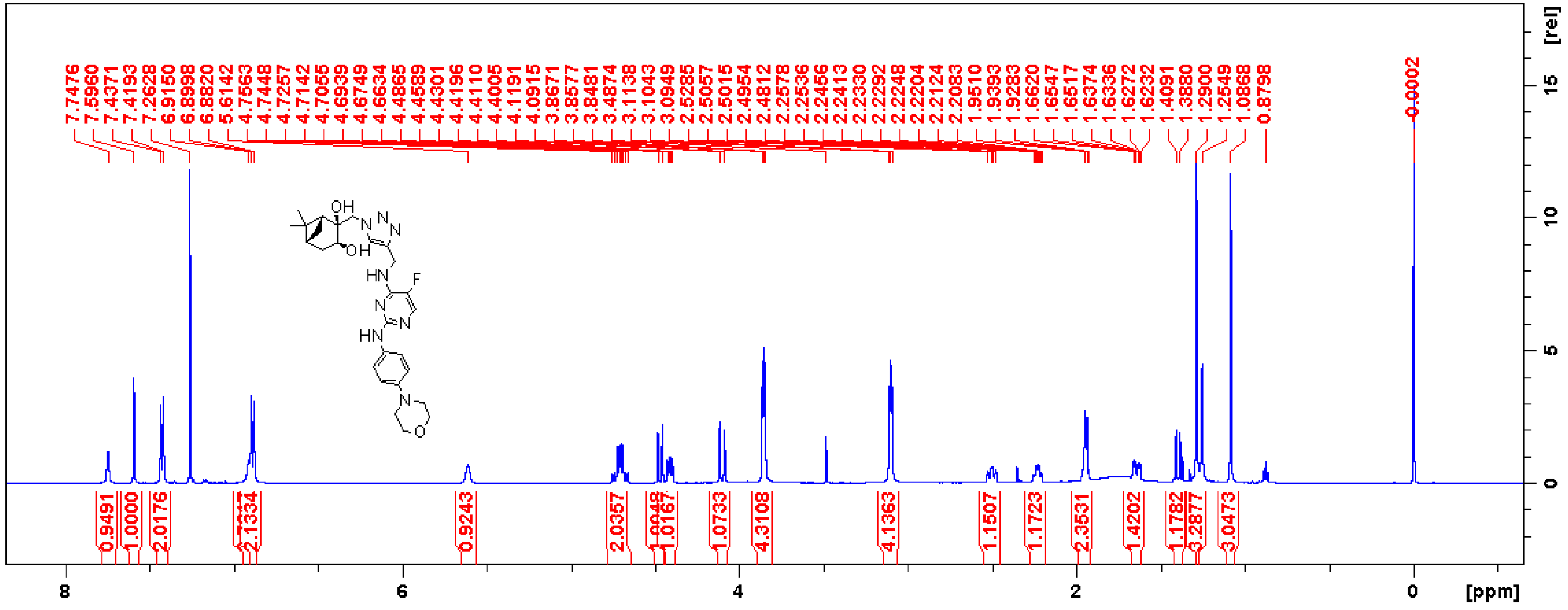

Figure S 120. ^{13}C-NMR of compound **(–)-28**

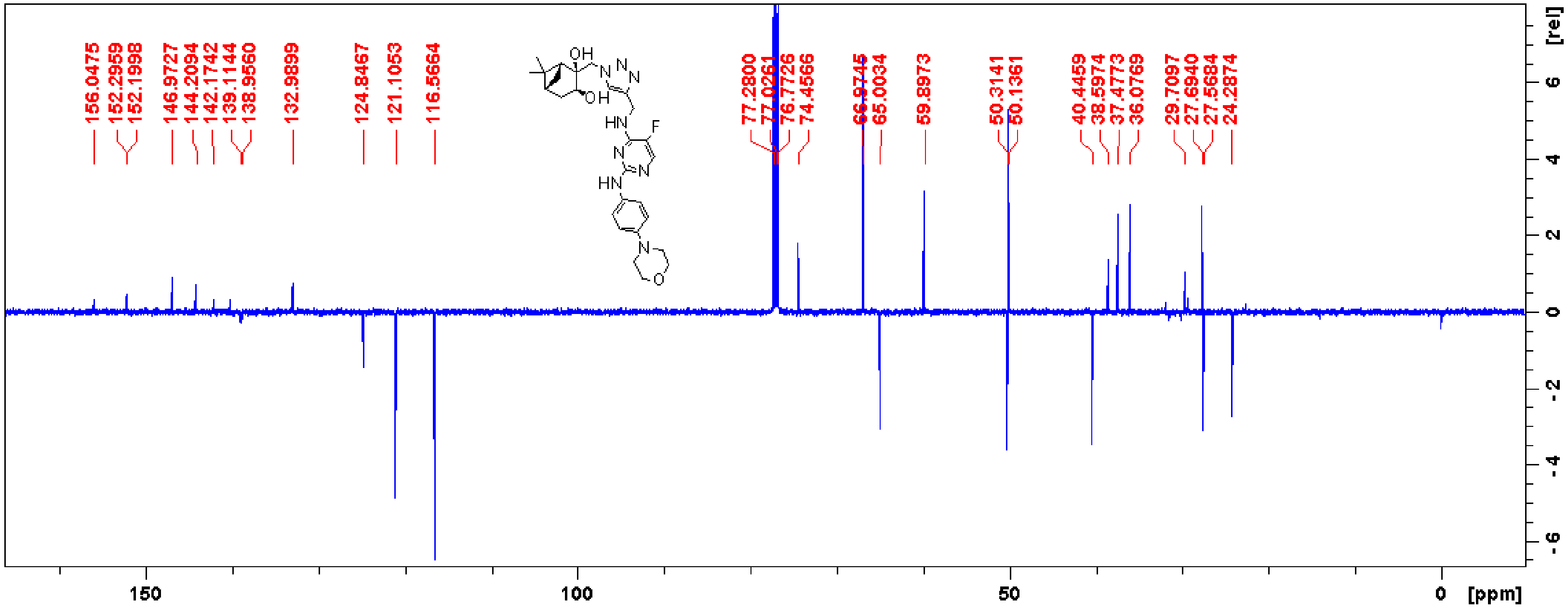

Figure S 121. COSY-NMR of compound **(–)-28**

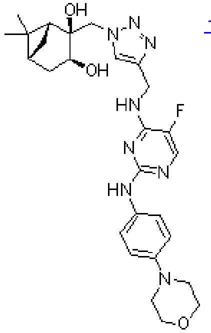

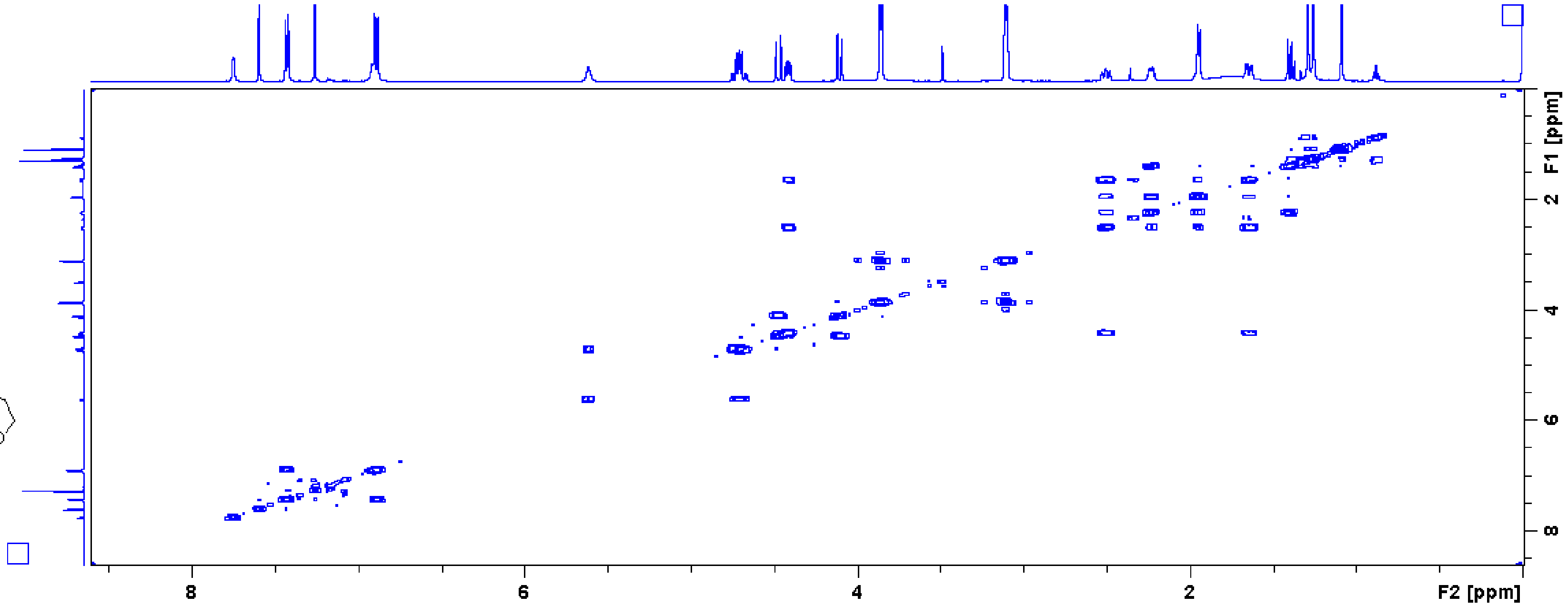

Figure S 122. NOESY-NMR of compound **(–)-28**

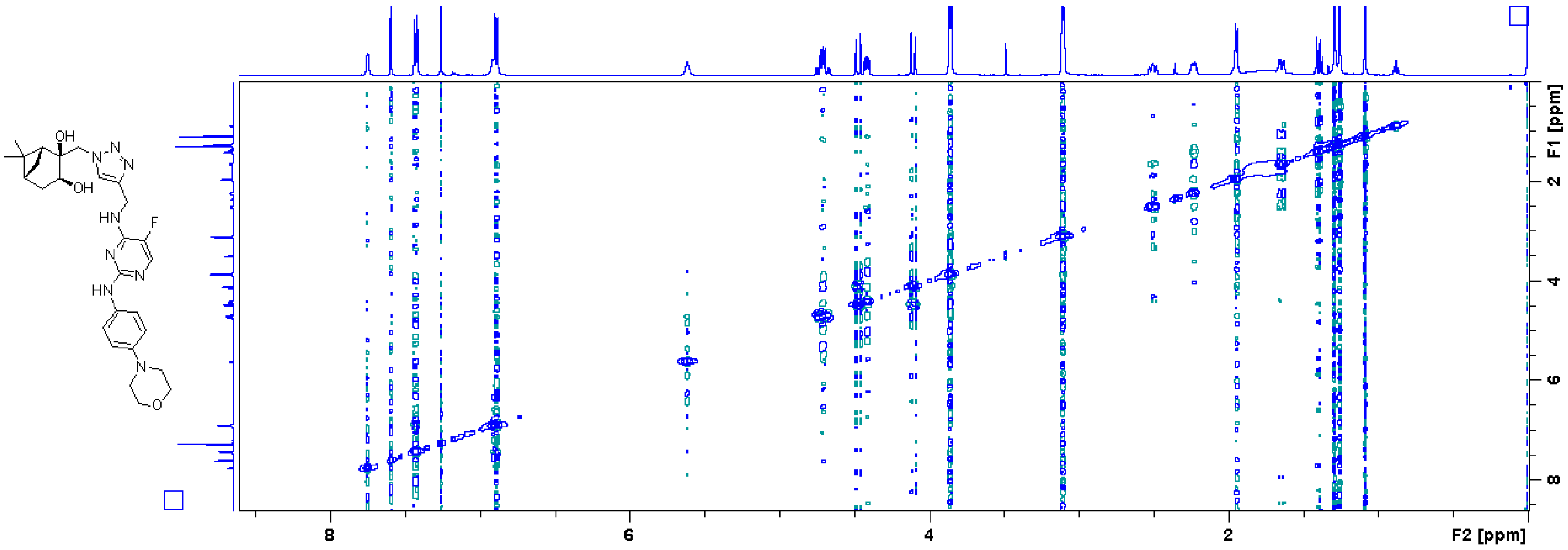

Figure S 123. HSQC-NMR of compound **(–)-28**

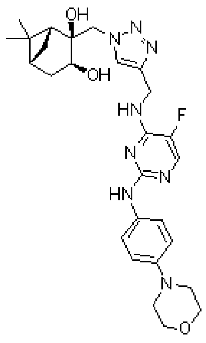

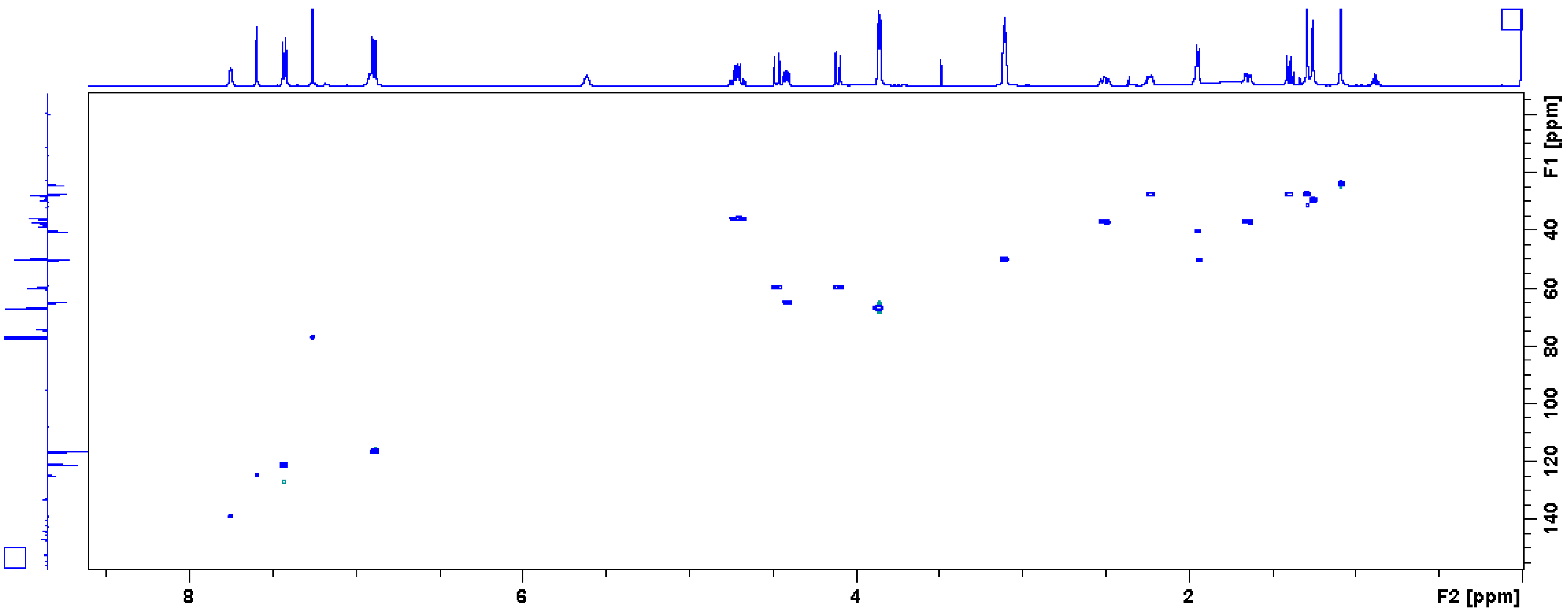

Figure S 124. HMBC-NMR of compound **(–)-28**

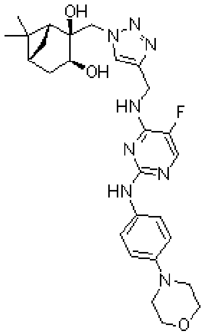

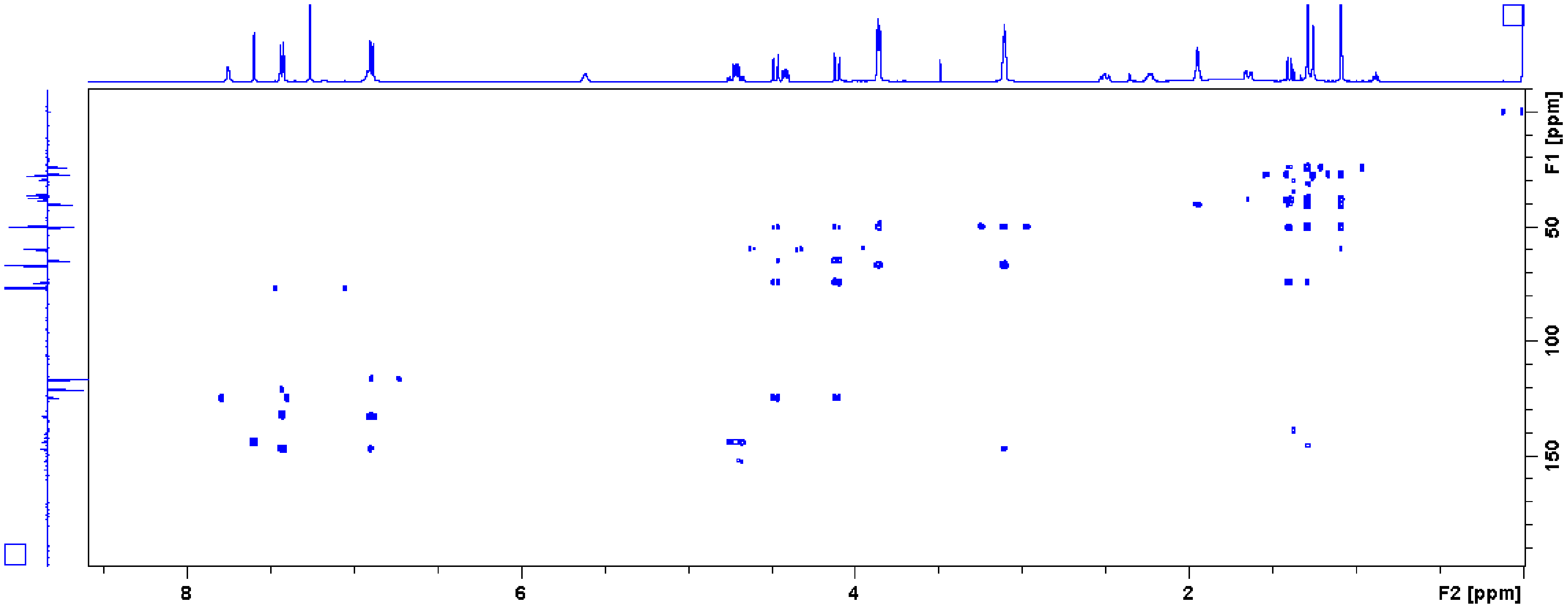

Figure S 125. ^{19}F-NMR of compound **(–)-28**

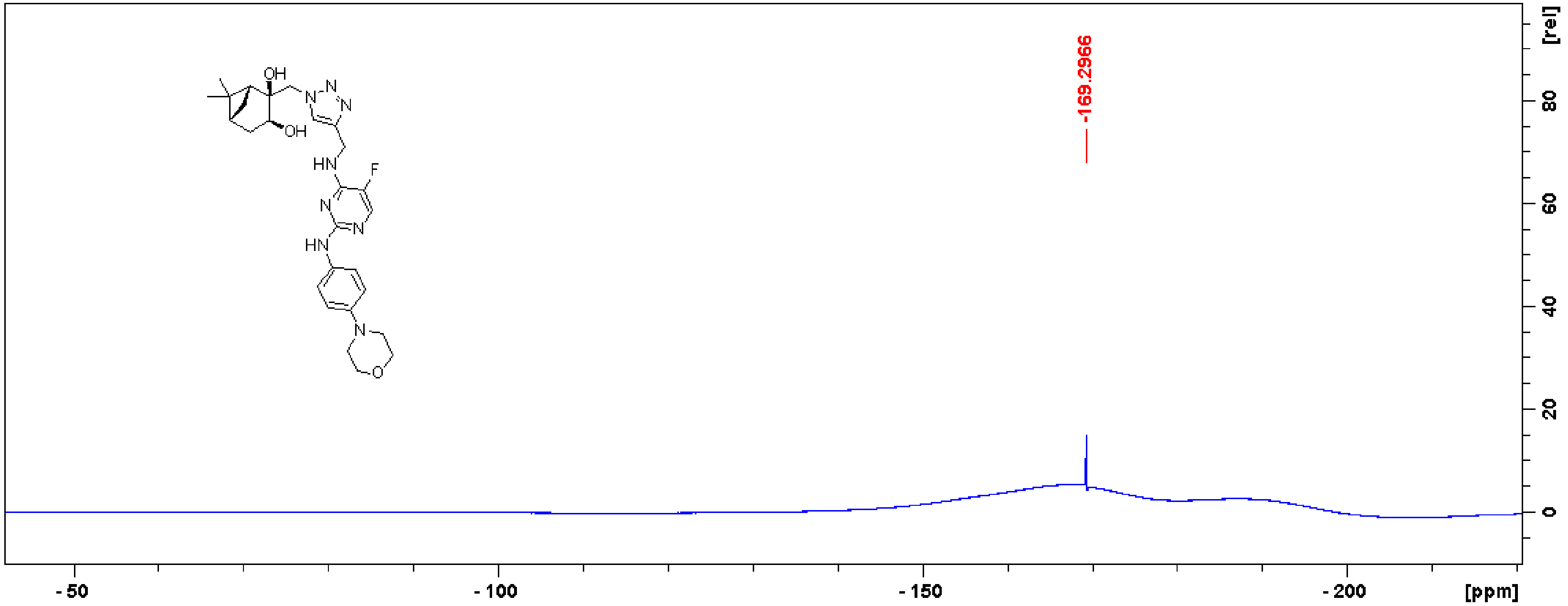

(1*S*,2*R*,3*R*,5*S*)-2-((4-((6-Chloro-9*H*-purin-9-yl)methyl)-1*H*-1,2,3-triazol-1-yl)methyl)-6,6 dimethylbicyclo[3.1.1]heptane-2,3-diol **(+)-29**

Figure S 126. ¹H-NMR of compound **(+)-29**

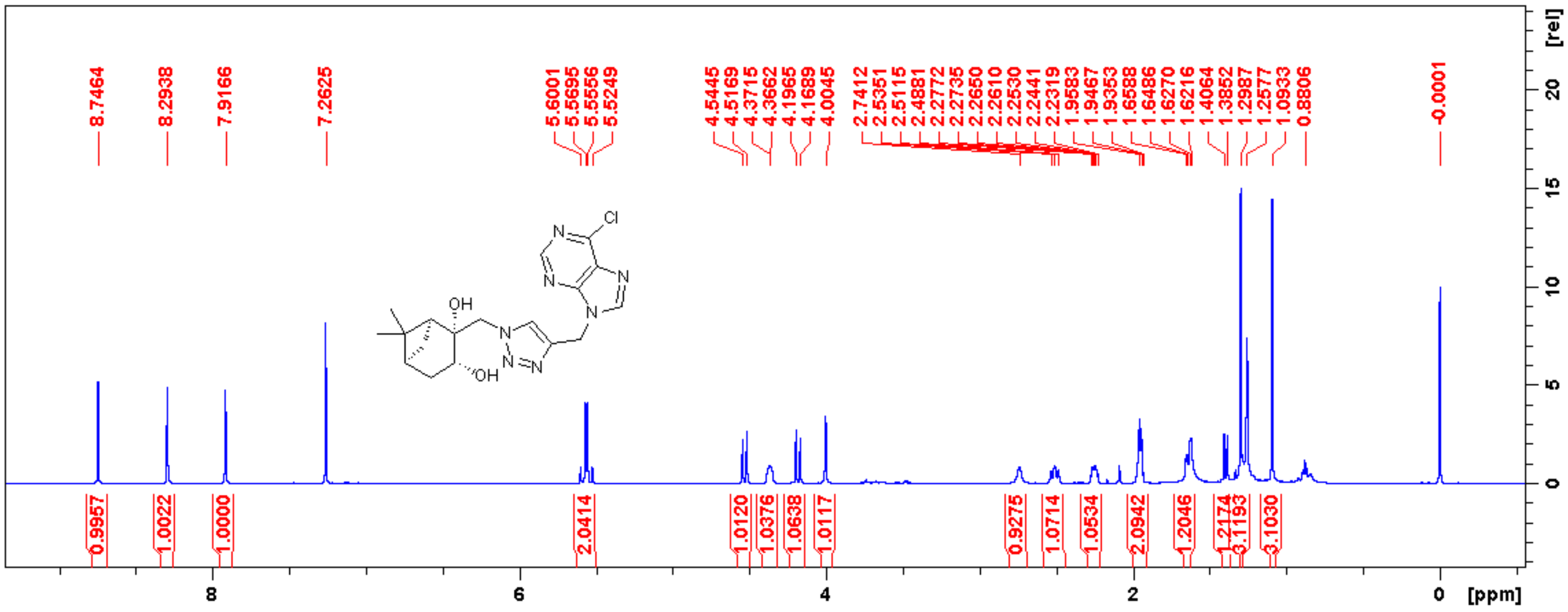

Figure S 127. ^{13}C-NMR of compound **(+)-29**

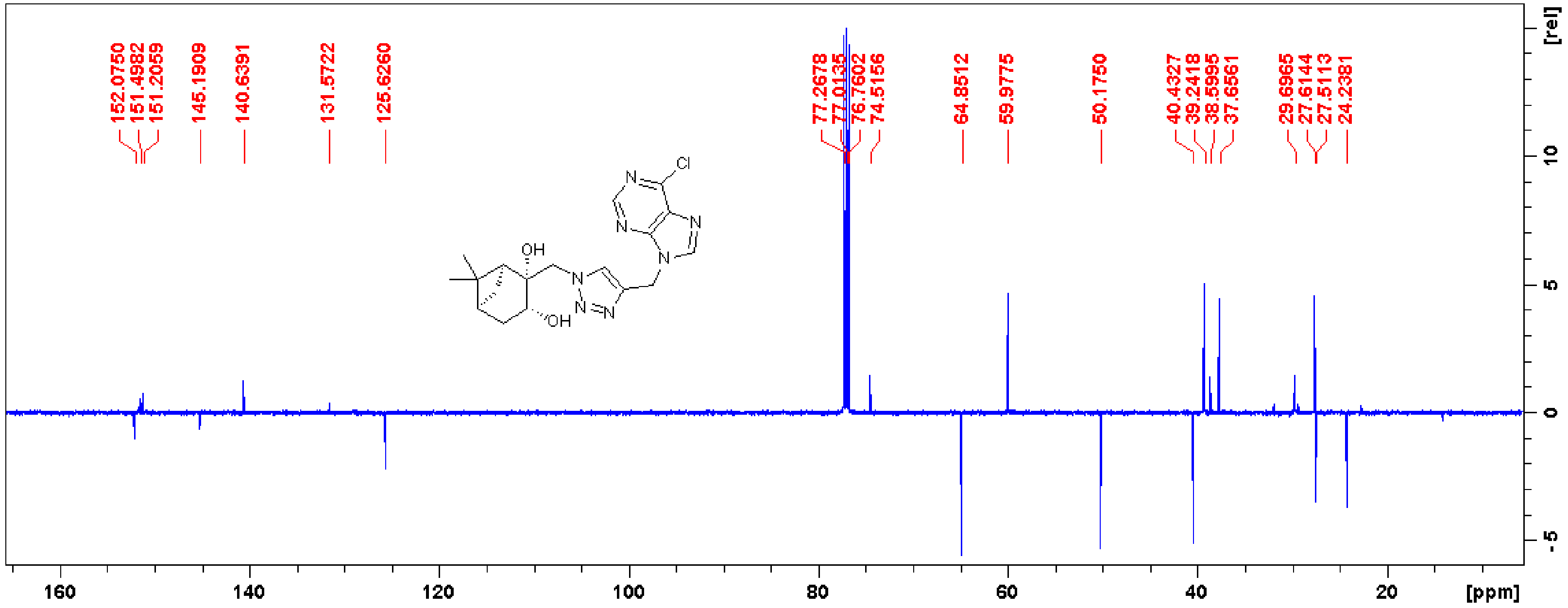

Figure S 128. COSY-NMR of compound **(+)-29**

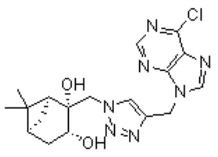

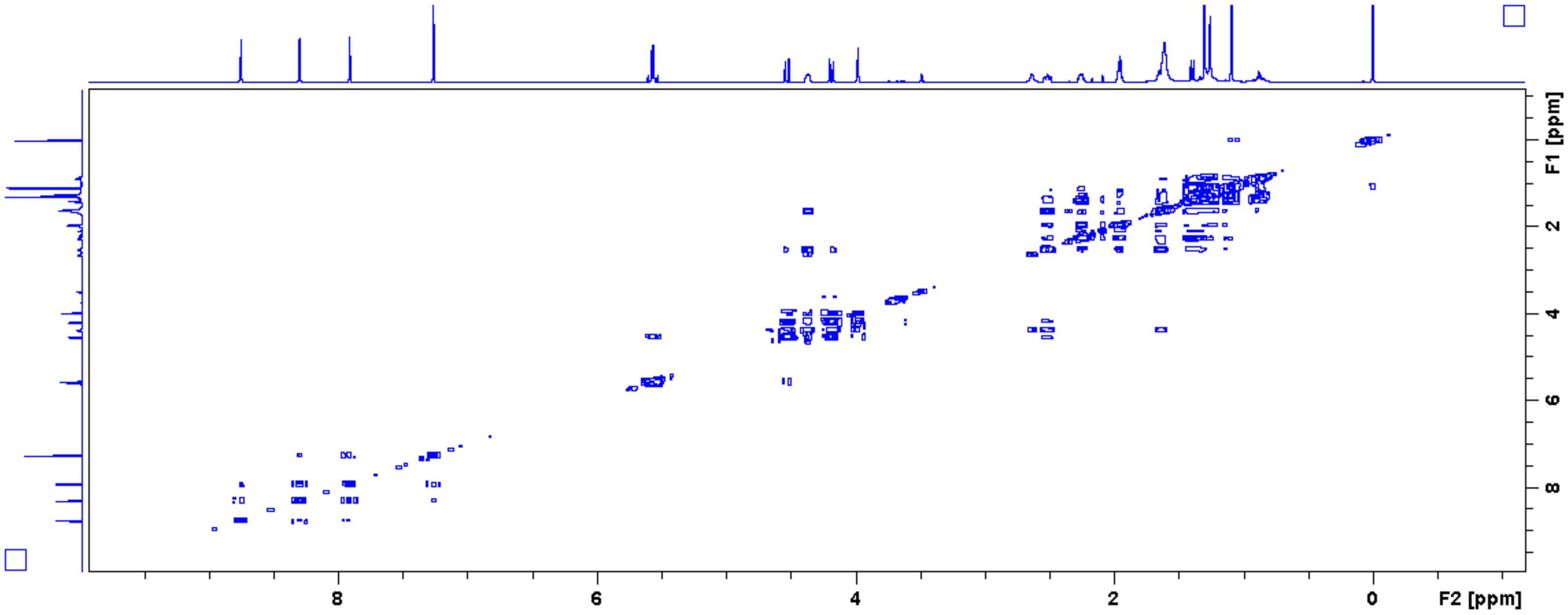

Figure S 129. NOESY-NMR of compound **(+)-29**

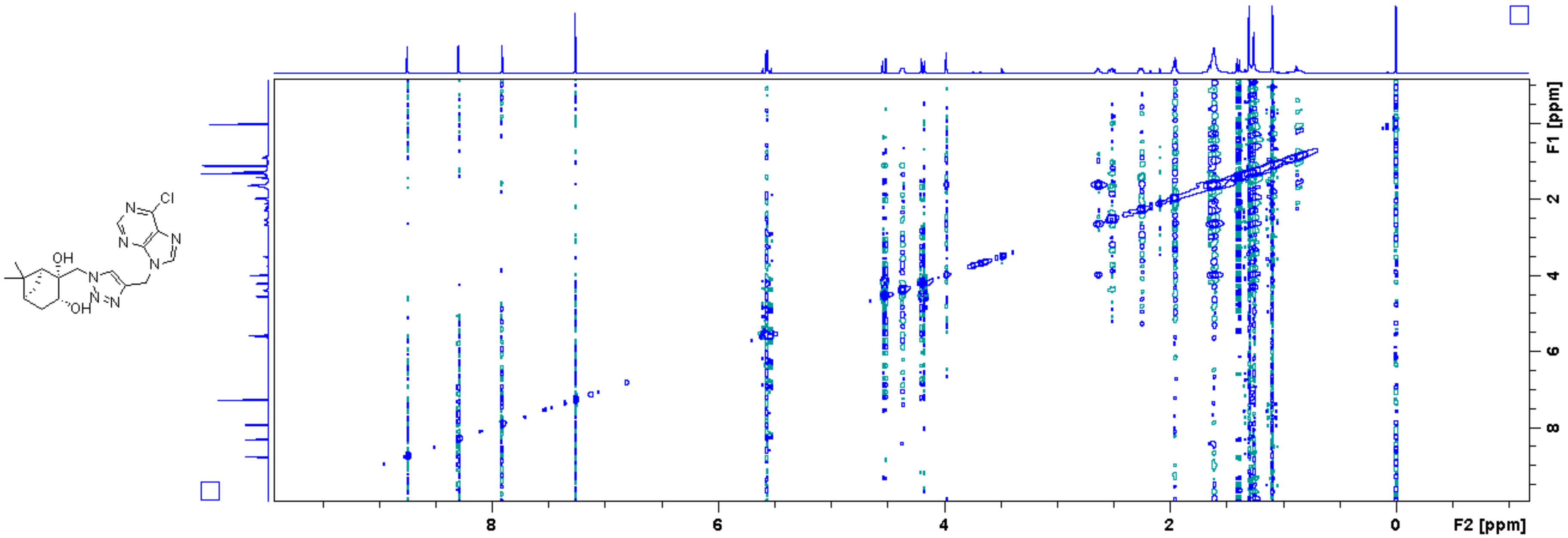

Figure S 130. HSQC-NMR of compound **(+)-29**

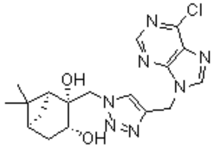

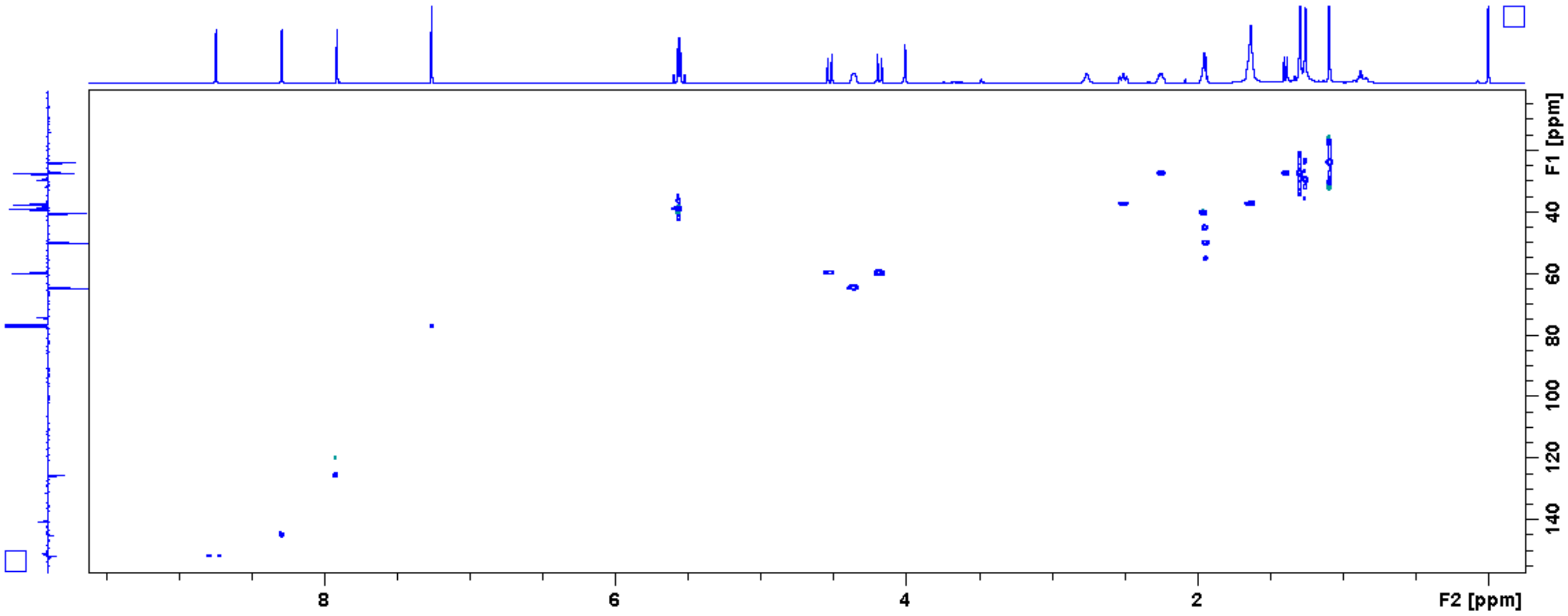

Figure S 131. HMBC-NMR of compound **(+)-29**

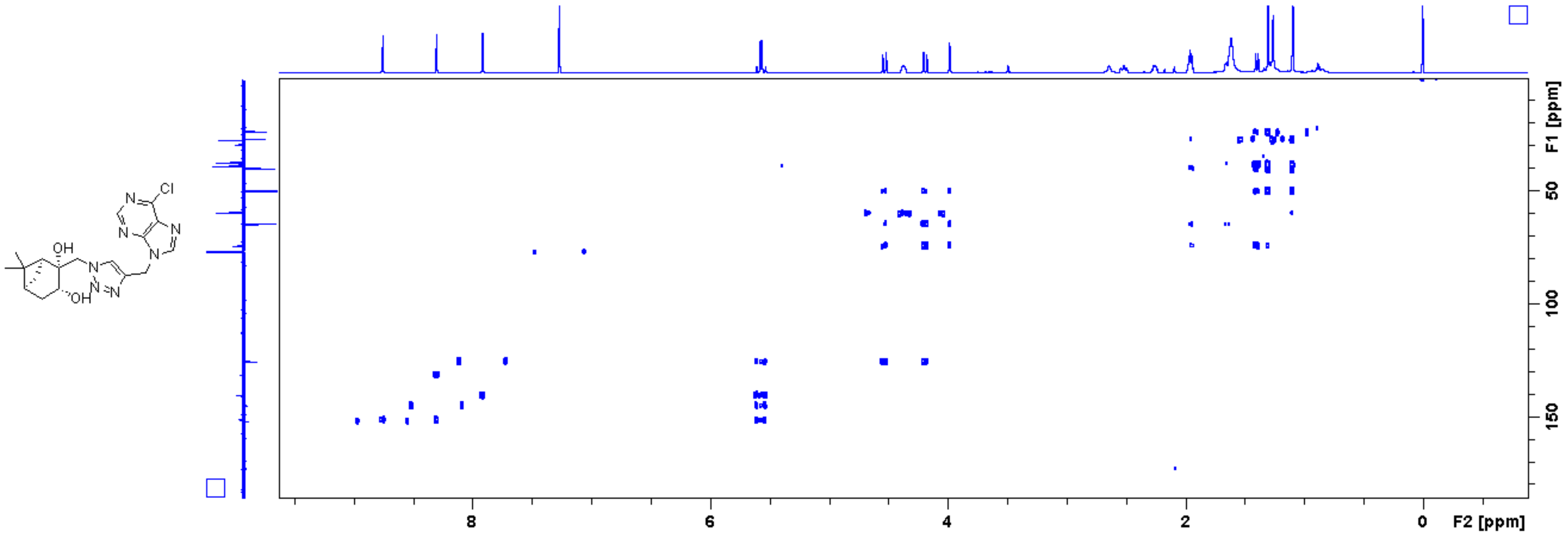

(1*R*,2*S*,3*S*,5*R*)-2-((4-((6-Chloro-9*H*-purin-9-yl)methyl)-1*H*-1,2,3-triazol-1-yl)methyl)-6,6-dimethylbicyclo[3.1.1]heptane-2,3-diol **(–)-29**

Figure S 132. ^{1}H-NMR of compound **(–)-29**

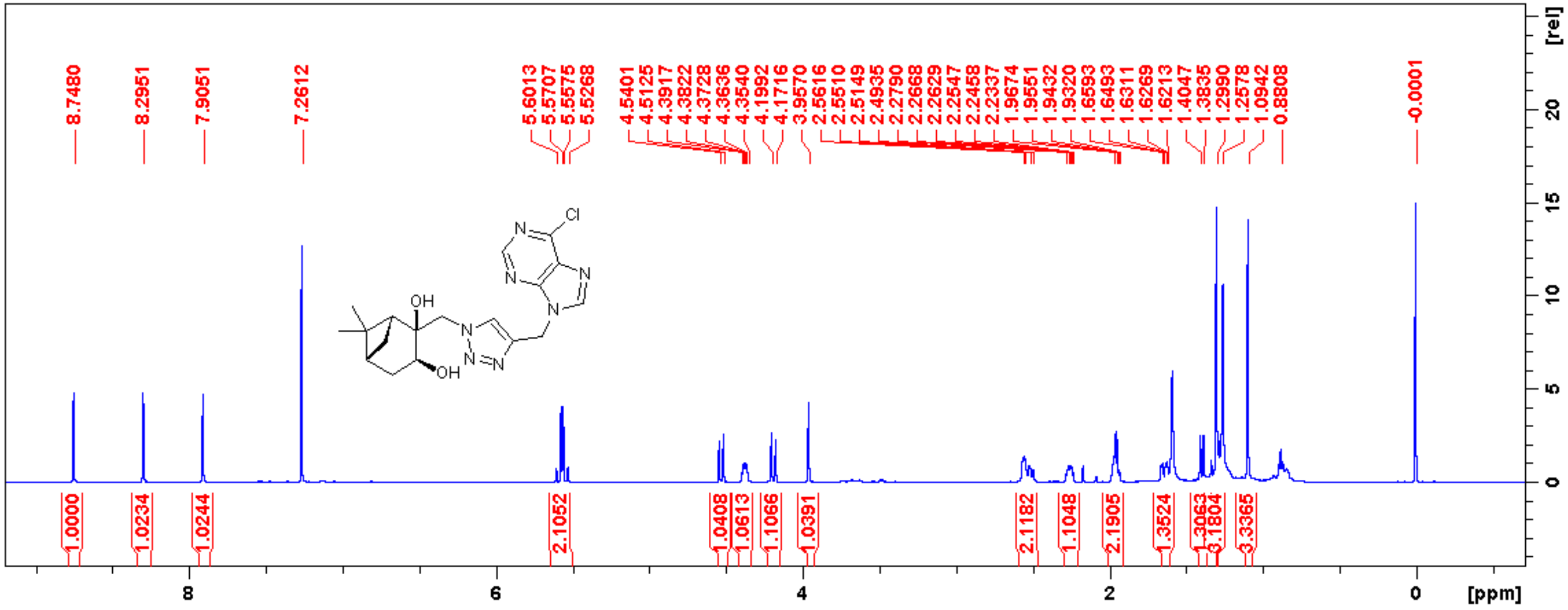

Figure S 133. ^{13}C-NMR of compound **(–)-29**

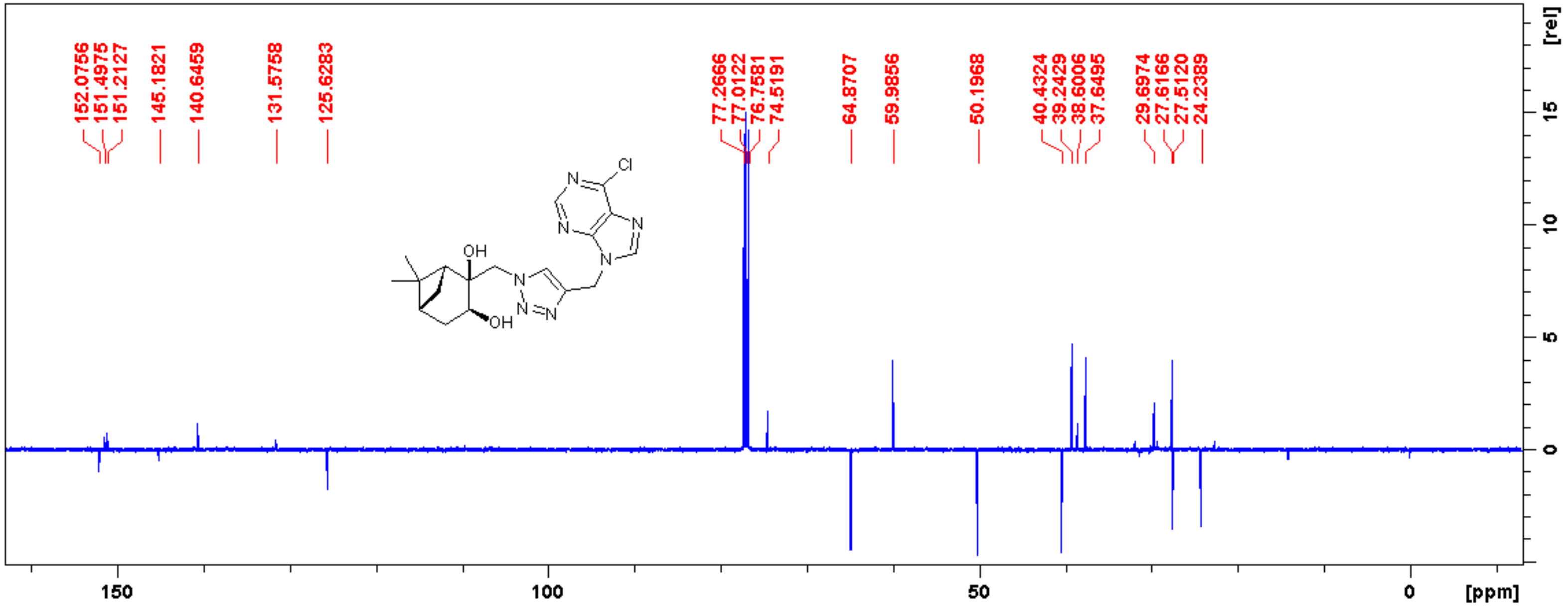

Figure S 134. COSY-NMR of compound **(–)-29**

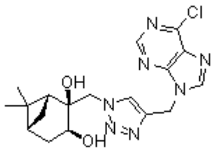

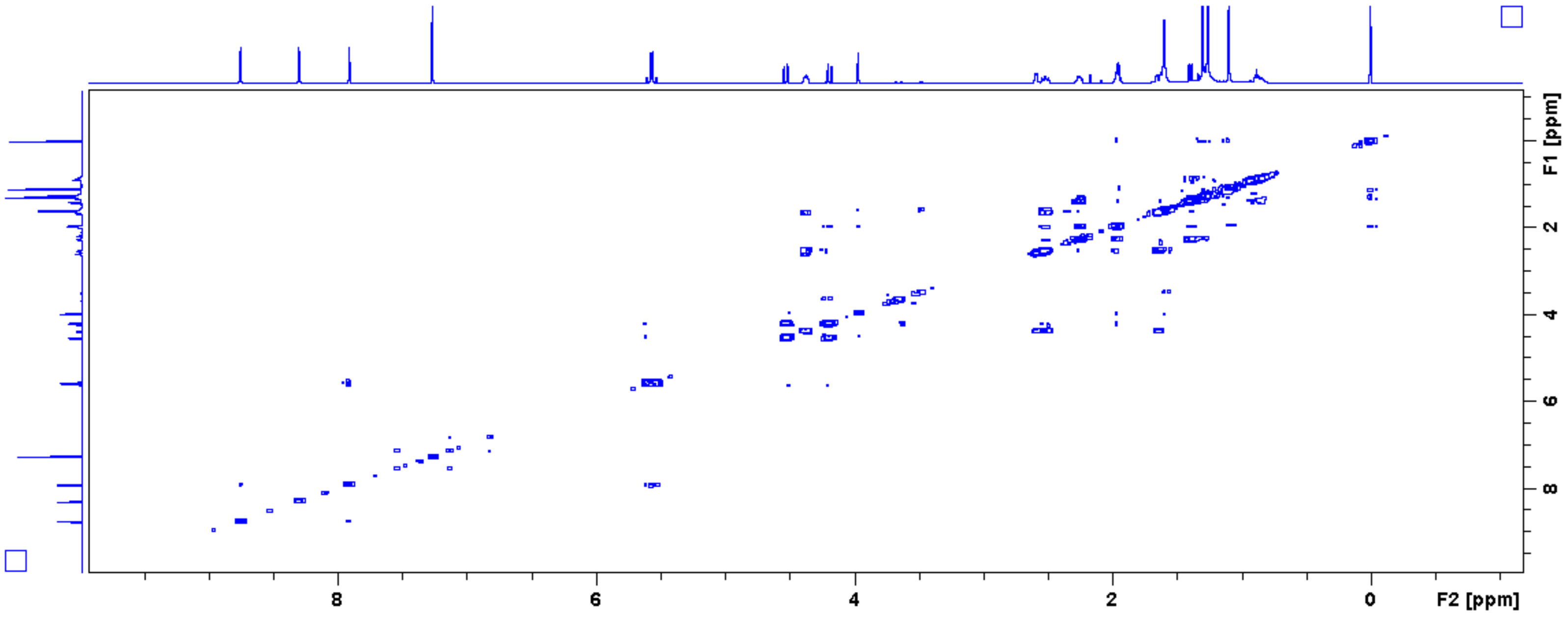

Figure S 135. NOESY-NMR of compound **(–)-29**

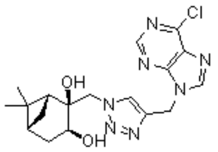

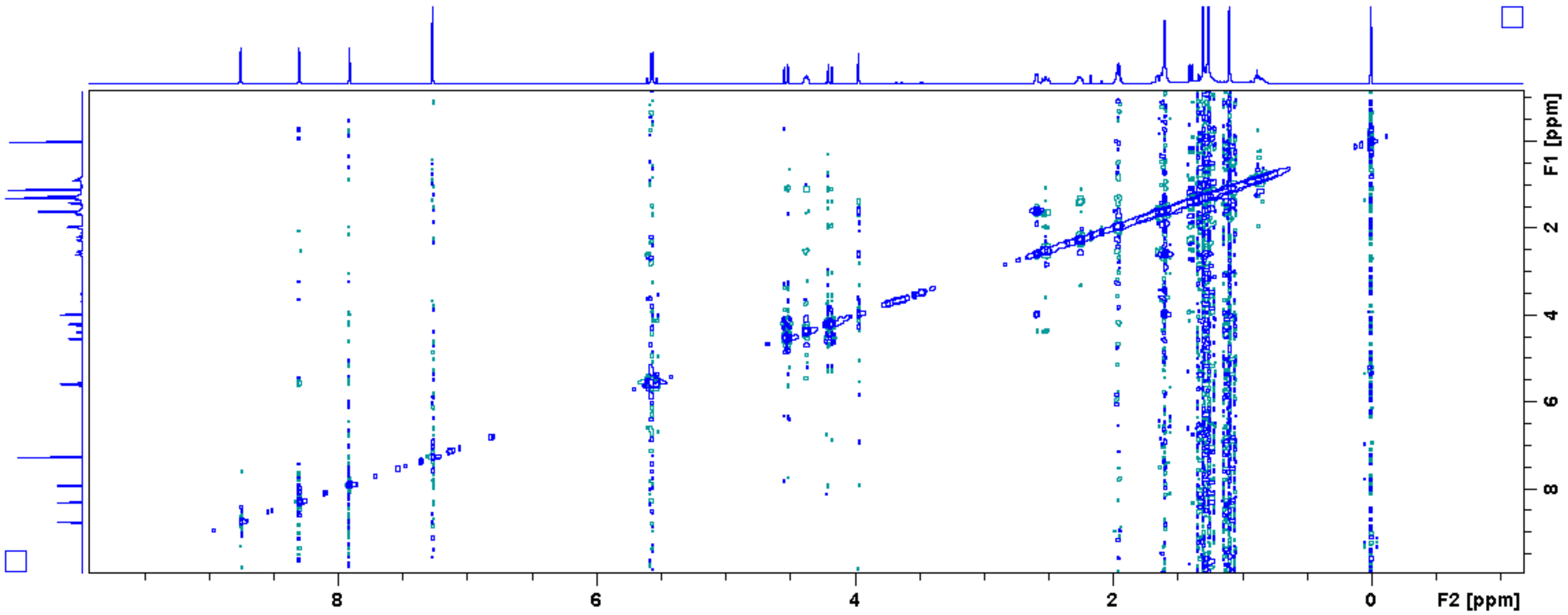

Figure S 136. HSQC-NMR of compound **(–)-29**

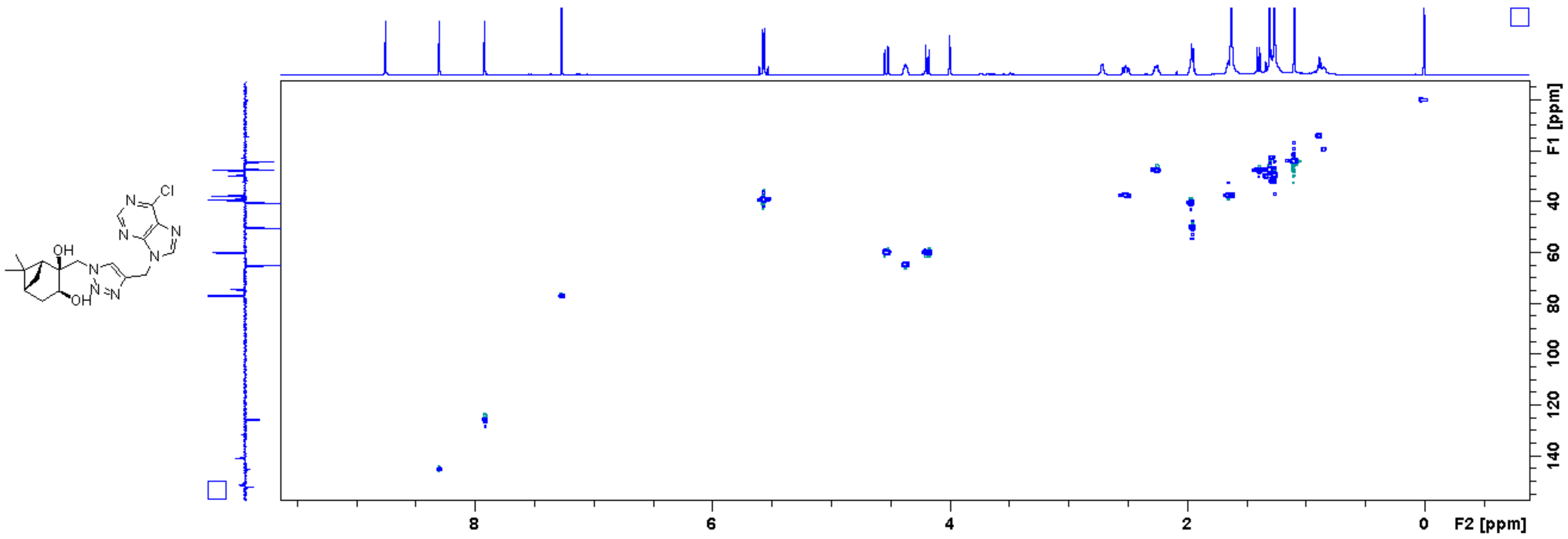

Figure S 137. HMBC-NMR of compound **(–)-29**

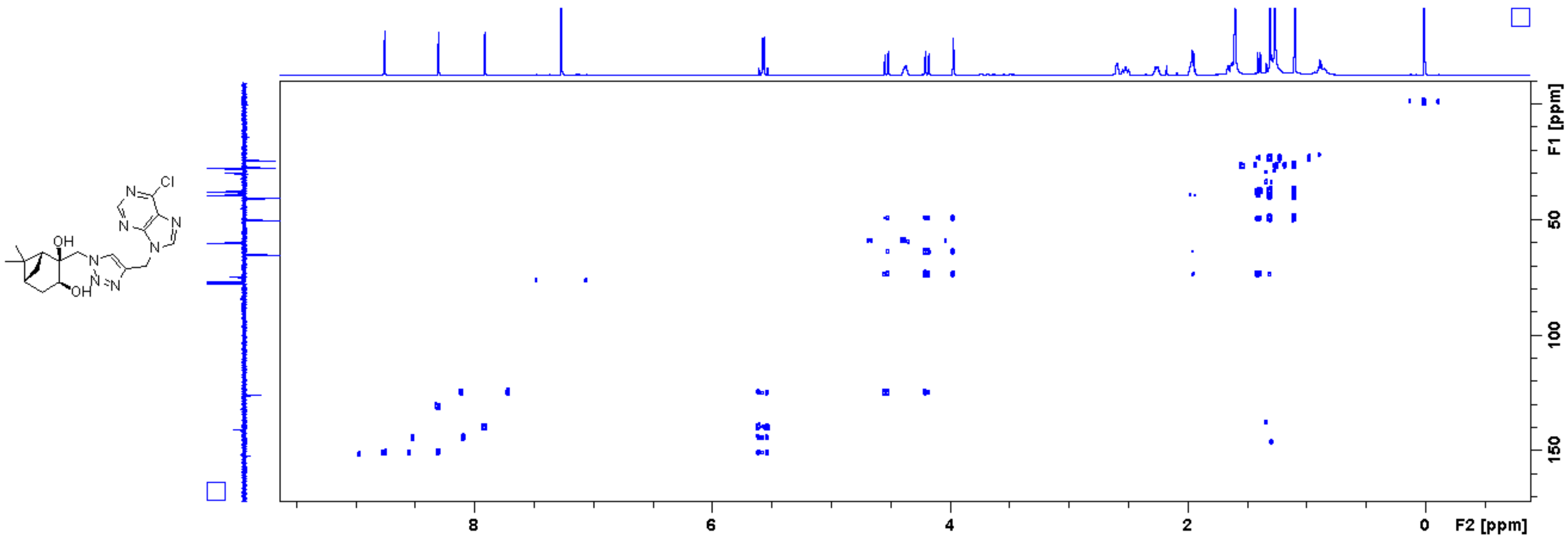

(1*S*,2*R*,3*R*,5*S*)-2-((4-((6-Chloro-8-phenyl-9*H*-purin-9-yl)methyl)-1*H*-1,2,3-triazol-1-yl)methyl)-6,6-dimethylbicyclo[3.1.1]heptane-2,3-diol **(+)-30**

Figure S 138. ^{1}H-NMR of compound **(+)-30**

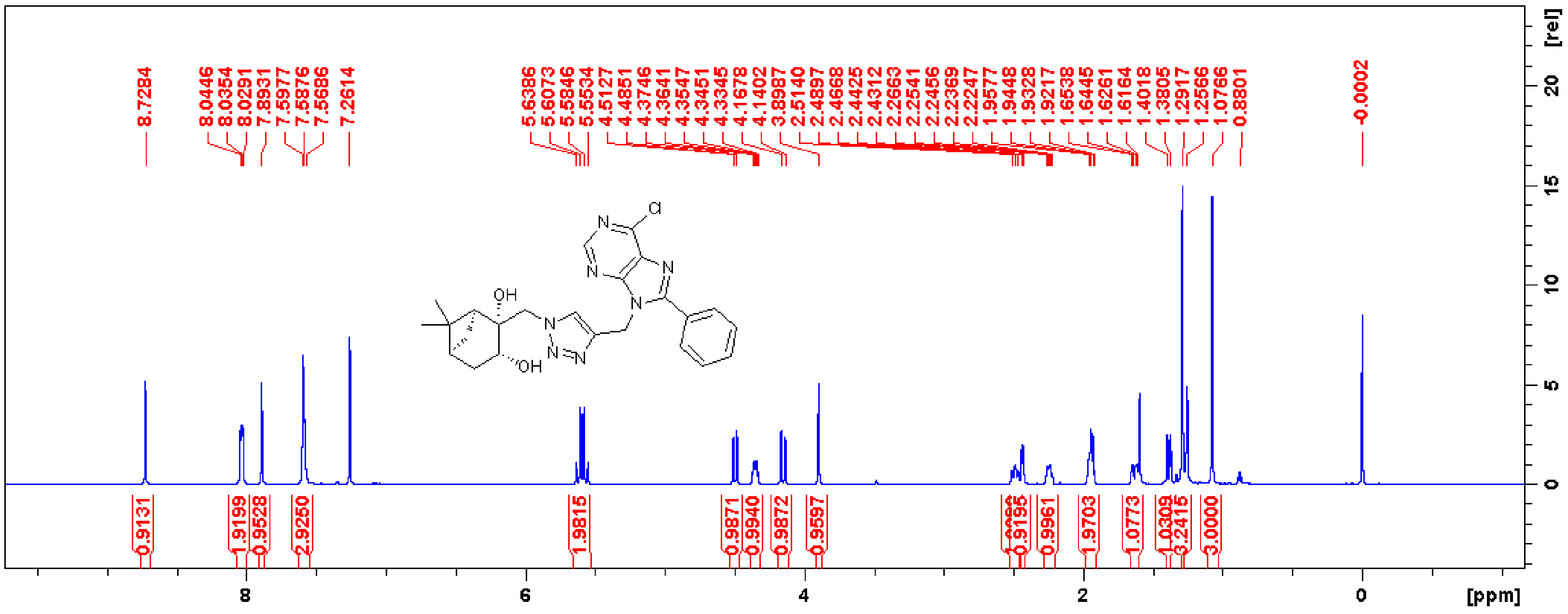

Figure S 139. ^{13}C-NMR of compound **(+)-30**

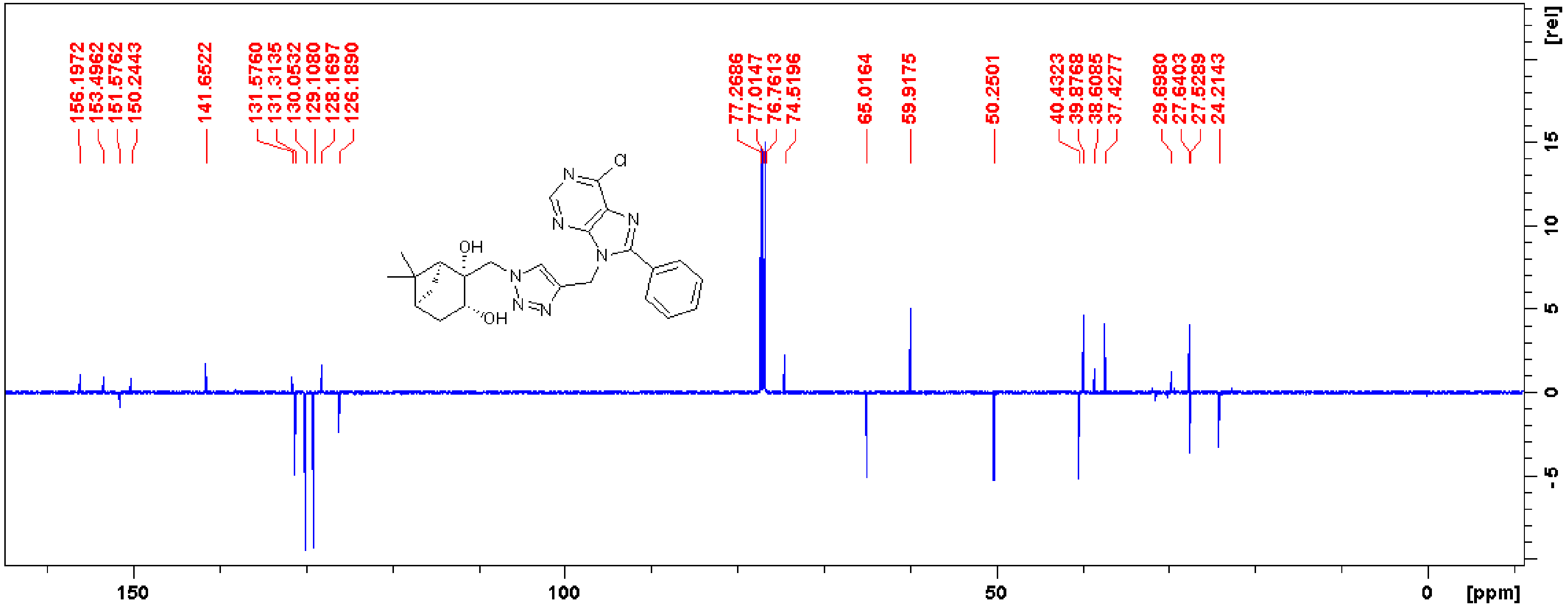

Figure S 140. COSY-NMR of compound **(+)-30**

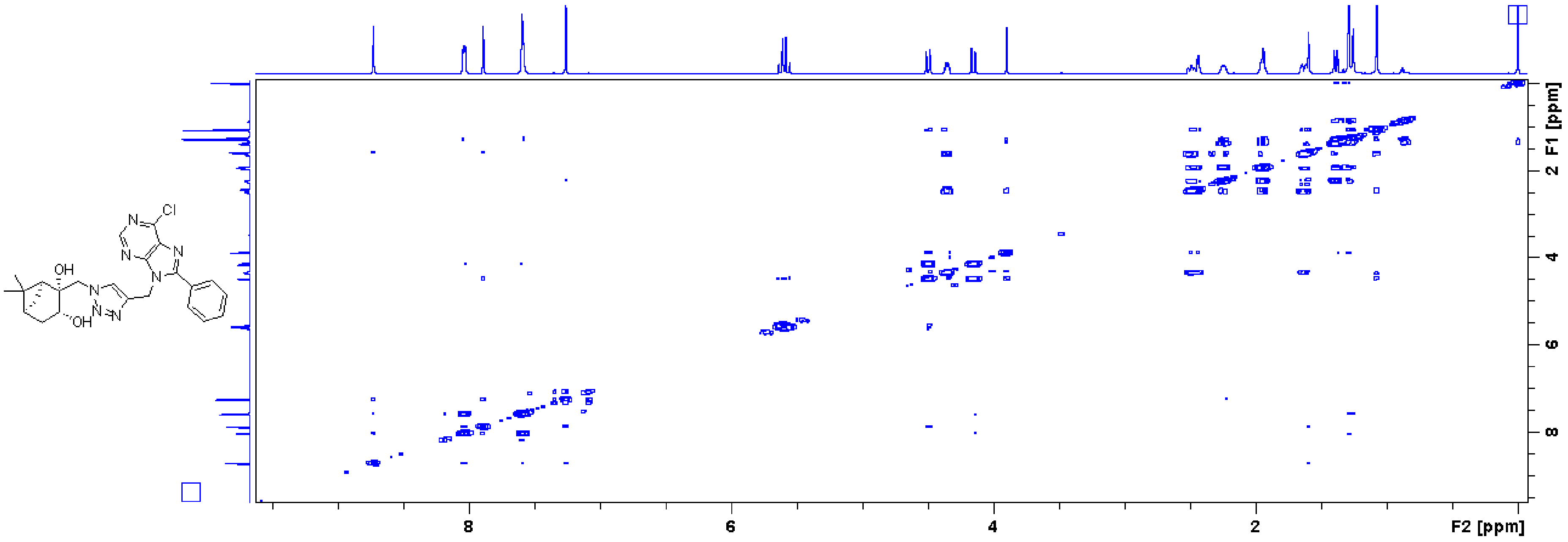

Figure S 141. NOESY-NMR of compound **(+)-30**

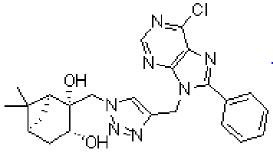

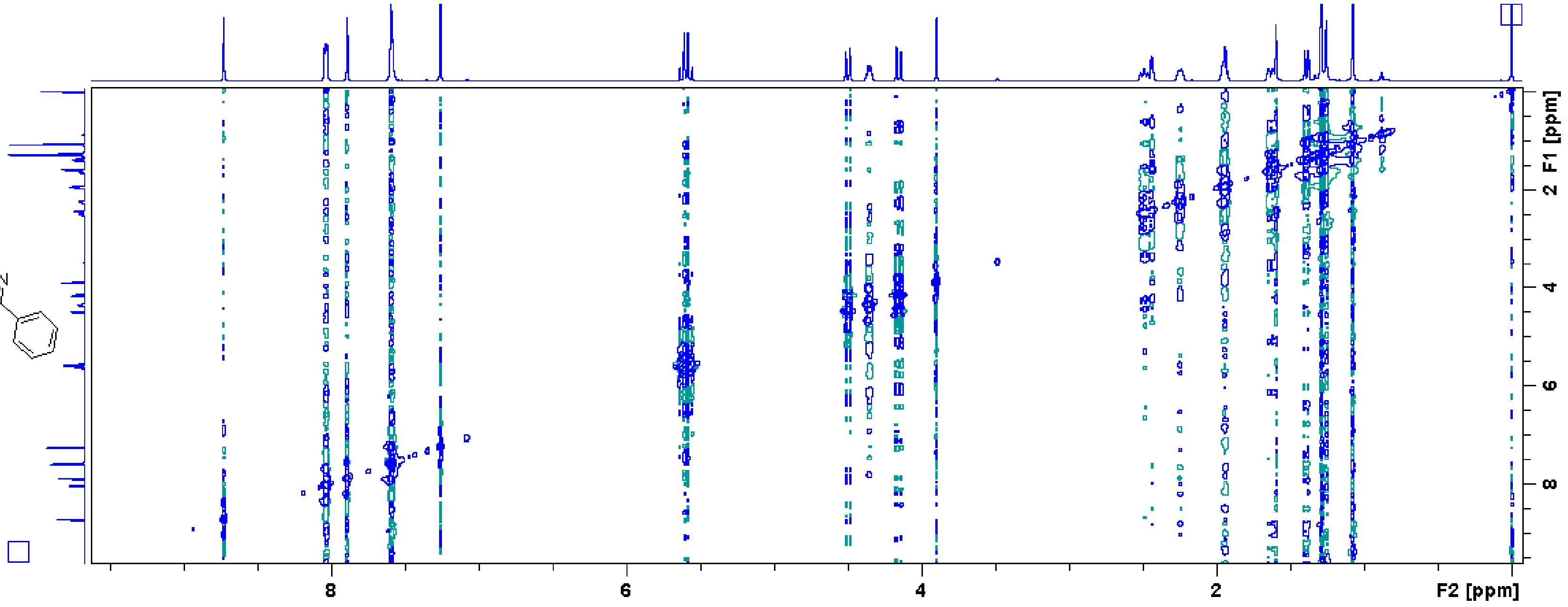

Figure S 142. HSQC-NMR of compound **(+)-30**

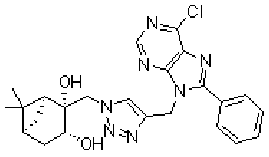

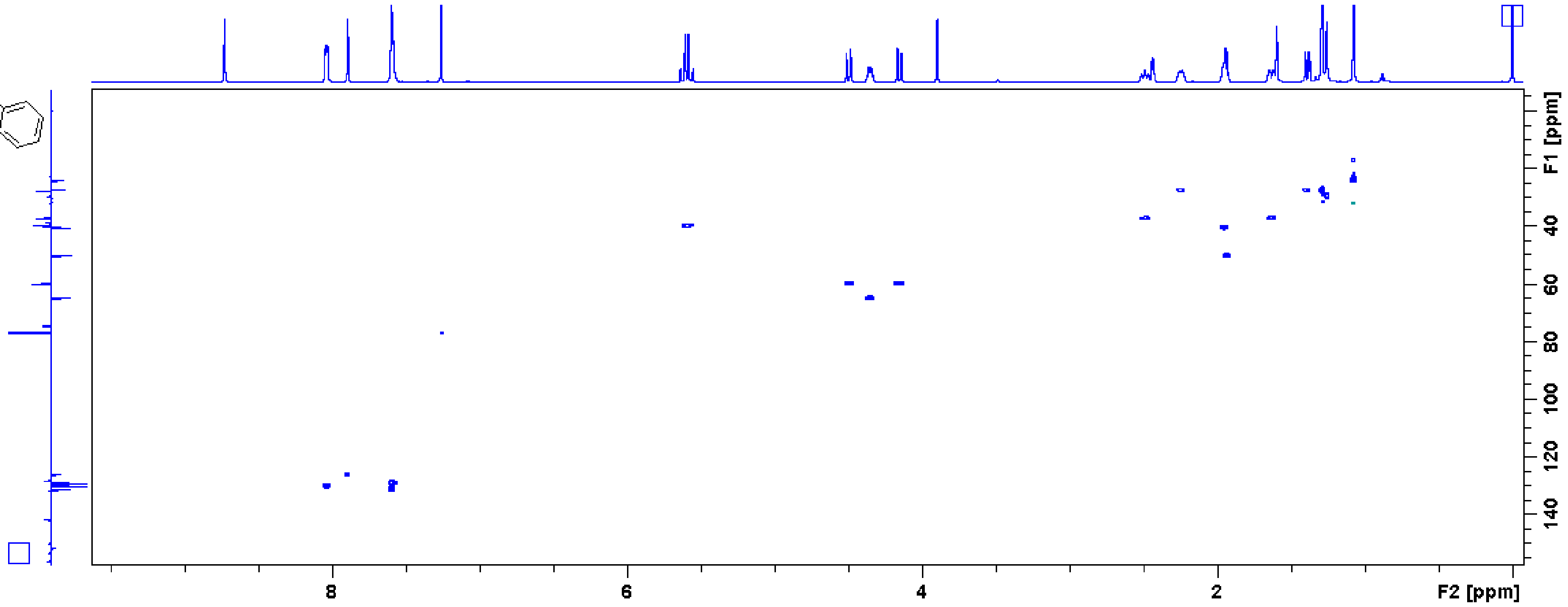

Figure S 143. HMBC-NMR of compound **(+)-30**

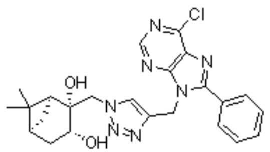

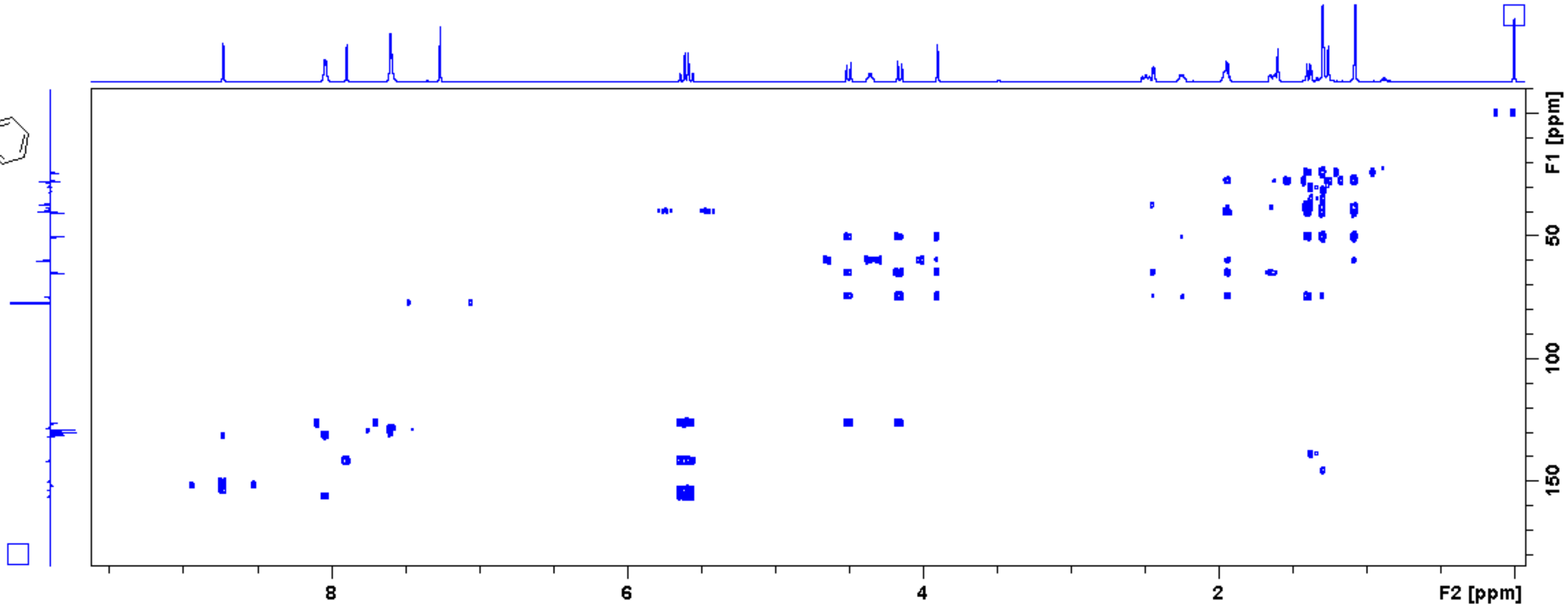

(1*R*,2*S*,3*S*,5*R*)-2-((4-((6-Chloro-8-phenyl-9*H*-purin-9-yl)methyl)-1*H*-1,2,3-triazol-1-yl)methyl)-6,6-dimethylbicyclo[3.1.1]heptane-2,3-diol **(–)-30**

Figure S 144. ^{1}H-NMR of compound **(–)-30**

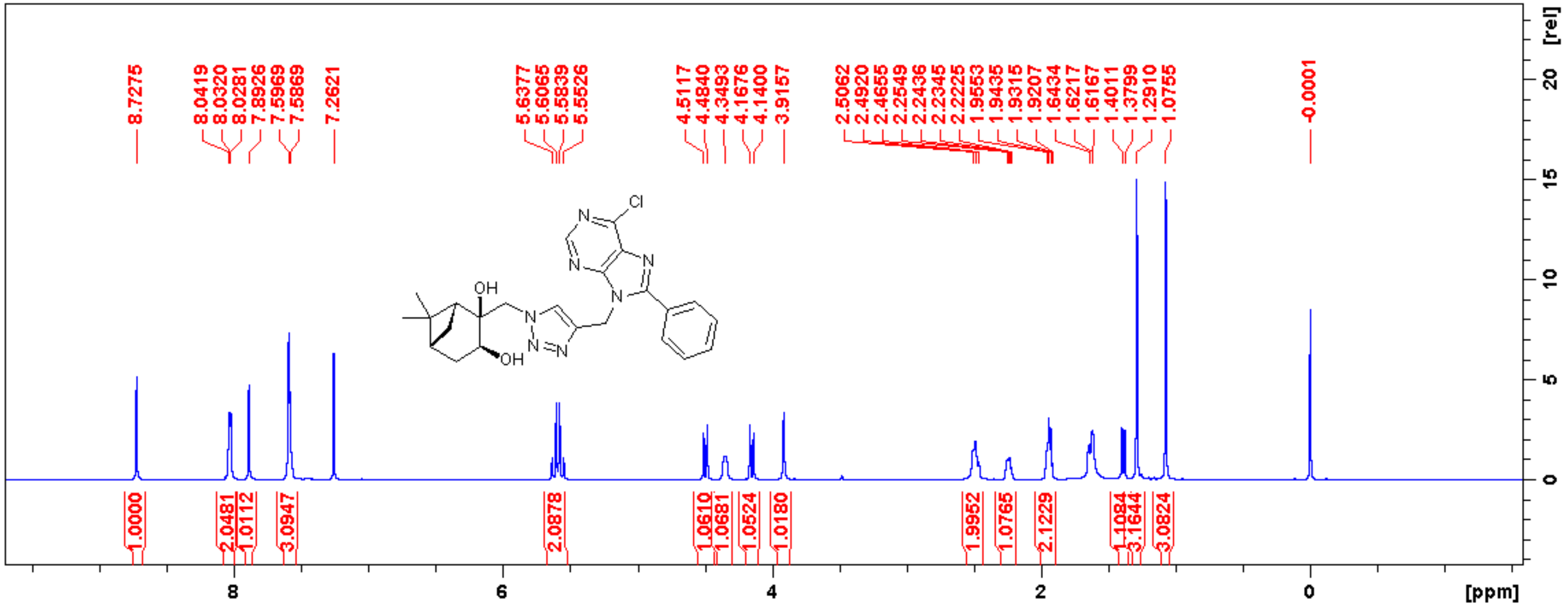

Figure S 145. ^{13}C-NMR of compound **(–)-30**

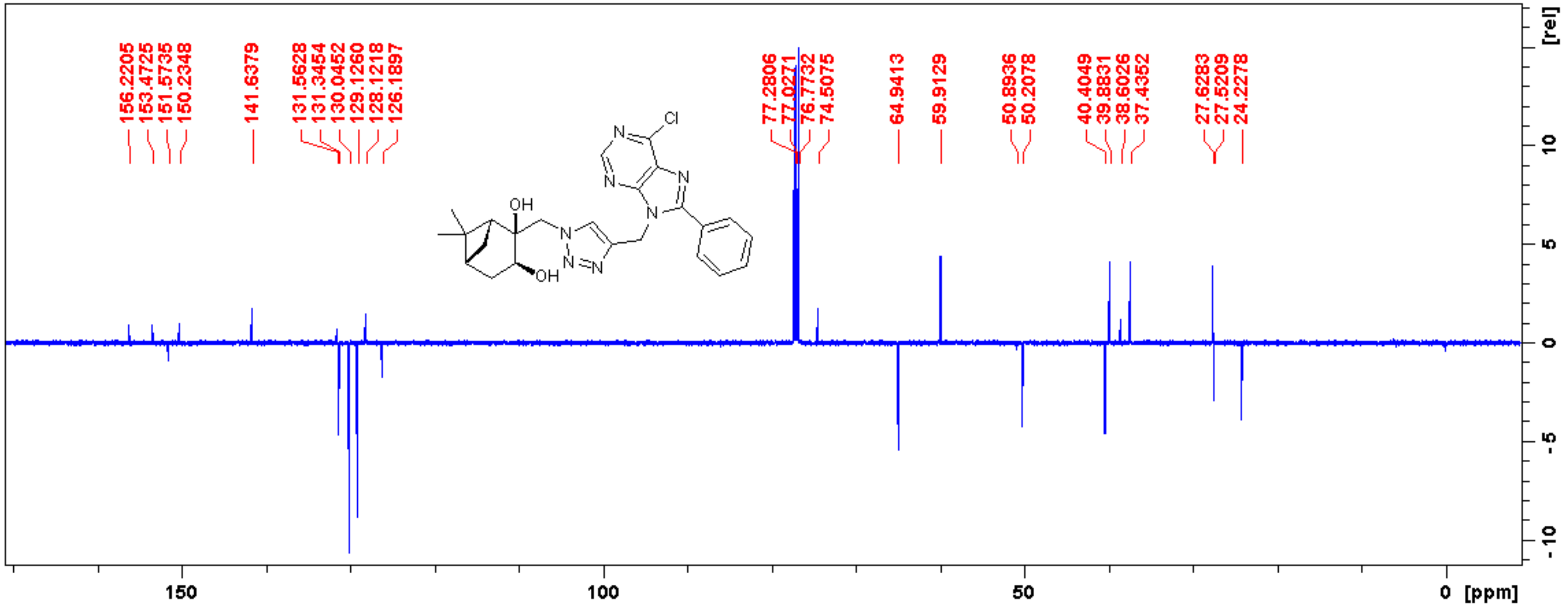

Figure S 146. COSY-NMR of compound **(–)-30**

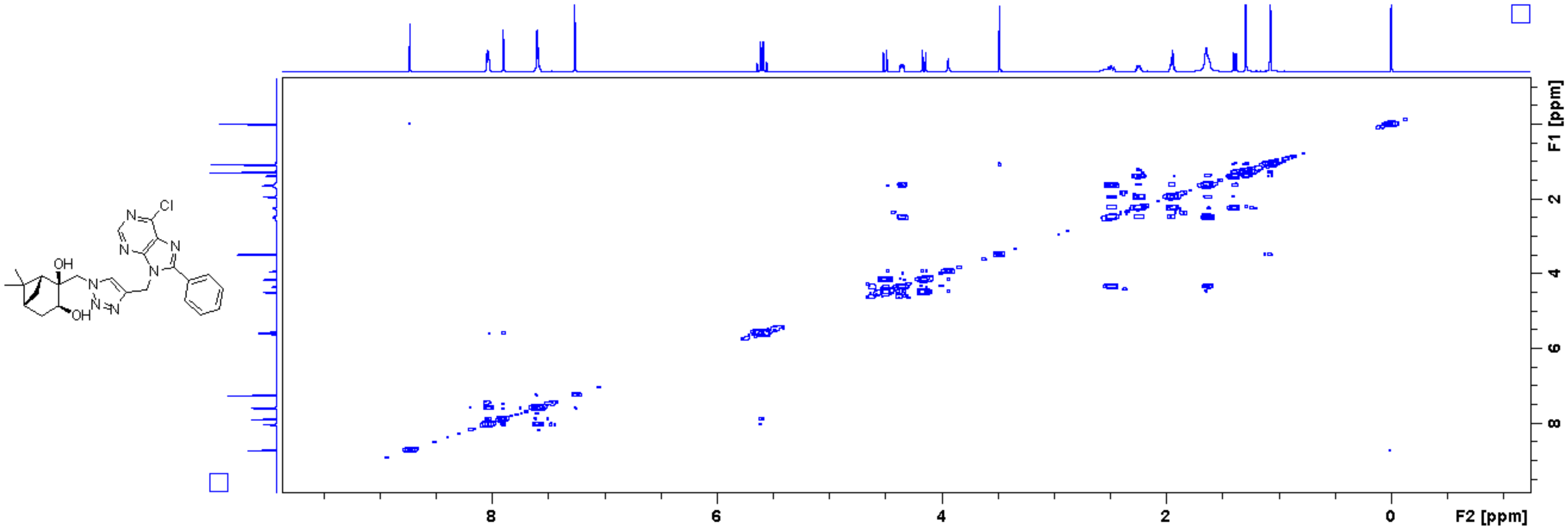

Figure S 147. NOESY-NMR of compound **(–)-30**

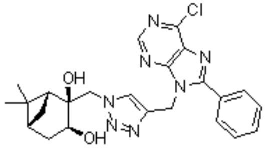

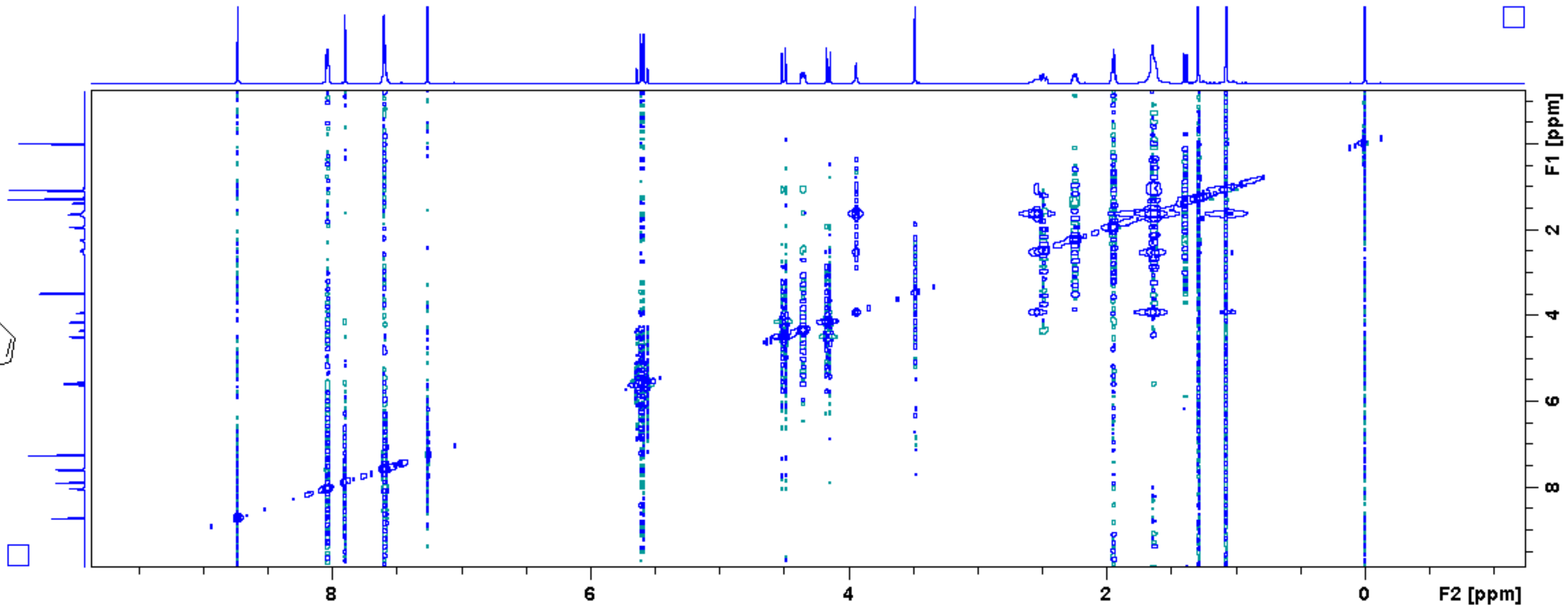

Figure S 148. HSQC-NMR of compound **(–)-30**

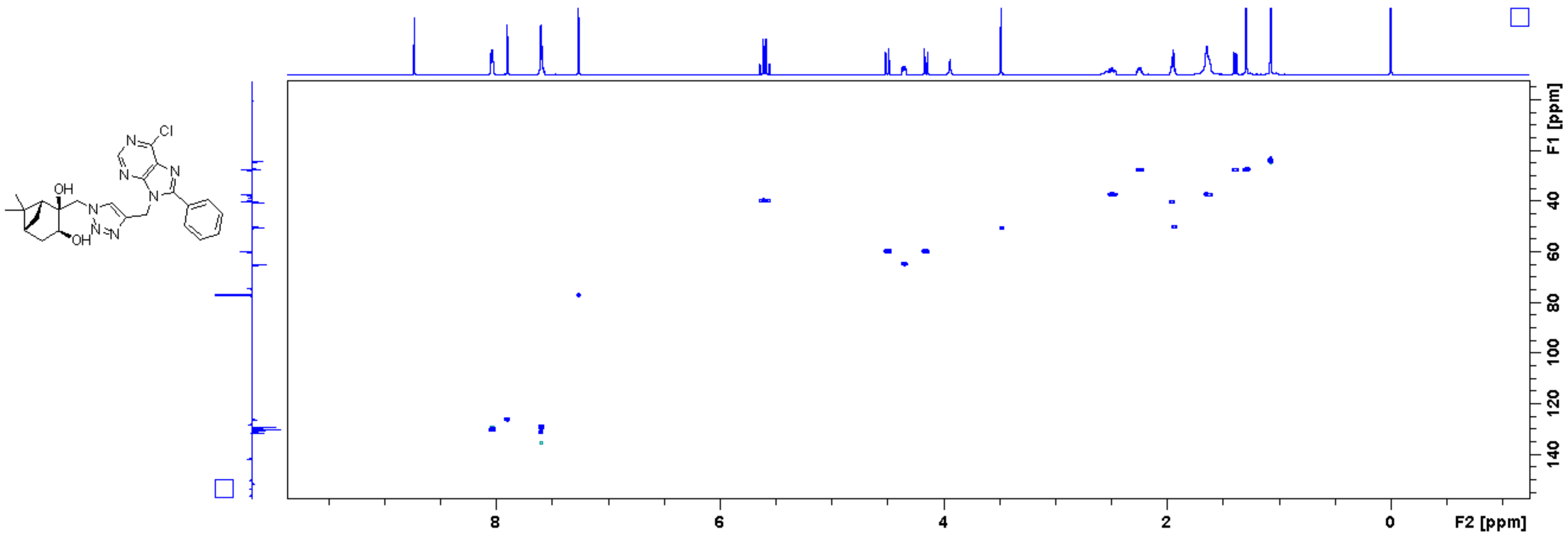

Figure S 149. HMBC-NMR of compound **(–)-30**

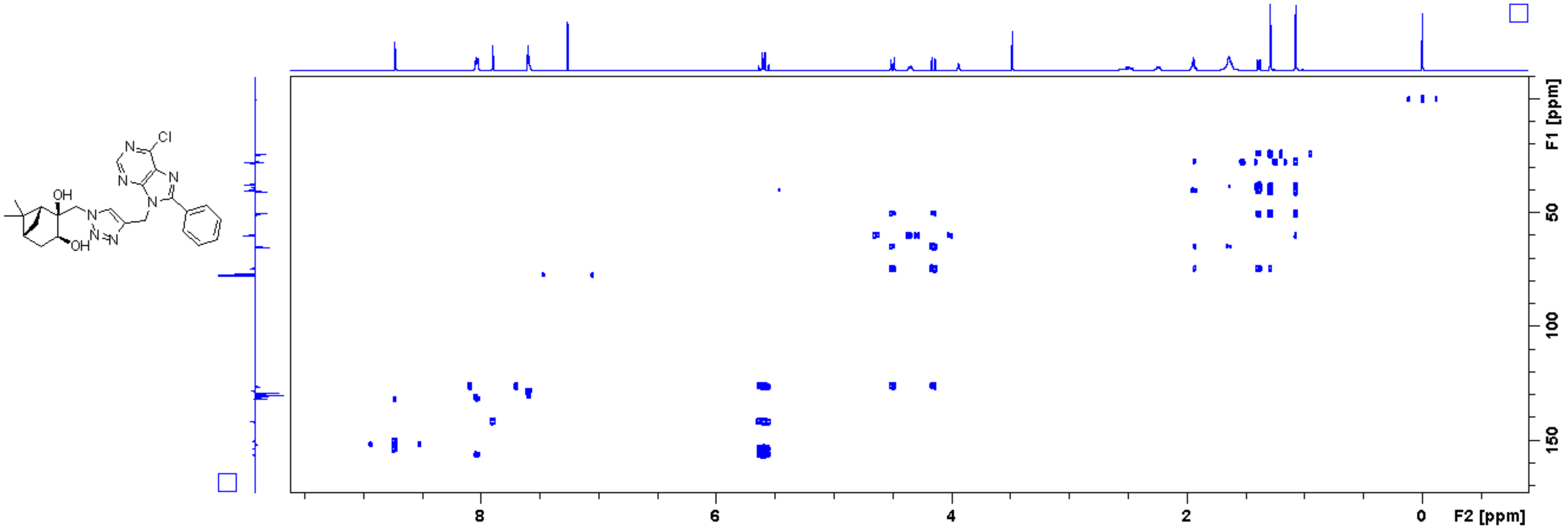

(1*S*,2*R*,3*R*,5*S*)-6,6-Dimethyl-2-((4-((6-phenyl-9*H*-purin-9-yl)methyl)-1*H*-1,2,3-triazol-1-yl)methyl)bicyclo[3.1.1]heptane-2,3-diol **(+)-31**

Figure S 150. ^{1}H-NMR of compound **(+)-31**

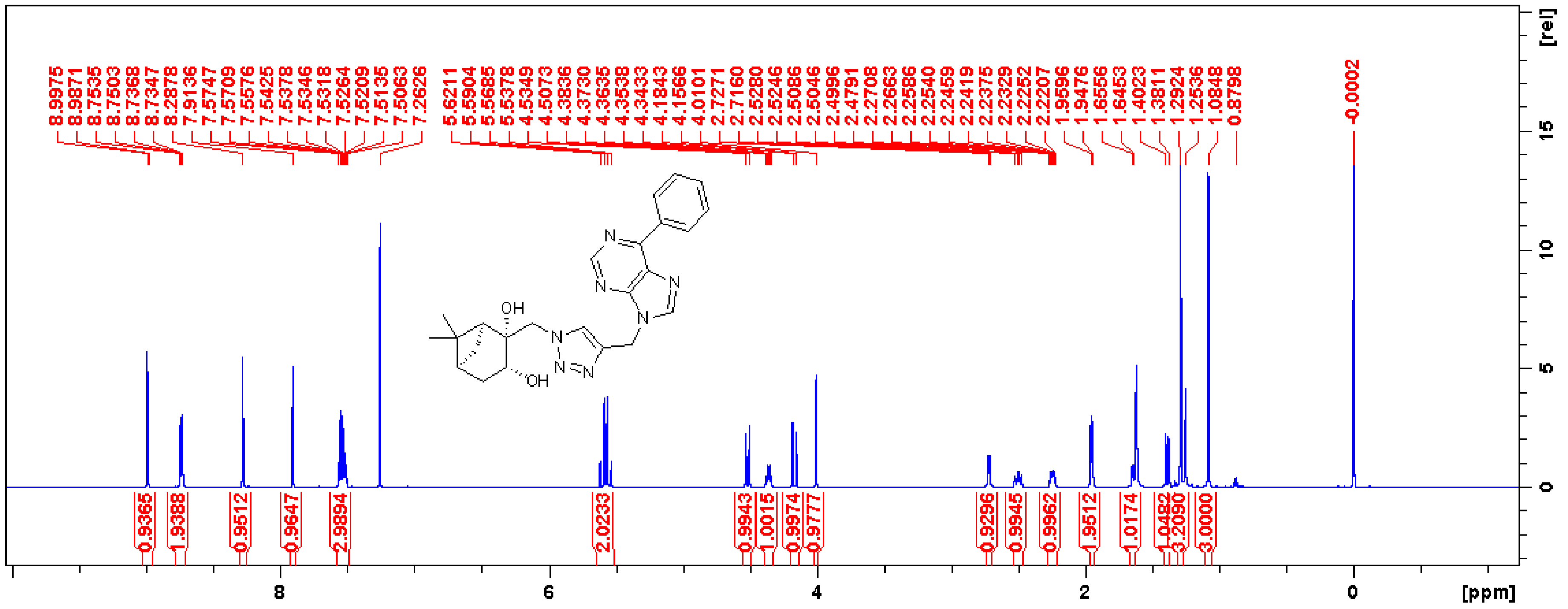

Figure S 151. ^{13}C-NMR of compound **(+)-31**

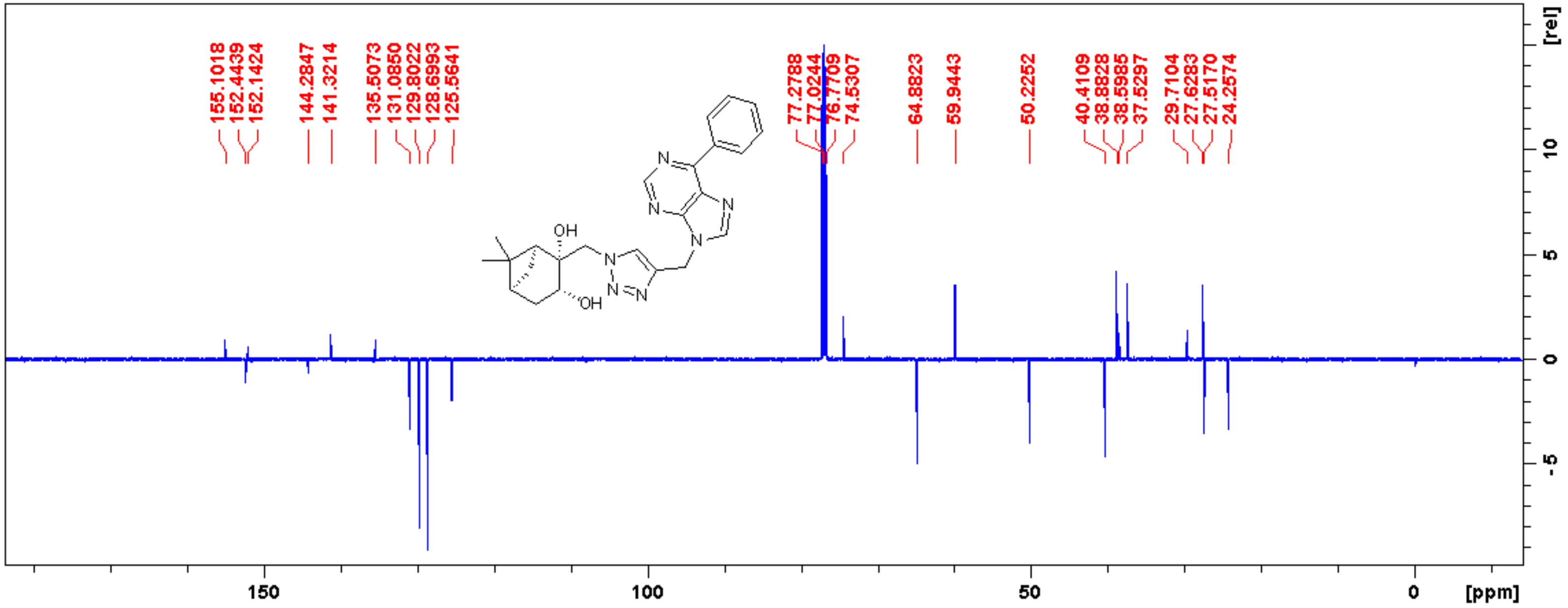

Figure S 152. COSY-NMR of compound **(+)-31**

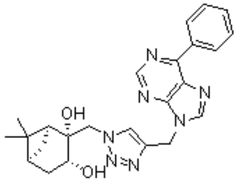

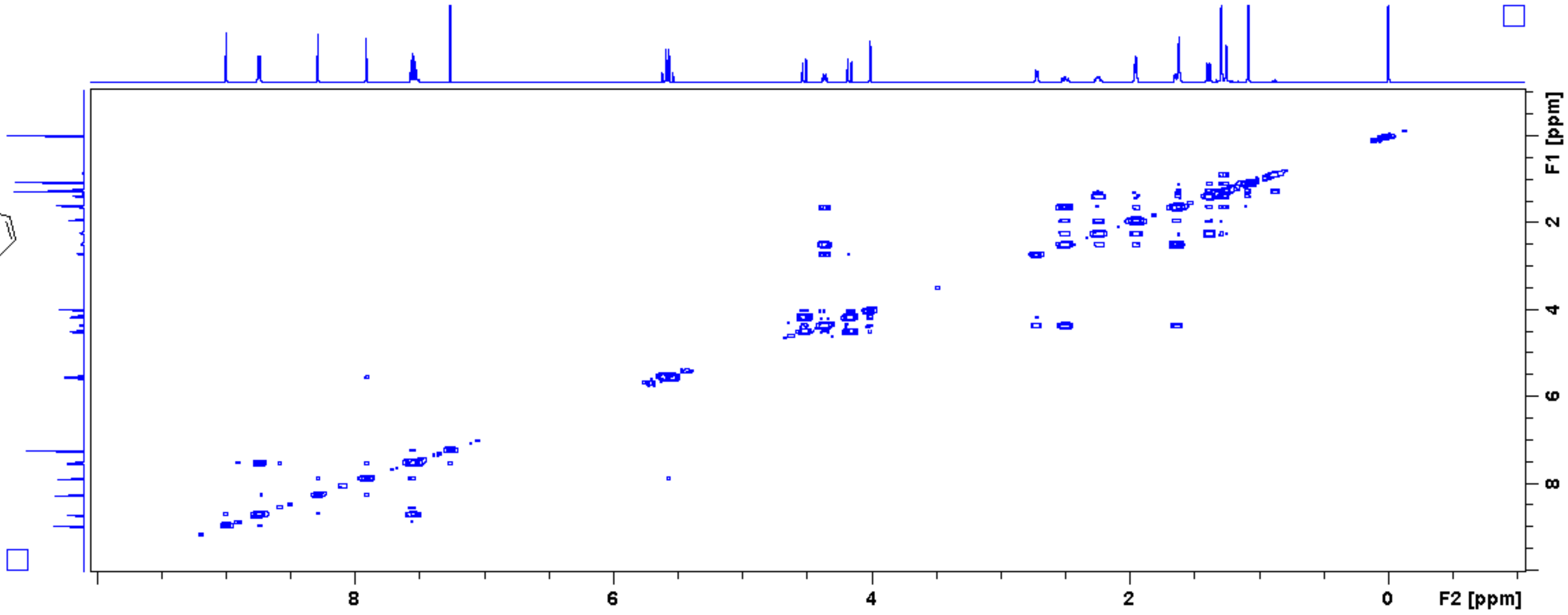

Figure S 153. NOESY-NMR of compound **(+)-31**

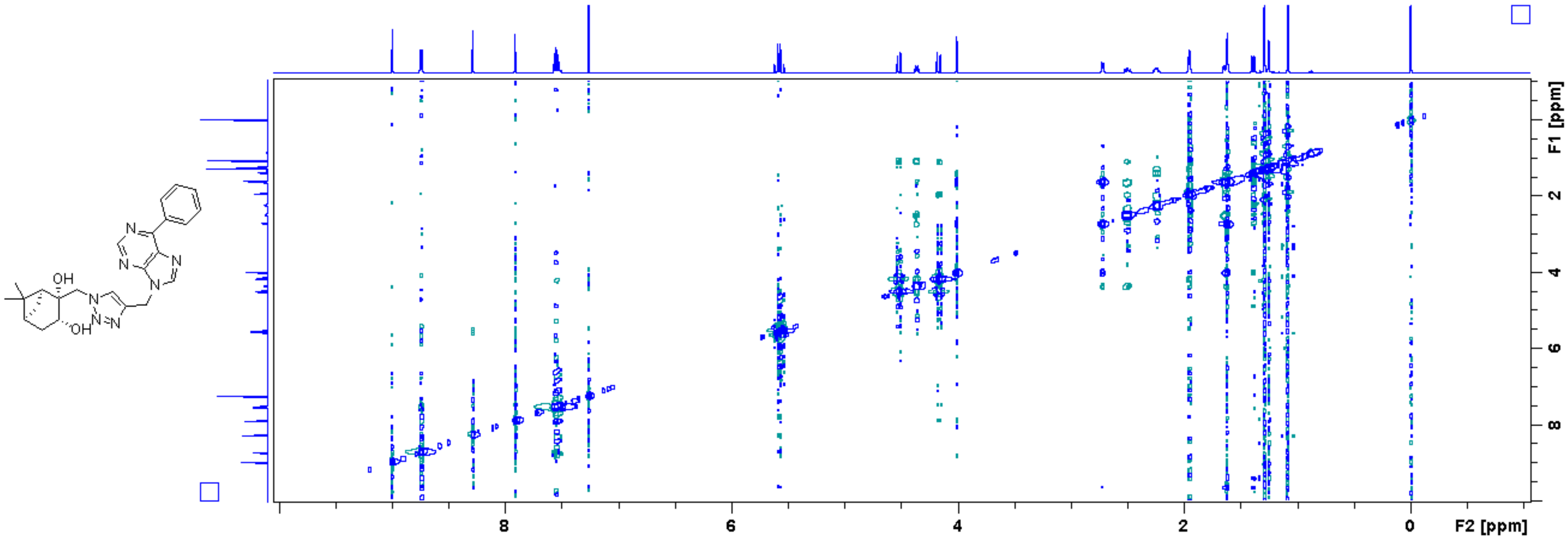

Figure S 154. HSQC-NMR of compound **(+)-31**

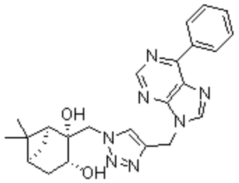

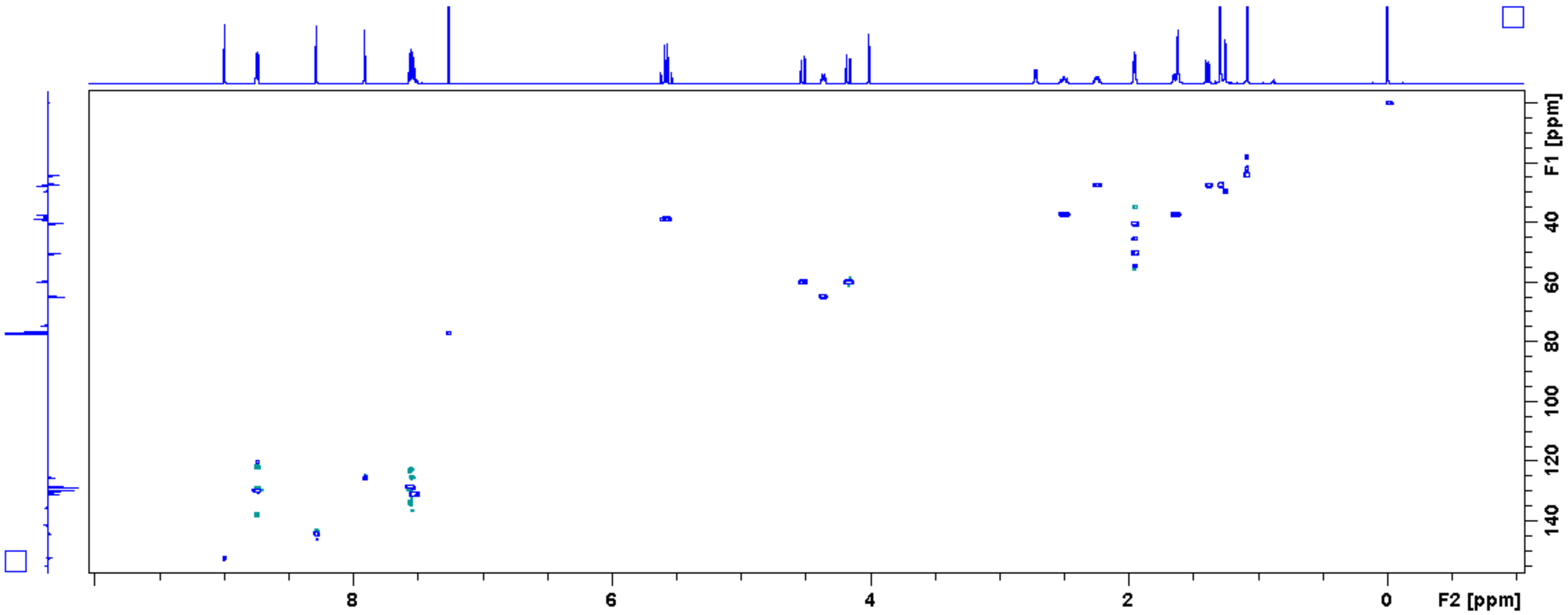

Figure S 155. HMBC-NMR of compound **(+)-31**

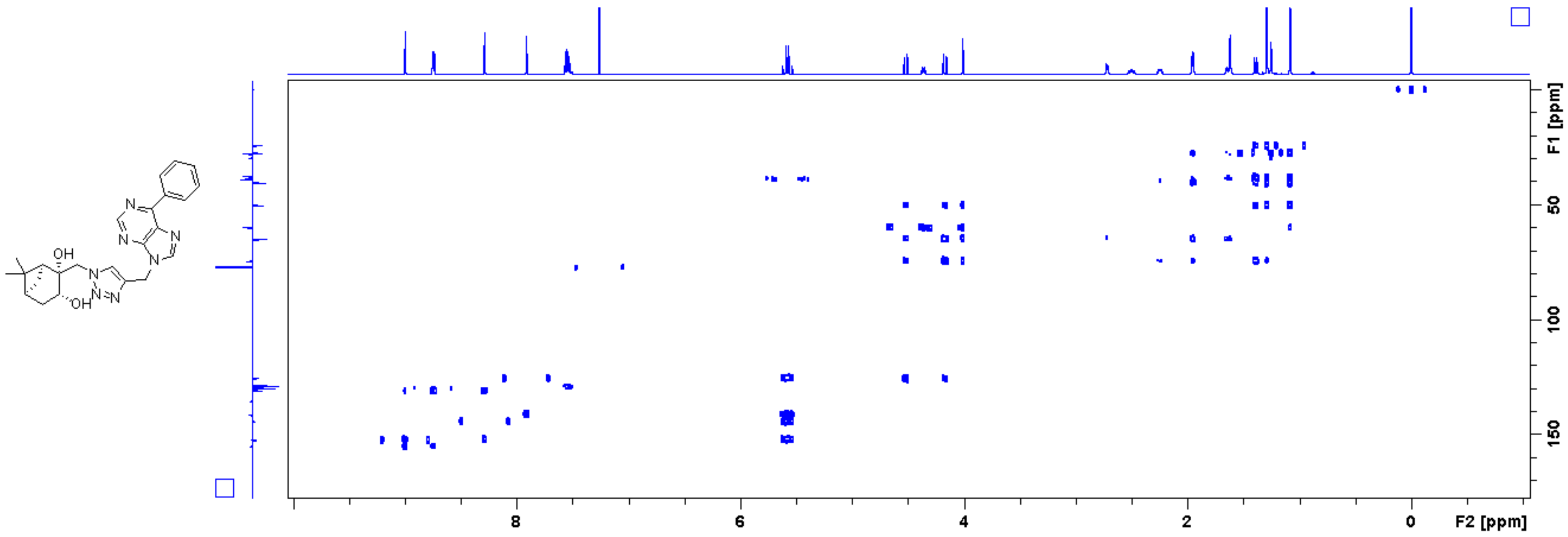

(1R,2S,3S,5R)-6,6-Dimethyl-2-((4-((6-phenyl-9H-purin-9-yl)methyl)-1H-1,2,3-triazol-1-yl)methyl)bicyclo[3.1.1]heptane-2,3-diol **(–)-31**

Figure S 156. ^{1}H-NMR of compound **(–)-31**

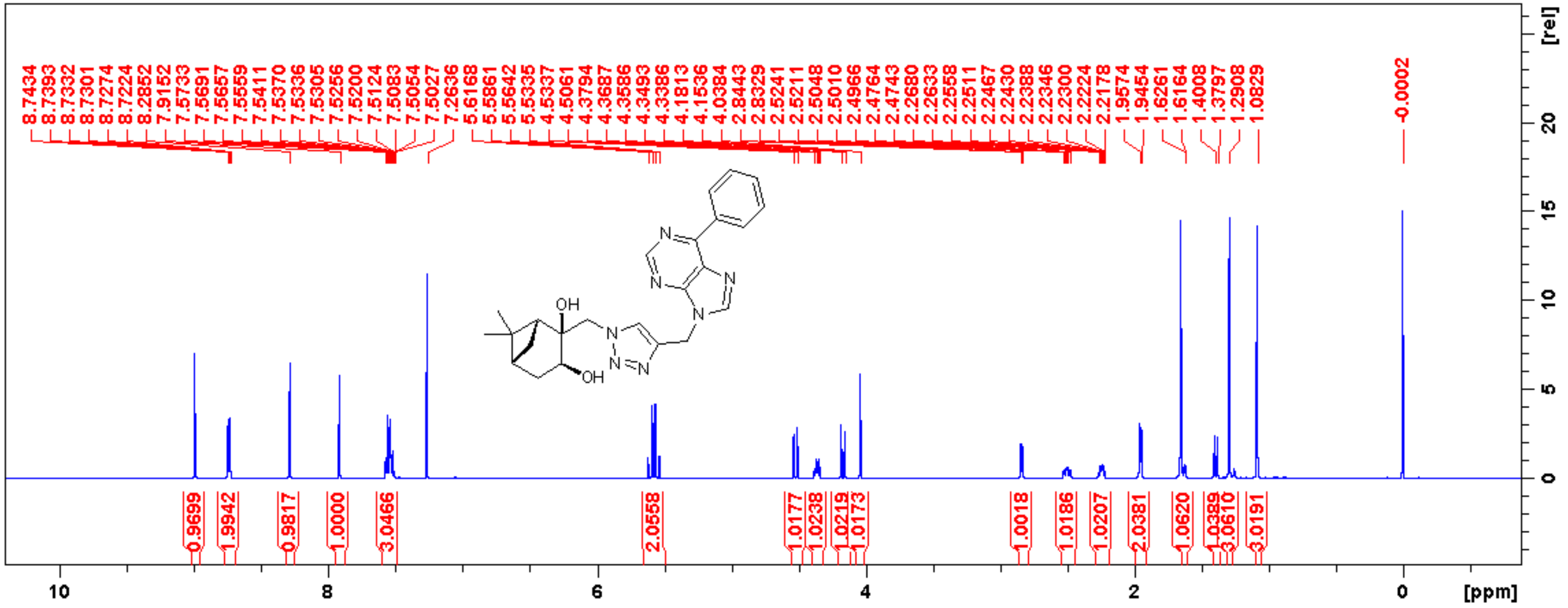

Figure S 157. ^{13}C-NMR of compound **(–)-31**

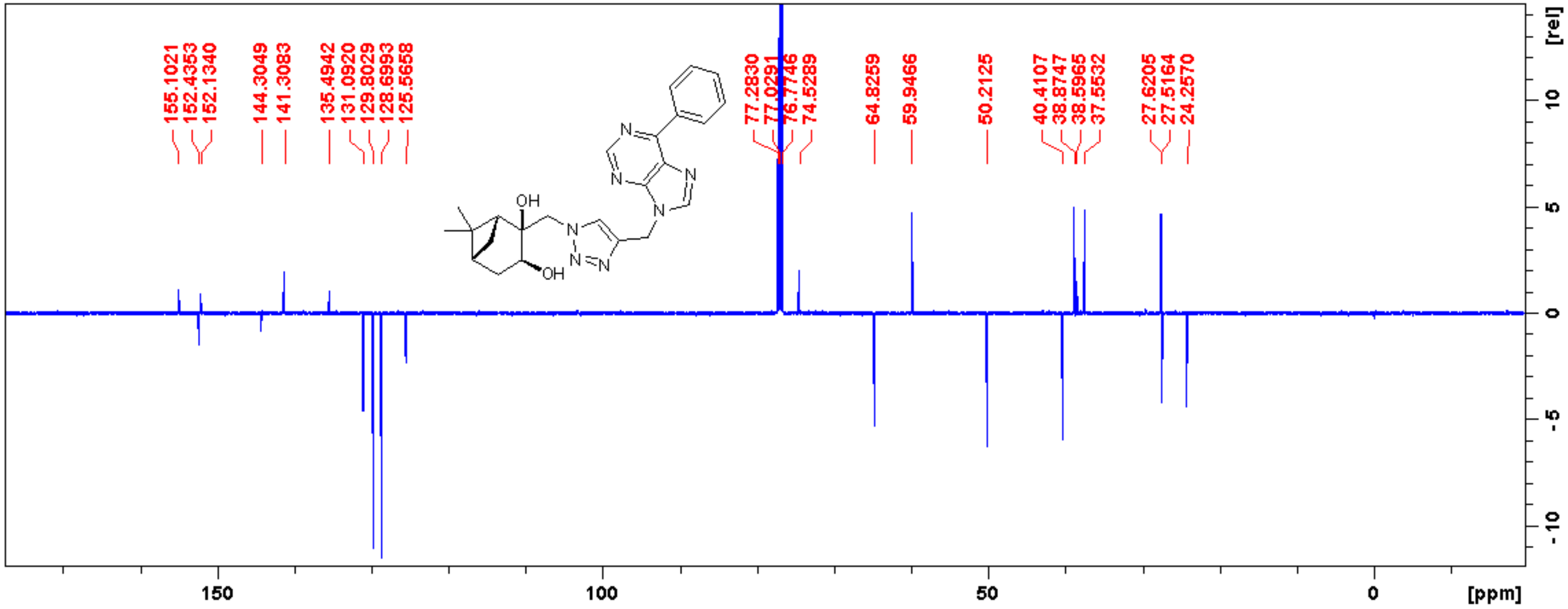

Figure S 158. COSY-NMR of compound **(–)-31**

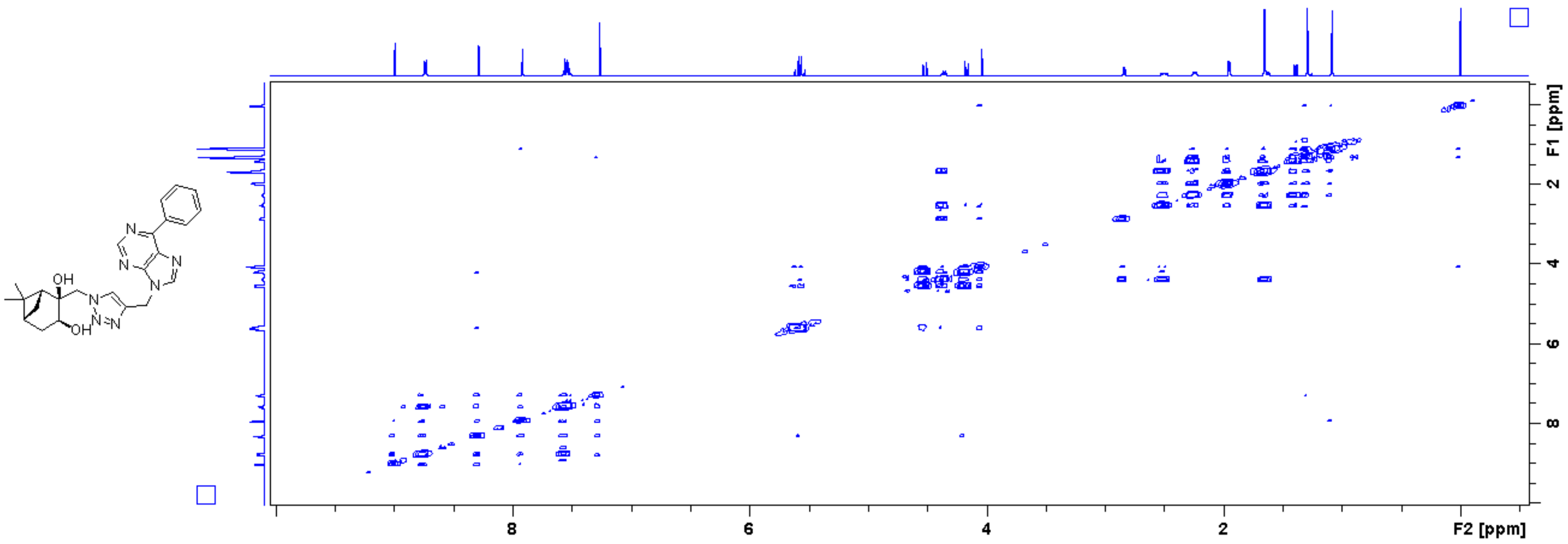

Figure S 159. NOESY-NMR of compound **(–)-31**

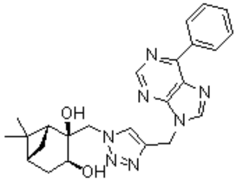

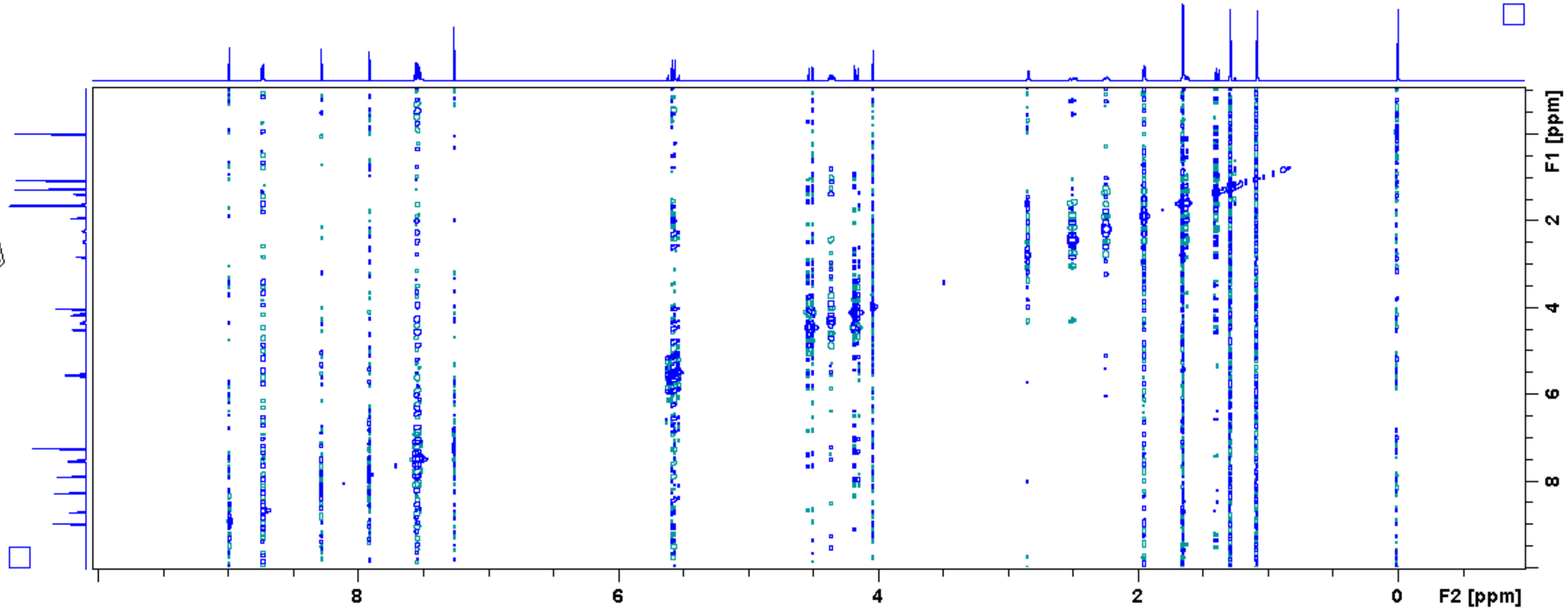

Figure S 160. HSQC-NMR of compound **(–)-31**

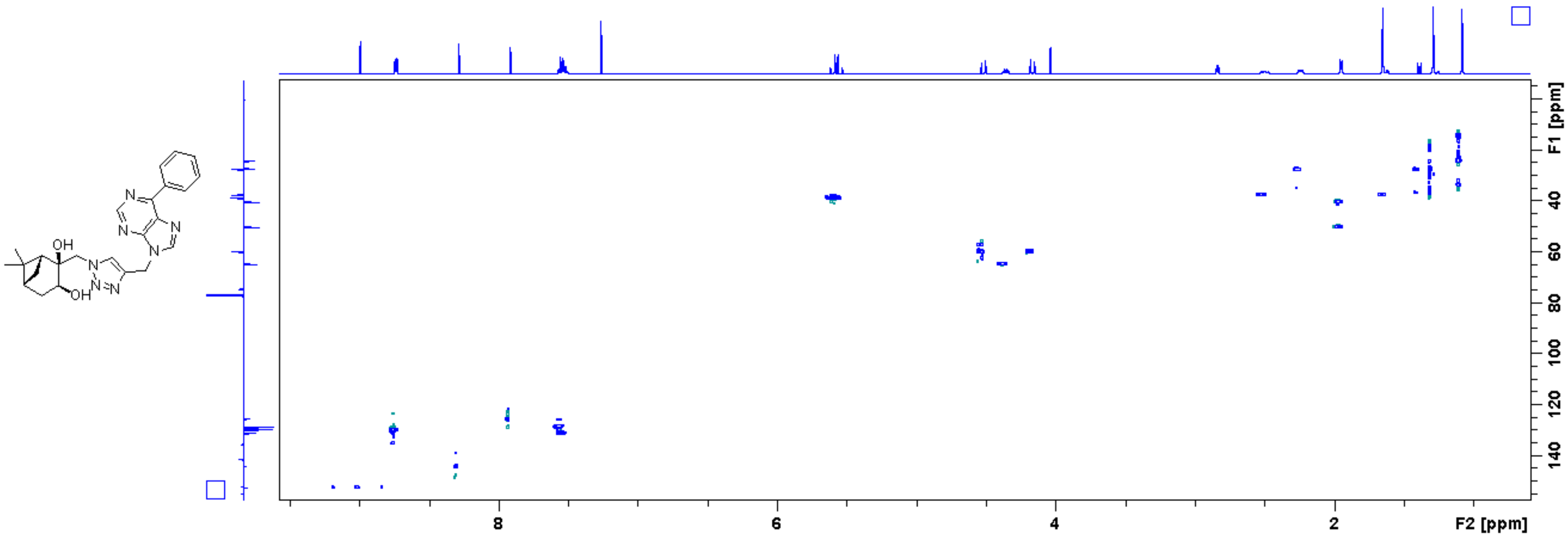

Figure S 161. HMBC-NMR of compound **(–)-31**

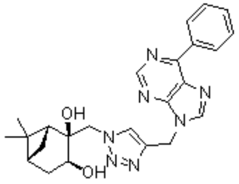

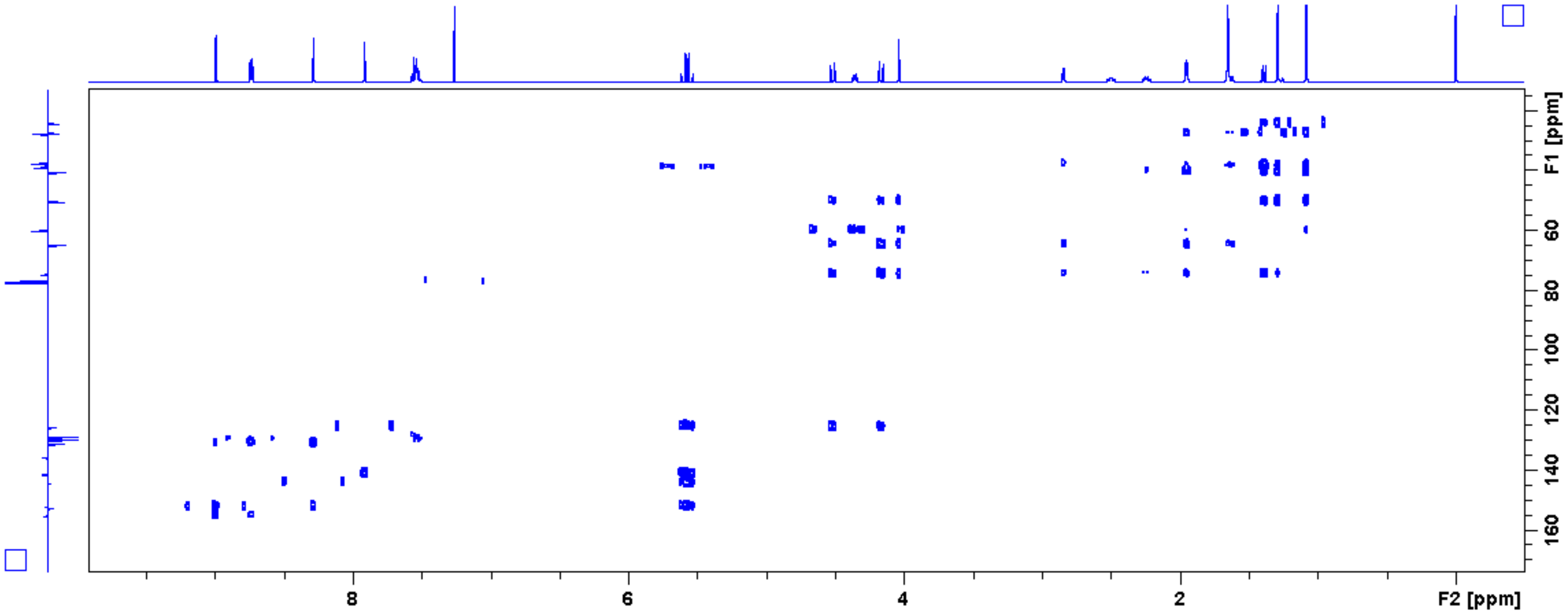

(1*S*,2*R*,3*R*,5*S*)-2-((4-((6-(Benzylamino)-9*H*-purin-9-yl)methyl)-1*H*-1,2,3-triazol-1-yl)methyl)-6,6-dimethylbicyclo[3.1.1]heptane-2,3-diol **(+)-32**

Figure S 162. ^{1}H-NMR of compound **(+)-32**

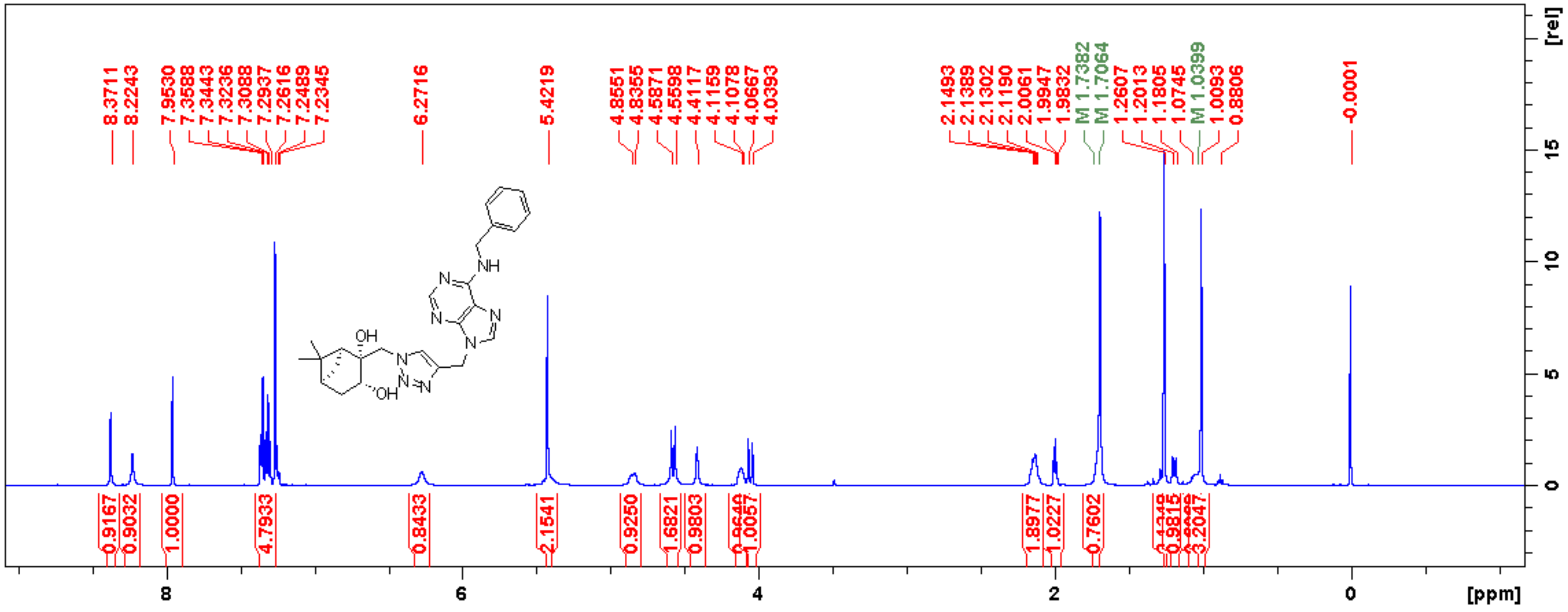

Figure S 163. ^{13}C-NMR of compound **(+)-32**

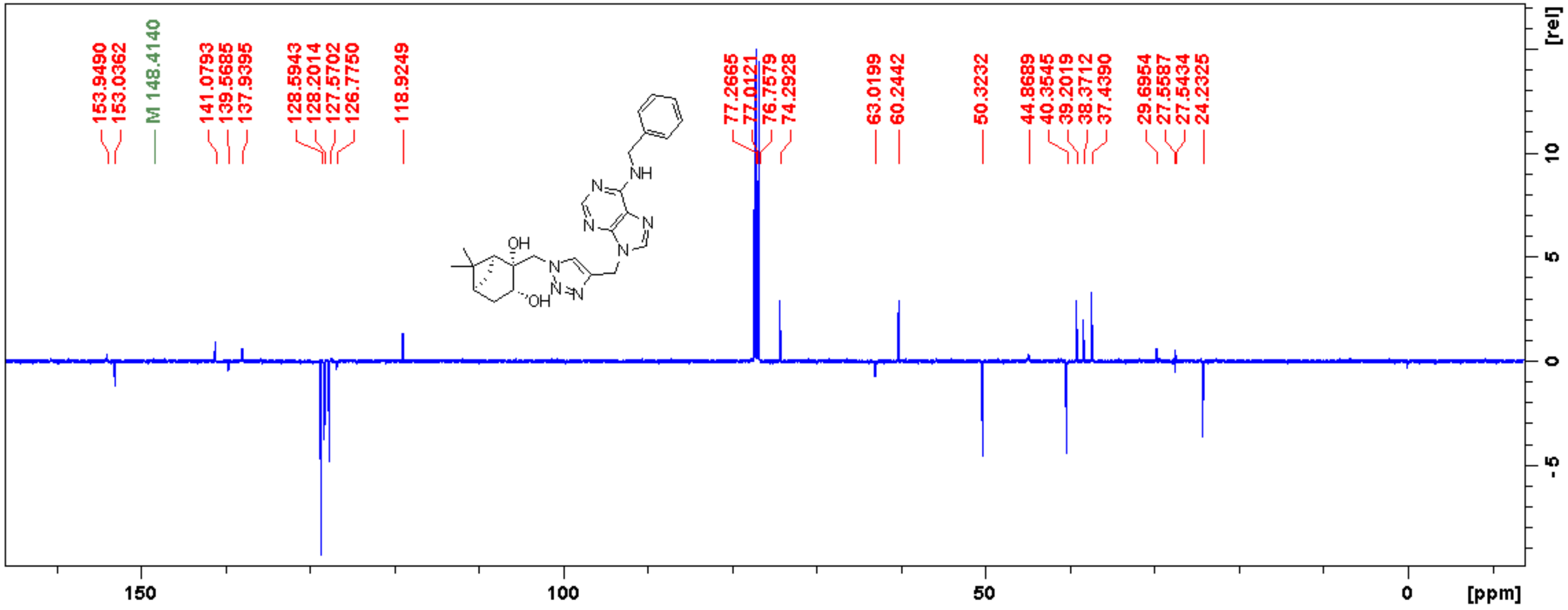

Figure S 164. COSY-NMR of compound **(+)-32**

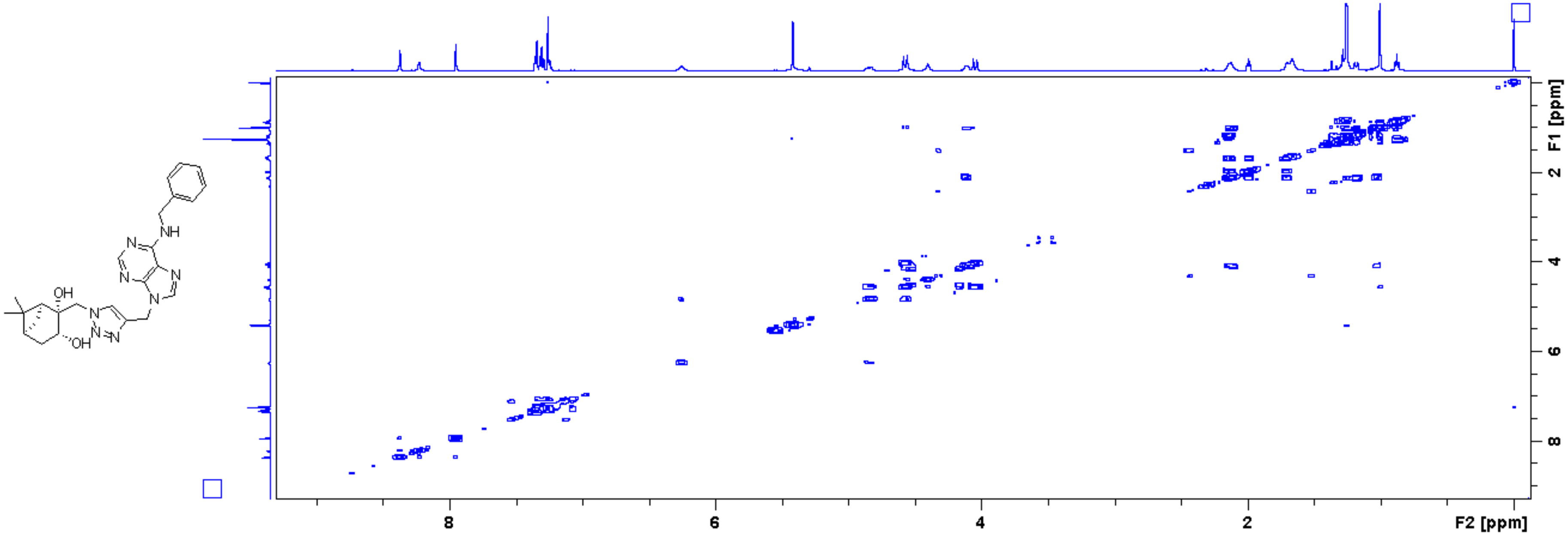

Figure S 165. NOESY-NMR of compound **(+)-32**

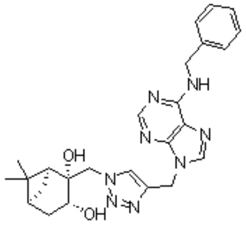

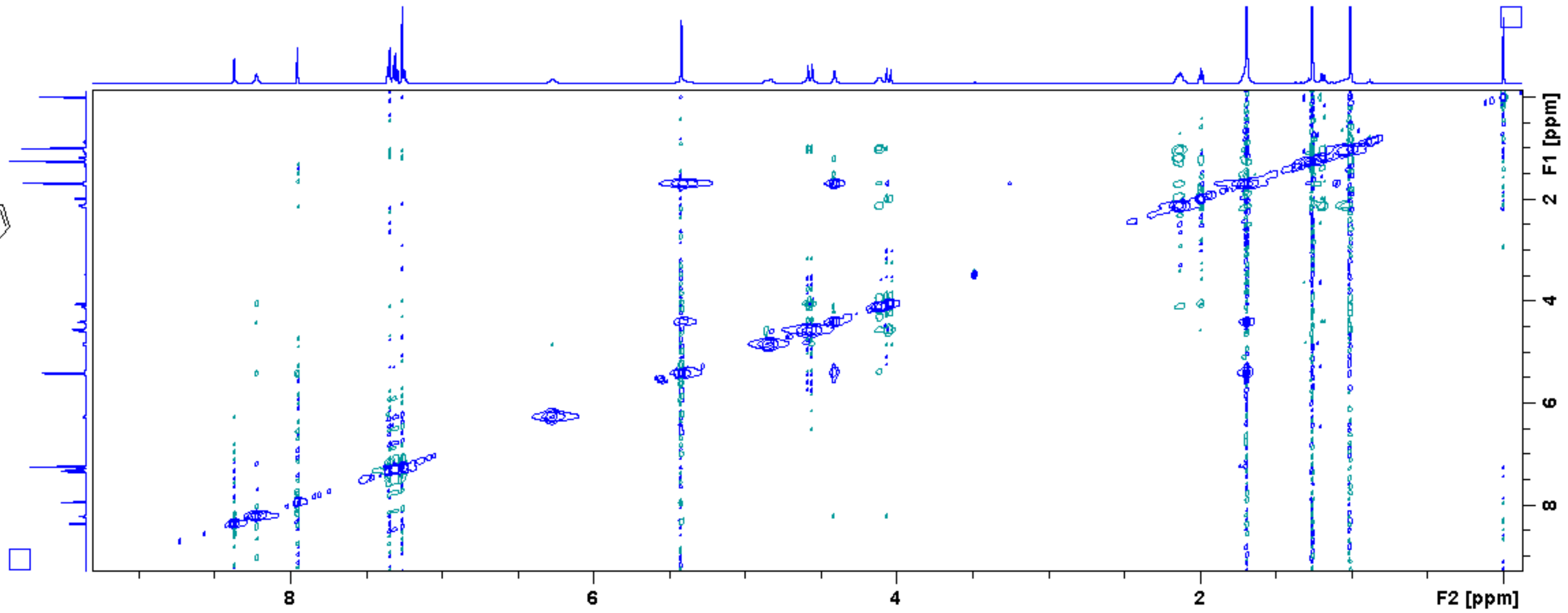

Figure S 166. HSQC-NMR of compound **(+)-32**

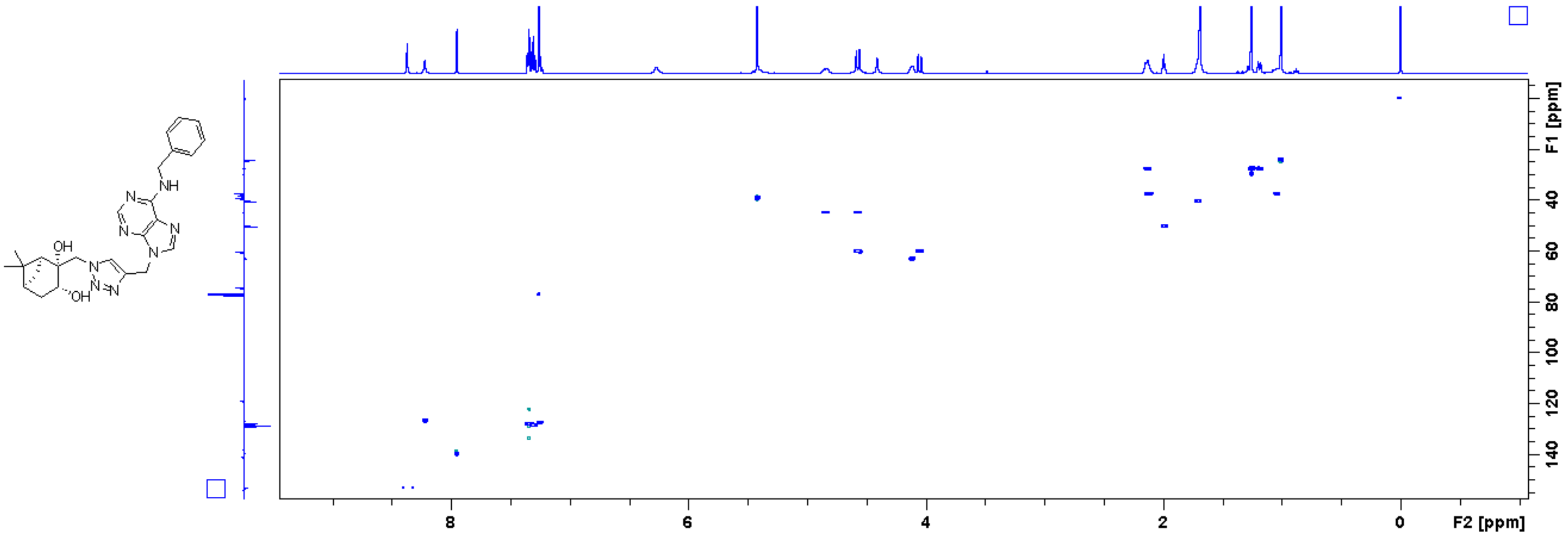

Figure S 167. HMBC-NMR of compound **(+)-32**

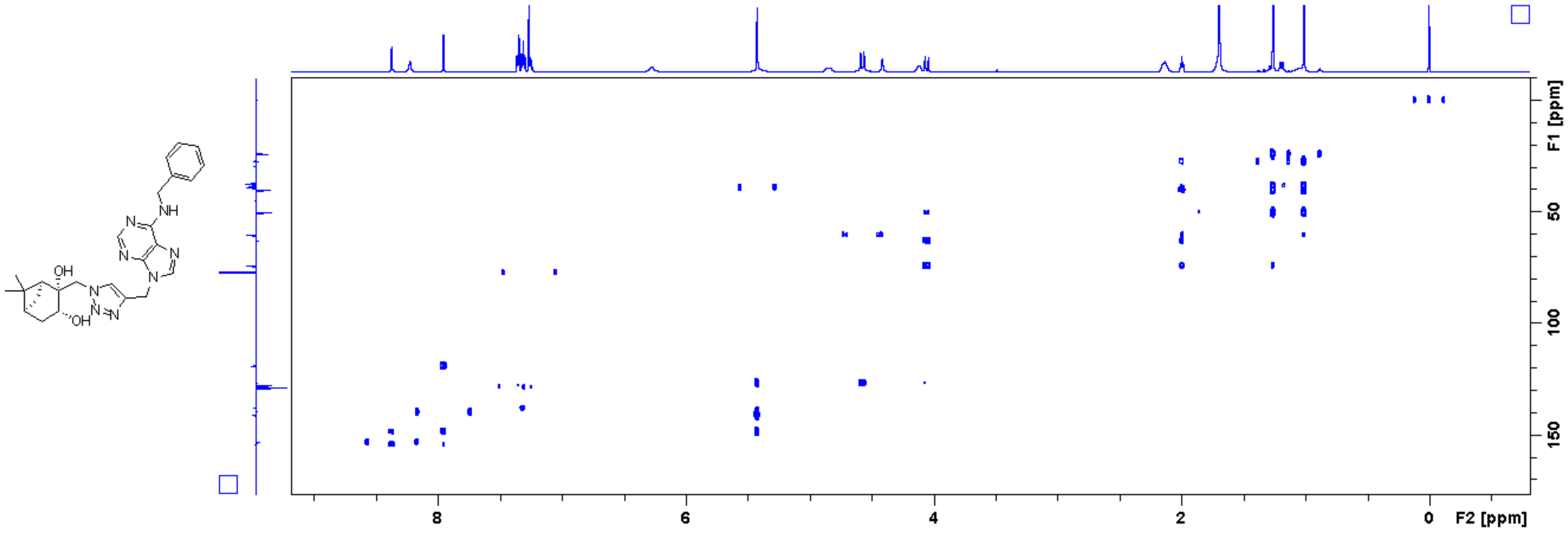

(1*R*,2*S*,3*S*,5*R*)-2-((4-((6-(Benzylamino)-9*H*-purin-9-yl)methyl)-1*H*-1,2,3-triazol-1-yl)methyl)-6,6-dimethylbicyclo[3.1.1]heptane-2,3-diol **(–)-32**

Figure S 168. ^{1}H-NMR of compound **(–)-32**

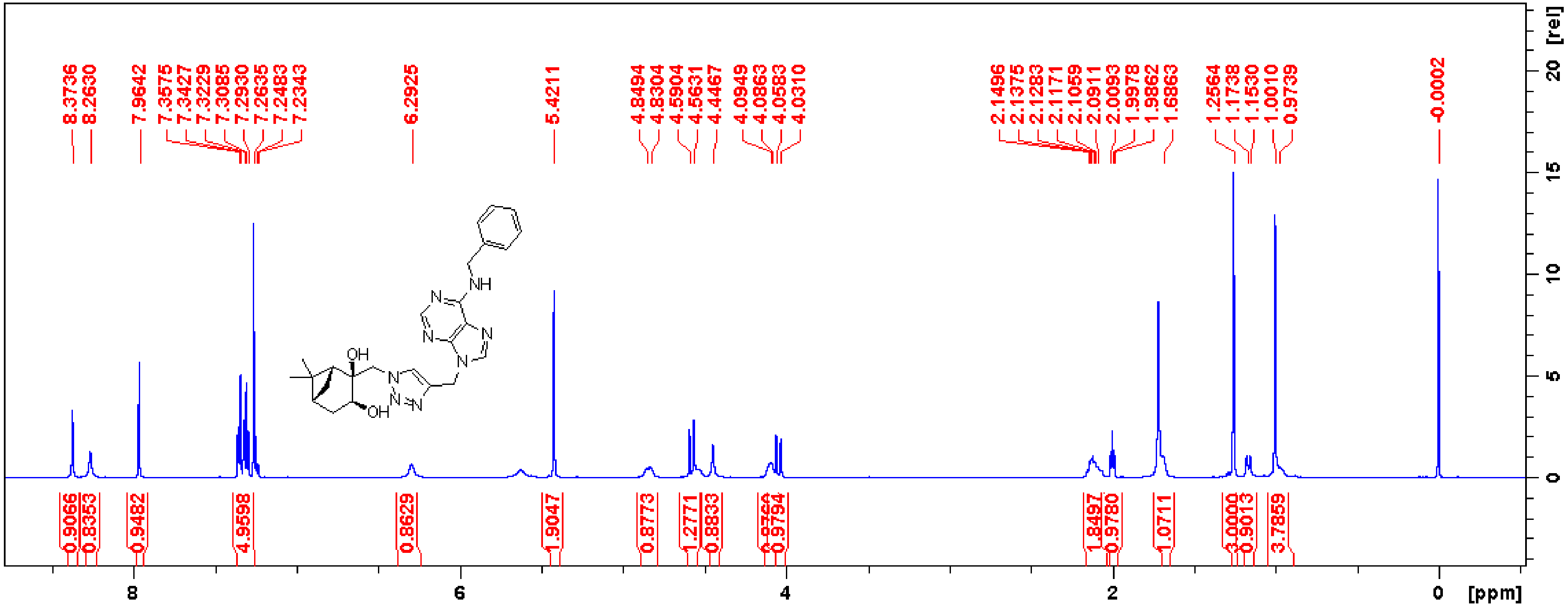

Figure S 169. ^{13}C-NMR of compound **(–)-32**

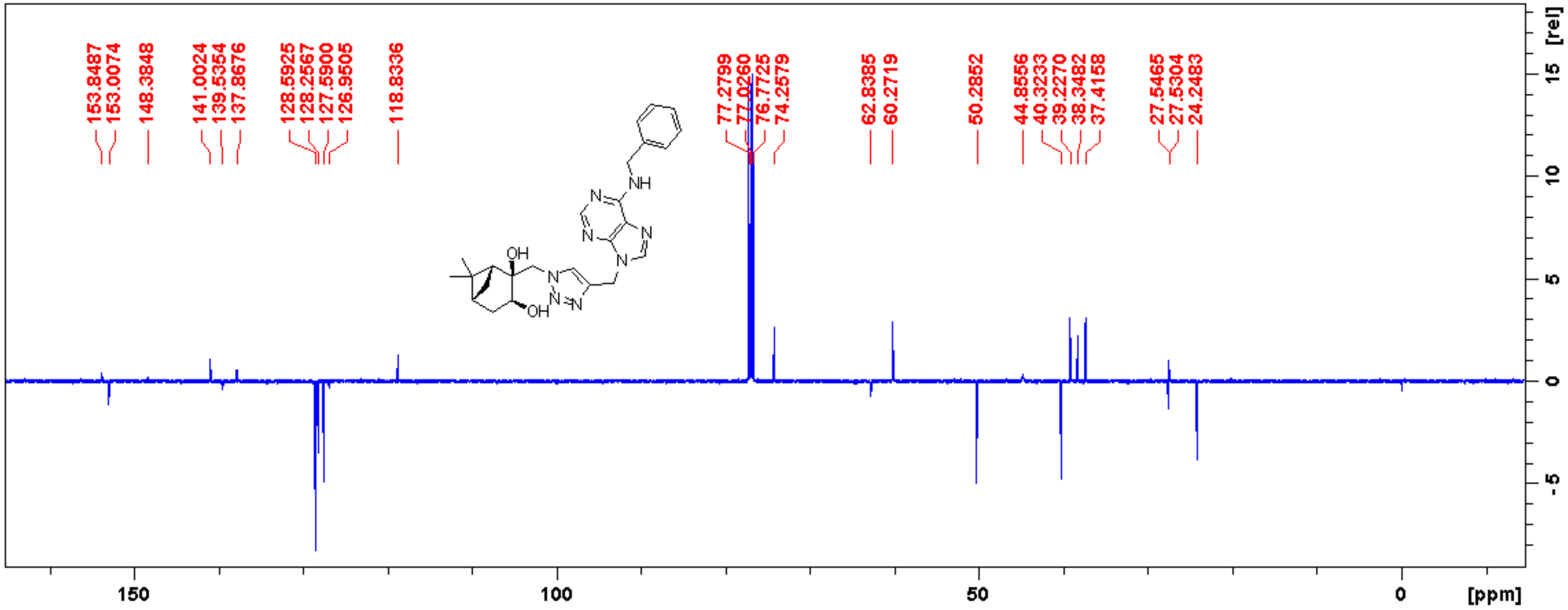

Figure S 170. COSY-NMR of compound **(–)-32**

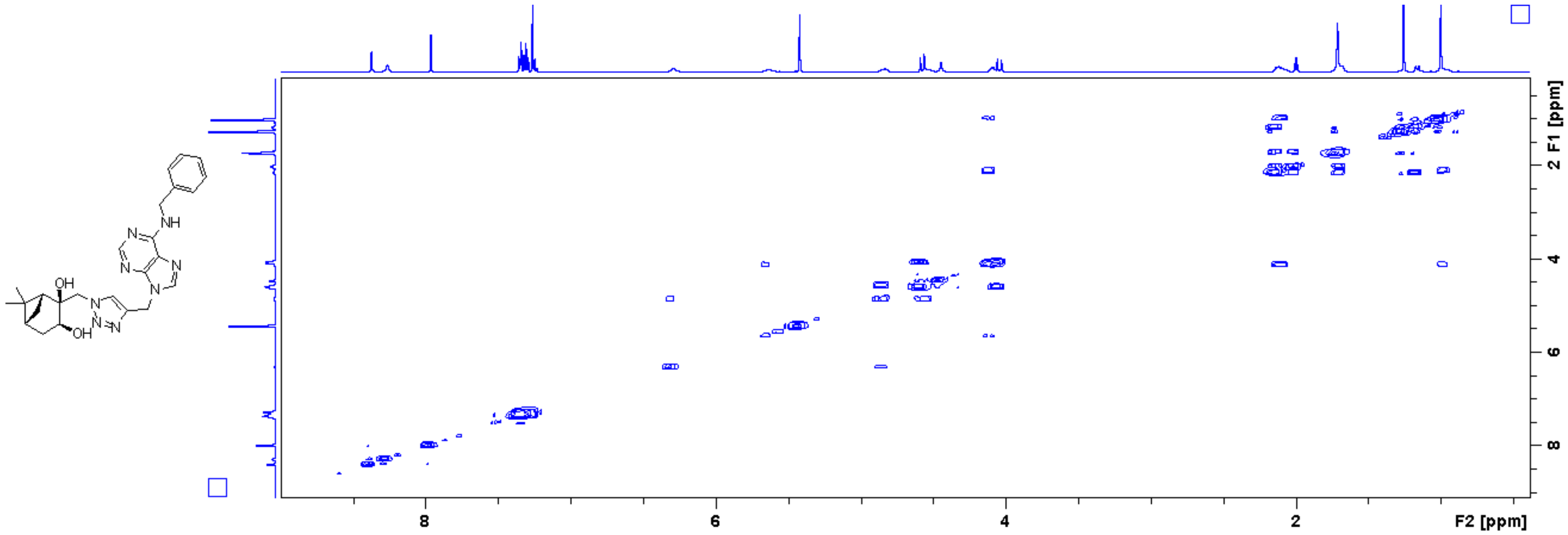

Figure S 171. NOESY-NMR of compound **(–)-32**

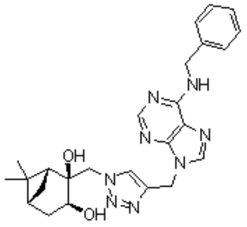

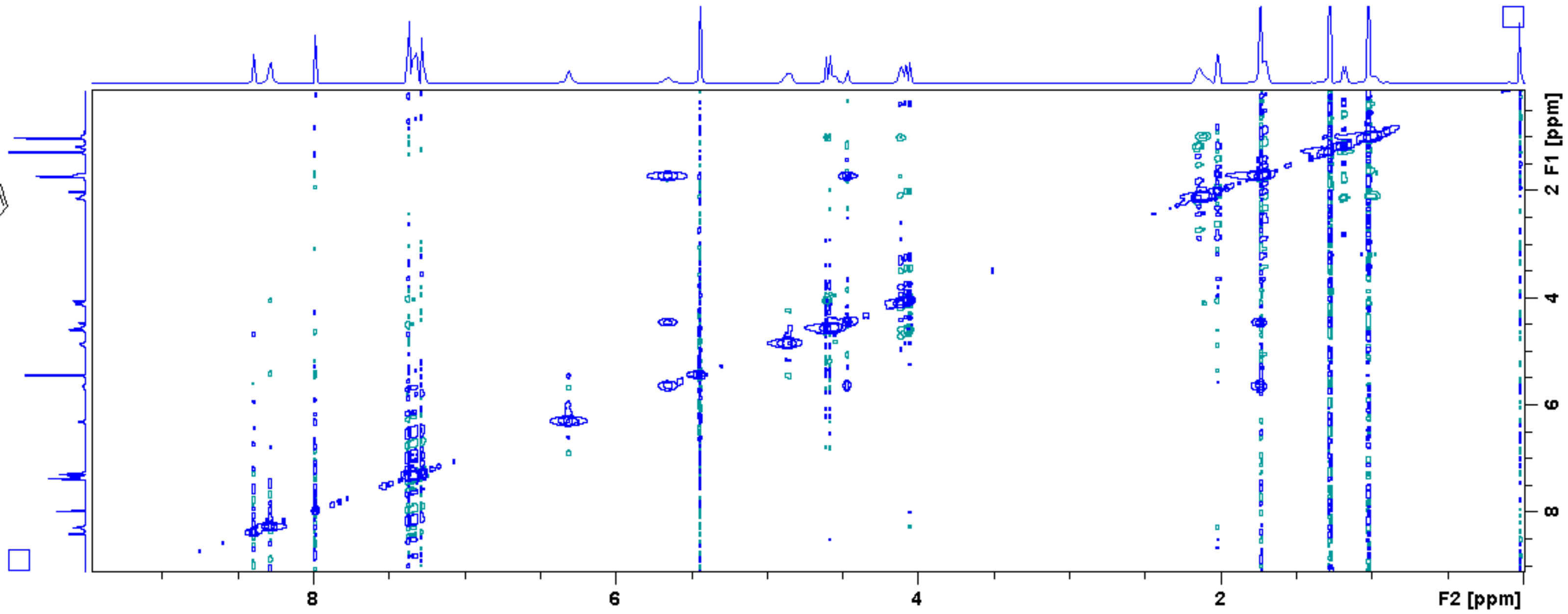

Figure S 172. HSQC-NMR of compound **(–)-32**

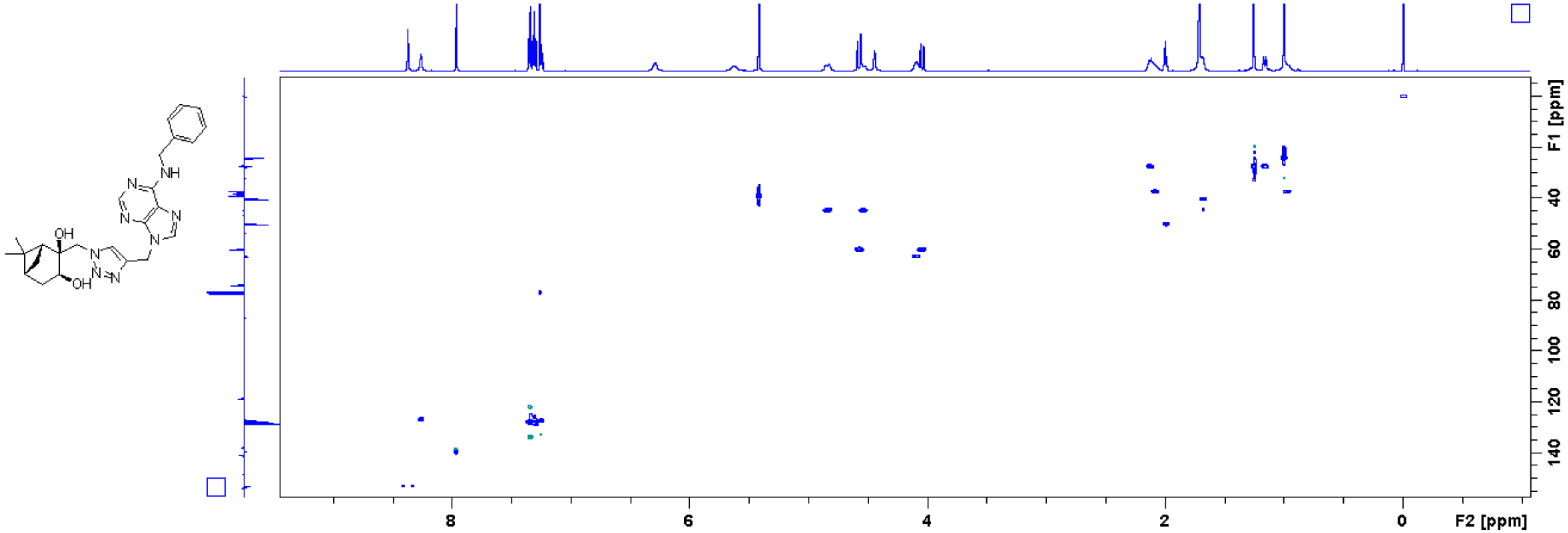

Figure S 173. HMBC-NMR of compound **(–)-32**

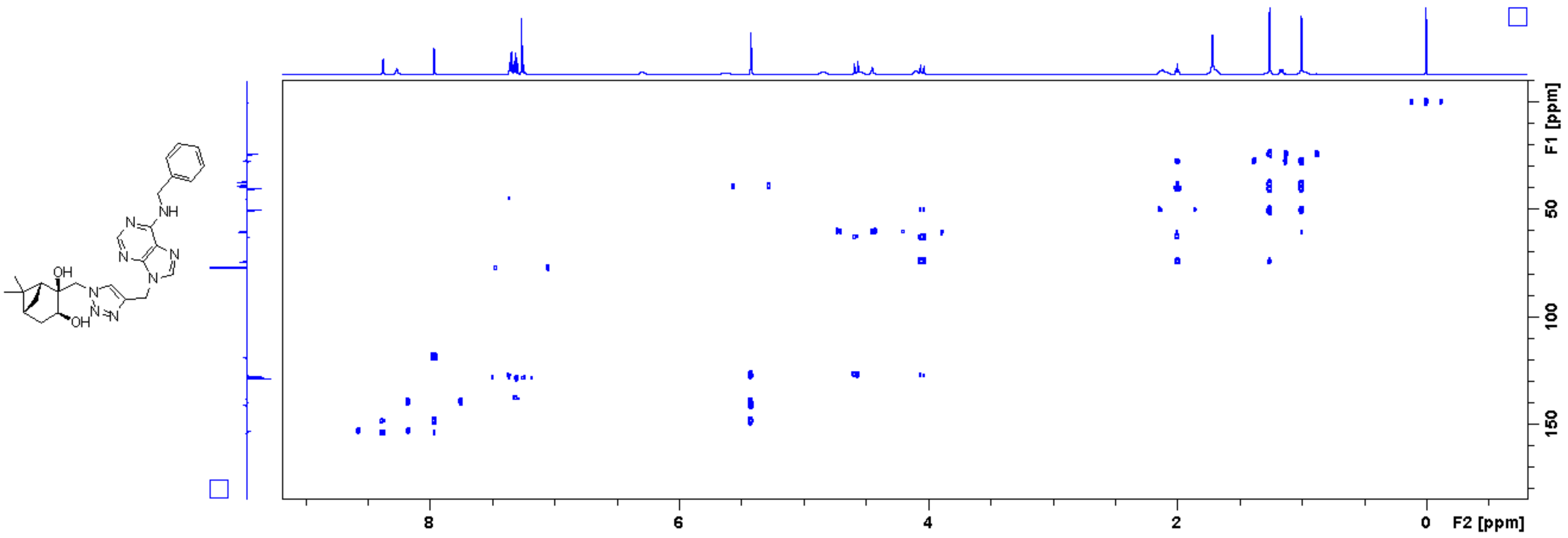

(1*S*,2*R*,3*R*,5*S*)-6,6-Dimethyl-2-((4-((6-((4-(trifluoromethyl)phenyl)amino)-9*H*-purin-9-yl)methyl)-1*H*-1,2,3-triazol-1-yl)methyl)bicyclo[3.1.1]heptane-2,3-diol **(+)-33**

Figure S 174. ^{1}H-NMR of compound **(+)-33**

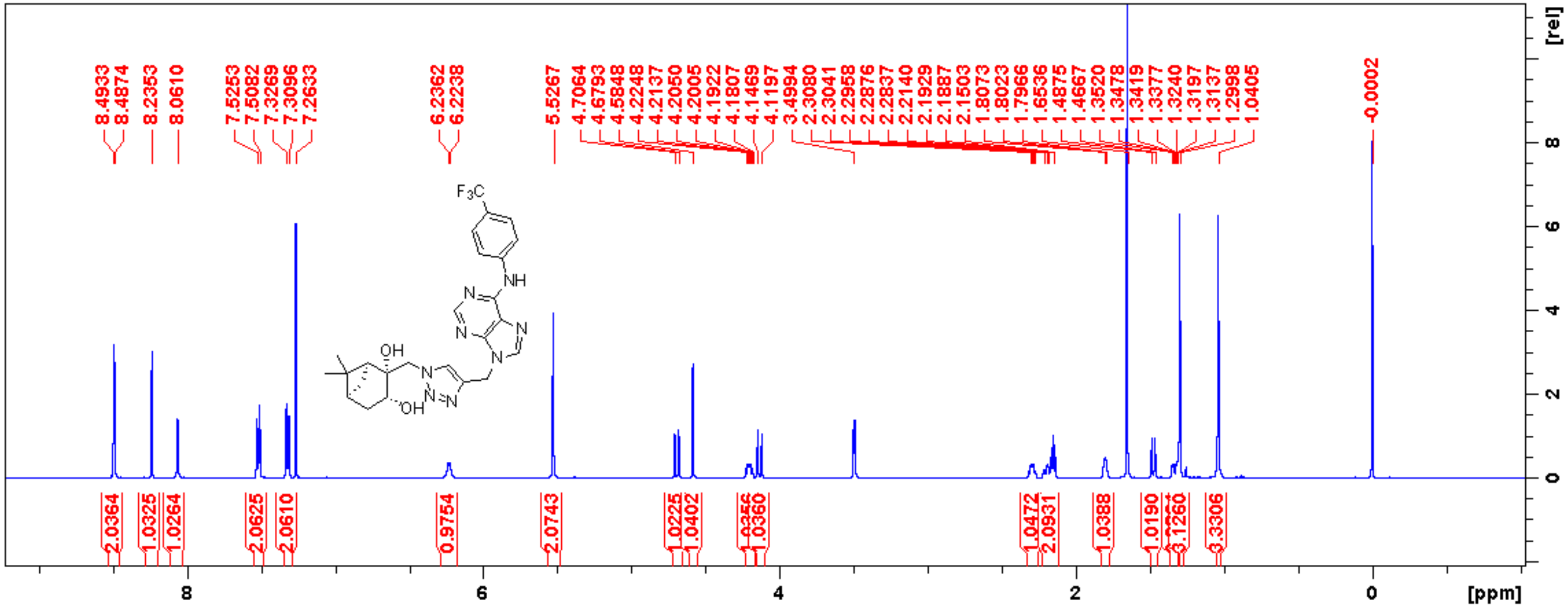

Figure S 175. ^{13}C-NMR of compound **(+)-33**

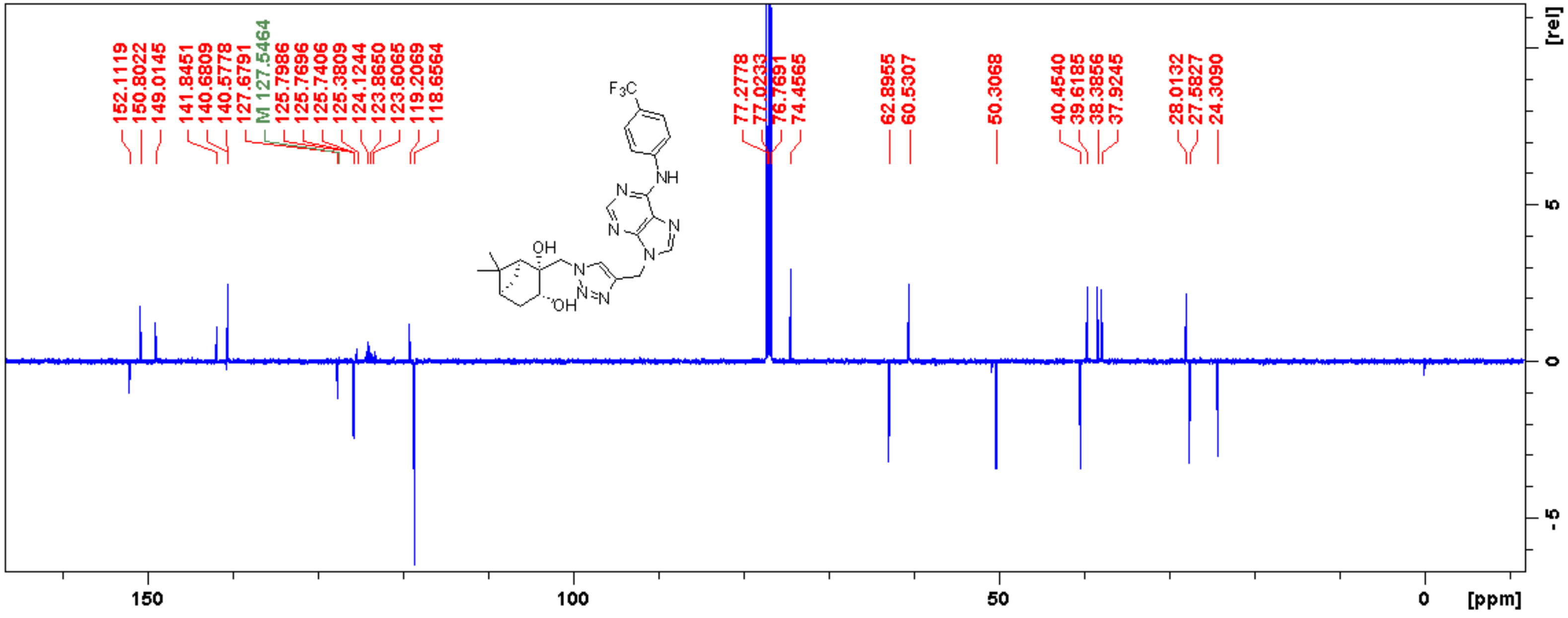

Figure S 176. COSY-NMR of compound **(+)-33**

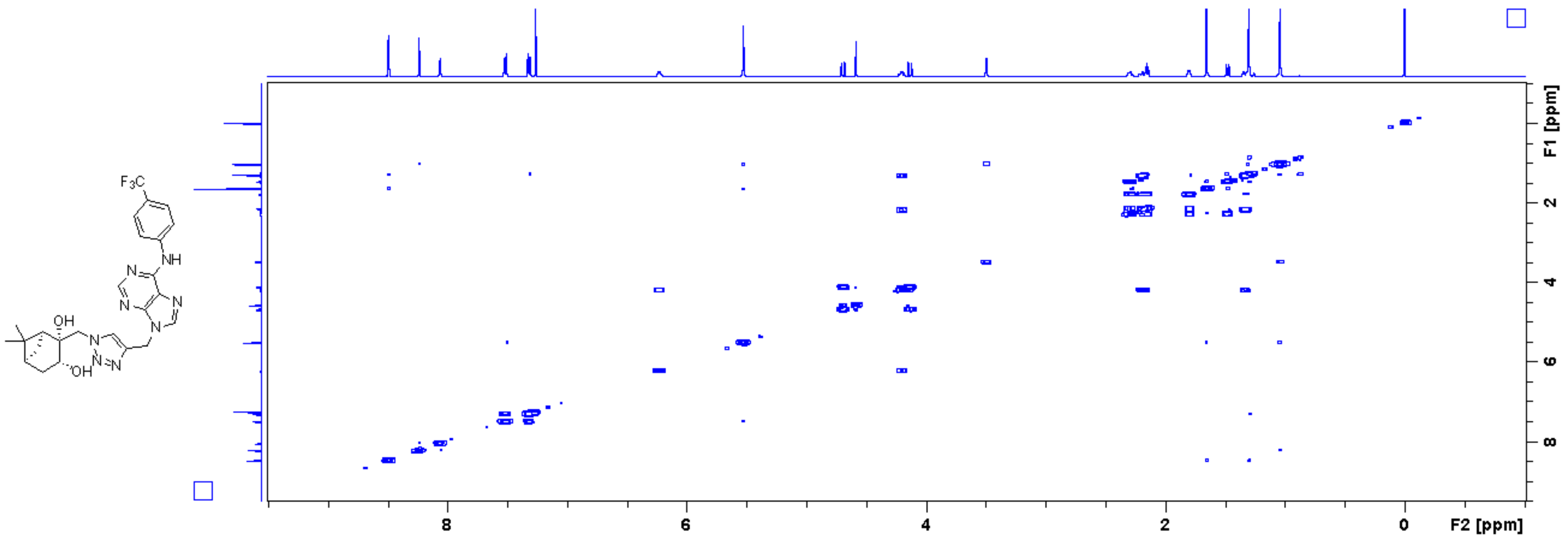

Figure S 177. NOESY-NMR of compound **(+)-33**

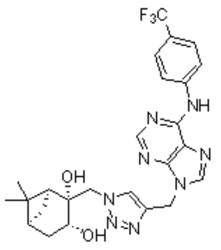

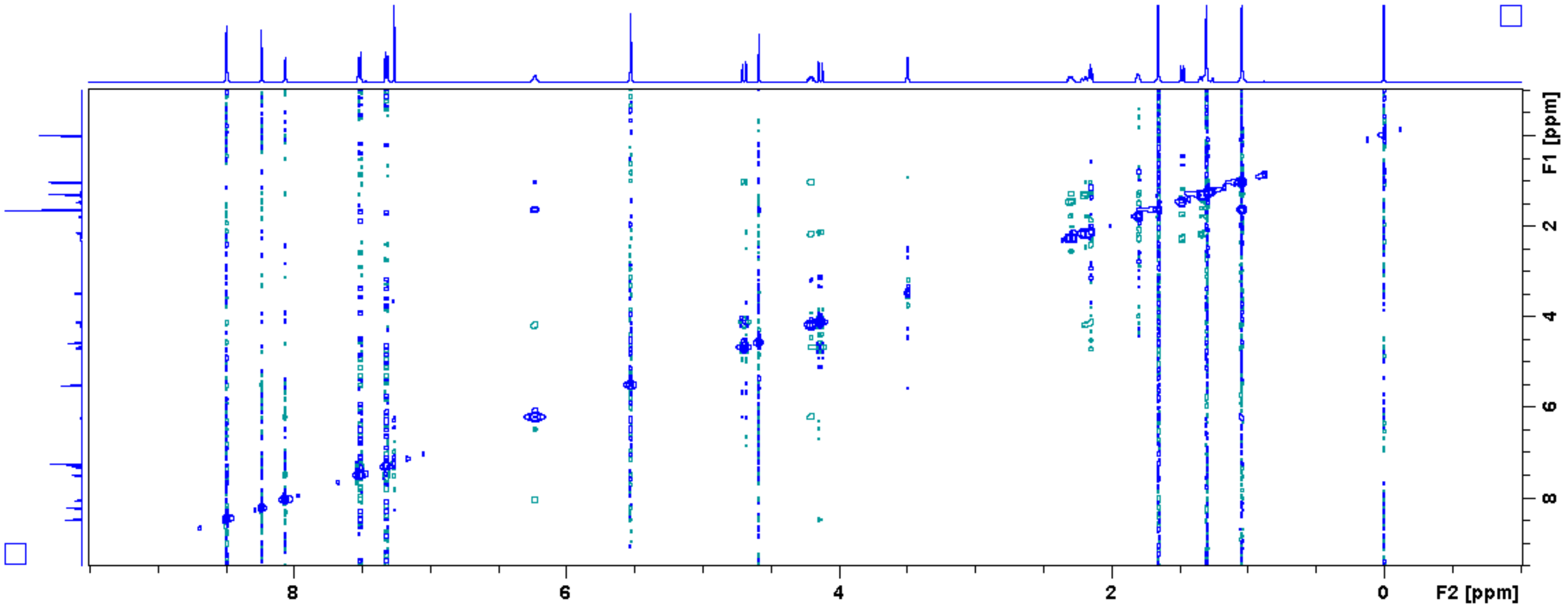

Figure S 178. HSQC-NMR of compound **(+)-33**

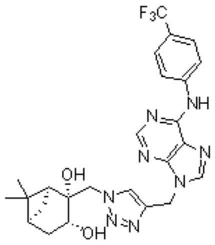

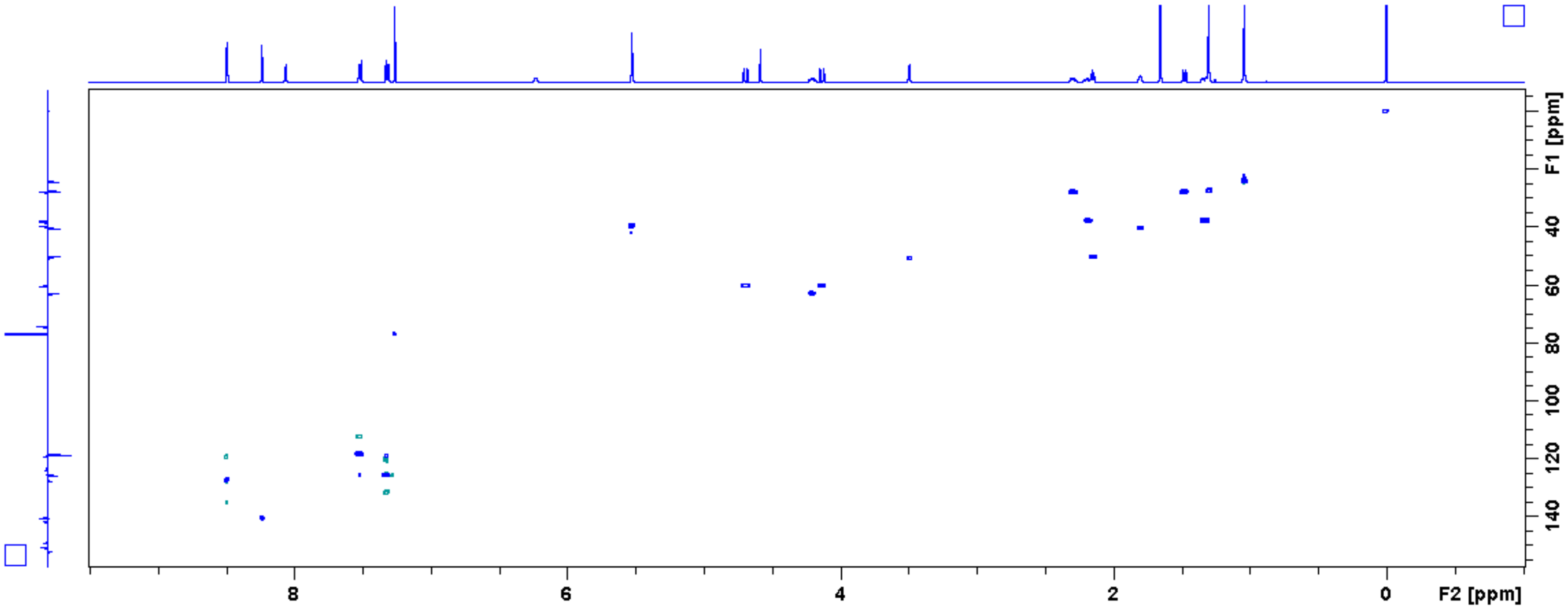

Figure S 179. HMBC-NMR of compound **(+)-33**

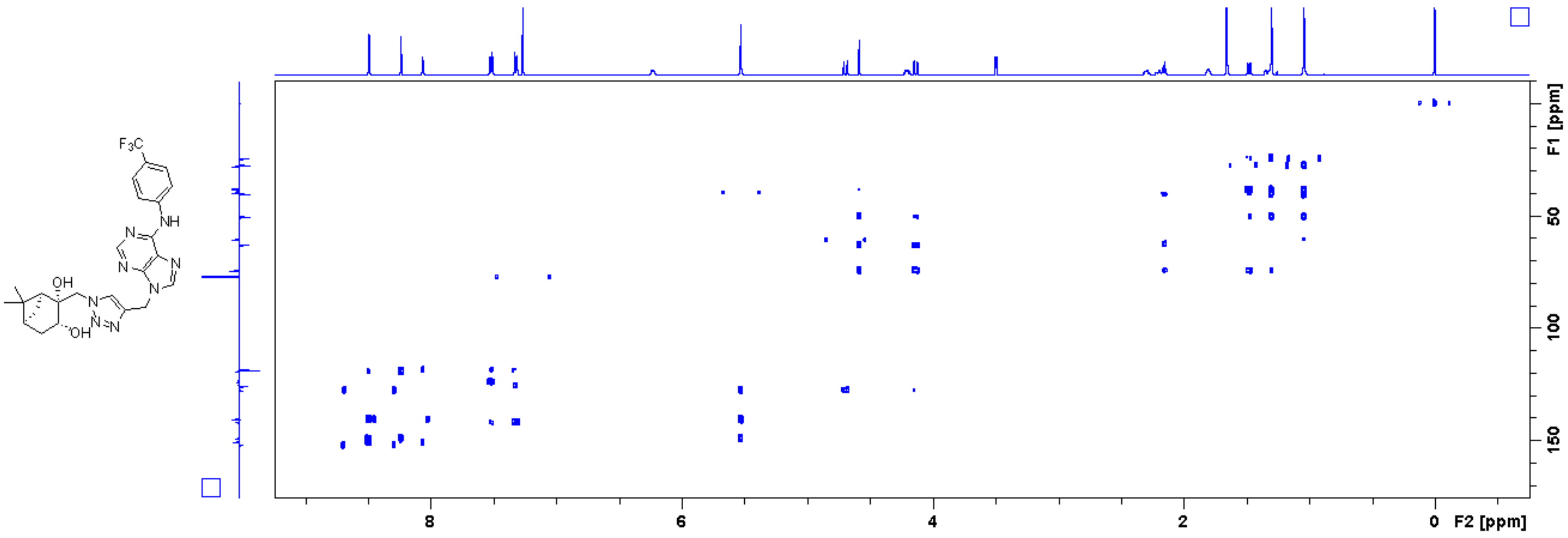

Figure S 180. ^{19}F-NMR of compound **(+)-33**

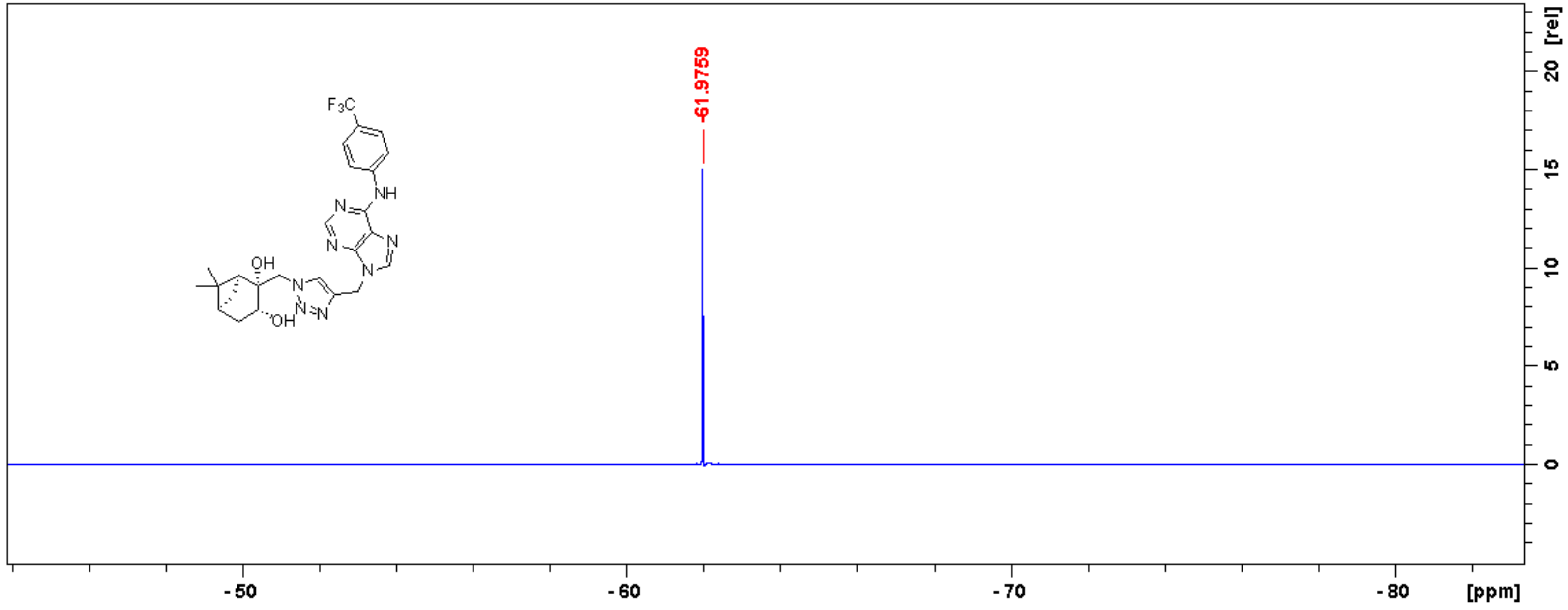

(1*R*,2*S*,3*S*,5*R*)-6,6-Dimethyl-2-((4-((6-((4-(trifluoromethyl)phenyl)amino)-9*H*-purin-9-yl)methyl)-1*H*-1,2,3-triazol-1-yl)methyl)bicyclo[3.1.1]heptane-2,3-diol **(–)-33**

Figure S 181. ^{1}H-NMR of compound **(–)-33**

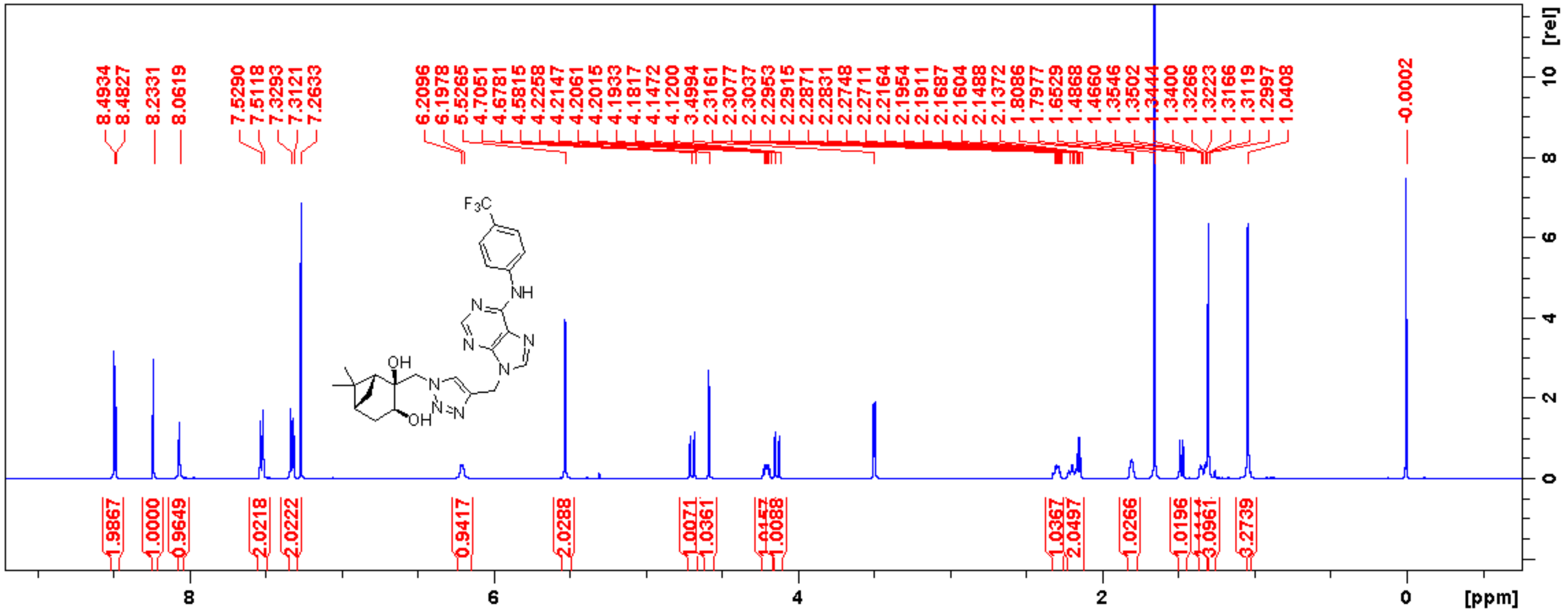

Figure S 182. ^{13}C-NMR of compound **(–)-33**

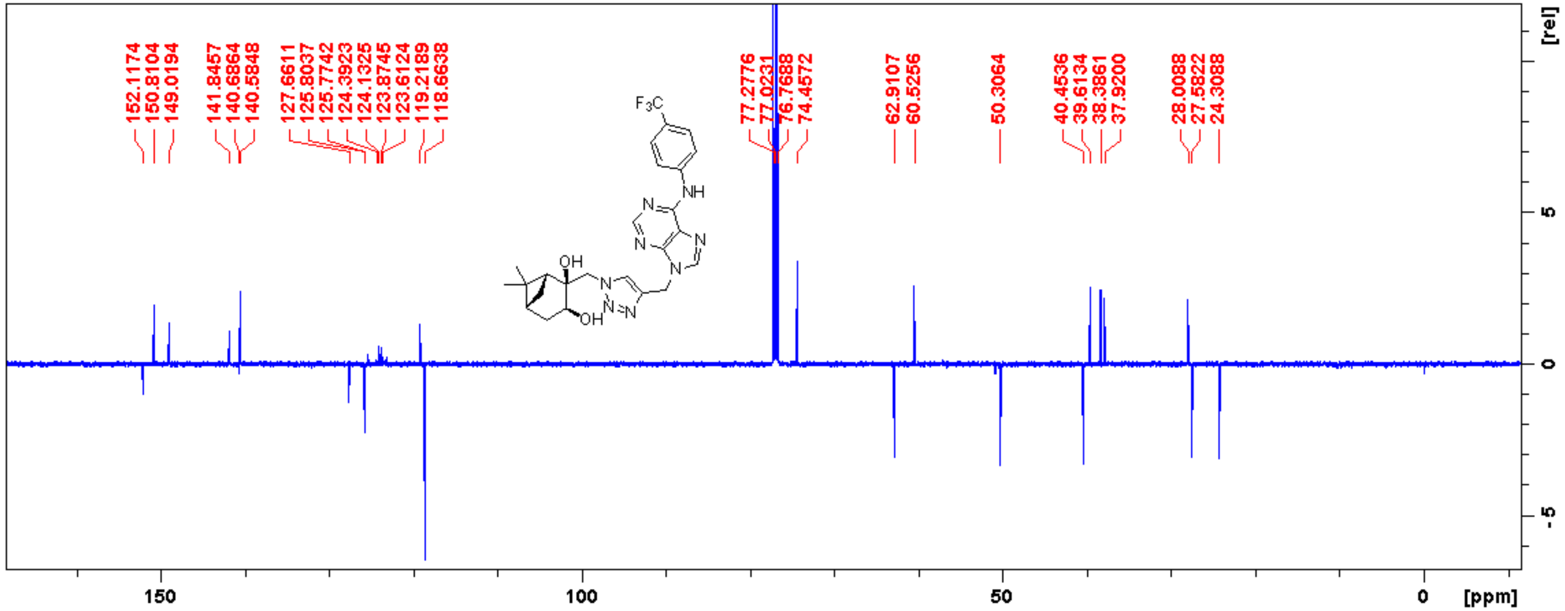

Figure S 183. COSY-NMR of compound **(–)-33**

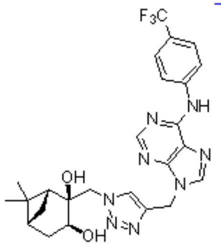

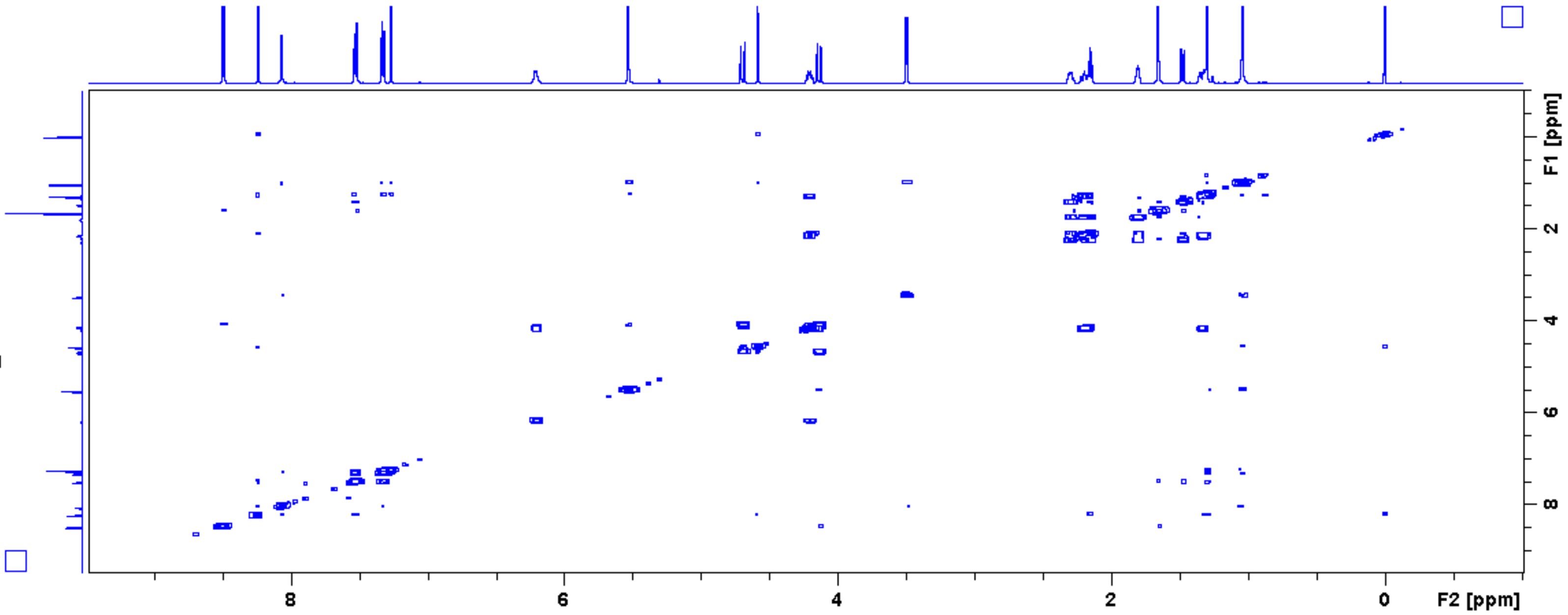

Figure S 184. NOESY-NMR of compound **(–)-33**

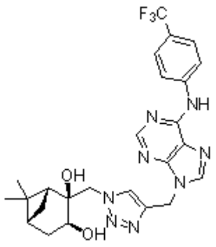

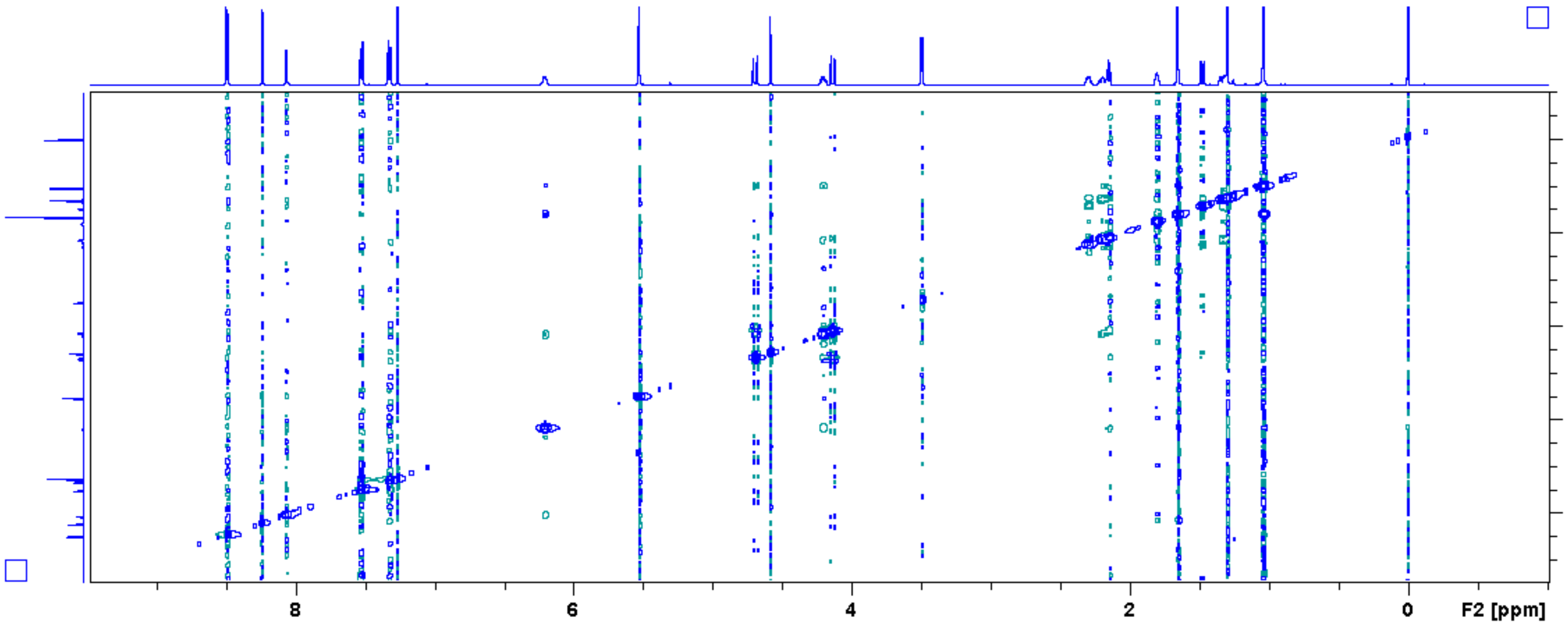

Figure S 185. HSQC-NMR of compound **(–)-33**

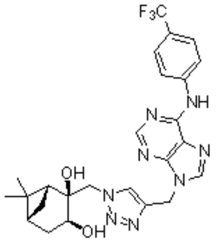

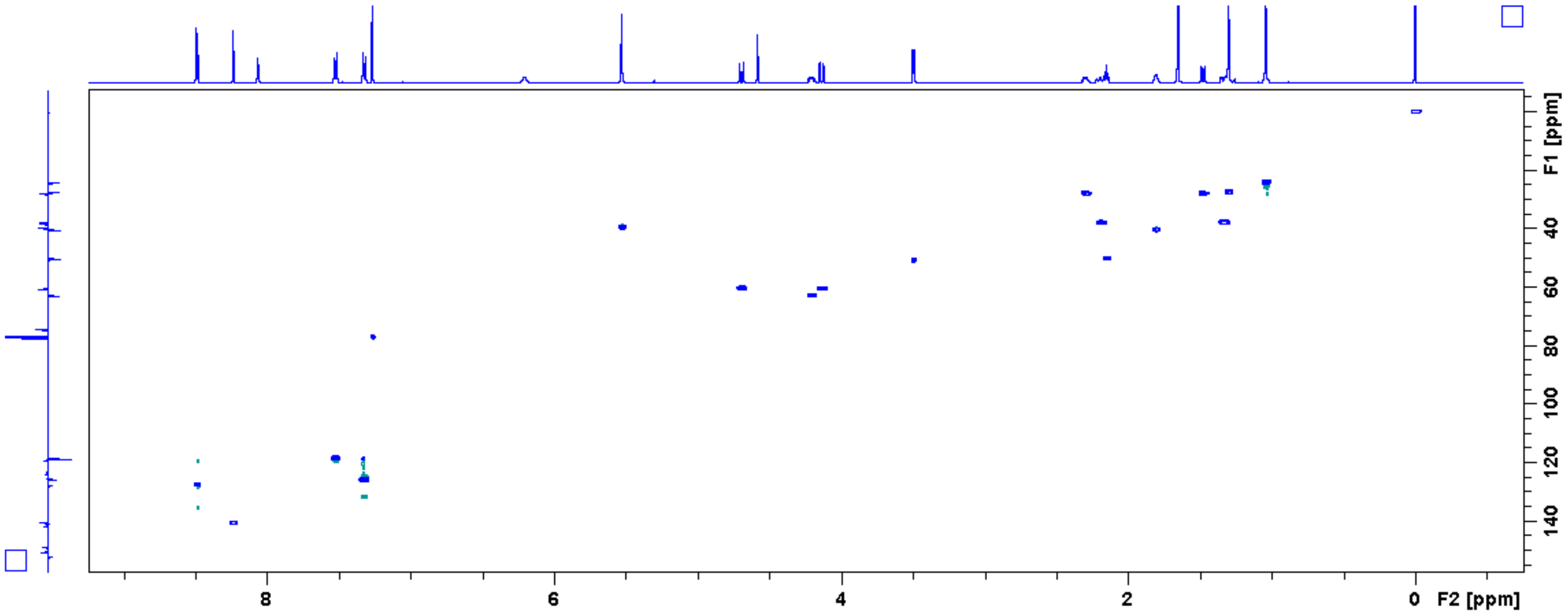

Figure S 186. HMBC-NMR of compound **(–)-33**

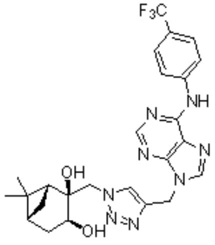

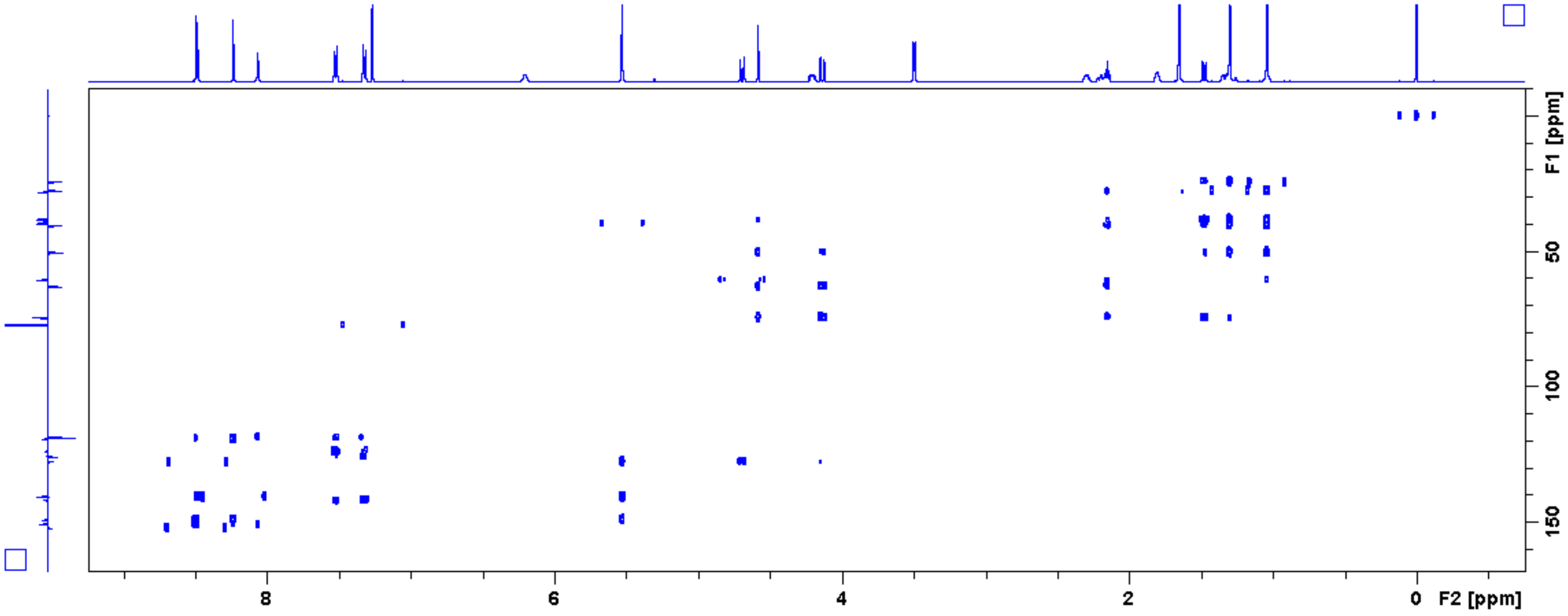

Figure S 187. ^{19}F-NMR of compound **(–)-33**

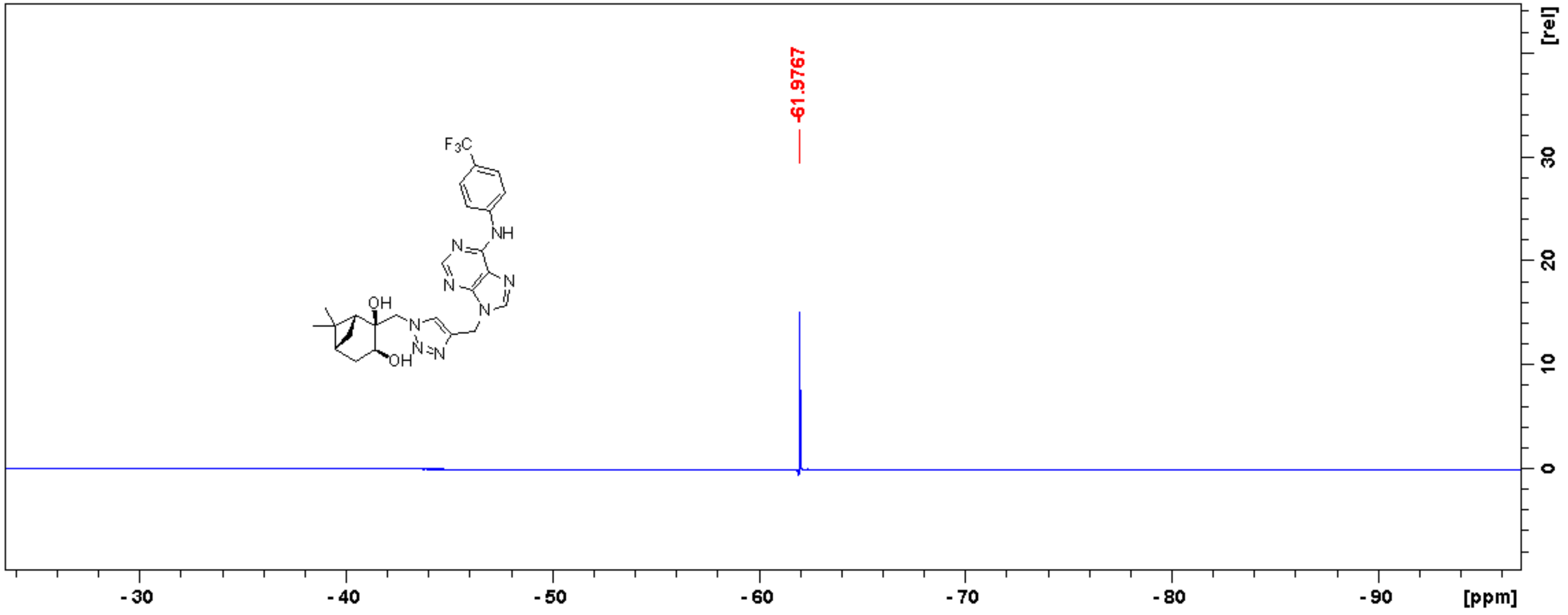

HRMS Spectra of compounds

6-Chloro-8-phenyl-9-(prop-2-yn-1-yl)-9*H*-purine (**16**)

Figure S 188. HRMS spectrum of compound **16**

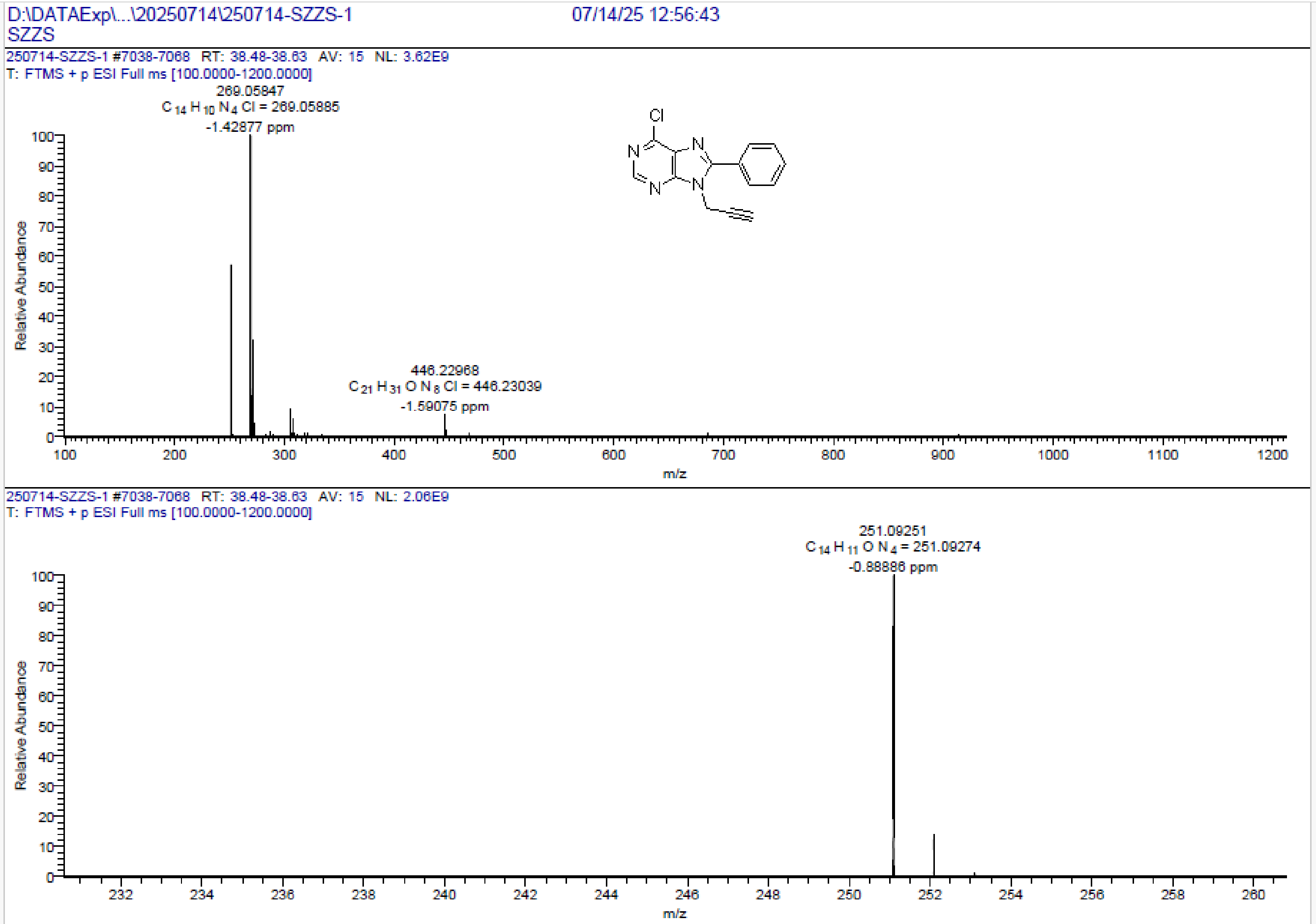

N-Benzyl-9-(prop-2-yn-1-yl)-9*H*-purin-6-amine (**18**)

Figure S 189. HRMS spectrum of compound **18**

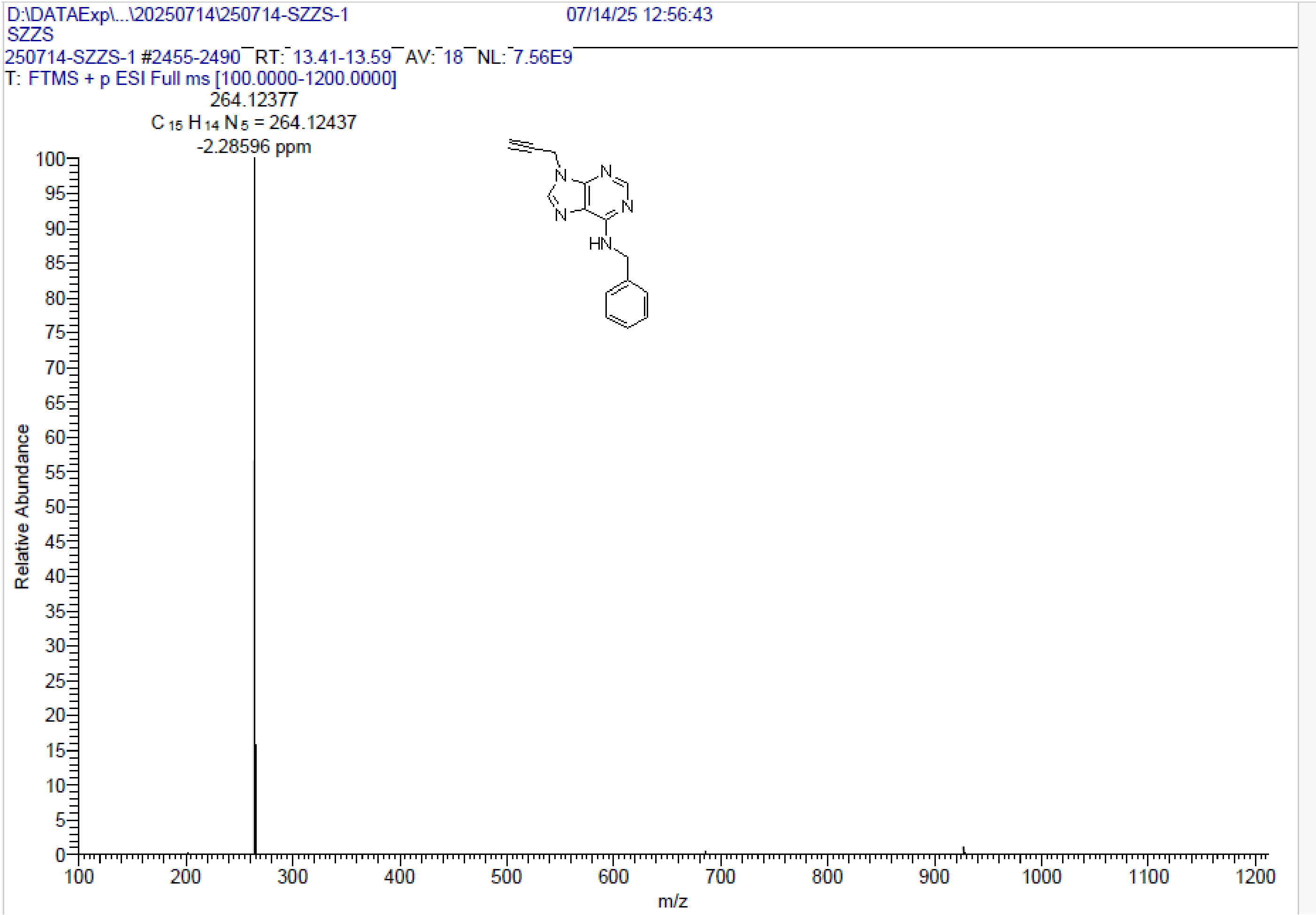

9-(prop-2-yn-1-yl)-*N*-(4-(trifluoromethyl)phenyl)-9*H*-purin-6-amine (**19**)

Figure S 190. HRMS spectrum of compound **19**

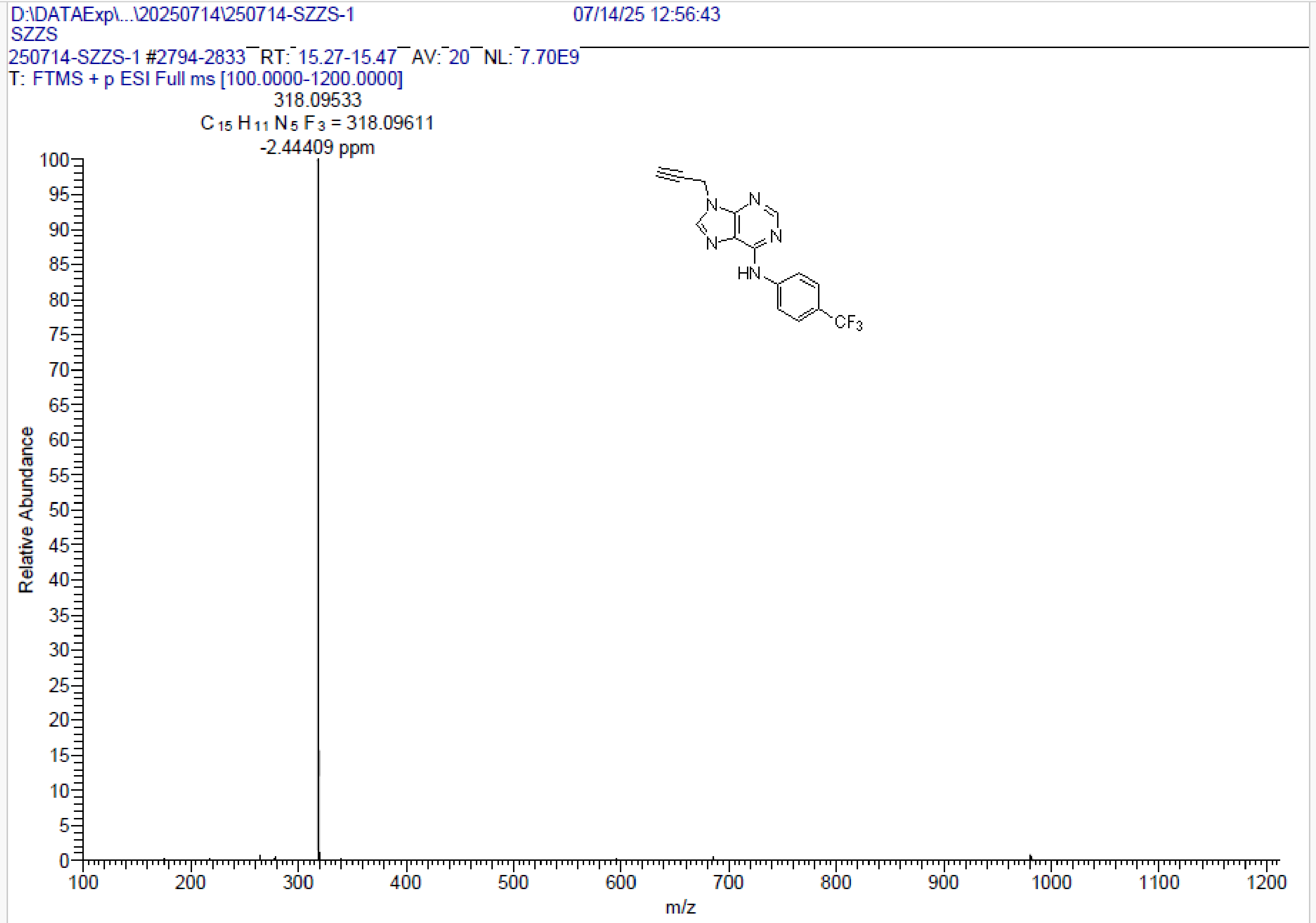

(1*S*,2*R*,3*R*,5*S*)-2-((4-(((2-Chloro-5-fluoropyrimidin-4-yl)amino)methyl)-1*H*-1,2,3-triazol-1-yl)methyl)-6,6-dimethylbicyclo[3.1.1]heptane-2,3-diol **(+)-20**

Figure S 191. HRMS spectrum of compound **(+)-20**

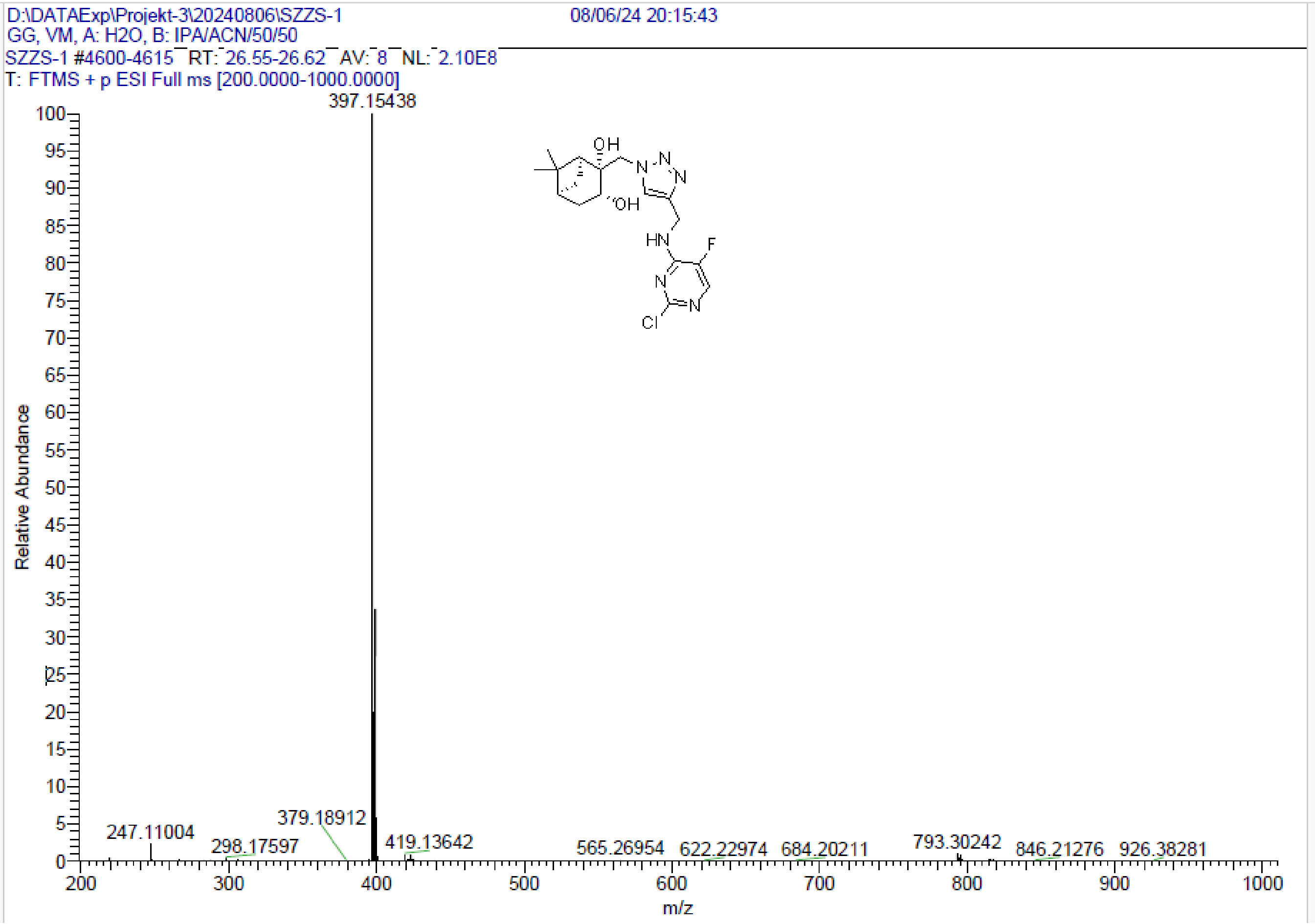

(1*R*,2*S*,3*S*,5*R*)-2-((4-(((2-Chloro-5-fluoropyrimidin-4-yl)amino)methyl)-1*H*-1,2,3-triazol-1-yl)methyl)-6,6-dimethylbicyclo[3.1.1]heptane-2,3-diol **(–)-20**

Figure S 192. HRMS spectrum of compound **(–)-20**

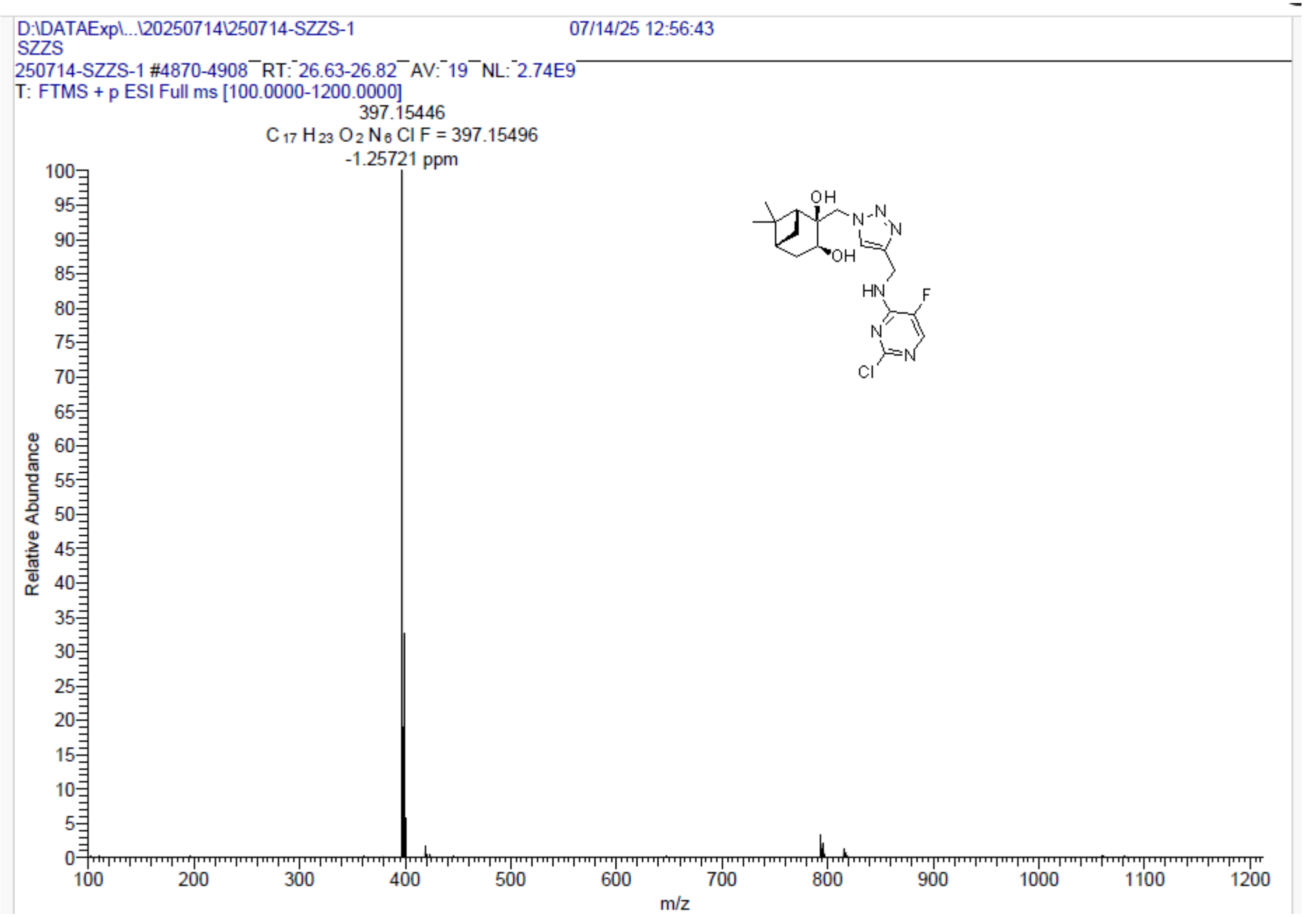

(1*S*,2*R*,3*R*,5*S*)-2-((4-(((2,5-Dichloropyrimidin-4-yl)amino)methyl)-1*H*-1,2,3-triazol-1-yl)methyl)-6,6-dimethylbicyclo[3.1.1]heptane-2,3-diol **(+)-21**

Figure S 193. HRMS spectrum of compound **(+)-21**

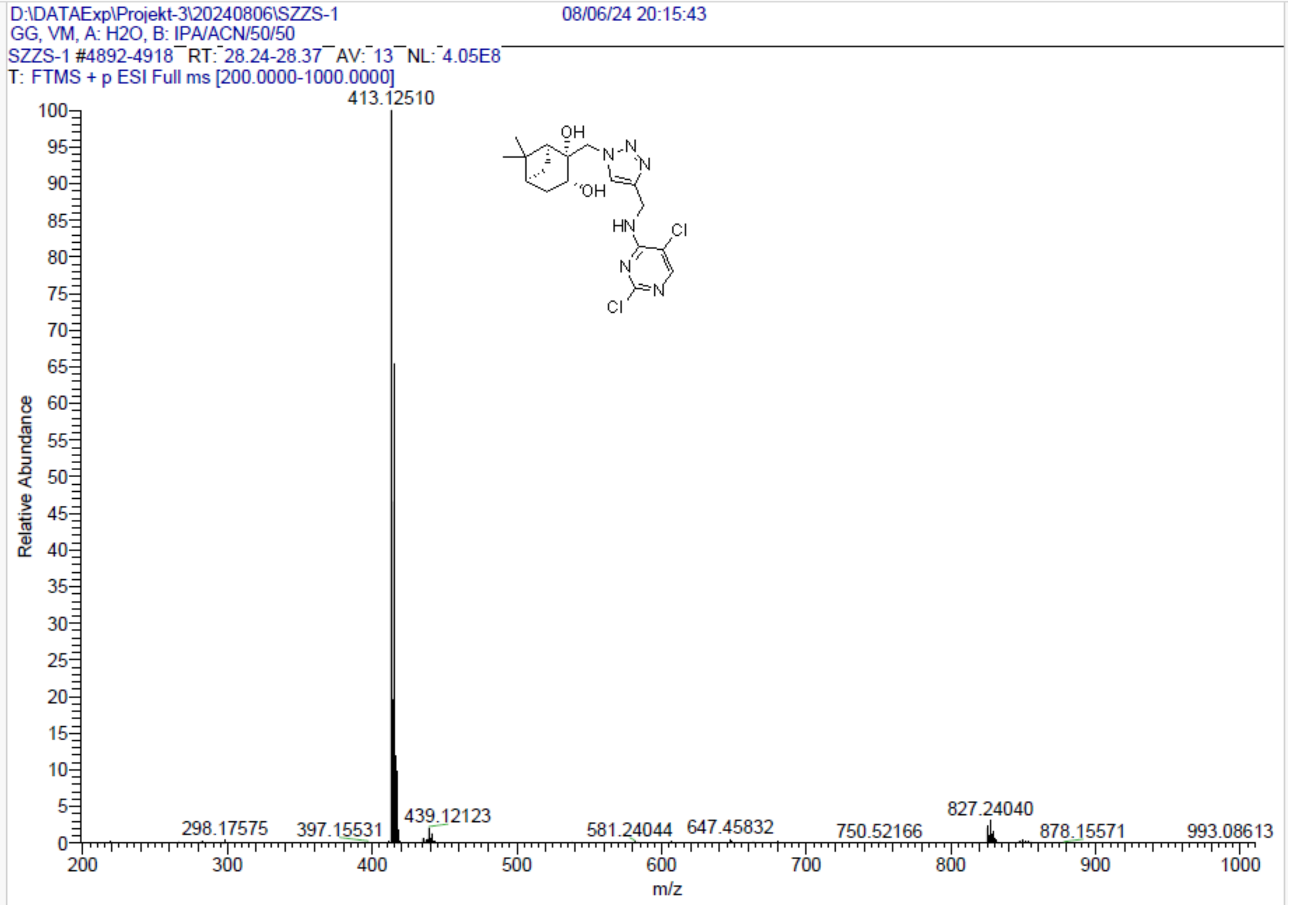

(1*R*,2*S*,3*S*,5*R*)-2-((4-(((2,5-Dichloropyrimidin-4-yl)amino)methyl)-1*H*-1,2,3-triazol-1-yl)methyl)-6,6-dimethylbicyclo[3.1.1]heptane-2,3-diol **(–)-21**

Figure S 194. HRMS spectrum of compound **(–)-21**

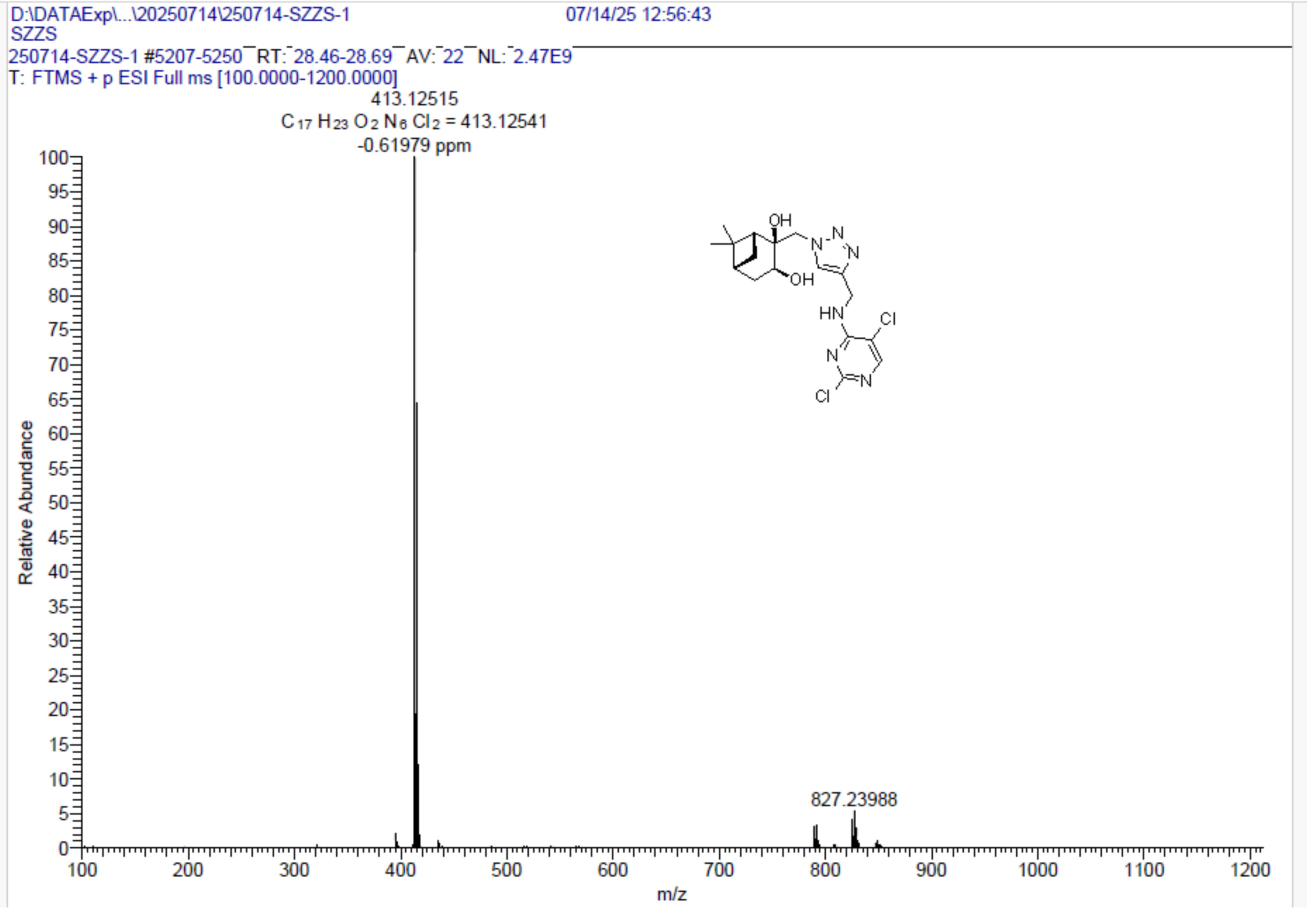

(1*S*,2*R*,3*R*,5*S*)-2-((4-(((5-Amino-6-chloropyrimidin-4-yl)amino)methyl)-1*H*-1,2,3-triazol-1-yl)methyl)-6,6-dimethylbicyclo[3.1.1]heptane-2,3-diol **(+)-22**

Figure S 195. HRMS spectrum of compound **(+)-22**

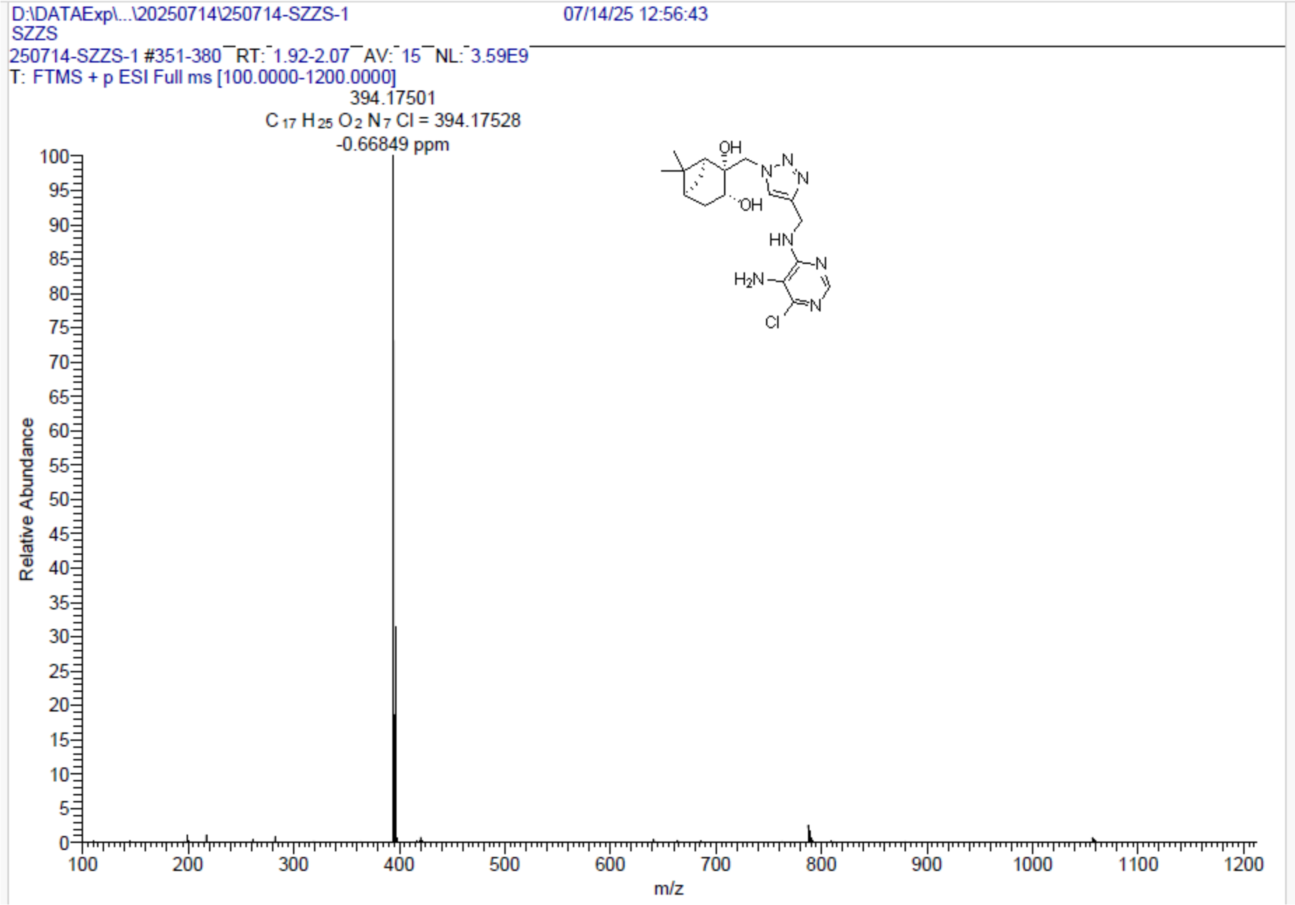

(1*R*,2*S*,3*S*,5*R*)-2-((4-(((5-Amino-6-chloropyrimidin-4-yl)amino)methyl)-1*H*-1,2,3-triazol-1-yl)methyl)-6,6-dimethylbicyclo[3.1.1]heptane-2,3-diol **(–)-22**

Figure S 196. HRMS spectrum of compound **(–)-22**

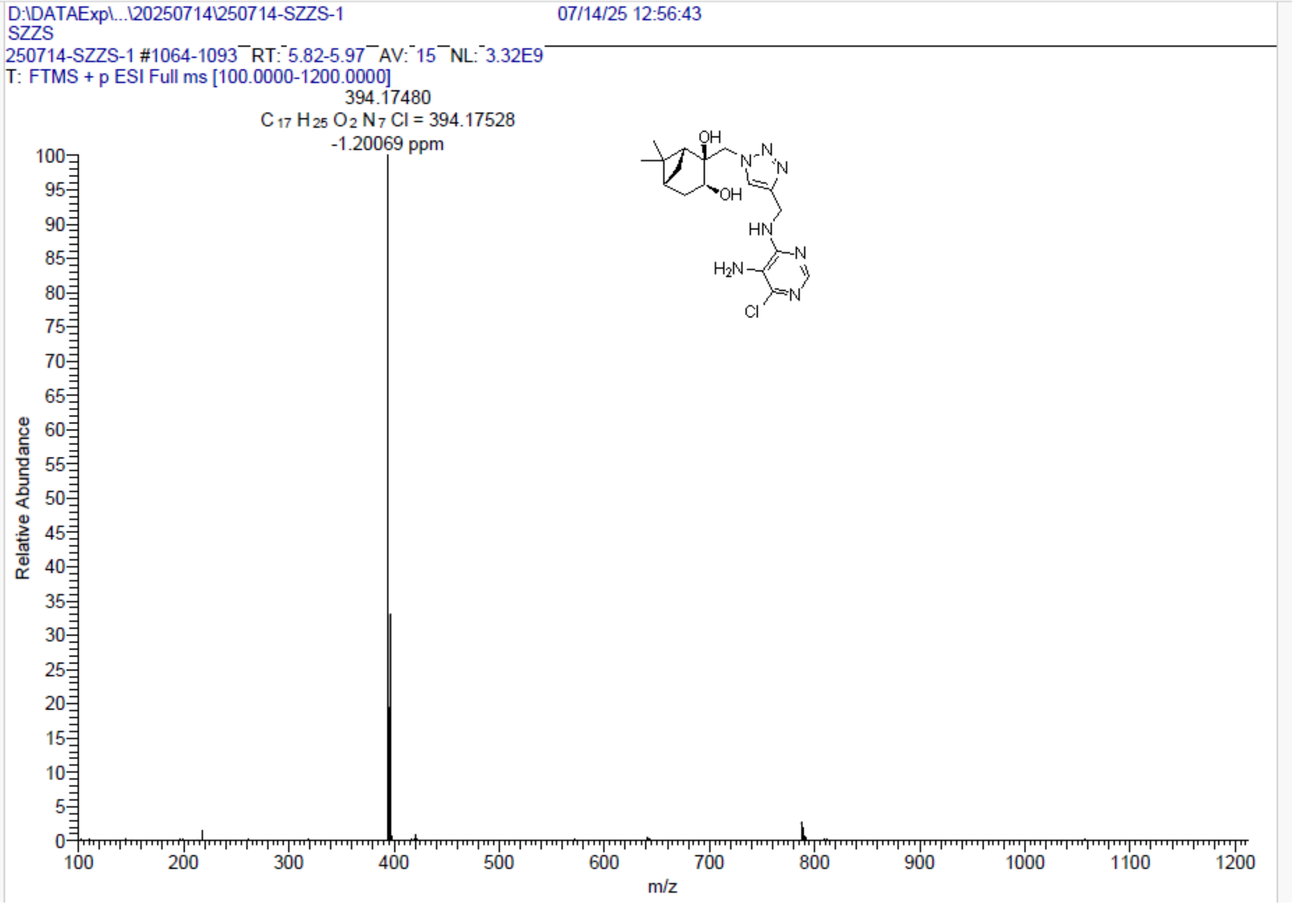

(1*S*,2*R*,3*R*,5*S*)-2-((4-(((5-Fluoro-2-((4-(trifluoromethyl)phenyl)amino)pyrimidin-4-yl)amino)methyl)-1*H*-1,2,3-triazol-1-yl)methyl)-6,6-dimethylbicyclo[3.1.1]heptane-2,3-diol **(+)-23**

Figure S 197. HRMS spectrum of compound **(+)-23**

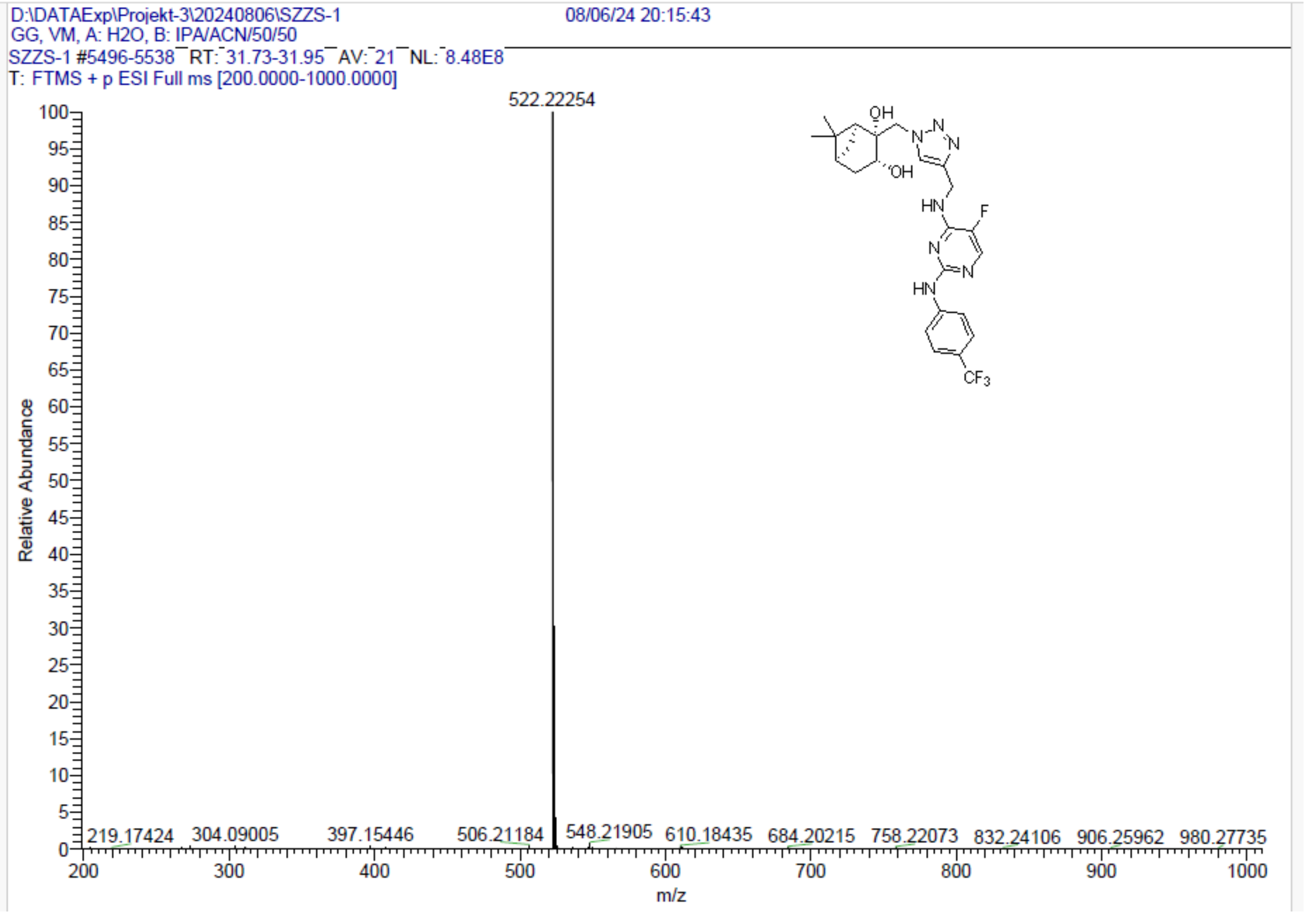

(1*R*,2*S*,3*S*,5*R*)-2-((4-(((5-Fluoro-2-((4-(trifluoromethyl)phenyl)amino)pyrimidin-4-yl)amino)methyl)-1*H*-1,2,3-triazol-1-yl)methyl)-6,6-dimethylbicyclo[3.1.1]heptane-2,3-diol **(–)-23**

Figure S 198. HRMS spectrum of compound **(–)-23**

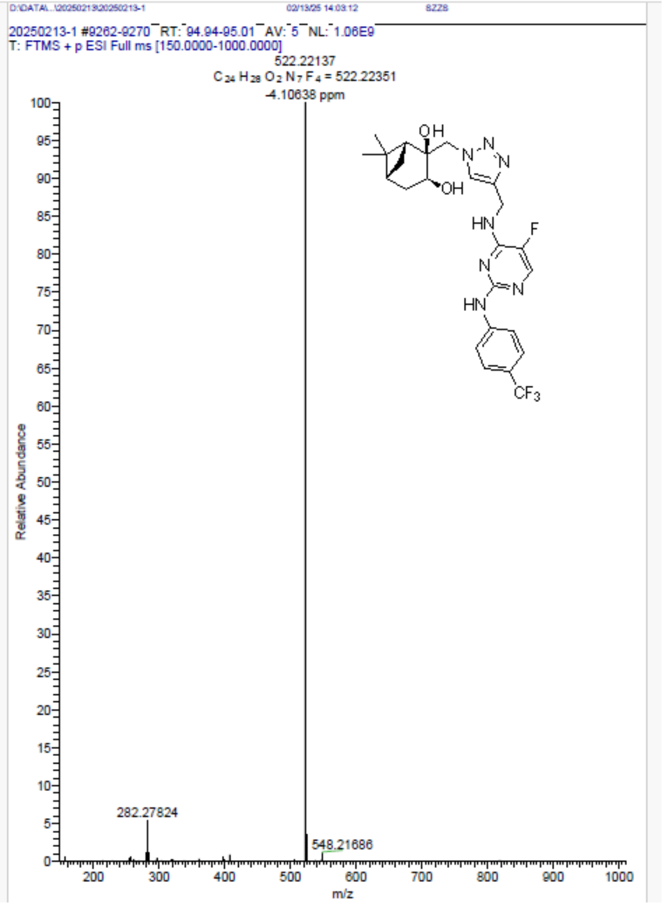

(1*S*,2*R*,3*R*,5*S*)-2-((4-(((5-Chloro-2-((4-(trifluoromethyl)phenyl)amino)pyrimidin-4-yl)amino)methyl)-1*H*-1,2,3-triazol-1-yl)methyl)-6,6-dimethylbicyclo[3.1.1]heptane-2,3-diol **(+)-24**

Figure S 199. HRMS spectrum of compound **(+)-24**

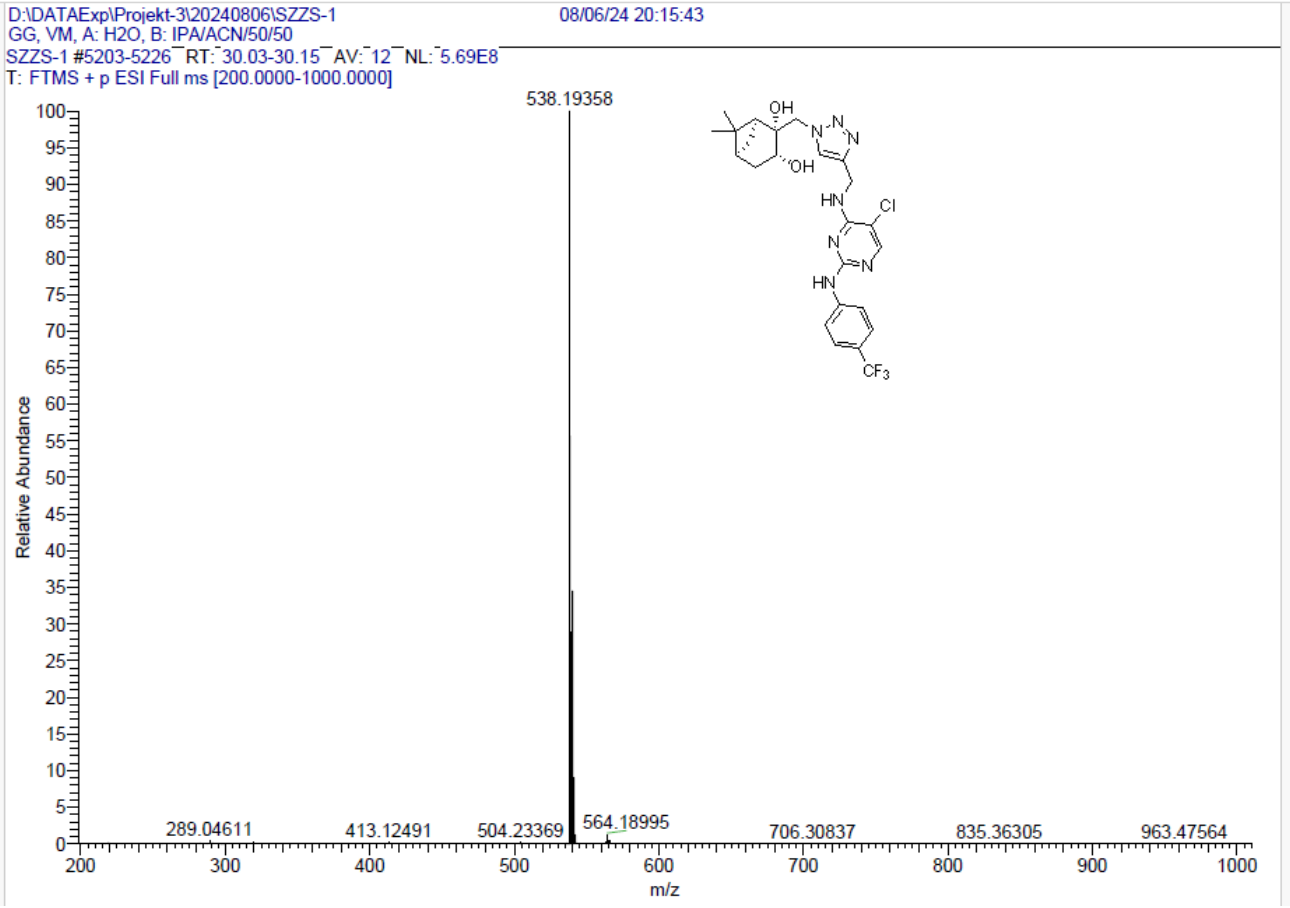

(1*R*,2*S*,3*S*,5*R*)-2-((4-(((5-chloro-2-((4-(trifluoromethyl)phenyl)amino)pyrimidin-4-yl)amino)methyl)-1H-1,2,3-triazol-1-yl)methyl)-6,6-dimethylbicyclo[3.1.1]heptane-2,3-diol **(–)-24**

Figure S 200. HRMS spectrum of compound **(–)-24**

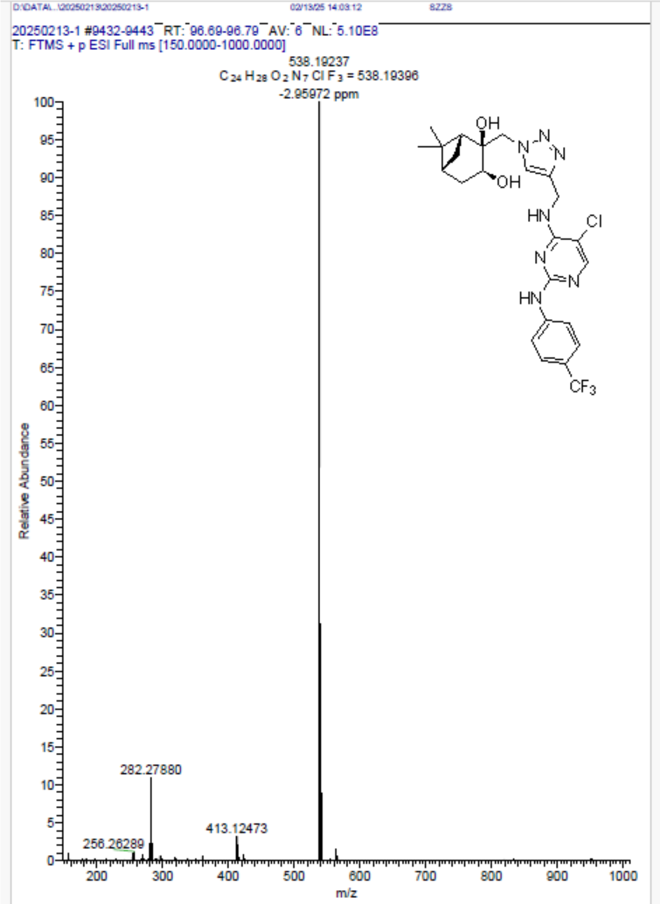

(1*S*,2*R*,3*R*,5*S*)-2-((4-(((5-Fluoro-2-((1-methyl-1H-pyrazol-4-yl)amino)pyrimidin-4-yl)amino)methyl)-1*H*-1,2,3-triazol-1-yl)methyl)-6,6-dimethylbicyclo[3.1.1]heptane-2,3-diol **(+)-25**

Figure S 201. HRMS spectrum of compound **(+)-25**

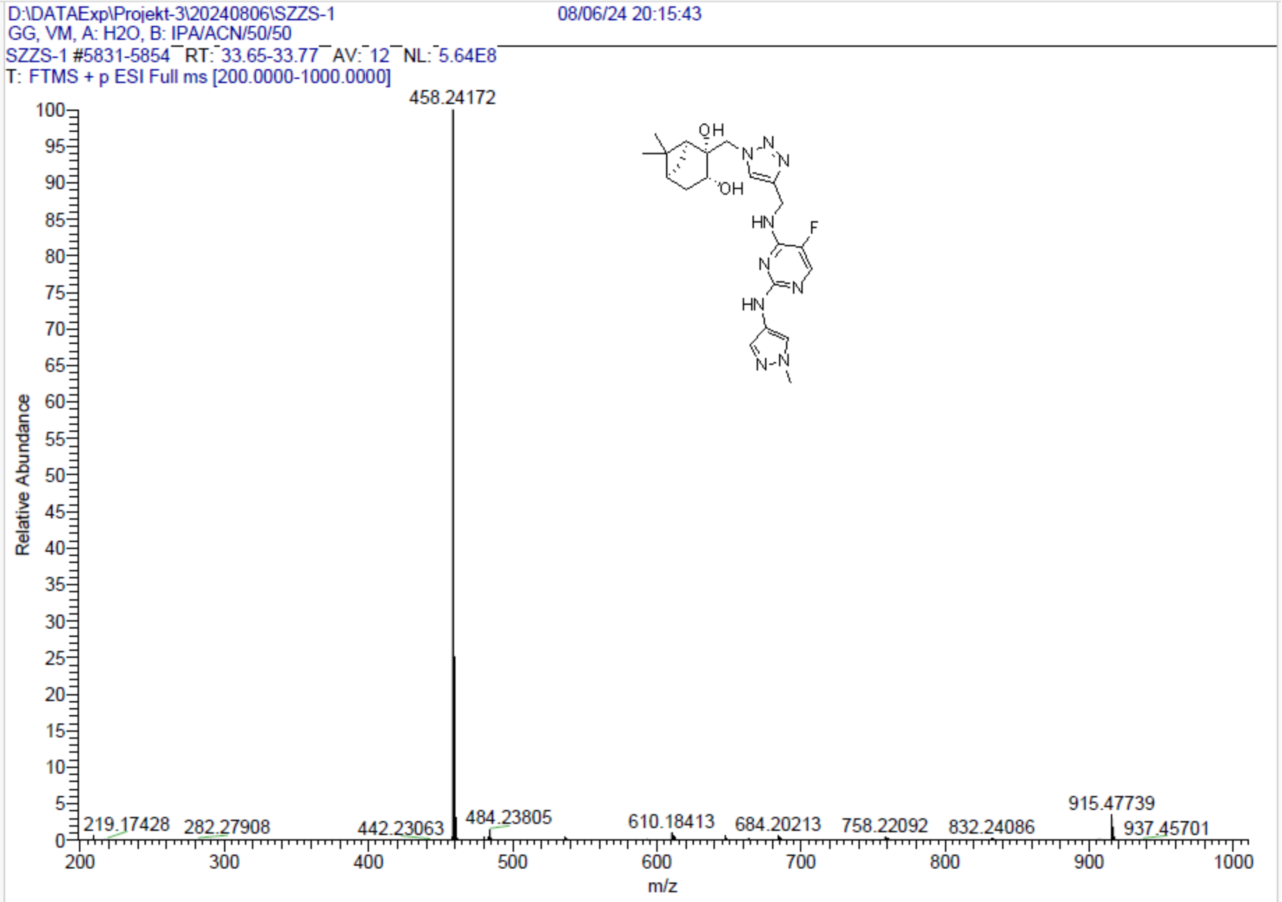

(1*R*,2*S*,3*S*,5*R*)-2-((4-(((5-fluoro-2-((1-methyl-1*H*-pyrazol-4-yl)amino)pyrimidin-4-yl)amino)methyl)-1H-1,2,3-triazol-1-yl)methyl)-6,6-dimethylbicyclo[3.1.1]heptane-2,3-diol **(–)-25**

Figure S 202. HRMS spectrum of compound **(–)-25**

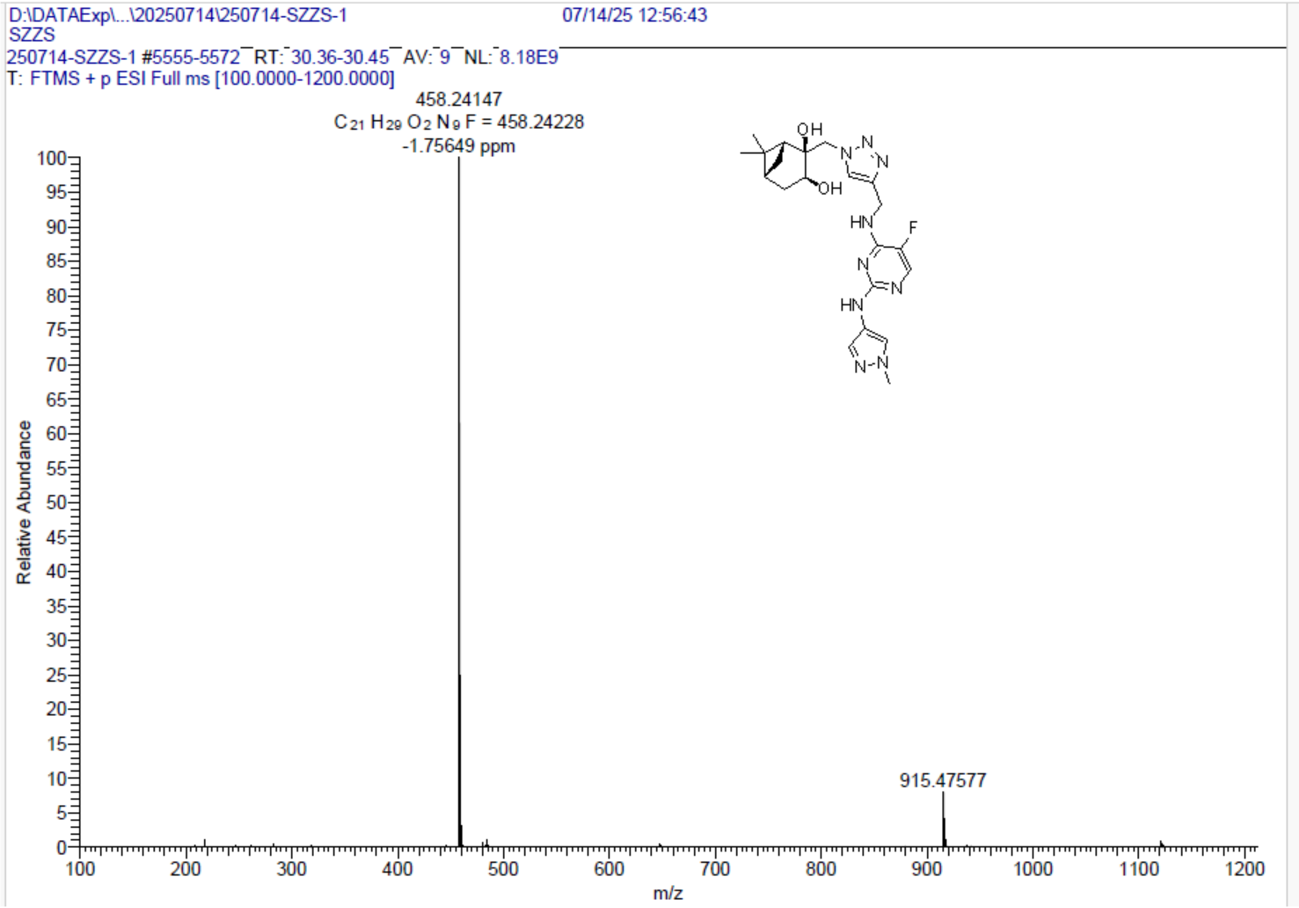

(1*S*,2*R*,3*R*,5*S*)-2-((4-(((5-Chloro-2-((1-methyl-1*H*-pyrazol-4-yl)amino)pyrimidin-4-yl)amino)methyl)-1*H*-1,2,3-triazol-1-yl)methyl)-6,6-dimethylbicyclo[3.1.1]heptane-2,3-diol **(+)-26**

Figure S 203. HRMS spectrum of compound **(+)-26**

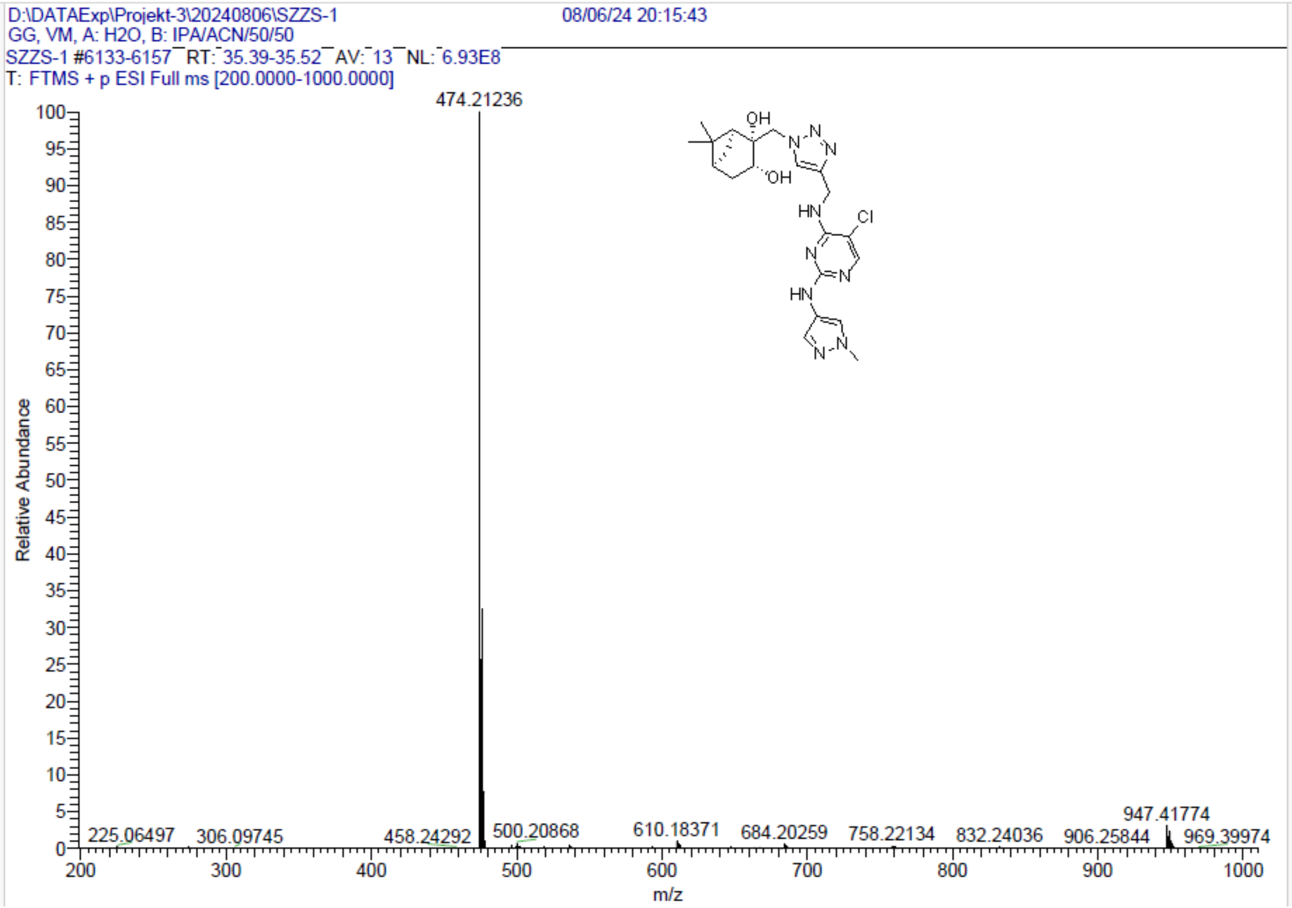

(1*R*,2*S*,3*S*,5*R*)-2-((4-(((5-chloro-2-((1-methyl-1*H*-pyrazol-4-yl)amino)pyrimidin-4-yl)amino)methyl)-1*H*-1,2,3-triazol-1-yl)methyl)-6,6-dimethylbicyclo[3.1.1]heptane-2,3-diol **(–)-26**

Figure S 204. HRMS spectrum of compound **(–)-26**

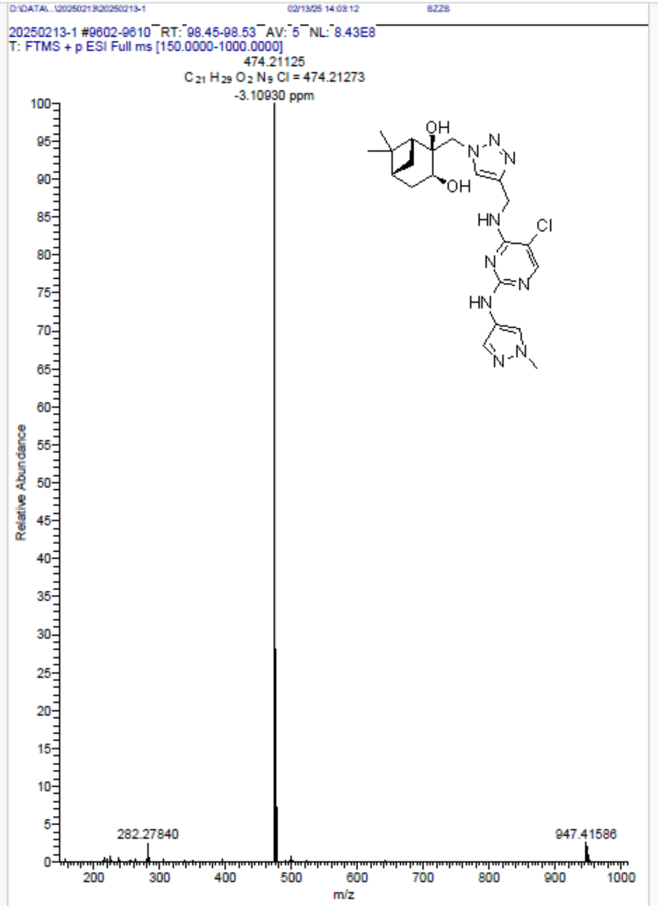

methyl 4-((5-Chloro-4-(((1-(((1*S*,2*R*,3*R*,5*S*)-2,3-dihydroxy-6,6-dimethylbicyclo[3.1.1]heptan-2-yl)methyl)-1*H*-1,2,3-triazol-4-yl)methyl)amino)pyrimidin-2-yl)amino)benzoate **(+)-27**

Figure S 205. HRMS spectrum of compound **(+)-27**

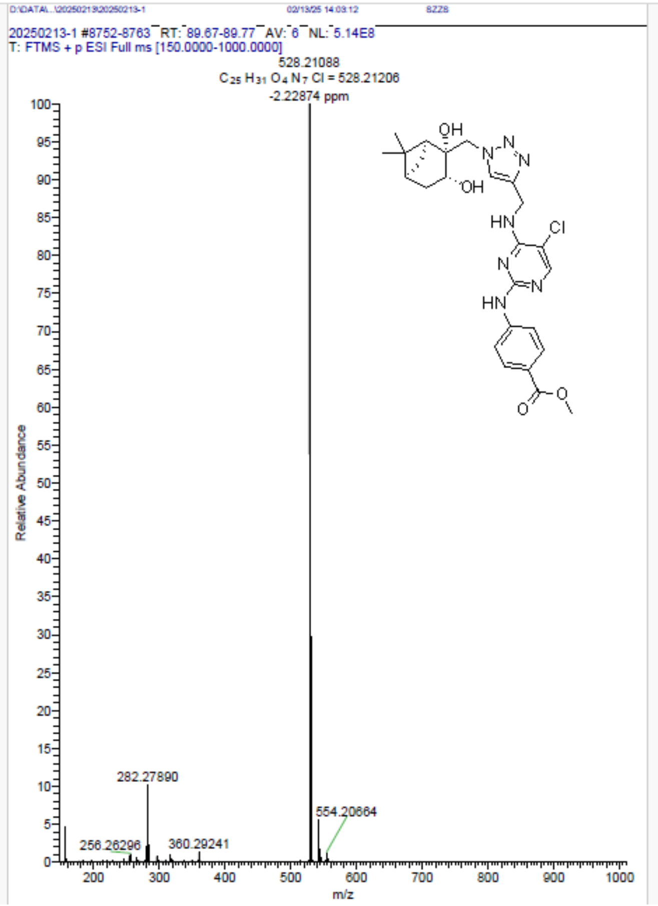

methyl 4-((5-chloro-4-(((1-(((1*R*,2*S*,3*S*,5*R*)-2,3-dihydroxy-6,6-dimethylbicyclo[3.1.1]heptan-2-yl)methyl)-1*H*-1,2,3-triazol-4-yl)methyl)amino)pyrimidin-2-yl)amino)benzoate **(–)-27**

Figure S 206. HRMS spectrum of compound **(–)-27**

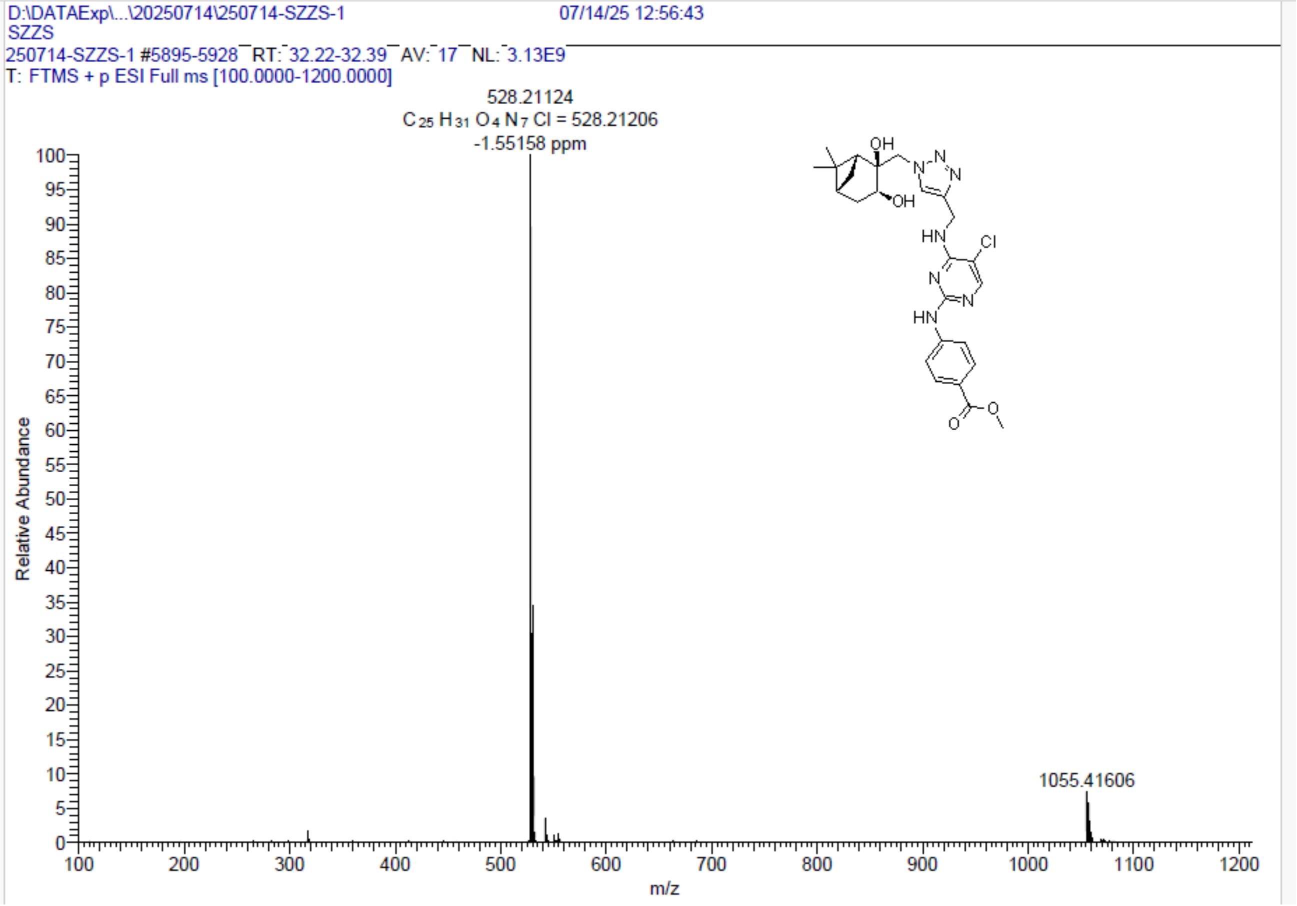

(1*S*,2*R*,3*R*,5*S*)-2-((4-(((5-Fluoro-2-((4-morpholinophenyl)amino)pyrimidin-4-yl)amino)methyl)-1*H*-1,2,3-triazol-1-yl)methyl)-6,6-dimethylbicyclo[3.1.1]heptane-2,3-diol **(+)-28**

Figure S 207. HRMS spectrum of compound **(+)-28**

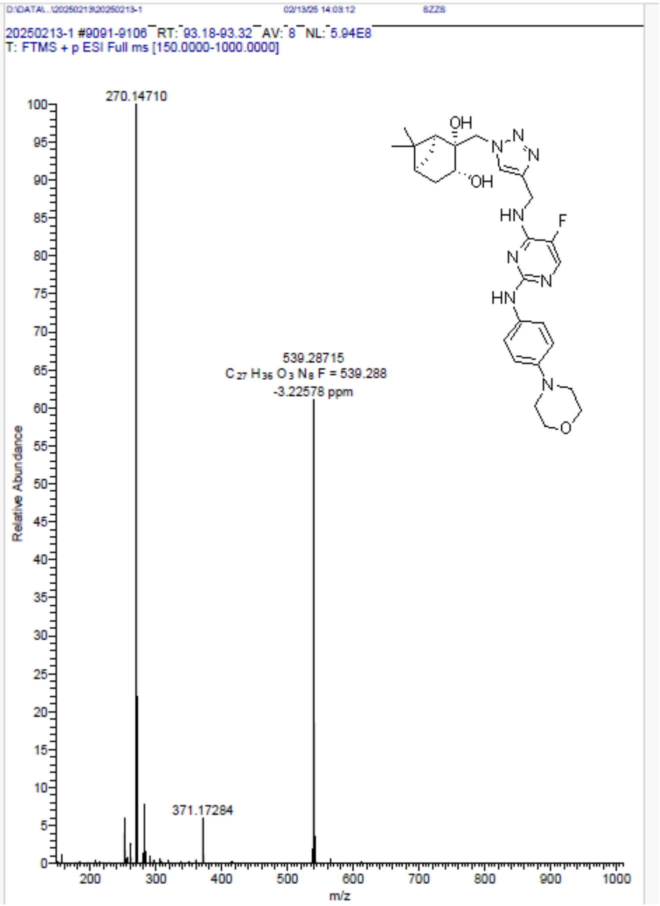

(1*R*,2*S*,3*S*,5*R*)-2-((4-(((5-Fluoro-2-((4-morpholinophenyl)amino)pyrimidin-4-yl)amino)methyl)-1*H*-1,2,3-triazol-1-yl)methyl)-6,6-dimethylbicyclo[3.1.1]heptane-2,3-diol **(–)-28**

Figure S 208. HRMS spectrum of compound **(–)-28**

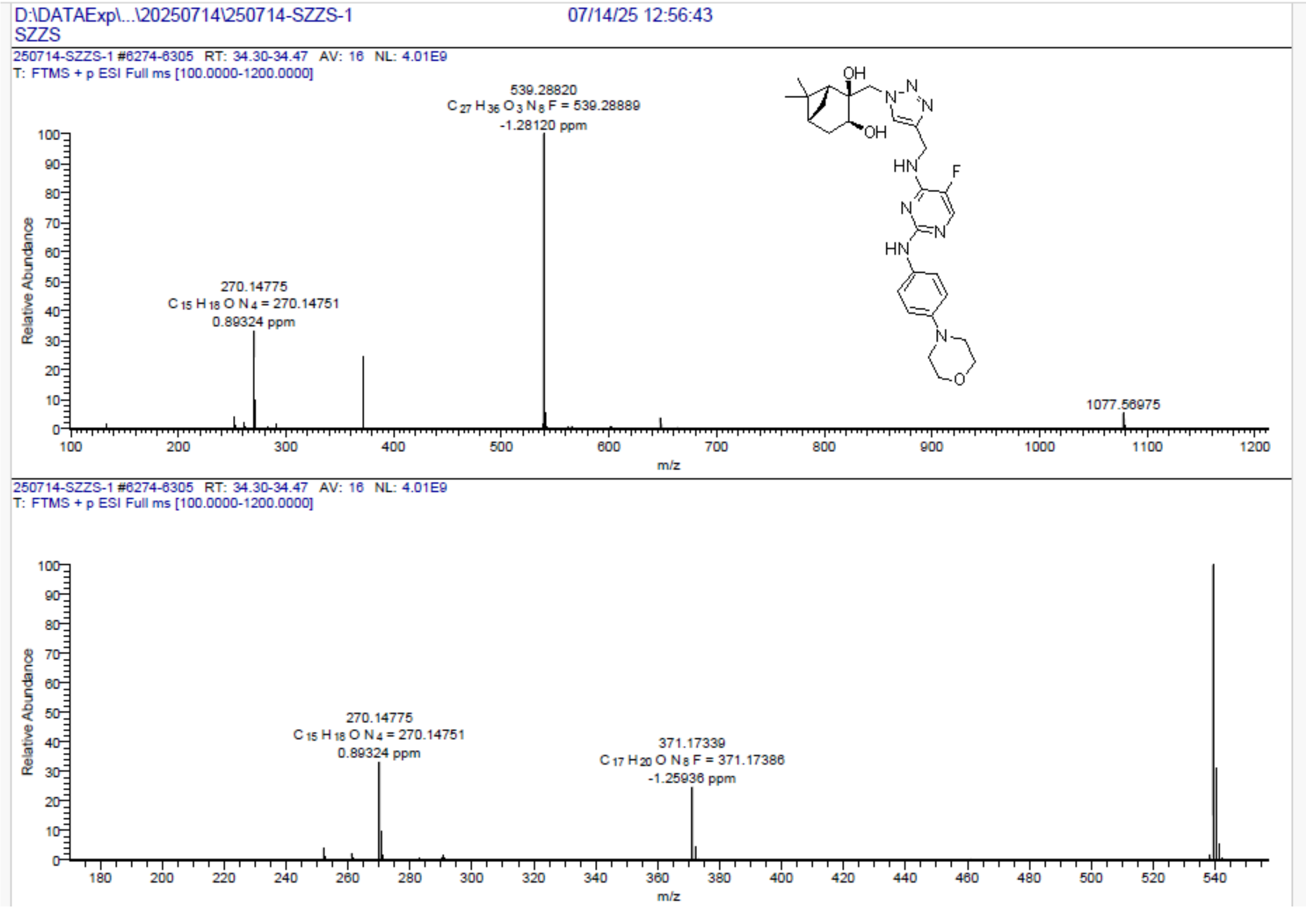

(1*S*,2*R*,3*R*,5*S*)-2-((4-((6-Chloro-9*H*-purin-9-yl)methyl)-1*H*-1,2,3-triazol-1-yl)methyl)-6,6 dimethylbicyclo[3.1.1]heptane-2,3-diol **(+)-29**

Figure S 209. HRMS spectrum of compound **(+)-29**

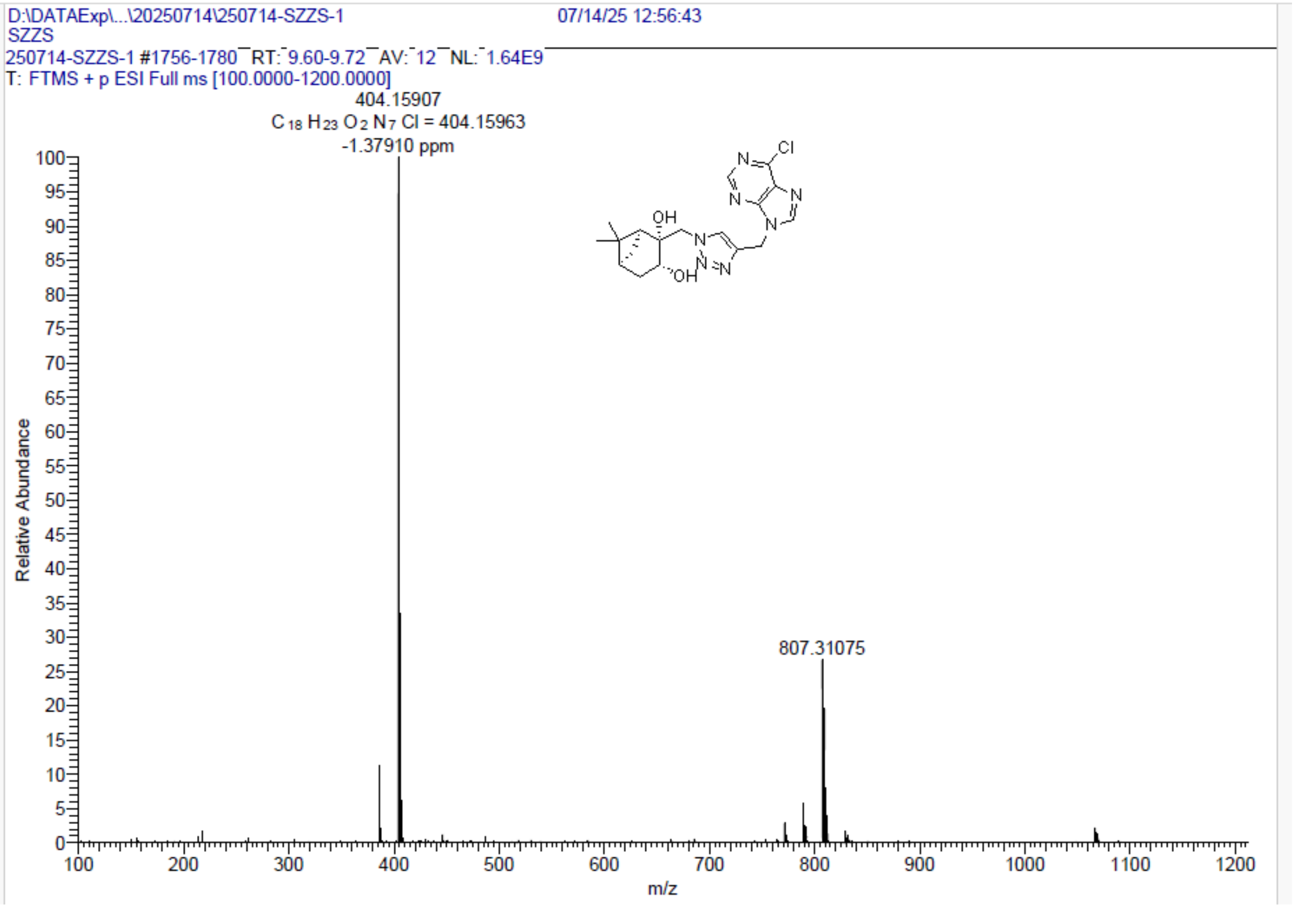

(1*R*,2*S*,3*S*,5*R*)-2-((4-((6-Chloro-9*H*-purin-9-yl)methyl)-1*H*-1,2,3-triazol-1-yl)methyl)-6,6-dimethylbicyclo[3.1.1]heptane-2,3-diol **(–)-29**

Figure S 210. HRMS spectrum of compound **(–)-29**

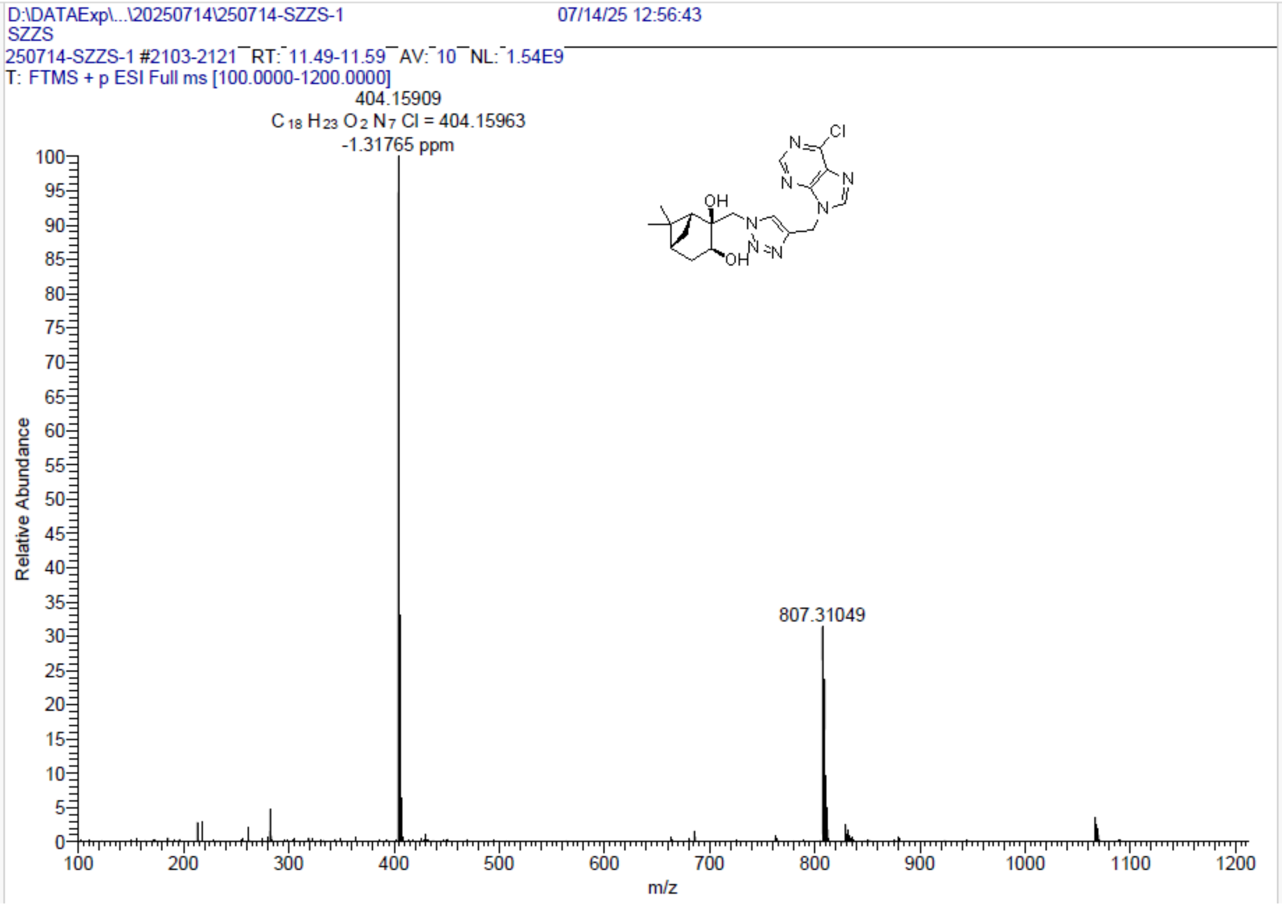

(1*S*,2*R*,3*R*,5*S*)-2-((4-((6-Chloro-8-phenyl-9*H*-purin-9-yl)methyl)-1*H*-1,2,3-triazol-1-yl)methyl)-6,6-dimethylbicyclo[3.1.1]heptane-2,3-diol **(+)-30**

Figure S 211. HRMS spectrum of compound **(+)-30**

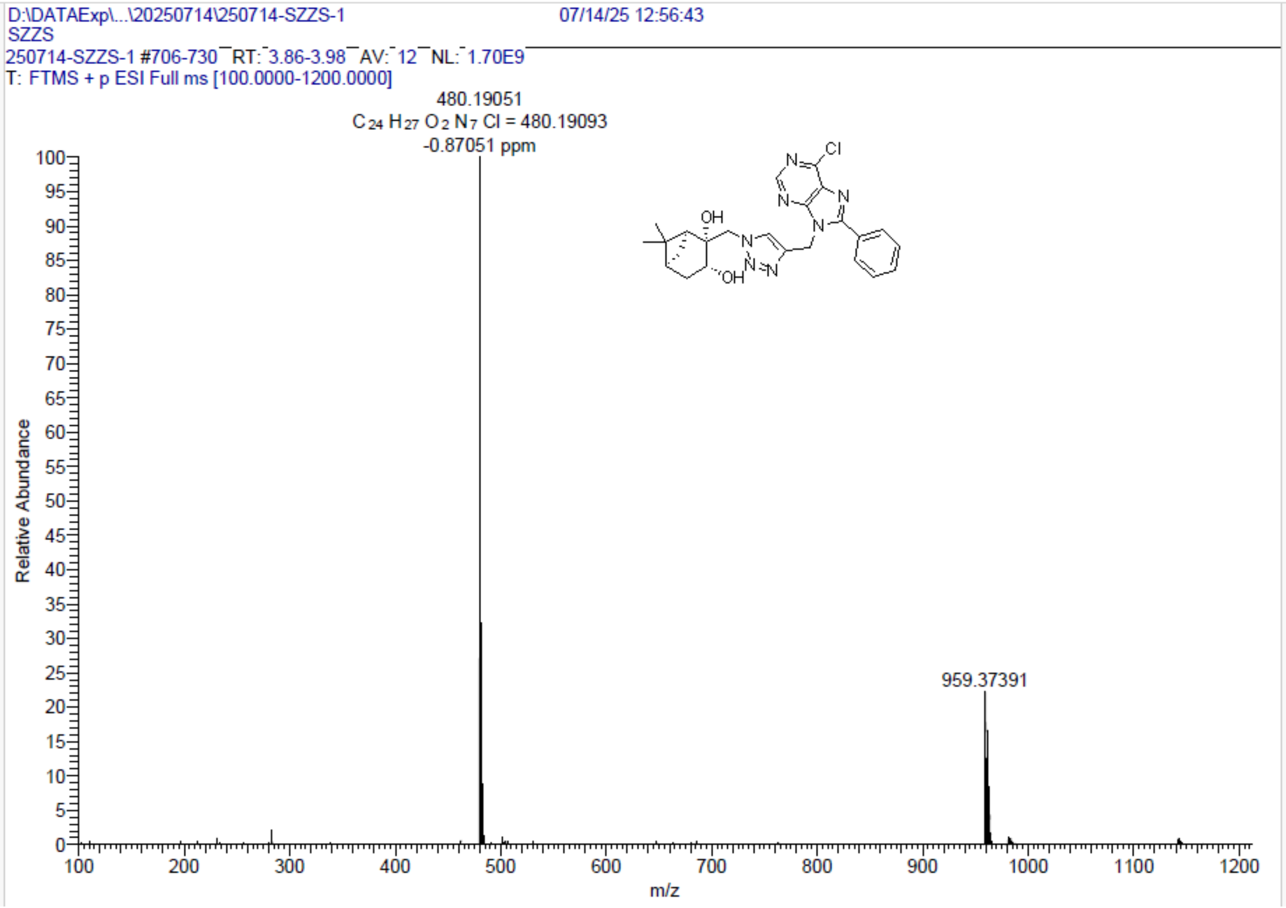

(1*R*,2*S*,3*S*,5*R*)-2-((4-((6-Chloro-8-phenyl-9*H*-purin-9-yl)methyl)-1*H*-1,2,3-triazol-1-yl)methyl)-6,6-dimethylbicyclo[3.1.1]heptane-2,3-diol **(–)-30**

Figure S 212. HRMS spectrum of compound **(–)-30**

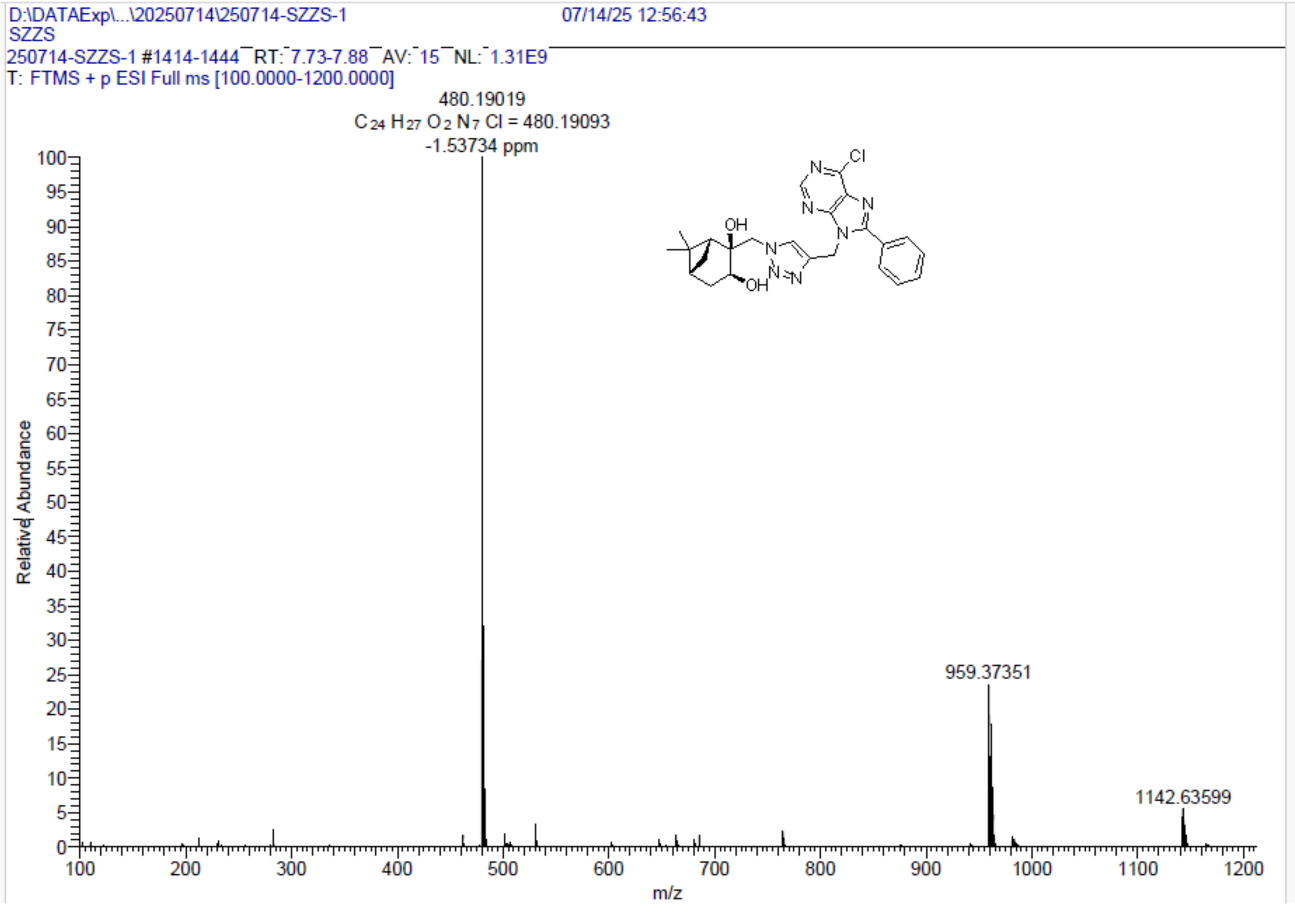

(1*S*,2*R*,3*R*,5*S*)-6,6-Dimethyl-2-((4-((6-phenyl-9*H*-purin-9-yl)methyl)-1*H*-1,2,3-triazol-1-yl)methyl)bicyclo[3.1.1]heptane-2,3-diol **(+)-31**

Figure S 213. HRMS spectrum of compound **(+)-31**

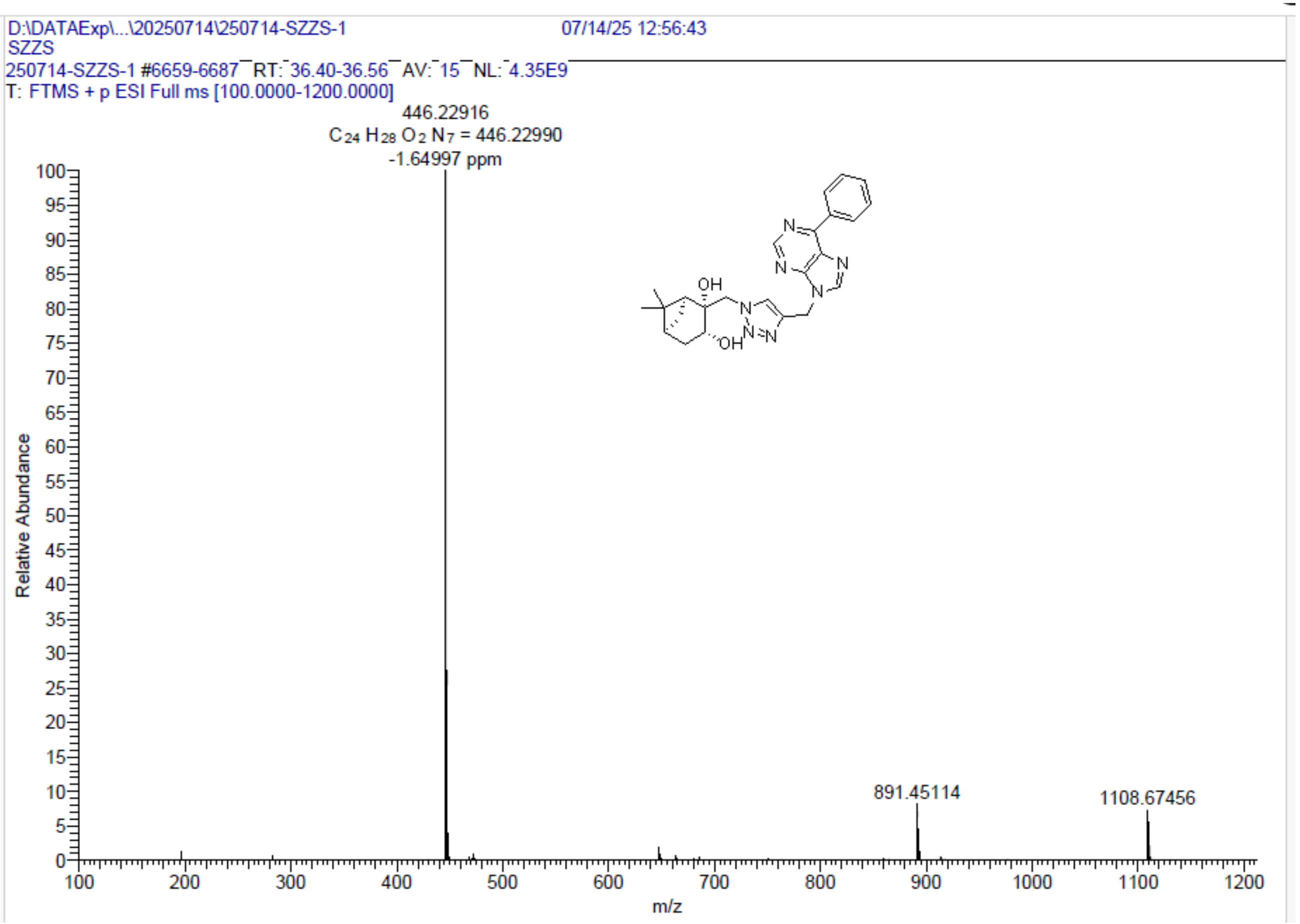

(1*R*,2*S*,3*S*,5*R*)-6,6-Dimethyl-2-((4-((6-phenyl-9*H*-purin-9-yl)methyl)-1*H*-1,2,3-triazol-1-yl)methyl)bicyclo[3.1.1]heptane-2,3-diol **(–)-31**

Figure S 214. HRMS spectrum of compound **(–)-31**

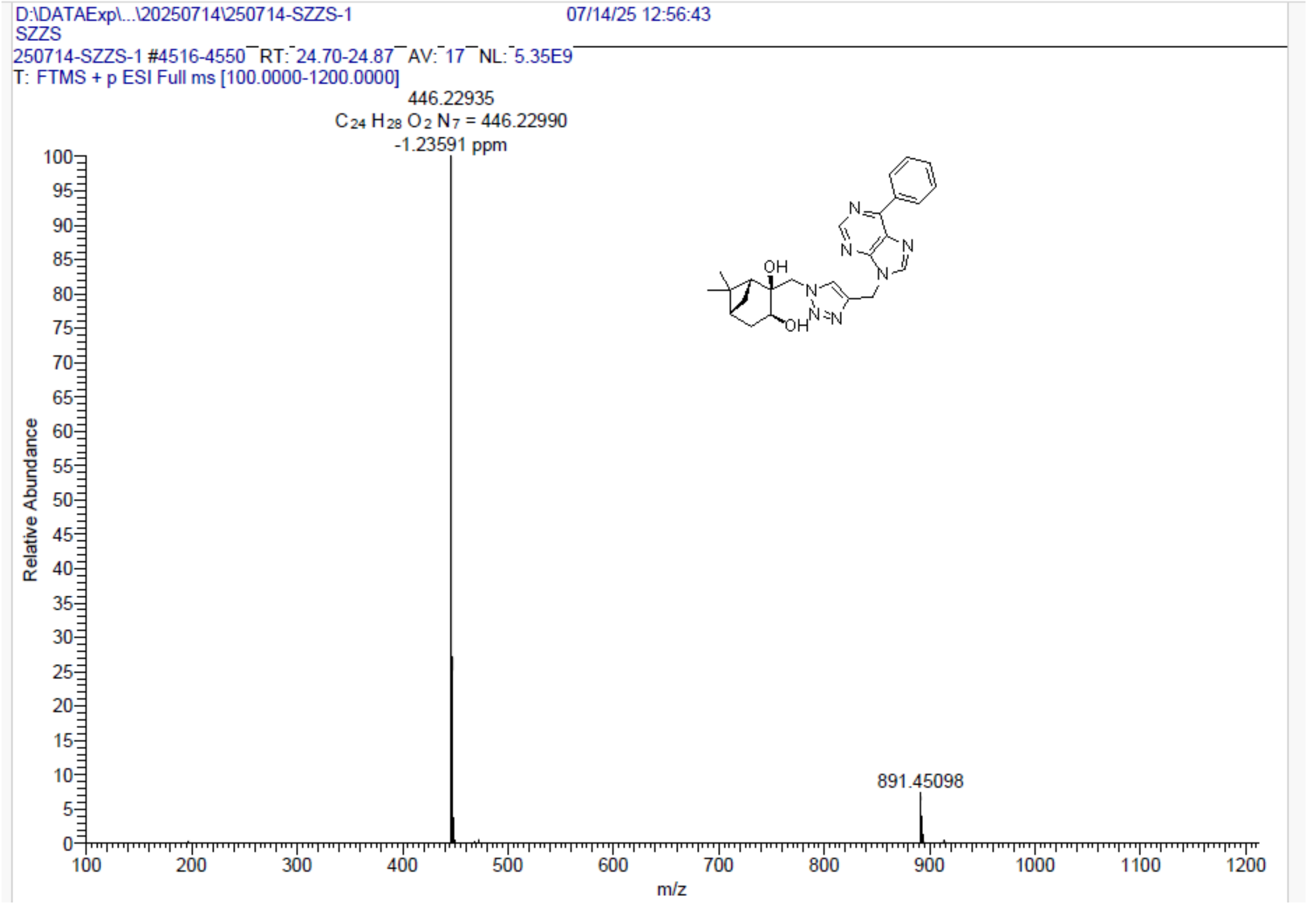

(1*S*,2*R*,3*R*,5*S*)-2-((4-((6-(Benzylamino)-9*H*-purin-9-yl)methyl)-1*H*-1,2,3-triazol-1-yl)methyl)-6,6-dimethylbicyclo[3.1.1]heptane-2,3-diol **(+)-32**

Figure S 215. HRMS spectrum of compound **(+)-32**

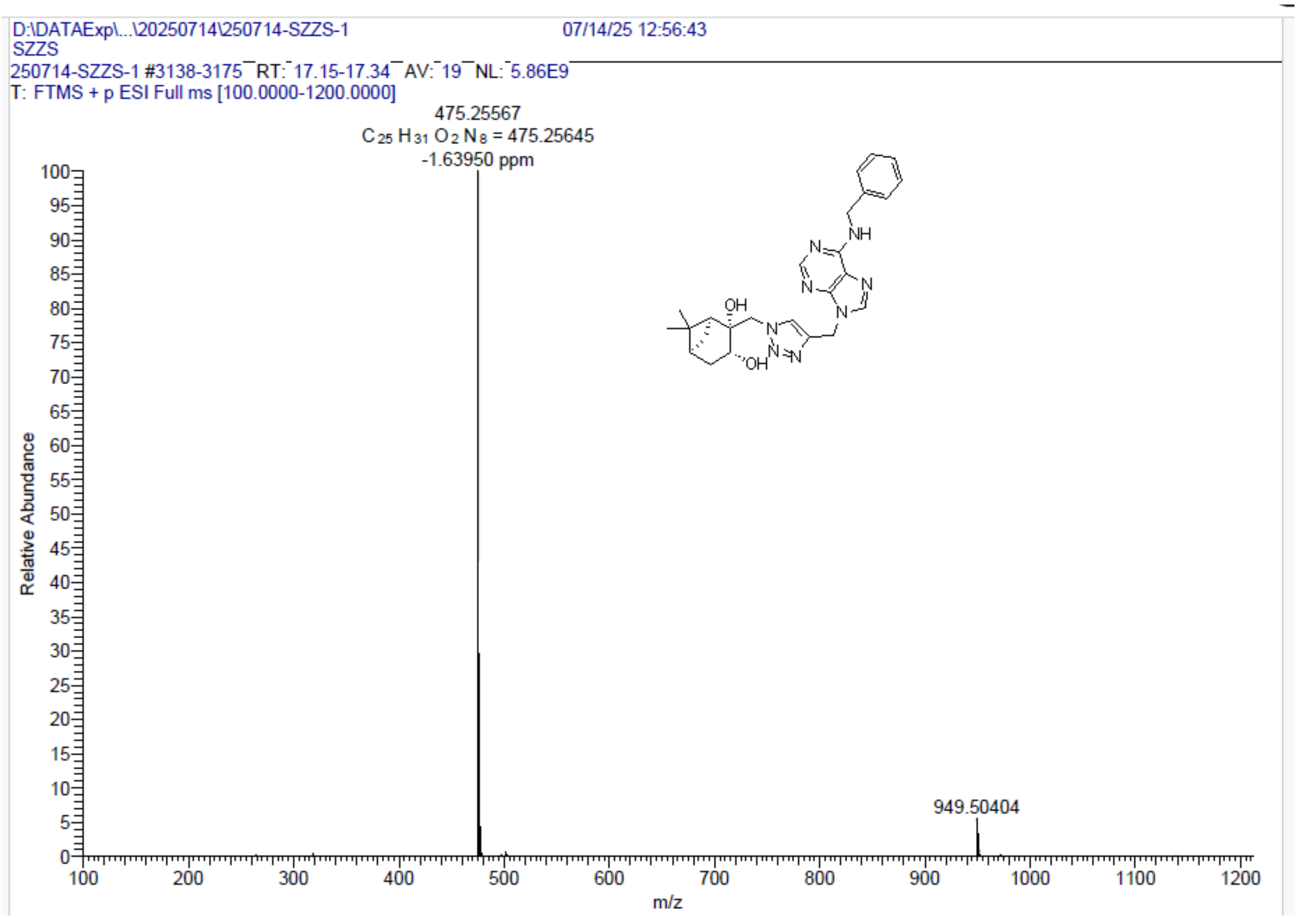

(1*R*,2*S*,3*S*,5*R*)-2-((4-((6-(Benzylamino)-9*H*-purin-9-yl)methyl)-1*H*-1,2,3-triazol-1-yl)methyl)-6,6-dimethylbicyclo[3.1.1]heptane-2,3-diol **(–)-32**

Figure S 216. HRMS spectrum of compound **(–)-32**

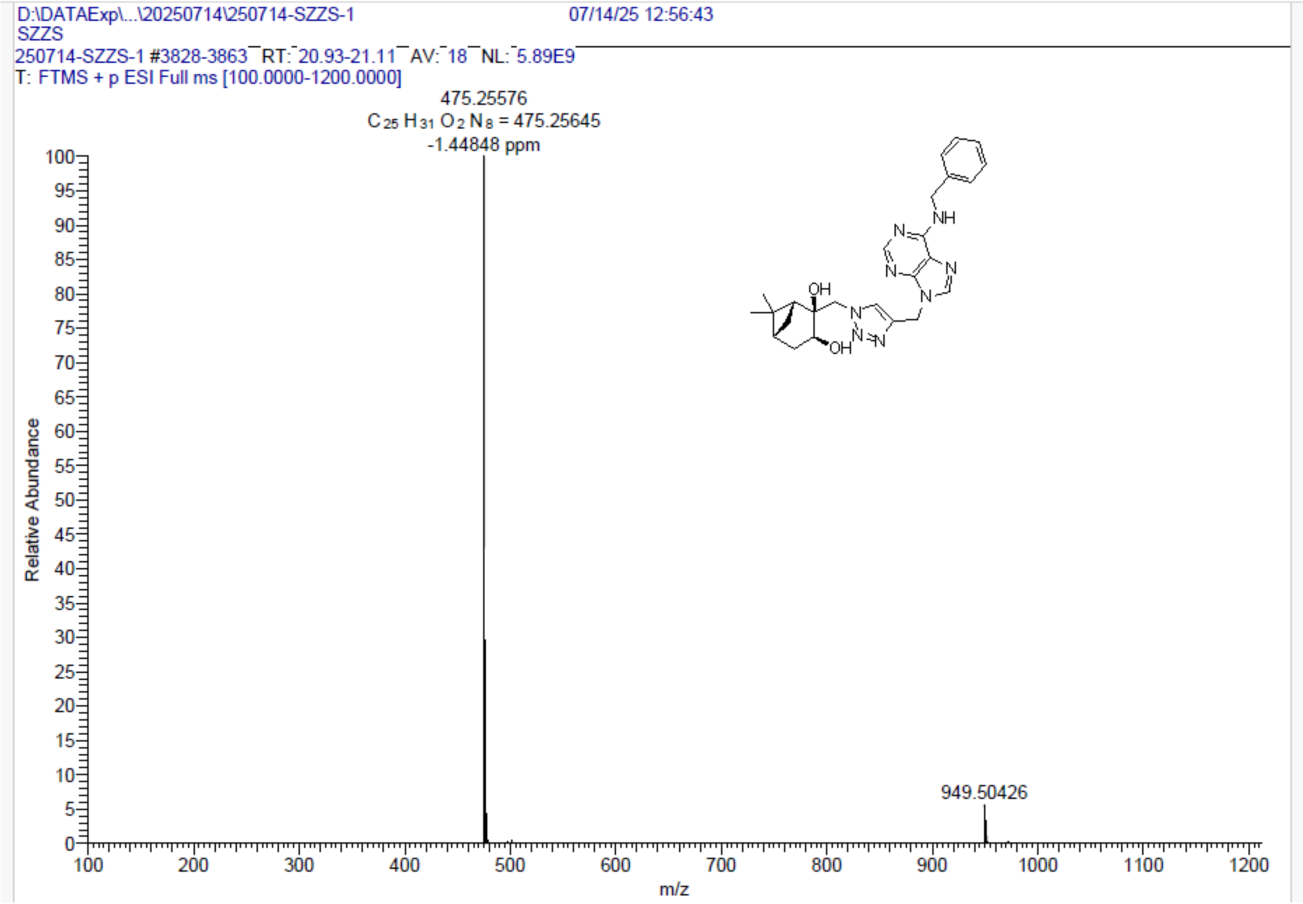

(1*S*,2*R*,3*R*,5*S*)-6,6-Dimethyl-2-((4-((6-((4-(trifluoromethyl)phenyl)amino)-9*H*-purin-9-yl)methyl)-1*H*-1,2,3-triazol-1-yl)methyl)bicyclo[3.1.1]heptane-2,3-diol **(+)-33**

Figure S 217. HRMS spectrum of compound **(+)-33**

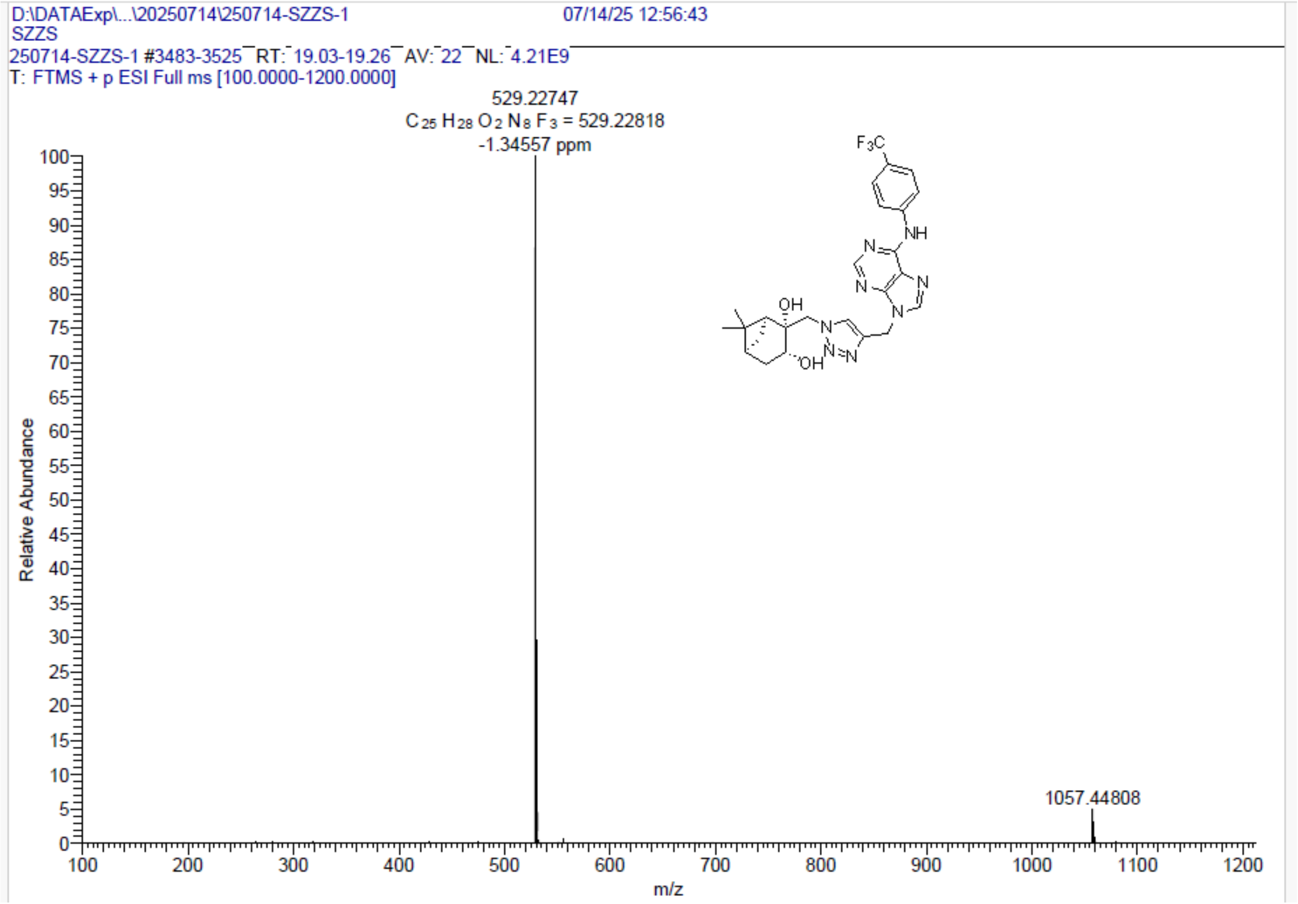

(1*R*,2*S*,3*S*,5*R*)-6,6-Dimethyl-2-((4-((6-((4-(trifluoromethyl)phenyl)amino)-9*H*-purin-9-yl)methyl)-1*H*-1,2,3-triazol-1-yl)methyl)bicyclo[3.1.1]heptane-2,3-diol **(–)-33**

Figure S 218. HRMS spectrum of compound **(–)-33**

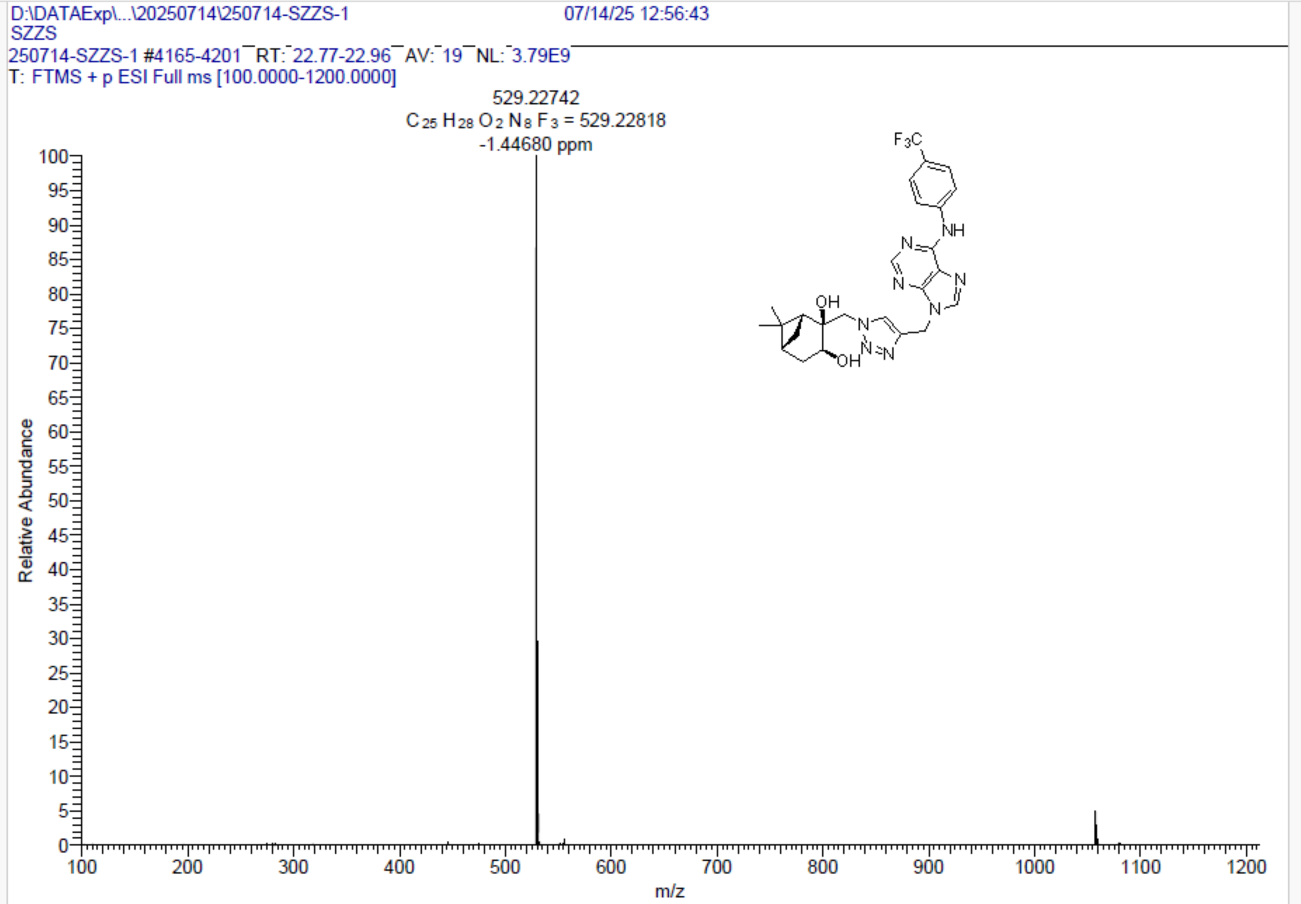

Supplement: Supplementary file 1 [file ijms-26-11705-s001.zip › ijms-3999210-supplementary.pdf]
